# Leveraging Trifluoromethylated Benzyl Groups toward the Highly 1,2-*Cis*-Selective Glucosylation of Reactive Alcohols

**DOI:** 10.1021/acs.orglett.1c02947

**Published:** 2021-10-22

**Authors:** Dancan K. Njeri, Erik Alvarez Valenzuela, Justin R. Ragains

**Affiliations:** Department of Chemistry, Louisiana State University 232 Choppin Hall, Baton Rouge, Louisiana 70806, United States

## Abstract

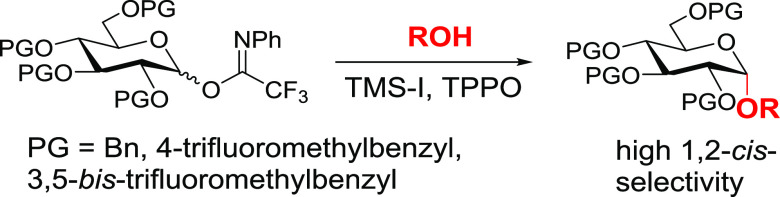

Here, we demonstrate that substitution of the benzyl groups of glucosyl imidate donors with trifluoromethyl results in a substantial increase in 1,2-*cis*-selectivity when activated with TMS-I in the presence of triphenylphosphine oxide. Stereoselectivity is dependent on the number of trifluoromethyl groups (4-trifluoromethylbenzyl vs 3,5-*bis*-trifluoromethylbenzyl). Particularly encouraging is that we observe high 1,2-*cis*-selectivity with reactive alcohol acceptors.

The *O*-glycosylation of alcohols is the most intensively studied transformation in carbohydrate chemistry, and the number of variants as far as electrophiles (i.e., “glycosyl donors”), reagents, protecting groups, and auxiliaries are concerned serves as a testament to the difficulties that have been incurred.^1−4^ Further, the selective preparation of 1,2-*trans* and 1,2-*cis O*-glycosidic linkages (Scheme 1) is a critical aspect of *O*-glycosylation. Neighboring-group participation of 2-position esters, carbonates, and carbamates ensures 1,2-*trans* selectivity. This approach works so well as to be effective for the iterative synthesis of glycans on solid phase.^4^ Despite intensive investigation, the development of 1,2-*cis*-selective *O*-glycosylation has proven more difficult, and a dearth of automated approaches to 1,2-*cis*-glycoside-rich targets attests to this.^5^ There is sentiment that dissociative pathways are detrimental to the development of 1,2-*cis*-selective *O*-glycosylation, and successful methods appear to avoid them.^6^*While these approaches vary in their complexity, a “Holy Grail” of 1,2-cis-selective O-glycosylation strategies would be broadly applicable and characterized by simple design.*

**Scheme 1:**
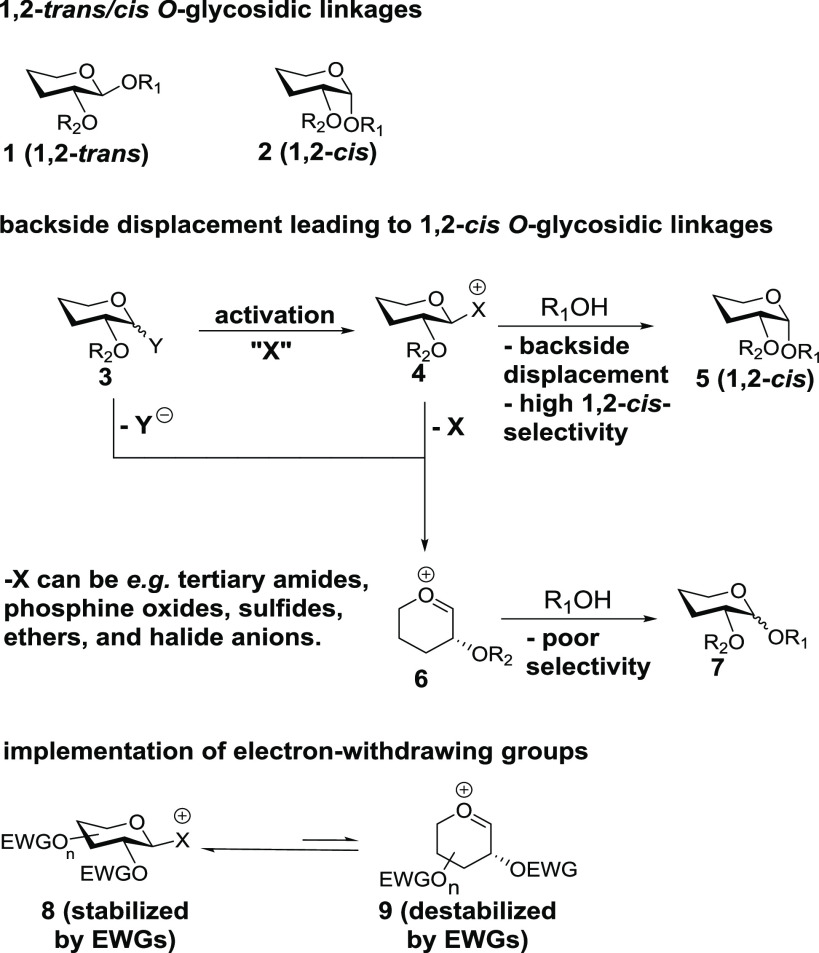
1,2-*Cis*-Selective Glycosylation by Backside Displacement

In hexose systems in which the 2-substituent is equatorial (e.g., glucose, galactose, and *N*-acetylglucosamine), the most simple approach is perhaps the backside displacement of an equatorial anomeric leaving group (Scheme 1, **4**→**5**). Such an approach could be effective when the anomeric leaving group consists of an additive “X” that either (a) “prefers” to be equatorial due to steric reasons^7^ or (b) confers greater reactivity when equatorial.^8,9^ Nevertheless, pitfalls exist. In particular, ionization of intermediates **4** to oxocarbenium ions **6** could provide leakage to dissociative pathways and erosion of selectivity.

A potential solution to this problem is the implementation of electron-withdrawing protecting groups that will (a) confer a high equilibrium constant *K* = [**8**]/[**9**] and (b) ensure that the backside displacement **4**→**5** can occur with high fidelity. There have been a small number of reports suggesting the utility of this strategy.^10−12^ Recently, we embarked on a study^13^ of a series of donors from our group known as 4-(4-methoxyphenyl)-3-butenylthioglycosides (MBTGs)^14a^ and 4-(4-methoxyphenyl)-4-pentenylthioglycosides (MPTGs)^14b^ in which we demonstrated that protection of glucose-derived MBTGs and MPTGs with *para*-substituted benzyl groups in which the substituent was F, Cl, or CF_3_ resulted in a steady improvement in 1,2-*cis*-selectivity relative to benzyl when activated with trifluoromethanesulfonic acid (HOTf) in 1,4-dioxane. Selectivity correlated with the Hammett σ parameter of each substituent, with 4-trifluoromethyl benzyl (CF_3_Bn) providing the highest selectivity. Most disappointing to us, however, was the unreliable 1,2-*cis*-selectivity incurred in our substrate scope study. In particular, very low selectivities were observed with highly reactive alcohol acceptors (e.g., 5.5:1 in favor of 1,2-*cis* with the acceptor *N*-carbobenzyloxy-3-amino-1-propanol).

In an effort to improve the selectivities from our initial report, we were intrigued by work from Mukaiyama^7c^ as well as Codée and co-workers’ α-glucan syntheses^15^ in which glucosyl *O*-imidates were activated by Lewis and protic acids in the presence of either DMF^8^ or triphenylphosphine oxide (TPPO).^7c,16^ In these systems, relatively electron-rich protecting groups were utilized. We were intrigued by the prospects of further improving 1,2-*cis*-selectivity through trifluoromethylated benzyl protecting groups. Herein, we demonstrate that 1,2-*cis*-selectivity improves in a manner dependent on the number of trifluoromethyl groups starting from glucosyl trichloroacetimidates (TCAIs) and *N*-phenyltrifluoroacetimidates (PTFAIs) when activated with iodotrimethylsilane (TMS-I) in the presence of TPPO. *Particularly exciting is the high 1,2-cis-selectivity incurred even with relatively reactive alcohol acceptors including those used as linker moieties.*(17)

In our initial study (Scheme 2), we implemented the glucosyl-*O*-trichloroacetimidates (TCAIs) **10** along with the reactive acceptor **12**. Employing 0.15 mmol of benzyl (Bn)-protected TCAI donor **10a** and TMS-I along with 6 equiv of TPPO in dichloromethane, we obtained a selectivity of 14:1 1,2-*cis*/1,2-*trans* (α/β, entry 1) of **13a**. Replacing the Bn with 4-trifluoromethylbenzyl (CF_3_Bn, **10b**) resulted in a dramatic increase in selectivity to 34:1 α/β (entry 2). Dilution of reaction mixtures of **10a**/**10b** and **12** under conditions that were otherwise identical to entries 1 and 2 resulted in comparable selectivities and decreases in yield which contrasts with our previous study^13^ (entries 3 and 4).

**Scheme 2:**
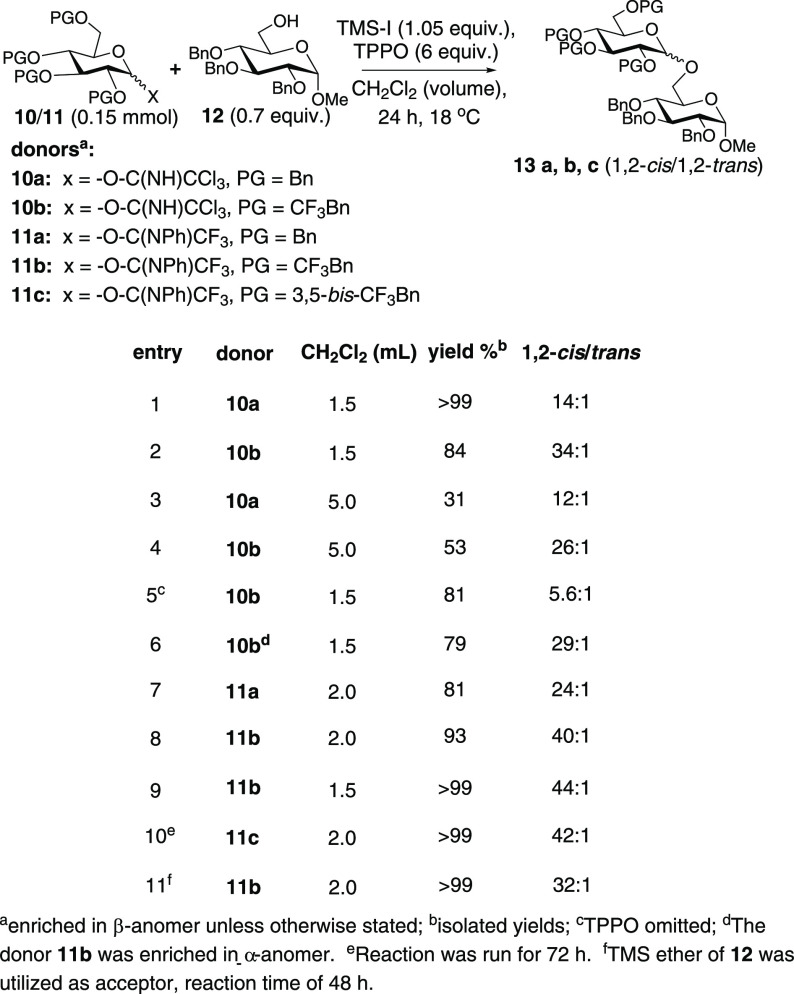
Protecting Group Screen/Optimization

Increasing the equivalents of TPPO to 15 resulted in incomplete consumption of acceptor after 24 h with similar selectivities as in entries 1 and 2 (see the Supporting Information). Likewise, an increase in equivalents of TMS-I from 1.05 to 2 or switching to 6 equiv of trimethylphosphine oxide or cyclohexyldiphenylphosphine oxide using donor **10b** did not provide improvements over entry 2 (see the Supporting Information). Finally, omission of TPPO resulted in dramatically reduced selectivity (5.6:1 α/β, entry 5). We were also intrigued by what effect the starting stereochemistry of donor **10b** might have on the stereochemical outcome. While the results in entries 1–4 were obtained with donor mixtures enriched in the β-TCAI, we prepared a mixture of **10b** enriched in α-TCAI and performed glycosylation under entry 2 conditions. The stereochemical outcome was similar (entry 6), and we attribute this to the relatively rapid (a few hours relative to the 24 h reaction time) formation of α-glycosyl iodide **α-25** (see Scheme 6 in addition to the Supporting Information) which then reacts slowly en route to glycosidic products.

Given the increased stability and decreased reactivity of PTFAIs relative to TCAIs, we were intrigued by the potential to effect increased selectivity. Implementation of Bn-protected PTFAI **11a** resulted in increased selectivity relative to **10a** which was still inferior to that of 4-CF_3_Bn-protected TCAI **10b** (entry 7, compare to entries 1 and 2). Implementation of donor **11b** (entry 8, compare to entry 2) resulted in an improvement (40:1 α/β) over analogue **11a**. While using 2 mL of CH_2_Cl_2_ in entries 7/8 in contrast to the 1.5 mL used in entries 1–6 was done for practical reasons (slow dissolution of substrates), implementation of 1.5 mL of CH_2_Cl_2_ (entry 9) did not provide substantially different yields or selectivities. In addition, we screened 3,5-bis*-*trifluoromethylbenzyl-protected **11c** which provided comparable selectivity to **11b** (entry 10) but would later prove useful for more “difficult” substrates than **12**. A final question regarding this set of transformations centered around the role of TMS ethers and HI derived from the reaction of **12** and TMSI. Thus (entry 11), the reaction of the TMS ether^18^ derived from **12** under conditions identical to entry 8 did not result in a significant change in stereoselectivity while significantly increasing reaction time. Any HI formed in these reactions appears to have little effect on yield and selectivity, while TMS ethers are less nucleophilic than alcohols.

Meanwhile, we had elected early on to evaluate TCAI donors **10a**/**b** with the poorly reactive acceptor **14** (Scheme 3). Implementing **10a** with acceptor **14** according to the entry 2 conditions in Scheme 2 resulted in a low yield of product **15a** but with no detected β-anomer (Scheme 3, entry 1). This likely reflects the poor reactivity of **14** which frequently correlates to high selectivity. Donor **10b** also provided poor yields and no detected β-anomer (entry 2). Codée had previously prescribed the use of the activator HOTf in the presence of DMF as the answer to poor reactivity on the part of hindered acceptors^15^ while the use of tertiary amide additives has often been prescribed to effect 1,2-*cis* selectivity.^8^ In switching to DMF (entries 3/4), we saw improvements in yield and no detected β-anomer. While our approach may be obviated for poorly reactive acceptors, these results are not surprising since less reactive acceptors tend to give higher 1,2-*cis* selectivity.^6^ Perhaps the most useful information to be gained from the Scheme 3 results is that leakage to dissociative pathways appears to pose a minimal threat in these systems. It has been suggested that decreased stereoselectivity upon moving from more reactive to less reactive acceptors could result from the reaction with oxocarbenium ions when more associative pathways have a prohibitive activation energy.^6a^

**Scheme 3:**
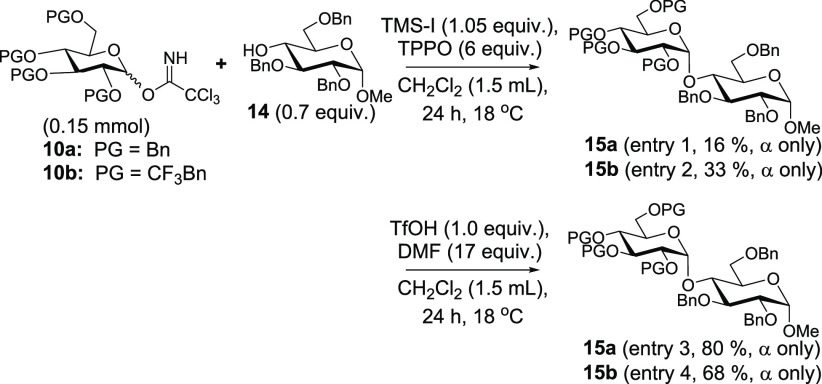
*O*-Glycosylation Studies with a Hindered Acceptor

To test the generality of our strategy, we conducted a substrate scope study (Scheme 4) using Bn-, CF_3_Bn-, and 3,5-bis-CF_3_Bn-protected donors **11** and conditions from entry 8 in Scheme 2. Using Bn-protected **11a** with the reactive acceptor *N*-carbobenzyloxy-3-aminopropan-1-ol, we obtained a high yield of **16a** in a ratio of 13:1 (α/β). As predicted, we observed an increase in selectivity to 23:1 (α/β) when implementing CF_3_Bn-protected **11b** (entry 1). In entry 2, we further demonstrated the efficacy of increasing numbers of trifluoromethyl groups when implementing **11a** (11:1 in favor of 1,2-*cis*), **11b** (16:1), and **11c** (31:1) with *N*-benzyl-*N*-carbobenzyloxy-5-aminopentan-1-ol. Similarly, donors **11b**,**c** with the acceptor 3-azidopropan-1-ol saw an increase from 11:1 as originally reported^15^ to 22:1 to 34:1 (α/β) as the number of trifluoromethyl groups was increased (entry 3). Implementation of **11c** requires longer reaction times (72 h) in contrast to the 24 h reaction time with **11a** and **11b**. It is also very significant that such high selectivities can be attained with relatively reactive acceptors such as these, and we are intrigued by the potential implementation of this or similar electron-withdrawing group strategies toward solid-phase and automated synthesis where highly 1,2-*cis*-selective installation of linker moieties is elusive.^17^

**Scheme 4:**
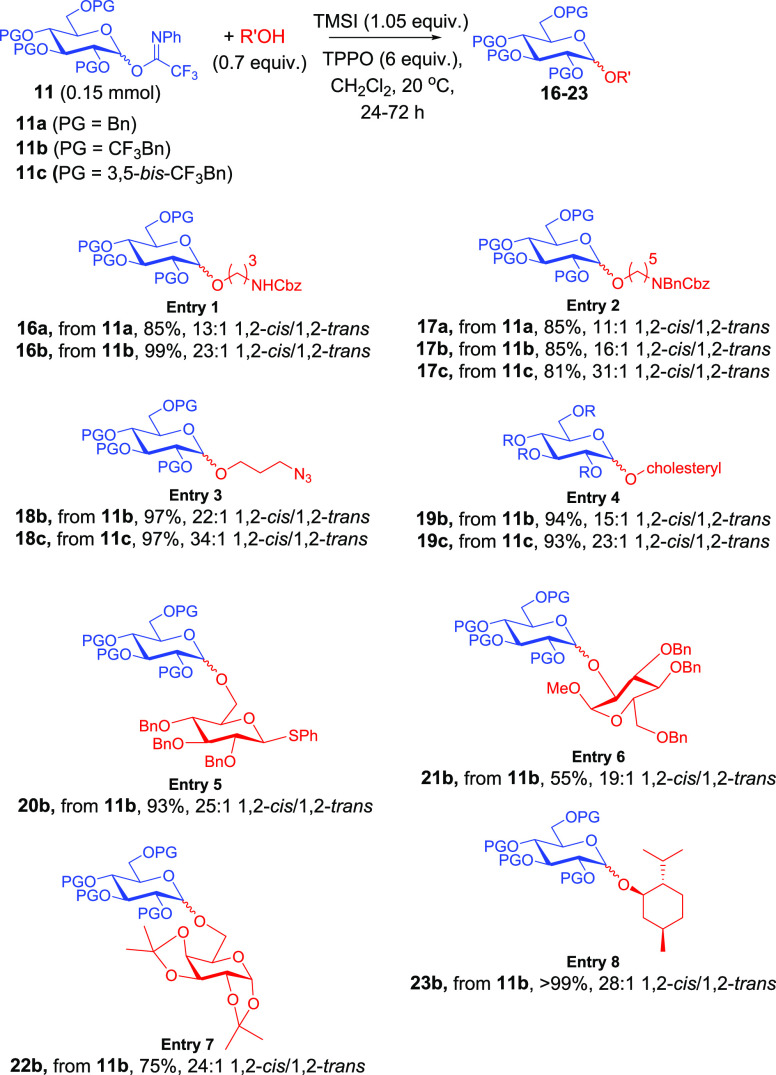
Substrate Scope Study

In continuing our study, we provided a direct comparison of CF_3_Bn and 3,5-bis-CF_3_Bn in entry 4 with cholesterol. Whereas **11b** afforded a somewhat disappointing 15:1 ratio, **11c** saw an improvement to 23:1. We also demonstrated highly 1,2-*cis*-selective *O*-glycosylation (25:1) with thioaglycone-containing acceptor to generate **20b** (entry 5). The C2-position of glucose also resulted in encouraging selectivity (19:1) but modest yield when reacted with **11b** (entry 6). Further, the acid-sensitive acceptor galactose diacetonide underwent a highly selective (24:1) *O*-glycosylation with **11b** (entry 7). Finally, the reactive acceptor menthol underwent *O*-glycosylation with donor **11b** in a ratio of 28:1 in favor of 1,2-*cis*. *That we were able to attain selectivities in excess of 20:1 (and approaching or greater than 30:1 in a number of cases) with a simple strategy implementing a substituted benzyl protecting groups with reactive acceptors at room temperature is a significant accomplishment.*

A final set of demonstrations includes the hydrogenolytic removal of 3,5-bis-CF_3_Bn groups and a 1 mmol-scale procedure. We demonstrated (Scheme 5) that hydrogenolysis with Pd(OH)_2_ resulted in an 82% yield of **24** using previously reported conditions.^13,19^ Further, we demonstrate the conversion of **11c** and **12** to **13c** with high selectivity on 1 mmol scale (Scheme 5).

**Scheme 5:**
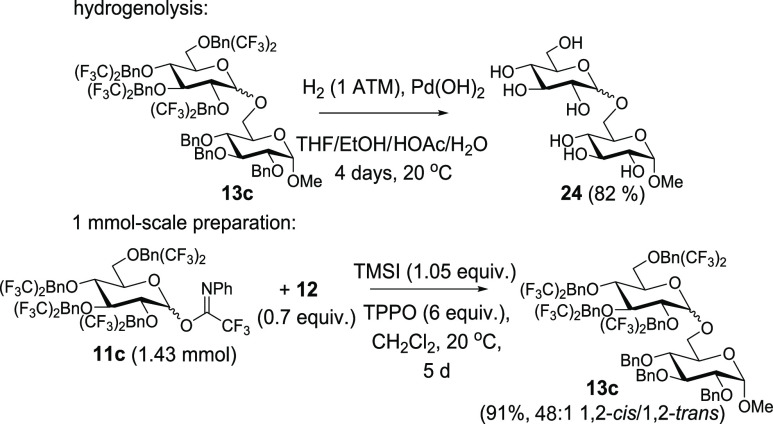
Hydrogenolytic Removal of 3,5-Bis-CF_3_Bn Groups/1 mmol Scale Preparation

Based on our observations here and the observations of others,^7c,9,15,16^ we provide the mechanistic hypothesis depicted in Scheme 6. Reaction of imidates (e.g., **11**) with TMS-I results in the conversion to a mixture of glycosyl iodides **25** that favors **α-25** dramatically. While it is tempting to suggest that reaction of alcohol with early intermediates in this process may result in an erosion of stereoselectivity, we note that preformation of the mixture of **25** followed by addition of alcohol acceptor does not provide significantly different results from those of Scheme 2, entry 8, using **11a** (see Table S1). Interception of **α-25** by TPPO may result in the formation of the intermediate **26** proposed (but not observed) by Codée.^15^ Our efforts to observe this and related intermediates by mass spectrometry failed. Reaction of **26** with alcohol is facile and results in formation of 1,2-*cis*-glycoside **α-27**. This scenario explains the formation of **α-27**; however, formation of 1,2-*trans***β-27** as the minor product deserves its own discussion.

**Scheme 6:**
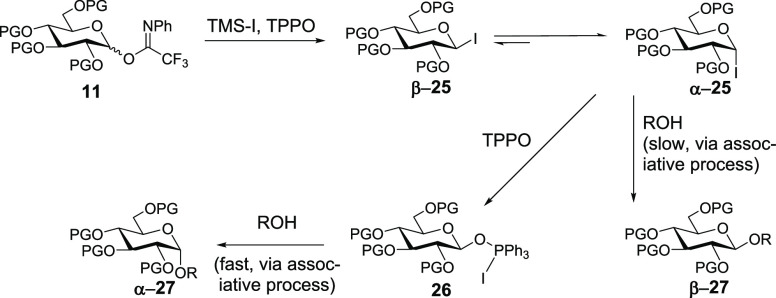
Mechanistic Hypothesis

While it is tempting to argue that 1,2-*trans* product **β-27** is formed according to a dissociative process, our results from Scheme 3 suggest otherwise. The high 1,2-*cis* selectivity there suggests that dissociative pathways are minor. While the steric bulk at the C4 hydroxyl of **14** is expected to slow any associative backside process, significant ionization leading to solvent-separated ion pairs should lead to facile reaction with **14** and an erosion of stereoselectivity, an outcome that is not observed. Instead, “top-side” attack of alcohol is likely to occur on a contact ion pair derived from **α-25** to generate **β-27**. The origin of increased selectivity in switching protecting groups from Bn to CF_3_Bn to 3,5-bis-CF_3_Bn may be due to increasing barriers to contact ion pair formation caused by electron-withdrawing effects rather than an increased rate in the conversion of **26** to **α-27**. Such deactivation will have a greater effect on less-reactive **α-25** than more-reactive **26** while the overall decrease of reaction rate in going from **11b** to **11c** attests to the deactivation.

As we were nearing completion of the present study, we became aware of a recent study published by Zhang et al.^20^ In their elegant work, they demonstrate that replacement of the 6-position benzyl of **11a** (Scheme 2) with 4-oxopentanoyl results in high 1,2-*cis* selectivity (>20:1 α/β) under nearly identical conditions (TMSI, TPPO, CH_2_Cl_2_) as those reported herein.

In conclusion, we have demonstrated a consistent increase in 1,2-*cis* selectivity in the glycosylation of relatively highly reactive alcohols with glucosyl TCAIs and PTFAIs when benzyl, 4-trifluoromethylbenzyl, and 3,5-bis-trifluoromethylbenzyl protecting groups are implemented. The simple design means that this could have important implications in the development of multistep oligosaccharide synthesis and even automated synthesis. While trifluoromethylated benzyl groups proved effective herein, it is probable that alternative electron-withdrawing protecting groups, substitution patterns, Lewis basic additives, and activation strategies can be implemented using this strategy. Inhibiting the formation of contact ion pairs while avoiding the deactivation that leads to poorly reactive intermediates will provide an ongoing challenge. These factors will be the subject of ongoing investigation.

## Supporting Information Available

Experimental procedures, characterization data, ^1^H and ^13^C spectra (PDF)

## References

[ref1] Nitz M.; Bundle D. R. Glycosyl Halides in Oligosaccharide Synthesis. In Glycoscience: Chemistry and Biology; Fraser-Reid B. O., Tatsuta K., Thiem J., Eds.; Springer: Berlin, 2001; pp 1498–1542.

[ref2] aCodée J. D. C.; Litjens R. E. J. N.; van den Bos K. J.; Overkleeft H. S.; van der Marel G. A. Thioglycosides in Sequential Glycosylation Strategies. Chem. Soc. Rev. 2005, 34, 769–782. 10.1039/b417138c. 16100617

[ref3] aSchmidt R. R.; Jung K. H. Trichloroacetimidates. In Carboydrates in Chemistry and Biology; Ernst B., Hart G. W., Sinay P., Eds.; Wiley-VCH: Weinheim, 2000; pp 5–59.

[ref4] aGuberman M.; Seeberger P. H. Automated Glycan Assembly: A Perspective. J. Am. Chem. Soc. 2019, 141, 5581–5592. 10.1021/jacs.9b00638. 30888803 PMC6727384

[ref5] aZhu Y.; Delbianco M.; Seeberger P. H. Automated Assembly of Starch and Glycogen Polysaccharides. J. Am. Chem. Soc. 2021, 143, 9758–9768. 10.1021/jacs.1c02188. 34115468 PMC8267850

[ref6] aNigudkar S. S.; Demchenko A. V. Stereocontrolled 1,2-*cis*-Glycosylation as the Driving Force of Progress in Synthetic Carbohydrate Chemistry. Chem. Sci. 2015, 6, 2687–2704. 10.1039/C5SC00280J. 26078847 PMC4465199

[ref7] aPark J.; Kawatkar S.; Kim J.-H.; Boons G.-J. Stereoselective Glycosylations of 2-Azido-2-Deoxy-Glucosides Using Intermediate Sulfonium Ions. Org. Lett. 2007, 9, 1959–1962. 10.1021/ol070513b. 17432867 PMC2533432

[ref8] aLu S.-R.; Lai Y.-H.; Chen J.-H.; Liu C.-Y.; Mong K.-K. T. Dimethylformamide: An Unusual Glycosylation Modulator. Angew. Chem., Int. Ed. 2011, 50, 7315–7320. 10.1002/anie.201100076. 21688356

[ref9] Lemieux R. U.; Hendriks K. B.; James K. Halide Ion Catalyzed Glycosidation Reactions. J. Am. Chem. Soc. 1975, 97, 4056 10.1021/ja00847a032.

[ref10] Moons S. J.; Mensink R. A.; Bruekers J. P. J.; Vercammen M. L. A.; Jansen L. M.; Boltje T. J. α-Selective Glycosylation with β-Glycosyl Sulfonium Ions Prepared via Intramolecular Alkylation. J. Org. Chem. 2019, 84, 4486–4500. 10.1021/acs.joc.9b00022. 30808170 PMC6454400

[ref11] Zheng Z.; Zhang L. Gold-Catalyzed Synthesis of α-D-Glucosides Using an *o*-Ethynylphenyl β-D-1-Thioglucoside Donor. Carbohydr. Res. 2019, 471, 56–63. 10.1016/j.carres.2018.10.010. 30439547 PMC6358439

[ref12] Zulueta M. M. L.; Lin S.-Y.; Lin Y.-T.; Huang C.-J.; Wang C.-C.; Ku C.-C.; Shi Z.; Chyan C.-L.; Irene D.; Lim L.-H.; Tsai T.-I.; Hu Y.-P.; Arco S. D.; Wong C.-H.; Hung S.-C. α-Glycosylation by D-Glucosamine-Derived Donors: Synthesis of Heparosan and Heparin Analogues That Interact with Mycobacterial Heparin-Binding Hemagglutinin. J. Am. Chem. Soc. 2012, 134, 8988–8995. 10.1021/ja302640p. 22587381

[ref13] Njeri D. K.; Pertuit C. J.; Ragains J. R. 1,2-*cis*-Selective Glucosylation Enabled by Halogenated Benzyl Protecting Groups. Org. Biomol. Chem. 2020, 18, 2405–2409. 10.1039/D0OB00373E. 32195525

[ref14] aLacey K. D.; Quarels R. D.; Du S.; Fulton A.; Reid N. J. Acid-Catalyzed *O*-Glycosylation with Stable Thioglycoside Donors. Org. Lett. 2018, 20, 5181–5185. 10.1021/acs.orglett.8b02125. 30148367

[ref15] Wang L.; Overkleeft H. S.; van der Marel G. A.; Codée J. D. C. Reagent Controlled Stereoselective Synthesis of α-Glucans. J. Am. Chem. Soc. 2018, 140, 4632–4638. 10.1021/jacs.8b00669. 29553729 PMC5890317

[ref16] Mukaiyama T.; Kobashi Y. Highly α-Selective Synthesis of Disaccharide Using Glycosyl Bromide by the Promotion of Phosphine Oxide. Chem. Lett. 2004, 33, 10–11. 10.1246/cl.2004.10.

[ref17] Schumann B.; Parameswarappa S. G.; Lisboa M. P.; Kottari N.; Guidetti F.; Pereira C. L.; Seeberger P. H. Nucleophile-Directed Stereocontrol Over Glycosylations Using Geminal-Difluorinated Nucleophiles. Angew. Chem., Int. Ed. 2016, 55, 14431–14434. 10.1002/anie.201606774. 27735117

[ref18] Sati G. C.; Martin J. L.; Xu Y.; Malakar T.; Zimmerman P. M.; Montgomery J. Fluoride Migration Catalysis Enables Simple, Stereoselective, and Iterative Glycosylation. J. Am. Chem. Soc. 2020, 142, 7235–7242. 10.1021/jacs.0c03165. 32207615 PMC7228505

[ref19] Bensel N.; Klär D.; Catala C.; Schneckenburger P.; Hoonakker F.; Goncalves S.; Wagner A. A Chemometric Approach to Map Reaction Media Chemoselectivity: Example of Selective Debenzylation. Eur. J. Org. Chem. 2010, 2010, 2261–2264. 10.1002/ejoc.200901497.

[ref20] Zhang Y.; He H.; Chen Z.; Huang Y.; Xiang G.; Li P.; Yang X.; Lu G.; Xiao G. Merging Reagent Modulation and Remote Anchimeric Assistance for Glycosylation: Highly Stereoselective Synthesis of α-Glycans up to a 30-mer. Angew. Chem., Int. Ed. 2021, 60, 12597–12606. 10.1002/anie.202103826. 33763930

