# Supplementary material for: Leveraging Trifluoromethylated Benzyl Groups toward the Highly 1,2-Cis-Selective Glucosylation of Reactive Alcohols
Source: Org Lett. 2021 Oct 22;23(21):8214–8. doi: 10.1021/acs.orglett.1c02947 (PMC8576833; doi:10.1021/acs.orglett.1c02947)
Supplement: Supplementary file 1 — ol1c02947_si_001.pdf [file ol1c02947_si_001.pdf]

Supplemental Information

Leveraging Trifluoromethylated Benzyl Groups Toward the Highly 1,2-*cis*-Selective Glucosylation of Reactive Alcohols

Dancan K. Njeri, Erik Alvarez Valenzuela, and Justin R. Ragains

Department of Chemistry

Louisiana State University

232 Choppin Hall

Baton Rouge, LA 70803

Table of Contents

**General Information**	**S3-4**
**Preparation/Characterization of Glucosyl Imidate Donors**	**S4-14**
**Glycosylation Procedures**	**S14-15**
**Optimization Table**	**S16**
**Information on Alcohol Acceptors**	**S17**
**Representative Procedures/Characterization for Glycosylation of 12 with 10/11**	**S18-24**
**Representative Procedures for Glycosylation of 14 with 10**	**S24-28**
**Procedures/Characterization for Substrate Scope Study**	**S28-49**
**Hydrogenolysis Procedure (3,5-*bis*-CF_3Bn Removal)**	**S49-50**
**1 mmol-Scale Preparation Procedure**	**S50-51**
**NMR Study Procedure (*in-situ* generation of glycosyl iodide α-25)**	**S51**
**References**	**S51-52**
**NMR Spectra (^{1}H, ^{13}C, ^{19}F) For New Compounds/Glycosylation Products**	**S53-131**
**NMR (^{1}H, ^{19}F) Estimation of Anomeric Ratios**	**S132-177**
**NMR Study (*in-situ* generation of glycosyl iodide α-25)**	**S167**

GENERAL INFORMATION

All reactions were performed under N_2 atmosphere which was achieved by vacuum purge backfill three times. Dried solvents (CH_2Cl_2, CH_3CN, Et_2O, THF, and DMF) were used directly from a PureSolv 400-5 solvent purification system. Reagents were purchased from commercial sources (Alfa Aesar, Acros Organics, Chem Impex, Matrix scientific (most *p*-substituted benzyl bromides), Sigma Aldrich, TCI). Column chromatography was performed using silica gel (60 Å) purchased from SiliCyle. Preparative TLC was performed on 60 Å silica gel with fluorescent indicator on a glass plate (Agela Technologies). Analytical TLC was performed using 60 Å silica gel with F254 indicator on aluminum sheets (Merck). Compound visualization on TLC was performed using a hand-held UV hand lamp and/or staining with anisaldehyde. ^{1}H NMR and ^{13}C NMR were performed using a Bruker AV-400 or a Bruker AV-500 NMR spectrometer. HRMS was performed using an Agilent 6210 electrospray time-of-flight mass spectrometer. Optical rotation values were obtained using a JASCO P-2000 instrument. Deuterated solvents were obtained from Cambridge Isotope Labs.

Determining Anomeric ratios.

For ^{1}H NMR of anomeric mixtures in both the crude and purified samples, the number of scans used was 16 while relaxation delays were set to 20 seconds. *Alpha:beta* ratios of glycosylation product mixtures were determined using ^{1}H NMR or ^{19}F integration of distinct signals from both the *alpha* and *beta* products. In the case of glycosylation reactions using acceptor **12** bearing a free hydroxyl at C6 and a methyl aglycone at the reducing end, anomeric ratios were determined according to the following set of commands using the GSD algorithm (deconvolution) in MestReNova:

1) Phase correction- Processing> Phase correction> Automatic
2) Baseline correction- Processing> Baseline> Full auto (Bernstein polynomials)
3) Analysis > Peak picking > options = Method - GSD, Refinement level – 5 fitting, Optimized for peaks – average. Then Ok.
4) Analysis > Integration > Options = Calculation method – Sum, Source – autodetect, Algorithm- peak picking, Minimum area – 3%. Then Ok.
5) Analysis > Peak picking > Automatic
6) View > Tables > Peaks.

A GSD table containing all the peaks with their respective height, width and area is then generated. For “CF_3Bn-protected glycosylation products, we analyzed reducing-end aglycone methyl signals from both the *alpha* and *beta* products which were located at 3.35-3.36 ppm for *alpha* and 3.32-3.33 ppm for *beta* when using acceptor **12.** Areas of these signals were used to determine anomeric ratios.

PREPARATION OF GLUCOSYL IMIDATE DONORS

Donors 10a (2,3,4,6-tetra-*O*-benzylglucosyl trichloroacetimidate),[2] 10b (2,3,4,6-tetra-*O*-(4-trifluoromethyl)benzylglucosyl trichloroacetimidate),[6] and 11a (2,3,4,6-tetra-*O*-benzylglucosyl *N*-phenyltrifluoroacetimidate)[2] were synthesized according to known procedures.

Synthesis of *N*-phenyltrifluoroacetimidate glucosyl donors with *p*-trifluoromethylbenzyl and 3,5-*bis*-trifluoromethylbenzyl protecting groups (11b, 11c)

Synthesis plan:

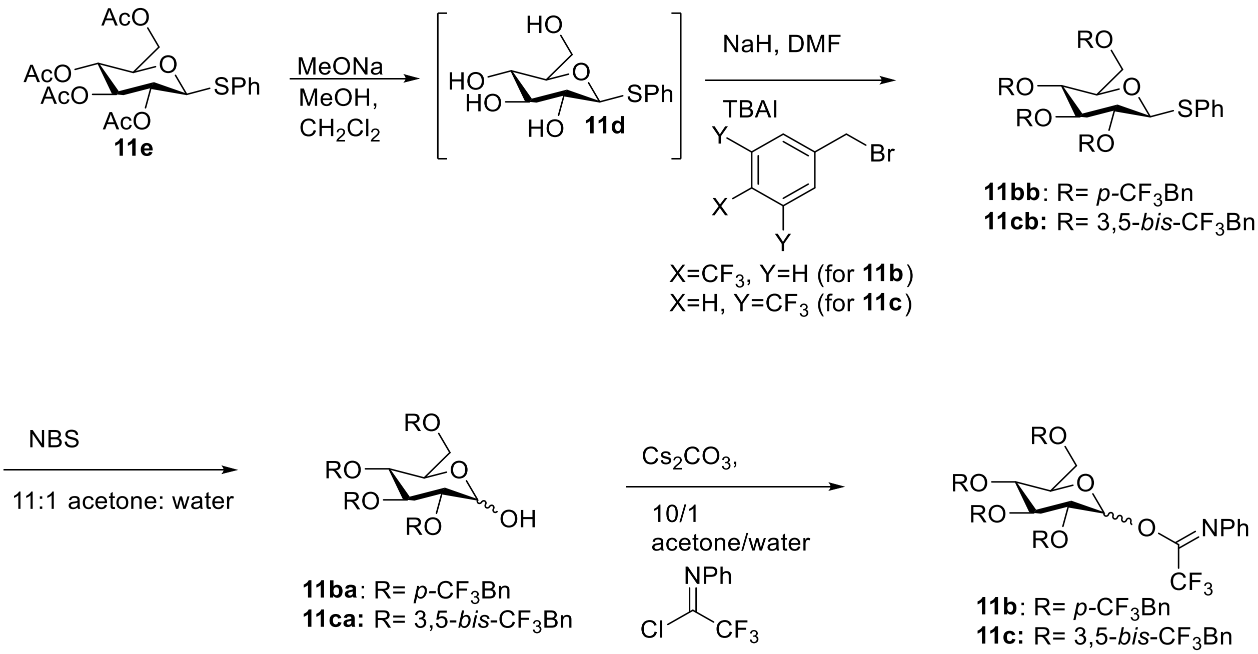

Synthesis of **11bb.**

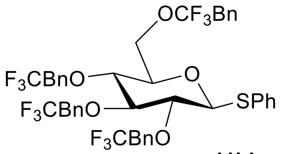

11bb

To 3.31 g of **11e**[14] (7.51 mmol) in a R.B.F with a magnetic stir bar, 30.0 ml of methanol was added followed by 10.0 ml of dichloromethane. The R.B.F was capped with a septum. Three cycles of nitrogen purge backfill were performed followed by dropwise addition of 0.5 ml of 5 M NaOMe. After 30 min, TLC showed that the reaction was complete. 1.43 g of Dowex ® 50WX8 200-400 mesh ion-exchange resin (Acros Organics) was added to neutralize the NaOMe. The mixture was filtered through celite and the filter cake was rinsed with 20.0 ml of methanol. Collected solution was concentrated then co-evaporated with 2.0 ml of toluene twice to give an off-white powder (**11d**). To the powder (**11d**) in R.B.F with a stir bar, 14.62 g of *p*-trifluoromethylbenzyl bromide (61.16 mmol) was added followed by 80.0 ml of DMF. 0.88 g of TBAI was then added. After all the solids had dissolved, the R.B.F was placed in an ice bath and cooled to 0 °C. Excess

60% NaH suspended in mineral oil (8.61 g) was then added carefully. After 10 min, the reaction was allowed to warm to room temperature under N_2 atmosphere and stirred for 16 hrs. The reaction was re-cooled to 0 °C and quenched by dropwise addition of water until gas evolution ceased. 200.0 ml of EtOAc was added and the contents transferred to a separatory funnel. 800.0 ml of water was then added, the contents were shaken vigorously to get rid of DMF and then allowed to stand to ensure separation of aqueous and organic layer. The organic layer was dried using Na_2SO_4 then concentrated to give a crude product which was purified using flash chromatography (5-15% EtOAc/hexanes) to give 5.92 g (6.54 mmol) of **11bb** as a white solid (87% two steps).

^{1}H NMR (500 MHz, $CDCl_3$) δ 7.63-7.51 (m, 10H), 7.49-7.40 (m, 4H), 7.34-7.25 (m, 7H), 5.00 (d, J = 11.2 Hz, 1H), 4.89 (d, J = 12.1 Hz, 1H), 4.86-4.79 (m, 2H), 4.77-4.65 (m, 4H), 4.61(d, J = 12.6 Hz, 1H), 3.86-3.76 (m, 2H), 3.75-3.68 (m, 2H), 3.60-3.51 (m, 2H). **^{13}C NMR (125 MHz, $CDCl_3$) δ** 142.2, 142.1, 141.8, 141.8, 133.4, 131.8, 130.2-129.8(m), 129.0, 127.8, 127.8, 127.5, 127.4, 127.2, 125.4, 125.4, 125.3, 125.3, 87.5, 86.7, 81.1, 78.9, 77.9, 74.7, 74.5, 74.0, 72.7, 69.1. **^{19}F NMR (471 MHz, $CDCl_3$) δ** -63.04 (s, 3F), -63.12 (s, 3F), -63.15 (s, 3F), -63.17 (s, 3F). **HRMS (ESI) m/z:** [M+Na]+ Calcd for $C_{44}H_{36,}F_{12}O_5SNa$ 927.1990; found 927.1997. **$[\alpha]_D^{25}$ =** +66.3° (*c* 1, CH_2Cl_2). **IR (cm^{-1})** 2874, 1326, 1264, 1165, 1111, 1066, 1019, 823 (*p*-disubstituted benzene), 733, 703.

Synthesis of **11ba**.

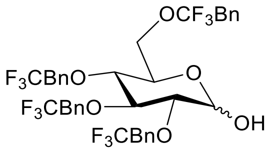

11ba

To 5.92 g (6.54 mmol) of **11bb** in a R.B.F with a stir bar, 60.0 ml of acetone was added followed by 5.5 ml of water. 3.71 g of NBS (20.8 mmol) was then added in one portion. The R.B.F was capped with a septum then three cycles of nitrogen purge backfill were performed and the reaction allowed to stir under nitrogen atmosphere. T.L.C showed the reaction was complete after 40 minutes. The contents were then concentrated followed by addition of 50.0 ml of dichloromethane. The resulting solution was then transferred to a separatory funnel and washed with 100.0 ml of water once. The organic layer was then concentrated and purified by flash chromatography (10-40% EtOAc/hexanes) to give 5.30 g (6.52 mmol) of **11ba** as a colorless gum (>99% yield).

^{1}H NMR (500 MHz, $CDCl_3$) δ 7.62-7.29 (m, 21H), 7.26-7.20 (m, 3H), 5.35 (t, *J* = 3.0 Hz, 1H), 5.04 (d, *J* = 12.0 Hz, 1H), 4.99-4.90 (m, 2H), 4.86-4.72 (m, 6H), 4.69-4.64 (m, 2H), 4.63-4.58 (m, 2H), 4.54 (d, *J* = 12.8 Hz, 1H), 4.09 (ddd, *J* = 10.2, 3.9, 2.0 Hz, 1H), 4.01 (t, *J* = 9.2 Hz, 1H), 3.79-3.54 (m, 7H), 3.42 (t, *J* = 7.7 Hz, 1H), 3.05-2.96 (m, 1H). **^{13}C NMR (125 MHz, $CDCl_3$) δ** 142.4-141.6 (m), 130.4-129.4 (m), 127.8, 127.7, 127.7, 127.4, 127.3, 127.3, 127.2, 125.6-125.2 (m), 125.1, 122.9, 97.6, 91.0, 84.5, 83.1, 81.7, 80.4, 77.9, 74.6, 74.6, 74.0, 73.7, 72.8, 72.7, 72.3, 70.2, 69.1, 68.8. **^{19}F NMR (471 MHz, $CDCl_3$) δ** -63.11, -63.12, -63.14, -63.16, -63.17, -63.19. **HRMS (ESI) m/z:** [M+Na]+ Calcd. for $C_{38}H_{32}F_{12}O_6Na$ 835.1905; found 835.1947. **$[\alpha]_D^{25}$ =** +32.6° (*c* 1, CH_2Cl_2). **IR (cm^{-1})** 3421, 2926, 1326, 1161, 1121, 1110, 1066, 1018, 822 (*p*-disubstituted benzene), 637.

Synthesis of 11b

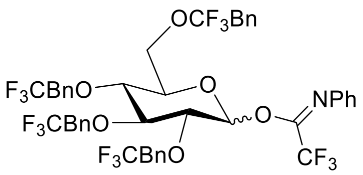

11b

To 5.30 g (6.5 mmol) of **11ba** in an R.B.F equipped with a stir bar, 64.0 ml of acetone was added followed by 6.8 ml of water. 5.50 g of Cs_2CO_3 (17 mmol) was then added. Lastly, 1.8 ml of 2,2,2-

trifluoro-*N*-phenylacetimidoyl chloride[5] was added dropwise. The reaction was allowed to stir at room temperature under nitrogen atmosphere for 24 h. The solvents were then evaporated using a rotary evaporator, then 75.0 ml of dichloromethane was added to dissolve the concentrate. One aqueous wash was then performed using 100.0 ml of water. The organic layer was concentrated then purified by flash chromatography (8-25% EtOAc/hexanes) to give 6.34 g (6.44 mmol) of a slightly yellow, sticky solid **11b** (99%).

^{1}H NMR (500 MHz, $CDCl_3$) δ 7.61-7.06 (m, 20H), 6.77 (d, *J* = 7.8 Hz, 1H), 6.65 (d, *J* = 7.8 Hz, 1H), 4.98 (d, *J* = 12.1 Hz, 1H), 4.90 (d, *J* = 11.9 Hz, 1H), 4.87-4.50 (m, 6H), 4.07-3.94 (m, 1H), 3.83-3.63 (m, 4H); **^{13}C NMR (125 MHz, $CDCl_3$) δ** 143.3, 143.2, 142.3, 142.1, 141.9, 141.7, 141.5, 130.4-129.7 (m), 128.8, 128.8, 127.9, 127.7, 127.7, 127.4, 127.3, 127.2, 126.4, 125.7 - 125.0 (m), 124.5, 124.4, 123.0, 120.4, 119.2, 84.5, 81.5, 81.0, 79.6, 77.2, 77.0, 75.4, 74.8, 74.6, 74.3, 74.0, 73.0, 72.8, 72.6, 72.4, 68.4, **^{19}F NMR (471 MHz, $CDCl_3$) δ -**63.07 (s, 3F), -63.08 (s, 3F), -63.11 (s, 6F), -63.13 (s, 3F), -63.15 (s, 3F), -63.17 (s, 3F), -76.25 (s, 3F) **HRMS (ESI) m/z:** [M+Na]+ Calcd. for $C_{46}H_{36}F_{15}NO_6Na$ 1006.2195; found 1006.2186. **$[\alpha]_D^{25}$ =** +21.7° (*c* 1, CH_2Cl_2). **IR (cm^{-1})** 2870, 1716, 1323, 1214, 1157, 1106, 1064, 1017, 920, 821 (*p*-disubstituted benzene), 694.

Synthesis of 11cb

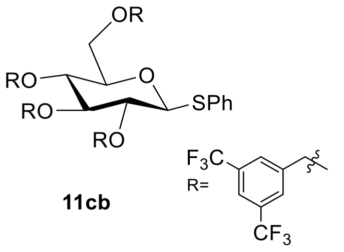

11cb

To a flame-dried round-bottom flask was added a stir bar, 1-thiophenyl-β-D-glucoside **11d** (3.03 g, 11.1 mmol, 1.0 equiv.), and TBAI (0.711 g 2.20 mmol, 0.20 equiv.). Then, anhydrous DMF

(15mL) was added and the mixture was cooled to 0 ^oC in an ice bath. In spatula portions, a 33% NaH suspension in mineral oil was quickly added (3.52 g , 88.1 mmol, 8.0 equiv.). After 10 minutes, 3,5-bis-trifluoromethyl benzyl bromide (27.1 g, 88.1 mmol, 8.0 equiv.) was added dropwise. The ice bath was removed, and the mixture was allowed to warm to room temperature and stir overnight. The next day, the mixture was cooled to 0 ^oC in an ice bath and the septum was removed. Excess NaH was quenched cautiously with the dropwise addition of 10ml of water, and further workup was not commenced until bubbling ceased. The reaction contents were then transferred to a separation funnel. 100ml of water and 50 mL of EtOAc were added and the funnel was shaken vigorously. The EtOAc layer was separated, dried over Na_2SO_4, and concentrated in vacuo to afford a dark red crude oil. The resulting crude oil was purified via flash column chromatography with 1-5% gradient of EtOAc in hexanes to give 2,3,4,6,-tetra-*O*-3,5-*bis*-(trifluoromethyl)benzyl-1-thio-β-D-glucoside **11cb** (10.66 g, 82%) as a red-orange oil. **^{1}H NMR (400 MHz, $CDCl_3$) δ** 7.79 (m, 4H), 7.72 (m *J* = 9.3 Hz, 4H), 7.58 (d, *J* = 10.3 Hz, 4H), 7.53 (m, 2H), 7.33-7.28 (m, 3H), 5.13 (d, *J* = 12.1 Hz, 1H), 4.93-4.64 (m, 8H), 3.86 (m, 2H), 3.76 (m, 2H), 3.62-3.56 (m, 2H). **^{13}CNMR (125 MHz, CDCl3) δ** 140.6, 140.5, 140.3, 140.3, 133.0, 132.2, 132.0, 131.9, 131.9, 131.9, 131.7, 131.6, 129.2, 128.1, 127.3, 127.0, 126.4, 126.1, 124.3, 124.3, 124.2, 122.2, 122.1, 122.0, 122.0, 121.7, 87.9, 86.9, 81.6, 78.9, 78.2, 74.0, 73.7, 73.3, 72.4, 69.5. **^{19}F NMR (471 MHz, $CDCl_3$) δ -**63.39 (s, 6F), -63.54 (s, 6F), -63.64 (s, 6F), -63.74 (s, 6F). **HRMS (ESI) m/z:** [M+Cl]- Calcd. for $C_{48}H_{32}O_5F_{24}SCl$ 1211.1276; found 1211.1284. **$[\alpha]_D^{25}$ =** -314.2° (*c* 0.4, CH_2Cl_2) **IR (cm^{-1})** 2922, 1376, 1355, 1277, 1169, 1126, 884, 843, 801, 747, 705, 682 *(m*-disubstituted aromatic*).*

Synthesis of 11ca

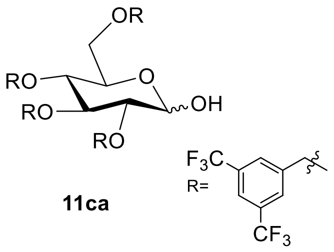

11ca

To a round-bottom flask was added 2,3,4,6,-tetra-*O*-3,5-*bis*-(trifluoromethyl)benzyl-1-thio-β-D-glucoside **11cb** (3.56 g, 3.03 mmol, 1.0 equiv.) followed by 10 mL of an acetone/water mixture (9:1) at room temperature (20 ^oC). In portions, NBS (2.16 g, 12.1 mmol, 4 equiv.) was added and the reaction color changed from dark-red to yellow-red after 10 minutes. After 3 hours stirring at room temperature, TLC indicated full consumption of the starting material. The mixture was concentrated under vacuum, dissolved in DCM (10 mL), and washed with saturated brine (10 mL). The organic layer was collected and dried over Na_2SO_4 and concentrated in vacuo to afford 4.17 g of crude product as an oil.). The resulting crude product was purified via flash column chromatography (in a gradient of 1-25% of Ethyl Acetate in hexanes) to give 2,3,4,6-tetra-*O*-3,5-*bis*(trifluoromethyl)benzyl-D-glucopyranose **11ca** (2.96 g, 90% yield) as a yellow oil. **^{1}H NMR (400 MHz, CDCl$_3$) δ** 7.80 (d, *J* = 6.2 Hz, 6H), 7.74 (s, 4H), 7.71 (m, 3H), 7.62 (s, 2H), 7.60 (m, 5H), 5.43 (t, *J* = 3.2 Hz, 1H), 5.16 (m, 2H), 5.03-4.97 (m, 2H), 4.88 (m, 5H), 4.77-4.63 (m, 7H), 4.16 (m, 1H), 4.09 (t, *J* = 9.3 Hz, 1H), 3.87-3.82 (m, 2H), 3.79 (m, 1H), 3.74 (m, 3H), 3.67 (m, 2H), 3.49 (m, 1H), 3.33 (d, *J* = 4.7, 1H), 2.98 (d, *J* = 3.1 Hz, 1H). **^{13}C NMR (125 MHz, CDCl3) δ**. 140.9, 140.7, 140.6, 140.5, 140.5, 140.4, 140.1, 132.1, 132.0, 131.9, 131.9, 131.8, 131.7, 131.7, 131.6, 131.6, 127.4, 127.1, 126.4, 126.3, 124.2, 122.1, 121.8, 121.6, 121.5, 97.5, 90.7, 84.9, 83.4, 81.9, 81.0, 78.2, 78.1, 74.5, 74.0, 73.9, 73.3, 72.9, 72.3, 72.2, 71.4, 70.2, 69.5, 69.4. **^{19}F NMR (471 MHz, CDCl$_3$) δ -**63.45, -63.49, -63.61, -63.63, -63.72, -63.74. **HRMS (ESI) m/z:** [M+Cl]- Calcd. for $C_{42}H_{28}O_6F_{24}$ 1119.1191; found 1119.1197. **$[\alpha]_D^{25}$ =** -11.9 (*c* 1.3, CH_2Cl_2) **IR (cm^{-1})** 2925, 1377, 1356, 1276, 1168, 1123, 908, 884, 843, 800, 733, 705, 682 (*m*-disubstituted aromatic).

Synthesis of 11c

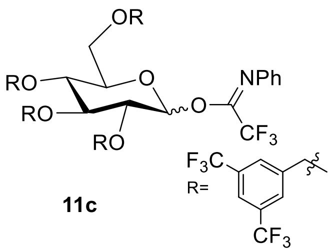

11c

To a round-bottom flask was added a stir bar, 2,3,4,6-tetra-*O*-3,5-*bis*(trifluoromethyl)benzyl-D-glucopyranose **11ca** (6.64 g, 6.12 mmol, 1.0 equiv.), and 10:1 mixture of acetone:deionized water (110 mL). Then, Cs_2CO_3 (1.91 g, 9.18 mmol, 1.5 equiv.) and 2,2,2-trifluoro-*N*-phenylacetimidoyl chloride, (2.99 g, 9.18 mmol, 1.5 equiv.) were added respectively at room temperature. The reaction mixture was allowed to stir overnight at room temperature, after which it was quenched with 1 mL of triethylamine. The contents were filtered and concentrated under vacuum to afford crude product as an oil. Purification on flash column chromatography in a solvent gradient of 1-10% ethyl acetate in hexanes gave 2,3,4,6-tetra-*O*-3,5-*bis*(trifluoromethyl)benzyl-D-glucopyranose *N*-phenyl trifluoroacetimidate **11c** as a viscous, orange oil (7.05 g, 5.62 mmol, 92 %).

^{1}H NMR (500 MHz, $CDCl_3$) δ 7.81-7.55 (m, 13H), 7.40 (t, *J* = 7.85 Hz, 1H), 7.41-7.23 (m,3H), 7.14-7.08 (m, 1H), 6.78 (d, *J* = 7.8 Hz, 1H), 6.66 (d, *J* = 7.7 Hz, 1H), 5.05-4.65 (m, 9H), 4.13 (m, 1H), 3.92-3.77 (m, 5H).**^{13}C NMR (125 MHz, CDCl3) δ** 143.1, 143.0, 140.7, 140.5, 140.5, 140.5, 140.3, 140.3, 140.2, 139.9, 132.2, 132.1, 132.1, 132.1, 132.0, 132.0, 132.0, 131.9, 131.8, 131.8, 131.8, 131.8, 131.7, 129.5, 128.9, 127.4, 127.0, 126.8, 126.5, 126.5, 126.4, 126.2, 124.8, 124.2, 124.2, 122.1, 122.0, 121.8, 120.5, 119.2, 84.8, 81.6, 79.9, 77.7, 75.5, 74.1, 74.0, 73.67, 73.4, 73.0, 72.3, 72.2, 71.5, 68.9, 68.8. **^{19}F NMR (471 MHz, $CDCl_3$) δ** -63.49, -63.60, -63.63, -63.68, -63.74. **HRMS (ESI) m/z:** [M]+ Calcd. for $C_{50}H_{32}NO_6F_{27}$ 1255.1798; found 1255.1989. **$[\alpha]_D^{25}$ =** -

78.1° (c 1.25, CH_2Cl_2) **IR (cm^{-1})** 2924, 1718, 1738, 1356, 1277, 1168, 1127, 884, 843, 705, 682 (*m*-disubstituted aromatic).

Synthesis of trichloroacetimidate glucosyl donor with *p*-trifluoromethylbenzyl protecting groups (10b) Synthesis plan:

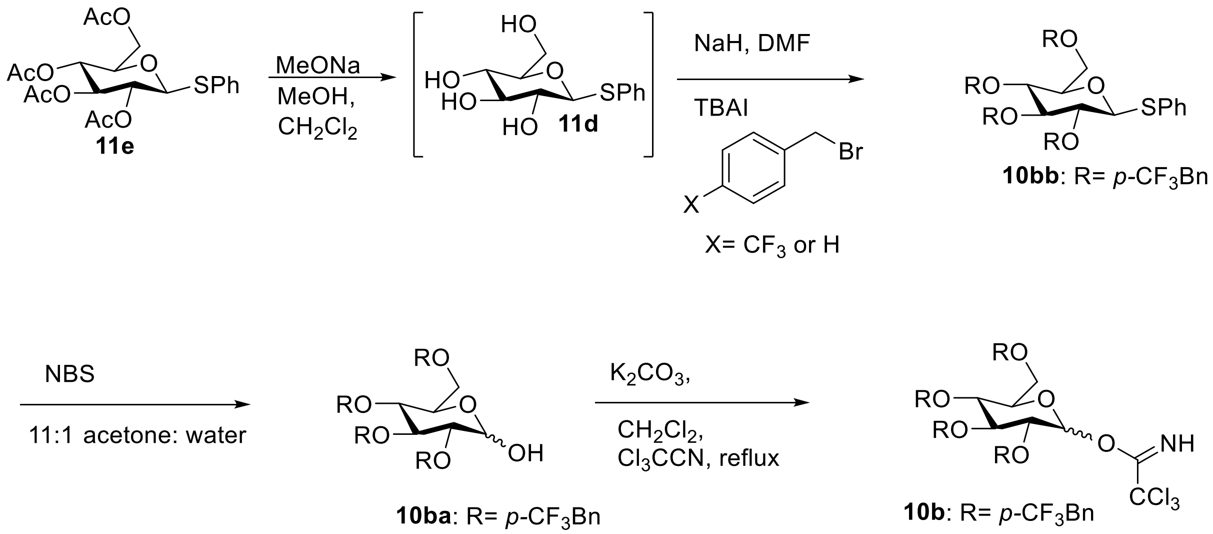

Synthesis of 10b

To 1.11 g (1.37 mmol) of **10ba** (a known compound which was prepared according to detailed procedures in reference 6) in an R.B.F with a stir bar, 25.0 ml of dichloromethane was added followed by of 4.5 g of K_2CO_3 (excess). Lastly, trichloroacetonitrile (5.0 ml, excess) was added. The reaction was allowed to stir at room temperature, however, after 5hrs, little product formation was observed as shown by T.L.C. A reflux condenser was then attached, and the reaction mixture was refluxed for two hours. Product was cleanly formed as judged by T.L.C and ^1H NMR. The reaction contents were filtered through celite, then the filtrate was concentrated and then put under high vacuum to give 1.20 g of a white powder **10b** (91%) that required no further purification.

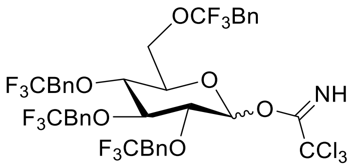

10b
mostly *beta* anomer

Data for *beta* anomer: ^{1}H NMR (500 MHz, $CDCl_3$) δ 8.75 (s, 1H), 7.56-7.24 (m, 16H), 5.82 (dd, *J* = 5.4, 2.2 Hz, 1H), 4.99 (d, *J* = 11.8 Hz, 1H), 4.88 (d, *J* = 12.2 Hz, 1H), 4.84-4.71 (m, 3H), 4.69-4.61 (m, 2H), 4.58 (d, *J* = 12.8 Hz, 1H), 3.83-3.63 (m, 6H). **^{13}C NMR (125 MHz, $CDCl_3$) δ** 161.1, 142.1, 141.8, 141.7, 130.3-129.6 (m), 127.8, 127.7, 127.4, 127.2, 125.4, 125.3, 125.3, 98.1, 90.8, 84.5, 81.0, 77.34, 75.6, 74.6, 74.0, 73.9, 72.6, 68.4. **^{19}F NMR (471 MHz, $CDCl_3$) δ** -63.08 (s, 3F), -63.15 (s, 6F), -63.18 (s, 3F). **HRMS (ESI) m/z:** [M+Na]+ Calcd. for $C_{40}H_{32}Cl_{3,}F_{12}NO_6Na$ 978.1001; found 978.1024. **$[\alpha]_D^{25}$ =** -15.5° (*c* 1, CH_2Cl_2). **IR (cm^{-1})** 2923, 1325, 1263, 1121, 1066, 1018, 822 (*p*-disubstituted benzene), 797, 736, 703.

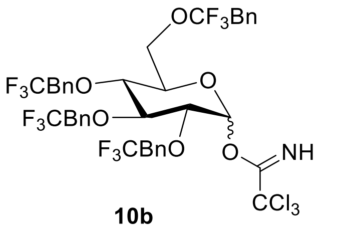

10b
mostly *alpha* anomer

Data for *alpha* anomer: ^{1}H NMR (500 MHz, $CDCl_3$) δ 8.63 (s, 1H), 7.64-7.22 (m, 16H), 6.60 (d, *J* = 3.4 Hz, 1H), 4.98 (d, *J* = 12.0 Hz, 1H), 4.92-4.74 (m, 3H), 4.74-4.59 (m, 3H), 4.54 (d, *J* = 12.7 Hz, 1H), 4.12-4.00 (m, 2H), 3.87-3.66 (m, 4H). **^{13}C NMR (125 MHz, $CDCl_3$) δ** 161.2, 142.3, 141.8, 141.7, 130.5-129.4 (m), 127.7, 127.5, 127.5, 127.4, 125.4-125.3 (m), 125.1, 123.0, 93.8, 91.1, 81.5, 79.7, 77.2, 77.0, 74.7, 74.3, 73.0, 72.7, 72.1, 68.4. **^{19}F NMR (471 MHz, $CDCl_3$) δ** -

63.11(s, 3F), -63.13 (s, 6F), -63.19 (s, 3F). **HRMS (ESI) m/z:** [M+Na]+ Calcd. for $C_{40}H_{32}Cl_{3,}F_{12}NO_6Na$ 978.1001; found 978.1003. **$[\alpha]_D^{25}$ =** +43.5° (*c* 1, CH_2Cl_2). **IR (cm^{-1})** 2921, 1738, 1672, 1325, 1163, 1121, 1066, 1018, 822 (*p*-disubstituted benzene), 795, 737, 643.

GLYCOSYLATION USING *N*-PHENYLTRIFLUOROACETIMIDATE AND TRICHLOROACETIMIDATE GLUCOSYL DONORS

General procedure A

0.15 mmol of 2,3,4,6-tetra-*O*-(4-trifluoromethylbenzyl)-D-glucopyranose glucosyl donor (**10b or 11b**) or 2,3,4,6-tetra-*O*-benzyl-D-glucopyranose glucosyl donor (**10a or 11a**) **or** 2,3,4,6-tetra-*O*-3,5-*bis*-(trifluoromethyl)benzyl-D-glucopyranose glucosyl donor (**11c**) (1 equiv.) was placed in an oven dried 4ml Wheaton vial with a stir bar followed by addition of 0.105 mmol of acceptor (0.7 equiv.). Triphenylphosphine oxide (0.9 mmol, 6 equiv.) was then added to vial. The vial was then capped with a septum and flushed with nitrogen gas for 1 min carefully to avoid agitation of the solids. 1.5 ml of dry dichloromethane was then added with nitrogen line still attached. Finally, 22.5 µL (0.158 mmol, 1.05 equiv) of TMSI was added using a micro-syringe and the reaction was allowed to stir at 18 °C for 20 hrs, for donor 3af the reaction was allowed to stir for 72 hours. The reaction mixture was then transferred to a separatory funnel and diluted with 5.0 ml of dichloromethane then vigorously washed once with 10.0 ml of 1M NaOH. The organic layer was dried using Na_2SO_4 then concentrated using rotary evaporator followed by purification using flash column chromatography. Note: bad (brown colored) or old TMSI (open for more than a month) was detrimental to selectivity and yield of glycosylation reactions.

General procedure for preparing mostly beta glycosides:

We prepared *beta* glycosides by addition of trimethyl phosphate (excess) as an additive. Below is a representative procedure on how that was achieved using *N*-benzyl benzyloxycarbonylaminopentanol acceptor **17**:

To a stirred solution of donor **11b** (145.0 mg) and *N*-benzyl benzyloxycarbonylaminopentanol (55.6 mg, acceptor) in dichloromethane (2.0 ml) in a vial, 0.3 ml of trimethyl phosphate was added. The vial was capped then flushed with nitrogen gas before addition of TfOH (15 µL). After 24 hrs, the reaction was stopped, diluted with 5ml DCM then washed with 10ml 1M NaOH. The Organic phase was dried using Na_2SO_4, and concentrated to give crude material that was purified by flash chromatography 20-28% EtOAc/hexanes to give 67.3 mg of a mixture of α/β glycoside (55%). The product was further subjected to preparative thin layer chromatography (14:1:5 CH_2Cl_2 :EtOAc:hexane) to give 10.0 mg of alpha glucoside and 34.1mg of beta glucoside.

Table 1[a]: Optimization using Primary acceptor 12

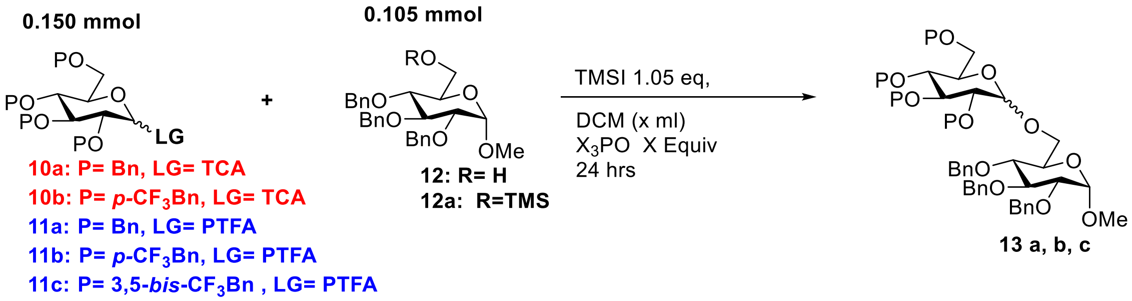

Entry	Donor	Acceptor	Additive (eq)	Solvent (ml)	crude (α/β)	purified (α/β)	yield%
1	10a	12	Ph_3PO (6eq)	CH_2Cl_2 (1.5 ml)	13:1	14:1	>99%
2	10b	12	Ph_3PO (6eq)	CH_2Cl_2 (1.5 ml)	34:1	34:1	84%
3[d]	10b	12	Ph_3PO (6eq)	CH_2Cl_2 (1.5 ml)	34:1	29:1	79%
4	10a	12	Ph_3PO (6eq)	CH_2Cl_2 (5.0 ml)	17:1	12:1	31%
5	10b	12	Ph_3PO (6eq)	CH_2Cl_2 (5.0 ml)	36:1	26:1	53%
6[b]	10a	12	Ph_3PO (15eq)	CH_2Cl_2 (1.5 ml)	13:1	-	crude
7[b]	10b	12	Ph_3PO (15eq)	CH_2Cl_2 (1.5 ml)	35:1	-	crude
8[b,c]	10b	12	Ph_3PO (6eq)	CH_2Cl_2 (1.5 ml)	28:1	-	crude
9[b]	10b	12	Me_3PO (6eq)	CH_2Cl_2 (1.5 ml)	25:1	-	crude
10[b]	10b	12	$MePh_2PO$ (6eq)	CH_2Cl_2 (1.5 ml)	28:1	-	crude
11[b]	10b	12	$CyPh_2PO$ (6eq)	CH_2Cl_2 (1.5 ml)	36:1	-	crude
12	11a	12	Ph_3PO (6eq)	CH_2Cl_2 (2.0 ml)	21:1	24:1	81%
13	11b	12	Ph_3PO (6eq)	CH_2Cl_2 (1.5 ml)	43:1	44:1	>99%
14	11b	12	Ph_3PO (6eq)	CH_2Cl_2 (2.0 ml)	40:1	40:1	93%
15	10b	12	-	CH_2Cl_2 (1.5 ml)	8.8: 1	5.6: 1	81%
16[e]	11a	12a	Ph_3PO (6eq)	CH_2Cl_2 (2.0 ml)	25:1	20:1	>99%
17[e]	11b	12a	Ph_3PO (6eq)	CH_2Cl_2 (2.0 ml)	28:1	32:1	>99%
18[f]	11c	12	Ph_3PO (6eq)	CH_2Cl_2 (2.0 ml)	43: 1	42:1	>99%

[a]Reaction proceeded at 18 °C, [b]ratio obtained from crude product mixture only,low acceptor consumption as judged by crude NMR, [c]2 equivalents of TMSI was used, [d]mostly alpha TCA was used. [e]TMS-ether acceptor 3e was used and reaction was ran 48hrs, [f]reaction was allowed run for 72hrs.

Various acceptors used in the substrate scope study:

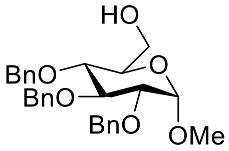

12[3]

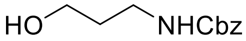

16[7]

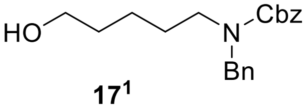

17[1]

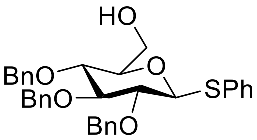

20[4]

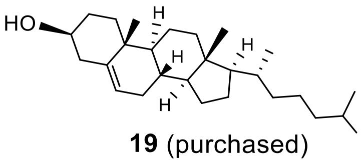

19 (purchased)

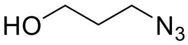

18[8]

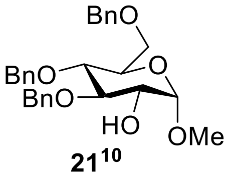

21[10]

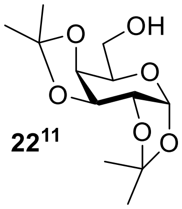

22[11]

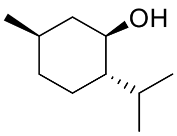

23 (purchased)

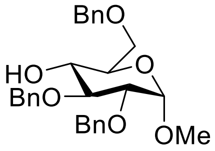

14[9]

Glycosylation using glycosyl acceptor 12 with donor 10a to give:

(13a) Methyl (2,3,4,6-tetra-*O*-benzyl-D-glucopyranosyl)-(1→6)-2,3,4-tri-O-benzyl-D-glucopyranoside

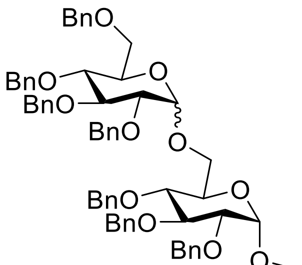

13a

Following the general procedure **A**, 1.5 ml of dichloromethane was used, 104.4 mg (0.1524 mmol) of 2,3,4,6-tetra-O-benzyl-D-glucopyranose trichloroacetimidate (**10a**), 48.9 mg (0.105 mmol) of acceptor **12,** 251.0 mg of triphenylphosphine oxide (0.9020 mmol,5.92 equiv.) and 25.0 µL (0.176 mmol) of TMSI (1.15 equiv.) were used. Purified by flash column chromatography (12-28% EtOAc/hexanes) to give 103.5 mg of a colorless oil **13a** (>99%) *alpha*:*beta* ratio 13:1. ^{1}H NMR spectra of both anomeric products matched the previously published spectra.[3]

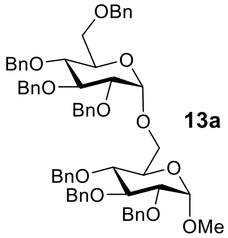

13a *alpha*

^{1}H NMR data for 13a alpha: (300 MHz, $CDCl_3$) δ 7.37 – 7.09 (m, 35H), 4.98 – 4.92 (m, 4H), 4.84 – 4.55 (m, 10H), 4.43 (t, *J* = 11.9 Hz, 1H), 4.00 (d, *J* = 8.7 Hz, 1H), 3.94 (d, *J* = 9.2 Hz, 1H), 3.85 – 3.62 (m, 7H), 3.59 – 3.52 (m, 2H), 3.44 (dd, *J* = 9.6, 3.8 Hz, 1H), 3.35 (s, 3H).

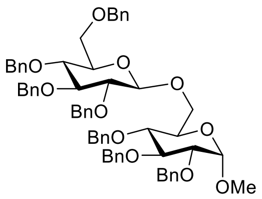

13a *beta*

^{1}H NMR data for 13a beta: (300 MHz, $CDCl_3$) δ 7.37 – 7.14 (m, 35H), 4.97 (dd, *J* = 11.3, 2.9 Hz, 1H), 4.90 (d, *J* = 11.0 Hz, 1H), 4.80 (d, *J* = 11.0 Hz, 1H), 4.78 (d, *J* = 11.0 Hz, 2H), 4.73 (d, *J* = 3.2 Hz, 1H), 4.68 (d, *J* = 9.2 Hz, 1H), 4.62 – 4.49 (m, 3H), 4.56 (d, *J* = 7.5 Hz, 2H), 4.35 (d, *J* = 7.7 Hz, 1H), 4.18 (dd, *J* = 10.7, 1.9 Hz, 1H), 3.99 (t, *J* = 9.2 Hz, 1H), 3.85 – 3.80 (m, 1H), 3.74 – 3.38 (m, 9H), 3.32 (s, 3H).

Glycosylation using glycosyl acceptor 12 with donor 10b to give:

Methyl (2,3,4,6-tetra-*O*-(4-trifluoromethylbenzyl)-D-glucopyranosyl)-(1→6)-2,3,4-tri-*O*-benzyl-D-glucopyranoside (13b)[6]

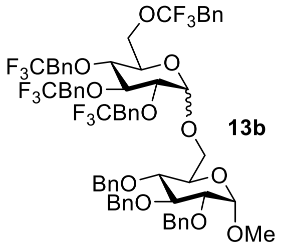

13b

Following the general procedure **A**, 1.5 ml of dichloromethane was used, 146.4 mg (0.1530 mmol) of 2,3,4,6-tetra-*O*-(4-trifluoromethylbenzyl)-D-glucopyranose trichloroacetimidate donor (**10b**), 49.3 mg (0.106 mmol) of acceptor **12,** 253.2 mg of triphenylphosphine oxide (0.9099 mmol, 5.95 equiv.) and 25.0 µL (0.176 mmol) of TMSI (1.15 equiv.) were used. Purified by flash column chromatography (12-28% EtOAc/hexanes) to give 112.2 mg of a colorless oil **13b**, yield 84%, *alpha*:*beta* ratio 34:1. ^{1}H NMR spectra of both anomeric products matched the previously published spectra.[6]

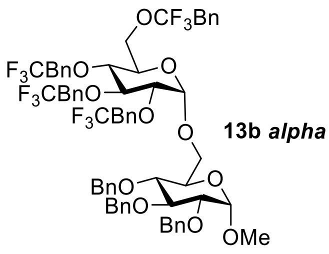

13b *alpha*

Data for 13b *alpha*: **^{1}H NMR** (400 MHz, $CDCl_3$) δ 7.85-7.25 (m, 29H), 7.17 (d, *J* = 8.0 Hz, 2H), 5.05 (d, *J* = 3.5 Hz, 1H) 4.98-4.89 (m, 3H), 4.81-4.65 (m, 6H), 4.62-4.43 (m, 6H), 4.00 (t, *J* = 9.1 Hz, 1H), 3.95-3.86 (m, 2H), 3.80-3.76 (m, 2H), 3.72-3.67 (m, 2H), 3.66-3.50 (m, 4H), 3.39 (dd, *J* = 9.6 Hz, 3.6 Hz, 1H), 3.36 (s, 3H); **^{13}C NMR** (125 MHz, $CDCl_3$) δ 142.8, 142.4, 142.3, 142.1, 138.8, 138.6, 138.2, 132.2, 130.3, 130.2, 130.1, 130.0, 129.9, 129.8, 128.7, 128.6, 128.6, 128.3, 128.1, 128.0, 127.9, 127.9, 127.8, 127.7, 127.6, 127.5, 127.3, 125.6, 125.6, 125.5, 125.5, 125.5, 125.5, 125.5, 125.4, 125.4, 125.3, 125.3, 123.2, 114.5, 98.3, 97.1, 82.3, 81.9, 80.5, 80.3, 77.9, 77.8, 76.1, 75.1, 74.7, 74.1, 73.6, 72.9, 71.7, 70.5, 70.3, 68.9, 66.3, 55.4; **^{19}F NMR** (471 MHz, $CDCl_3$) δ -63.08(s, 3F), -63.21 (s, 3F), -63.22 (s, 3F), -63.26 (s, 3F). **HRMS (ESI) m/z:** [M+K]+ Calcd. for $C_{66}H_{62,}F_{12}O_{11}K$ 1297.3738; found 1297.3758. **$[\alpha]_D^{25}$ =** +11.5° (*c* 1, CH_2Cl_2). **IR (cm^{-1})** 3055, 2926, 1325, 1264, 1163, 1123, 1065, 1017, 822 (*p*-disubstituted benzene), 734, 702.

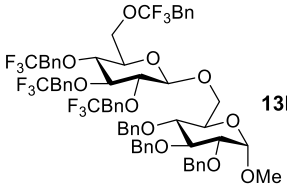

13b *beta*

Data for 13b *beta*: **1H NMR** (400 MHz, $CDCl_3$) δ 7.60-7.14 (m, 31H), 5.02 (d, *J* = 12.0 Hz, 1H), 4.96 (d, *J* = 10.8 Hz, 1H), 4.91-4.56 (m, 12H), 4.51 (dd, *J* = 12.3 Hz, 6.4 Hz, 2H), 4.36 (d, *J* = 7.7 Hz, 1H), 4.16 (dd, *J* = 10.8 Hz, 2.0 Hz, 1H), 4.00 (t, *J* = 9.2 Hz, 1H), 3.86-3.81 (m, 1H), 3.73-3.66 (m, 3H), 3.62-3.55 (m, 2H), 3.51-3.38 (m, 4H), 3.32 (s, 3H); **^{13}C NMR** (125 MHz, $CDCl_3$) δ 142.5, 142.3, 142.3, 142.1, 138.8, 138.4, 138.2, 130.4-129.8 (m), 128.7, 128.6, 128.6, 128.3, 128.2, 128.2, 127.9, 127.9, 127.8, 127.7, 127.7, 127.6, 127.5, 127.5, 127.5, 125.7-125.4 (m), 123.2,

103.9, 98.3, 84.9, 82.2, 82.1, 80.0, 78.2, 78.2, 76.1, 75.1, 75.0, 74.8, 74.2, 74.0, 73.6, 72.8, 70.0, 69.2, 69.0, 55.4. **^{19}F NMR** (471 MHz, $CDCl_3$) δ -63.11 (s, 3F), -63.15 (s, 3F), -63.26 (s, 3F), -63.28 (s, 3F). **HRMS (ESI) m/z:** [M+K]+ Calcd. for $C_{66}H_{62,}F_{12}O_{11}K$ 1297.3738; found 1297.3772. **$[\alpha]_D^{25}$ =** -0.6° (*c* 1, CH_2Cl_2). **IR (cm^{-1})** 3063, 2917, 1620, 1324, 1264, 1162, 1121, 1065, 1017, 912, 821 (*p*-disubstituted benzene), 736, 670.

Glycosylation using glycosyl acceptor 12 with donor 11a

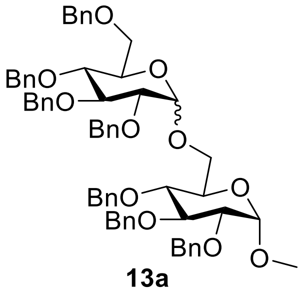

13a

Following the general procedure **A**, 2.0 ml of dichloromethane was used, 107.4 mg (0.1509 mmol) of 2,3,4,6-tetra-*O*-benzyl-D-glucopyranose *N*-phenyl trifluoroacetimidate (**11a**), 49.0 mg (0.106 mmol) of acceptor **12,** 250.9 mg of triphenylphosphine oxide (0.9016 mmol 5.97 equiv.) and 22.5 µL (0.158 mmol) of TMSI (1.05 equiv.) were used. Purified by flash column chromatography (12-28% EtOAc/hexanes) to give 84.9 mg of a colorless oil **13a**, (81%, *alpha*:*beta* ratio 24:1). ^{1}H NMR spectra of both anomeric products matched the previously published spectra.[6]

Glycosylation using glycosyl acceptor 12 with donor 11b

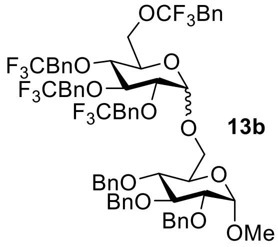

13b

Following the general procedure **A**, 2.0 ml of dichloromethane was used, 148.2 mg (0.1506 mmol) of 2,3,4,6-tetra-*O*-(4-trifluoromethylbenzyl)-D-glucopyranose *N*-phenyl trifluoroacetimidate donor (**11b**), 49.3 mg (0.106 mmol) of acceptor **12,** 251.0 mg of triphenylphosphine oxide 0.9020 mmol (5.98 equiv.) and 22.5 µL (0.158 mmol) of TMSI (1.05 equiv.) were used. Purified by flash column chromatography (12-28% EtOAc/hexanes) to give 124.5 mg of a colorless oil **13b**, (93%, *alpha*:*beta* ratio 40:1). ^{1}H NMR spectra of both anomeric products matched the previously published spectra.[6]

Glycosylation using glycosyl acceptor 12 with donor 11c to give:

(13c) Methyl (2,3,4,6-tetra-*O*-3,5-*bis*-(trifluoromethyl)benzyl-D-glucopyranosyl)-(1→6)-2,3,4-tri-O-benzyl-D-glucopyranoside

Following the general procedure **A**, 2.0 ml of dichloromethane was used, 188.4 mg (0.1511 mmol) of 2,3,4,6-tetra-O-3,5-*bis*(trifluoromethyl)benzyl-D-glucopyranose *N*-phenyl trifluoroacetimidate (**11c**) , 49.1 mg (0.106 mmol) of acceptor **12,** 250.7 mg of triphenylphosphine oxide (0.9009 mmol, 5.96 equiv.) and 22.5 µL (0.158 mmol, 1.05 equiv.) of TMSI were used. The reaction was stirred at room temperature for 72 hours. Purified by flash column chromatography (2-10% EtOAc/hexanes) to give 166.9 mg of a white solid **13c**, >99% *alpha*:*beta* ratio 42:1.

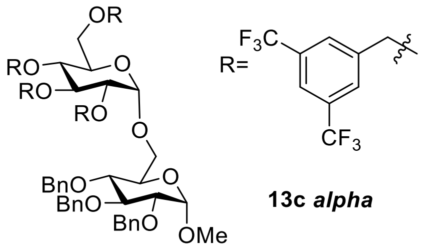

13c *alpha*

Data for *alpha* anomer 13c: **^{1}H NMR (500 MHz, CDCl3) δ** 7.79-7.68 (m, 10H), 7.60 (s, 2H), 7.56 (m, 2H), 7.31-7.21 (m, 13H), 5.07 (d, J = 3.5 Hz, 1H), 4.98 (t, J = 10.7 Hz, 3H), 4.83-4.71 (m, 6H), 4.67-4.53 (m, 7H), 3.99 (m, 2H), 3.93 (dd, J = 7.1 Hz, J = 4.4 Hz, 1H), 3.83 (m, 2H), 3.69-3.56 (m, 4H), 3.52 (d, J = 10.3 Hz, 1H), 3.44 (dd, J = 6.0 Hz, J = 3.7 Hz,1H), 3.38 (s, 3H). **^{13}C NMR (125 MHz, CDCl3) δ** 141.0, 140.6, 140.6, 140.5, 138.5, 138.4, 138.0, 131.9, 131.9, 131.8, 131.6, 131.6, 131.5, 128.5, 128.4, 128.4, 128.1, 127.9, 127.9, 127.8, 127.6, 127.3, 126.9, 126.3, 124.3, 124.2, 122.0, 121.7, 98.0, 96.6, 82.3, 82.1, 80.2, 80.2, 77.9, 77.6, 75.9, 74.8, 73.8, 73.3, 73.2, 72.0, 70.2, 69.9, 69.9, 69.0, 66.7, 55.1. **^{19}F NMR (471 MHz, CDCl3) δ** -63.48 (s, 6F), -63.49 (s, 6F), 63.61 (s, 6F), -63.72 (s, 6F). **HRMS (ESI) m/z:** [M+Na]+ Calcd. for $C_{70}H_{58}F_{24}O_{11}$ 1553.3494; found 1533.3457. **$[\alpha]_D^{25}$ =** -352.4° (*c* 0.38) **IR (cm^{-1})** 2958, 2925, 2854, 1728, 1279, 1260, 1175, 1073, 1019, 799, 739, 682 (*m*-disubstituted aromatic).

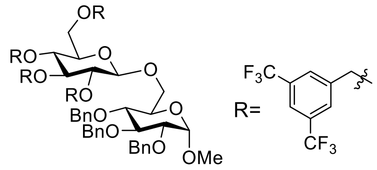

13c *beta*

Data for *beta* anomer 13c: **^{1}H NMR (500 MHz, CDCl3) δ** 7.79-7.64 (m, 8H), 7.57 (s, 2H), 7.53 (s, 2H), 7.34-7.23 (m, 13H), 7.20 (m, 2H), 5.15 (d, J = 12.5 Hz, 1H), 4.96 (d, J = 10.9 Hz, 1H), 4.93 (d, J = 12.9 Hz, 1H), 4.83-4.75 (m, 6H), 4.73-4.59 (m, 5H), 4.52-4.49 (m, 2H), 4.45 (d, J = 7.7 Hz, 1H), 4.17 (d, J = 9.6 Hz, 1H), 3.98 (t, J = 9.3 Hz, 1H), 3.88 (m, 2H), 3.77 (d, J = 11.2 Hz, 1H), 3.71-3.63 (m, 3H), 3.51-3.45 (m, 2H), 3.23 (t, J = 9.45 Hz, 1H), 3.28 (s, 3H). **^{13}C NMR (125 MHz, CDCl3) δ** 140.7, 140.7, 140.6, 140.5, 138.5, 138.1, 138.0, 131.9, 131.9, 131.8, 131.7, 131.5, 131.5, 128.8, 128.5, 128.4, 128.4, 128.1, 128.0, 128.0, 127.8, 127.7, 127.6, 127.3, 126.9, 126.4, 126.1, 124.2,121.5, 103.5, 98.0, 85.0, 82.3, 81.8, 79.9, 78.4, 78.1, 75.8, 74.9, 74.6, 73.9,

73.4, 73.3, 72.8, 72.3, 70.0, 69.4, 69.1, 55.1. **^{19}F NMR (471 MHz, CDCl3) δ** -63.42 (s, 6H), -63.54 (s, 6H), -63.65 (s, 6H), -63.78 (s, 6H) **HRMS (ESI) m/z:** [M+Na]+ Calculated for $C_{70}H_{58}O_{11}F_{24}$ 1553.3494; found 1533.3476. **$[\alpha]_D^{25}$ =** -166.8° (*c* 0.78) **IR (cm^{-1})** 2959, 2918, 2067, 1725, 1377, 1356, 1277, 1171, 1129, 883, 842, 800, 735, 704, 682 (*m*-disubstituted aromatic).

Glycosylation using acceptor 14 and donor 10a

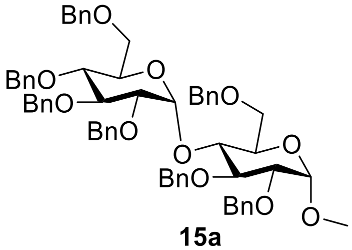

15a

Following the general procedure **A** with slight modification, 1.5 ml of dichloromethane was added to 105.6 mg (0.1541 mmol) of 2,3,4,6-tetra-O-benzyl-D-glucopyranose trichloroacetimidate (**10a**), 49.7 mg (0.107 mmol) of acceptor **14** and 254.5 mg of triphenylphosphine oxide (0.9145 mmol, 5.93 equiv.) in a 4ml Wheaton vial with a stir bar. Immediately after, 225.1 mg of activated 3A molecular sieves was added and the vial was capped with a septum. N_2 gas was flushed through the vial for 2 minutes then the mixture was allowed to stir for 40 minutes before 25.0 µL (0.176 mmol) of TMSI (1.15 equiv.) was added dropwise. Reaction was allowed to run for 24 hrs. Purified by flash column chromatography (44-56% DE/hexanes (where DE= 5:1 DCM/EtOAc)) to give 16.9 mg of a colorless oil **15a**, yield 16%, *alpha* only. ^{1}H NMR spectra matched the previously published spectra.[12]

*Detailed tabulated characterization data for **15a** (both anomers) can be found in reference 12.*

Glycosylation using acceptor 14 and donor 10b

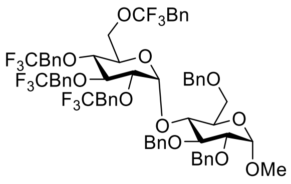

15b

Following the general procedure **A** with slight modification, 1.5 ml of dichloromethane was added to 144.1 mg (0.1506 mmol) of 2,3,4,6-tetra-*O*-(4-trifluoromethylbenzyl)-D-glucopyranose trichloroacetimidate donor (**10b**), 49.5 mg (0.107 mmol) of acceptor **14** and 255.0 mg of triphenylphosphine oxide (0.9163 mmol, 6.08 equiv.) in a 4 ml Wheaton vial with a stir bar. Immediately after, 220.0 mg of activated 3A molecular sieves was added and the vial was capped with a septum. N_2 gas was flushed through the vial for 2 minutes then the mixture was allowed to stir for 40 minutes before 25.0 µL (0.176 mmol) of TMSI (1.15 equiv.) was added dropwise. Reaction was allowed to run for 24 hrs. Purified by flash column chromatography (52-56% DE/hexanes (where DE= 3:1 DCM/EtOAc)) to give 44.7 mg of a colorless oil **15b**, yield 33%, *alpha* only. ^{1}H NMR spectra matched the previously published spectra.[6]

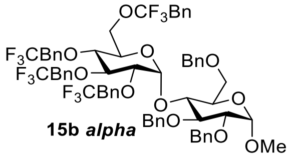

15b *alpha*

Data for *alpha* anomer 15b: ^{1}H NMR (400 MHz, $CDCl_3$) δ 7.51-7.13 (m, 31H), 5.78 (d, *J* = 3.6 Hz, 1H), 5.10 (d, *J* = 11.8 Hz, 1H), 4.87 (d, *J* = 12.0 Hz, 1H), 4.76-4.70 (m, 4H), 4.65 (d, *J* = 3.7 Hz, 1H), 4.60-4.46 (m, 7H), 4.30 (d, *J* =12.8 Hz, 1H), 4.10-4.08 (m, 2H), 3.92-3.86 (m, 3H), 3.74-3.77 (m, 1H), 3.70-3.61 (m, 3H), 3.46-3.42 (m, 2H), 3.40 (s, 3H), 3.39-3.36 (m, 1H); **^{13}C NMR (125 MHz, $CDCl_3$)** δ 142.5, 142.2, 141.9, 141.8, 138.9, 138.2, 137.8, 130.3-129.3 (m), 128.5, 128.3, 128.3, 128.2, 128.0, 127.8, 127.5, 127.4, 127.3, 127.2, 127.2, 127.1, 126.4, 125.3-125.1 (m), 123.0, 123.0, 97.7, 96.3, 82.1, 82.0, 80.2, 79.7, 77.8, 74.4, 74.3, 74.0, 73.4, 73.3, 72.7, 72.4, 72.1,

70.8, 69.6, 69.1, 68.5, 55.3**;** **^{19}F NMR** (471 MHz, $CDCl_3$) δ -63.08 (s, 3F), -63.09 (s, 3F), -63.12 (s, 3F), -63.15 (s, 3F). **HRMS (ESI) m/z:** [M+Na]+ Calcd. for $C_{66}H_{62,}F_{12}O_{11}Na$ 1281.3998; found 1281.3996. **$[\alpha]_D^{25}$ =** +39.8° (*c* 1, CH_2Cl_2). **IR (cm^{-1})** 3059, 2914, 2867, 1620, 1324, 1266, 1161, 1121, 1065, 1017, 850, 822 (*p*-disubstituted benzene),735, 699, 593.

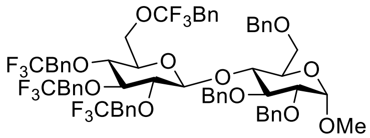

15b *beta*

Data for *beta* anomer 15b: ^{1}H NMR (400 MHz, $CDCl_3$) δ 7.53-7.17 (m, 31H), 5.05 (d, *J* = 11.4 Hz, 1H), 4.87-4.58 (m, 12H), 4.45-4.35 (m, 4H), 3.98 (t, *J* = 9.5 Hz, 1H), 3.85 (t, *J* = 9.3 Hz, 1H), 3.79 (dd, *J* = 10.8 Hz, 2.9 Hz, 1H), 3.70 (dd, *J* = 11.2 Hz, 1.9 Hz, 1H), 3.60-3.38 (m, 6H), 3.36 (s, 3H), 3.31-3.27 (m, 1H); **^{13}C NMR (125 MHz, $CDCl_3$)** δ 142.4, 142.4, 142.3, 142.1, 139.4, 138.2, 137.8, 130.0-129.4 (m), 128.5, 128.4, 128.1, 128.0, 128.0, 127.9, 127.9, 127.9, 127.4, 127.3, 127.2, 127.2, 127.2, 127.1, 125.3-125.1 (m), 123.0, 102.2, 98.4, 84.8, 82.8, 80.3, 79.0, 78.3, 76.3, 75.4, 75.0, 74.5, 73.8, 73.8, 73.6, 73.6, 72.5, 70.0, 69.0, 67.9, 55.5; **^{19}F NMR** (471 MHz, $CDCl_3$) δ -62.98 (s, 3F), -63.06 (s, 3F), -63.14 (s, 3F), -63.15 (s, 3F) **HRMS (ESI) m/z:** [M+Na]+ Calcd. for $C_{66}H_{62,}F_{12}O_{11}Na$ 1281.3998; found 1281.3990. **$[\alpha]_D^{25}$ =** +25.6° (*c* 1, CH_2Cl_2). **IR (cm^{-1})** 3056, 2916, 1324, 1265, 1163, 1122, 1065, 1018, 822 (*p*-disubstituted benzene), 736, 701.

Glycosylation between acceptor 14 and donor 10a using DMF as additive.

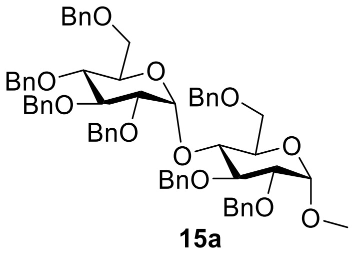

15a

To 2,3,4,6-tetra-O-benzyl-D-glucopyranose trichloroacetimidate (105.4 mg, 0.1538 mmol) (**10a**), in a 4 mL Wheaton vial with a stir bar, 1.0 mL of dichloromethane (DCM) and 200 µL of dimethylformamide (DMF) were added. The vial was capped and flushed with N_2 gas for 2 minutes then allowed to stir at -70 °C. 15.0 µL of TfOH was then added and solution allowed to stir. After 40 minutes, acceptor **14** (49.3 mg, 0.106 mmols) dissolved in 1 ml of DCM was added dropwise under N_2 atmosphere. After 10 minutes the reaction was allowed to stir at 0 °C for 24 hrs. The reaction was quenched by addition of two drops of Et_3N, diluted with 5 ml DCM then washed vigorously with 10 ml 1M NaOH. The Organic layer was purified by flash column chromatography (44-56% DE/hexanes (where DE= 5:1 DCM/EtOAc)) to give 90.7 mg of a colorless oil **15a**, yield 80%, *alpha* only. ^{1}H NMR spectra matched the previously published spectra.[12]

Glycosylation between acceptor 14 and donor 10b using DMF as additive.

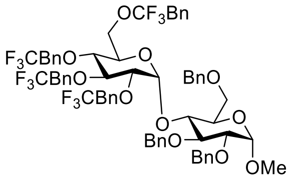

15b

To 2,3,4,6-tetra-*O*-(4-trifluoromethylbenzyl)-D-glucopyranose trichloroacetimidate donor (146.1mg, 0.1527mmol) (**10b**) in a 4 mL Wheaton vial with a stir bar, 1.0 mL of dichloromethane (DCM) and 200 µL of dimethylformamide (DMF) were added. The vial was capped and flushed with N_2 gas for 2 minutes then allowed to stir at -70 °C. 15.0 µL of TfOH was then added and solution allowed to stir. After 40 minutes, acceptor **14** (49.2 mg, 0.1059 mmols) dissolved in 1ml of DCM was added dropwise under N_2 atmosphere. After 10 minutes the reaction was allowed to stir at 0 °C for 24 hrs. The reaction was quenched by addition of two drops of Et_3N, diluted with 5 ml DCM then washed vigorously with 10 ml 1M NaOH. The Organic layer was purified by flash column chromatography (52-56% DE/hexanes (where DE= 3:1

DCM/EtOAc)) to give 90.7 mg of a colorless oil **15b**, yield 68%, *alpha* only. ^{1}H NMR spectra matched the previously published spectra.[6]

Glycosylation using linker acceptor 16 with donor 11a

Following the general procedure **A**, 2.0 ml of dichloromethane was used, 107.3 mg (0.1507 mmol) of 2,3,4,6-tetra-O-benzyl-D-glucopyranose *N*-phenyl trifluoroacetimidate (**11a**), 22.3 mg (0.107 mmol) of acceptor **16,** 251.4 mg of triphenylphosphine oxide (0.9034 mmol, 5.99 equiv.) and 22.5 μL (0.158 mmol) of TMSI (1.05 equiv.) were used. Purified by flash column chromatography (32-40% EtOAc/hexanes) to give 66.3 mg of a colorless oil **16a**, yield 85%, *alpha*:*beta* ratio 13:1.

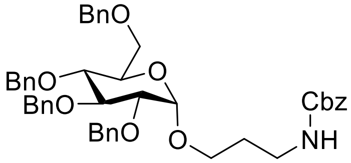

16a
alpha

Data for *alpha* anomer 16a: ^{1}H NMR (400 MHz, $CDCl_3$) δ; 7.47-7.03 (m, 25H), 5.92 (t, *J* = 5.6 Hz, 1H), 5.04 (s, 2H), 4.87 (d, *J* = 10.8 Hz, 1H), 4.80 (d, *J* = 10.7 Hz, 1H), 4.77-4.69 (m, 2H), 4.67 (d, *J* = 3.6 Hz, 1H), 4.59 (d, *J* = 12.1 Hz, 1H), 4.55 (d, *J* = 12.1Hz, 1H), 4.50-4.41 (m, 2H), 3.94 (t, *J* = 9.3 Hz, 1H), 3.87-3.79 (m, 1H), 3.74 (dt, *J* = 10.1, 3.2 Hz, 1H), 3.68-3.47 (m, 5H), 3.43-3.36 (m, 1H), 3.25-3.16 (m, 1H), 1.89-1.74 (m, 2H); **^{13}C NMR (125 MHz, $CDCl_3$) δ;** 156.6, 138.8, 138.2, 137.9, 136.8, 128.5, 128.4, 128.4, 128.4, 128.1, 128.0, 128.0, 128.0, 128.0, 127.9, 127.9, 127.7, 127.6, 97.5, 82.2, 79.8, 77.6, 77.3, 75.8, 75.1, 73.5, 73.4, 70.6, 68.5, 67.6, 66.5, 39.7. **HRMS (ESI) m/z:** [M+Na]+ Calcd. for $C_{45}H_{49}NO_8Na$ 754.3350; found 754.3347. **$[\alpha]_D^{25}$ =** +12.2° (*c* 1, CH_2Cl_2). **IR (cm^{-1})** 2923, 1719, 1519, 1070, 1028, 735, 698.

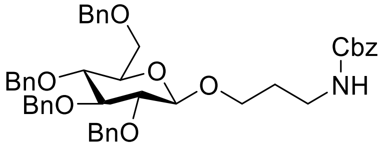

16a
beta

Data for *beta* anomer 16a: ^{1}H NMR (500 MHz, $CDCl_3$) δ; 7.40-7.09 (m, 25H), 5.41 (t, *J* = 6.1 Hz, 1H), 5.15-5.01 (m, 2H), 4.89 (t, *J* = 11.1 Hz, 2H), 4.81-4.76 (m, 2H), 4.71 (d, *J* = 11.1 Hz, 1H), 4.55-4.45 (m, 3H), 4.37(d, *J* = 7.8 Hz, 1H), 3.92 (m, 1H), 3.75-3.66 (m, 2H), 3.65-3.56 (m, 2H), 3.53 (t, *J* = 9.3 Hz, 1H), 3.47-3.39 (m, 2H), 3.39-3.24 (m, 2H), 1.91-1.74 (m, 2H); **^{13}C NMR (125 MHz, $CDCl_3$) δ;** 156.6, 138.9, 138.5, 138.0, 138.0, 136.8, 128.5, 128.4, 128.4, 128.2, 128.1, 128.0, 128.0, 127.9, 127.9, 127.8, 127.7, 127.7, 127.7, 103.6, 84.7, 82.3, 77.9, 75.7, 75.0, 74.9, 74.6, 73.4, 68.8, 67.5, 66.5, 38.1, 29.7; **HRMS (ESI) m/z:** [M+Na]+ Calcd. for $C_{45}H_{49}NO_8Na$ 754.3350; found 754.3348. **$[\alpha]_D^{25}$ =** -29.7° (*c* 1, CH_2Cl_2). **IR (cm^{-1})** 2869, 1720, 1520, 1453, 1247, 1067, 1028, 735, 697.

Glycosylation using linker acceptor 16 with donor 11b

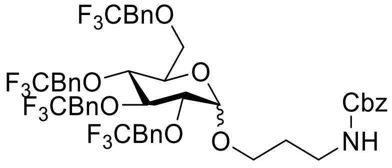

16b

Following the general procedure **A**, 2.0 ml of dichloromethane was used, 147.9 mg (0.1503 mmol) of 2,3,4,6-tetra-*O*-(4-trifluoromethylbenzyl)-D-glucopyranose *N*-phenyl trifluoroacetimidate donor (**11b**), 22.6 mg (0.108 mmol) of acceptor **16,** 251.3 mg of triphenylphosphine oxide (0.9030 mmol, 6.01 equiv.) and 22.5 µL (0.158 mmol) of TMSI (1.05 equiv.) were used. Purified by flash column

chromatography (36-56% EtOAc/hexanes) to give 107.5 mg of a colorless oil **16b**, yield 99%, *alpha*:*beta* ratio 23:1. Spectra match what is reported in literature.[6]

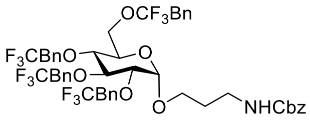

16b *alpha*

Data for *alpha* anomer 16b: ^{1}**H NMR** (400 MHz, $CDCl_3$) δ 7.59-7.17 (m, 21H), 5.76 (t, *J* = 5.7 Hz, 1H), 5.04 (s, 2H), 4.86-4.76 (m, 3H), 4.71-4.58 (m, 4H), 4.51 (m, 2H), 3.99-3.86 (m, 2H), 3.80-3.74 (d, *J* = 9.6 Hz, 1H), 3.70-3.51 (m, 5H), 3.51-3.42 (m, 1H), 3.31-3.21 (m, 1H), 1.85 (m, 2H); ^{13}**C NMR** (125 MHz, $CDCl_3$) δ 156.5, 142.4, 141.9, 141.8, 136.6, 130.4-128.7 (m), 128.4, 128.1, 128.0, 127.8, 127.7, 127.7, 127.4, 127.3, 125.5-125.1 (m), 122.9, 97.0, 82.2, 80.1, 77.7, 74.6, 74.1, 72.7, 72.3, 70.4, 68.7, 67.7, 66.6, 39.7, 29.2; ^{19}**F NMR** (471 MHz, $CDCl_3$) δ -63.16 (s, 9F), -63.23 (s, 3F). **HRMS (ESI) m/z:** [M+H]+ Calcd. for $C_{49}H_{46}F_{12}NO_8$ 1004.3032; found 1004.3061. **$[\alpha]_D^{25}$ =** +26.6° (*c* 1, CH_2Cl_2). **IR (cm^{-1})** 3054,2917, 1716, 1516, 1324,1264, 1163, 1123, 1065, 1018, 823 (*p*-disubstituted benzene), 735, 703.

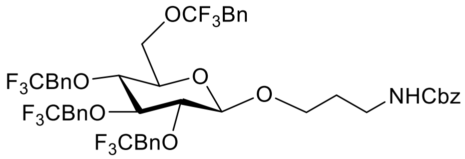

16b *beta*

Data for *beta* anomer 16b: **1H NMR** (400 MHz, $CDCl_3$) δ 7.56-7.26 (m, 19H), 7.18 (d, *J* = 8.0 Hz, 2H), 5.29 (t, *J* = 5.9 Hz, 1H), 5.06 (d, *J* = 4.7 Hz, 2H), 4.95 (d, *J* = 12.0 Hz, 1H), 4.88 (d, *J* = 12.1 Hz, 1H), 4.76 (m, 2H), 4.69 (d, *J* = 12.0 Hz, 1H), 4.59-4.54 (m, 2H), 4.48 (d, *J* = 12.8 Hz, 1H), 4.40 (d, *J* = 7.7 Hz, 1H), 3.97-3.91 (m, 1H), 3.73-3.64 (m, 3H), 3.63-3.56 (m, 2H), 3.50-3.27 (m, 4H), 1.83 (m, 2H); ^{13}**C NMR** (125 MHz, $CDCl_3$) δ 156.4, 142.3, 142.2, 141.9, 141.8, 136.6, 130.4-129.6 (m), 128.5, 128.2, 128.1, 127.8, 127.7, 127.4, 127.2, 125.4-125.1 (m), 123.0, 123.0, 122.9, 120.8, 103.5, 84.6, 82.2, 78.0, 74.6, 74.4, 74.0, 73.8, 72.6, 68.9, 67.5, 66.7, 38.1, 29.7**,** ^{19}**F**

NMR (471 MHz, $CDCl_3$) δ -63.12 (s, 3F), -63.14 (s, 3F), -63.19 (s, 3F), -63.24 (s, 3F) **HRMS (ESI) m/z:** [M+H]+ Calcd. for $C_{49}H_{46,}F_{12}NO_8$ 1004.3032; found 1004.3061. **$[\alpha]_D^{25}$ =** +52.4° (*c* 1, CH_2Cl_2). **IR (cm^{-1})** 3054, 2917, 1716, 1516, 1324, 1264, 1163, 1123, 1065, 1018, 823 (*p*-disubstituted benzene), 735, 703.

Glycosylation using linker acceptor 17 with donor 11a

Following the general procedure **A**, 2.0 ml of dichloromethane was used, 106.8 mg (0.1501 mmol) of 2,3,4,6-tetra-*O*-benzyl-D-glucopyranose *N*-phenyl trifluoroacetimidate (**11a**), 35.2 mg (0.108 mmol) of acceptor **17,** 251.1 mg of triphenylphosphine oxide 0.9023 mmol (6.02 equiv.) and 22.5 μL (0.158 mmol) of TMSI (1.05 equiv.) were used. Purified by flash column chromatography (20-28% EtOAc/hexanes) to give 77.7 mg of a colorless oil **17a**, yield 85%, *alpha*:*beta* ratio 11:1.

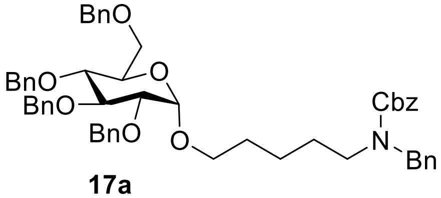

17a
alpha

Data for *alpha* anomer 17a: ^{1}H NMR (500 MHz, $CDCl_3$) δ 7.40-7.08 (m, 30H), 5.15 (m, 2H), 4.97(d, *J* = 10.8 Hz, 1H), 4.84-4.69 (m, 4H), 4.66-4.57 (m, 2H), 4.51-4.43 (m, 4H),3.96 (t, *J* = 9.3 Hz, 1H), 3.76-3.67 (m, 2H), 3.64-3.51 (m, 4H), 3.40-3.29 (m, 1H), 3.27-3.14 (m, 2H), 1.68-1.44 (m, 4H), 1.38-1.19 (m, 2H); **^{13}C NMR (125 MHz, $CDCl_3$) δ;** 156.8, 156.5, 138.9, 138.3, 138.2, 137.9, 139.9, 128.6, 128.5, 128.5, 128.4, 128.4, 128.4, 128.1, 128.0, 128.0, 127.9, 127.9, 127.8, 127.6, 127.2, 97.0, 82.1, 80.0, 75.8, 75.2, 73.5, 73.2, 70.1, 68.4, 68.0, 67.2, 50.5, 50.2, 47.2, 46.2, 29.2, 28.0, 27.6, 23.5. **HRMS (ESI) m/z:** [M+K]+ Calcd. for $C_{54}H_{59}NO_8K$ 888.3872; found 888.3877. **$[\alpha]_D^{25}$ =** -1.5° (*c* 1, CH_2Cl_2). **IR (cm^{-1})** 2924, 1698, 1454, 1071, 1028, 734, 698.

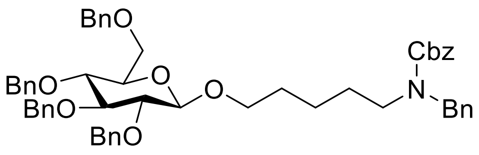

17a
beta

Data for *beta* anomer 17a: ^{1}H NMR (500 MHz, $CDCl_3$) δ; 7.44-7.06 (m, 30H), 5.16 (m, 2H), 4.91 (d, *J* = 10.9 Hz, 2H), 4.84-4.75 (m, 2H), 4.70 (d, *J* = 11.0 Hz, 1H), 4.60 (d, *J* = 12.2 Hz, 1H), 4.56-4.50 (m, 2H), 4.46 (m, 2H), 4.35 (t, *J* = 8.1 Hz, 1H), 3.96-3.83 (m, 1H), 3.73 (dd, *J* = 10.8, 2.0 Hz, 1H), 3.70-3.60 (m, 2H), 3.57 (t, *J* = 9.3 Hz, 1H), 3.52-3.40 (m, 3H), 3.29-3.12 (m, 2H), 1.69-1.45 (m, 4H), 1.42-1.21(m, 2H). **^{13}C NMR (125 MHz, $CDCl_3$) δ;** 156.8, 156.2, 138.7, 138.6, 138.3, 138.2, 138.0, 136.9, 128.6, 128.5, 128.4, 128.4, 128.4, 128.3, 128.0, 128.0, 127.9, 127.9, 127.8, 127.8, 127.7, 127.6, 127.3, 127.2, 103.7, 84.8, 82.3, 78.0, 75.7, 75.0, 74.9, 74.8, 73.5, 69.9, 69.1, 67.2, 53.5, 50.6, 50.3, 47.2, 46.3, 29.5, 28.0, 27.6, 23.5. **HRMS (ESI) m/z:** [M+Na]+ Calcd. for $C_{54}H_{59}NO_8Na$ 872.4133; found 872.4128. **$[\alpha]_D^{25}$ =** -20.6° (*c* 1, CH_2Cl_2). **IR (cm^{-1})**. 2927, 2864, 1699, 1496, 1454, 1421, 1069, 1028, 735, 698.

Glycosylation using linker acceptor 17 with donor 11b

Following the general procedure **A**, 2.0 ml of dichloromethane was used, 148.3 mg (0.1507 mmol) of 2,3,4,6-tetra-*O*-(4-trifluoromethylbenzyl)-D-glucopyranose *N*-phenyl trifluoroacetimidate donor (**11b**), 35.2 mg (0.108 mmol) of acceptor **17,** 251.1 mg of triphenylphosphine oxide (0.9023 mmol, 5.99 equiv.) and 22.5 µL (0.158 mmol) of TMSI (1.05 equiv.) were used. Purified by flash column chromatography (28-32% EtOAc/hexanes) to give 102.4 mg of a colorless oil **17b**, yield 85%, *alpha*:*beta* ratio 16:1.

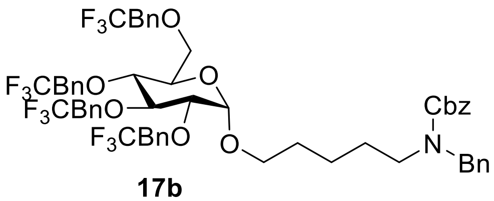

17b
alpha

Data for 17b *alpha* anomer: ^{1}H NMR (500 MHz, $CDCl_3$) δ 7.66-7.06 (m, 26H), 5.22-5.10 (m, 2H), 4.98 (d, *J* = 12.0 Hz, 1H), 4.88-4.76 (m, 3H), 4.73-4.61 (m, 3H), 4.59-4.45 (m, 4H), 3.98 (t, *J* = 9.2 Hz, 1H), 3.82-3.70 (m, 2H), 3.70-3.59 (m, 3H), 3.56 (dd, *J* = 9.6, 3.5 Hz, 1H), 3.37 (m, 1H), 3.30-3.16 (m, 2H), 1.71-1.48 (m, 4H), 1.42-1.21 (m, 2H); **^{13}C NMR (125 MHz, $CDCl_3$) δ** 156.8, 156.3, 142.7, 142.1, 141.9, 137.9, 136.9, 136.7, 130.6-129.3 (m), 128.6, 128.5, 128.0, 127.9, 127.8, 127.7, 127.4, 127.4, 127.2, 125.5, 125.5, 125.4, 125.4, 125.4, 125.3, 125.3, 125.2, 125.2, 123.1, 123.0, 120.9, 96.6, 82.1, 80.4, 77.8, 74.6, 74.1, 72.7, 72.1, 70.1, 68.7, 68.2, 67.2, 50.5, 50.2, 47.1, 46.1, 29.2, 28.0, 27.6, 23.5. **^{19}F NMR (471 MHz, $CDCl_3$) δ -**63.05 - -63.12(m, 9F), -63.16 (s, 3F). **HRMS (ESI) m/z:** [M+Na]+ Calcd. for $C_{58}H_{55}F_{12}NO_8Na$ 1144.3628; found 1144.3633. **$[\alpha]_D^{25}$ =** -1.3° (*c* 1, CH_2Cl_2). **IR (cm^{-1})**. 2928, 1698, 1421, 1324, 1162, 1120, 1065, 1018, 823 (*p*-disubstituted benzene), 699.

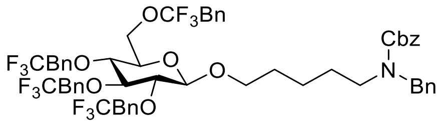

17b *beta*

Data for 17b beta anomer: ^{1}H NMR (500 MHz, $CDCl_3$) δ; 7.67-7.04 (m, 26H), 5.23-5.09 (m, 2H), 5.01-4.86 (m, 2H), 4.84-4.52 (m, 6H), 4.51-4.42 (m, 2H), 4.36 (m, 1H), 3.90 (m, 1H), 3.73-3.72 (m, 2H), 3.65-3.55 (m, 2H), 3.53-3.37 (m, 3H), 3.29-3.11 (m, 2H), 1.70-1.45 (m, 4H), 1.40-1.18 (m, 2H); **^{13}C NMR (125 MHz, $CDCl_3$) δ** 156.7, 156.2, 142.4, 142.1, 142.0, 137.9, 136.9, 136.7, 130.3-129.3 (m), 128.6, 128.5, 128.0, 127.8, 127.8, 127.7, 127.4, 127.3, 127.1, 125.4, 125.4, 125.3, 125.3, 125.3, 125.2, 125.1, 123.0, 123.0, 123.0, 103.5, 84.6, 82.2, 77.9, 74.6, 74.6, 74.0, 73.7, 72.7, 70.0, 69.0, 67.2, 50.5, 50.2, 47.1, 46.0, 29.4, 27.9, 27.5, 23.4; **^{19}F NMR**

(471 MHz, $CDCl_3$) δ -63.04 (s, 6F), -63.13 (s, 3F), -63.16 (s, 3F); **HRMS (ESI) m/z:** [M+Na]+ Calcd. for $C_{58}H_{55}F_{12}NO_8Na$ 1144.3628; found 1144.3627. **$[\alpha]_D^{25}$ =** -18.7° (*c* 1, CH_2Cl_2). **IR (cm^{-1})**. 2931, 1698, 1421, 1324, 1162, 1120, 1065, 1018, 821 (*p*-disubstituted benzene), 699.

Glycosylation using linker acceptor 17 with donor 11c

Following the general procedure **A**, 2.0 ml of dichloromethane was used, 189.7 mg (0.1511 mmol) of 2,3,4,6-tetra-O-3,5-*bis*(trifluoromethyl)benzyl-D-glucopyranose *N*-phenyl trifluoroacetimidate (**11c**), 34.1 mg (0.104 mmol) of acceptor **17,** 257.7 mg of triphenylphosphine oxide (0.9260 mmol, 6.12 equiv.) and 22.5 µL (0.158 mmol, 1.05 equiv.) of TMSI were used. Purified by flash column chromatography (5-20% EtOAc/hexanes) to give 118.8 mg of a pale-yellow oil **17c**, 81% *alpha*:*beta* ratio 31:1.

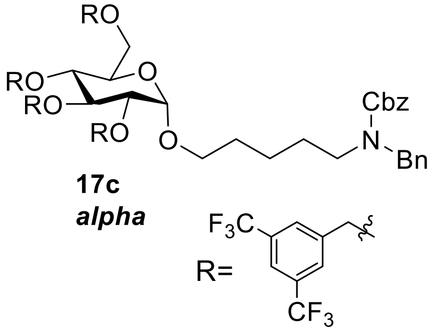

17c
alpha

Data for *alpha* anomer 17c: ^{1}H NMR (400 MHz, $CDCl_3$) δ 7.81 (s, 2H), 7.80 (s, 2H), 7.73 (m, 4H), 7.62 (d, *J* = 10.2 Hz, 4H), 7.34-7.16 (m, 10H), 5.15 (m, 2H), 5.03 (d, *J* = 13.0 Hz, 1H), 4.92-4.60 (8H), 4.48 (m, 2H), 4.03 (t, *J* = 9.2 Hz, 1H), 3.84 (m,2H), 3.72-3.61 (m, 4H), 3.40 (m, 1H), 3.23 (m, 2H), 1.63-1.55 (m, 4H), 1.33-1.21 (m, 2H). **^{13}C NMR (125 MHz, $CDCl_3$) δ** 156.7, 156.2, 141.1, 140.6, 137.9, 136.9, 136.7, 132.1, 131.9, 131.9, 131.8, 131.8, 131.7, 131.6, 131.6, 131.5, 131.4, 131.2, 128.6, 128.5, 128.0, 127.8, 127.3, 127.1, 127.0, 126.5, 126.3, 124.3, 124.2, 122.2,

122.1, 121.7, 121.7, 121.3, 120.0, 119.9, 96.2, 82.3, 80.9, 78.1, 73.8, 73.3, 72.1, 71.0, 69.9, 69.2, 68.3, 67.2, 50.5, 50.2, 47.0, 46.0, 29.1, 27.9, 27.4, 23.3.**^{19}F NMR (471 MHz, $CDCl_3$) δ -**63.48 (s, 6F), -63.54 (s, 6F), -63.60 (s, 6F), -63.70 (s, 6F). **HRMS (ESI) m/z:** [M+H]+ Calcd. for $C_{62}H_{51}F_{24}NO_8$ 1394.3310; found 1394.3291. **$[\alpha]_D^{25}$ =** -672.8° (*c* 0.24, CH_2Cl_2). **IR (cm^{-1})** 2923, 2854, 1697, 1464, 1356, 1276, 1169, 1125, 884, 843, 803, 733, 704, 682 (*m*-disubstituted aromatic).

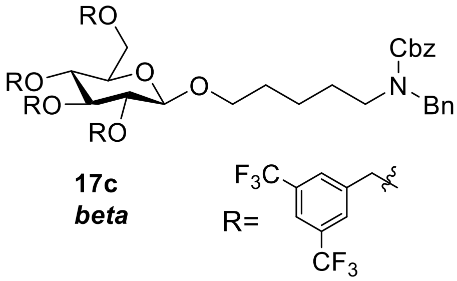

17c
beta

Data for *beta* anomer 17c: ^{1}H NMR (500 MHz, $CDCl_3$) δ 7.79 (d, *J* = 6.6 Hz, 3H), 7.73 (s, 2H), 7.69 (m, 3H), 7.59 (d, *J* = 8.6 Hz, 4H), 7.34-7.14 (m, 10H), 5.16 (m, 2H), 5.07 (m, 1H), 4.97 (d, *J* = 13.0 Hz, 1H), 4.87-4.61 (m, 6H), 4.46 (m, 3H), 3.88-3.79 (m, 3H), 3.72 (m, 2H), 3.53-3.45 (m, 3H), 3.22 (m, 2H), 1.60 (m, 4H), 1.24 (m, 2H). **^{13}C NMR (125 MHz, $CDCl_3$) δ** 156.5, 156.2, 140.9, 140.8, 140.6, 140.5, 137.8, 131.9, 131.8, 131.7, 131.6, 131.6, 131.5, 131.4, 128.5, 128.4, 127.9, 127.8, 127.3, 127.1, 126.9, 126.4, 126.1, 124.3, 124.2, 124.2, 124.1, 122.1, 122.1, 122.0, 122.0, 121.5, 119.8, 103.2, 85.0, 82.6, 78.1, 74.4, 73.9, 73.2, 72.8, 72.1, 69.9, 69.2, 67.1, 50.4, 50.1, 46.9, 45.9, 29.3, 27.8, 27.3, 23.2, 22.7.**^{19}F NMR (471 MHz, $CDCl_3$) δ** -63.44 (6F), -63.54 (6F), -63.63 (6F), -63.74 (6F). **HRMS (ESI) m/z:** [M+H]+ Calcd. for $C_{62}H_{51}NO_8F_{24}$ 1394.3310; found 1394.3311. **$[\alpha]_D^{25}$ =** -346.3° (*c* 0.39, CH_2Cl_2) **IR (cm^{-1})** 2923, 1697, 1624, 1464, 1423, 1377, 1356, 1278, 1171, 1128, 883, 843, 801, 734, 704, 682 (*m*-disubstituted aromatic).

Glycosylation using acceptor 18 and donor 11b

Following the general procedure **A**, 2.0 ml of dichloromethane was used, 148.1 mg (0.1505 mmol) of 2,3,4,6-tetra-*O*-(4-trifluoromethylbenzyl)-D-glucopyranose *N*-phenyl trifluoroacetimidate donor (**11b**), 10.6 mg (0.105 mmol) of acceptor **18,** 251.2 mg of triphenylphosphine oxide (0.9027 mmol, 6.00 equiv.) and 22.5 µL (0.158 mmol) of TMSI (1.05 equiv.) were used. Purified by flash column chromatography (28-32% EtOAc/hexanes) to give 91.0 mg of a colorless oil **18b**, yield 97%, *alpha*:*beta* ratio 22:1.

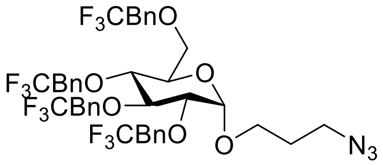

18b
alpha

Data for 18b *alpha* anomer: ^{1}H NMR (400 MHz, $CDCl_3$) δ; 7.60-7.18 (m, 16H), 4.98 (d, *J* = 12.0 Hz, 1H), 4.88 (d, *J* = 3.6 Hz, 1H), 4.85-4.78 (m, 2H), 4.72 (s, 2H), 4.66 (d, *J* = 12.6 Hz, 1H), 4.60-4.48 (m, 2H), 3.98 (t, *J* = 9.2 Hz, 1H), 3.86-3.73 (m, 3H), 3.70-3.62 (m, 2H), 3.58 (dd, *J* = 9.6, 3.6 Hz, 1H), 3.54-3.41 (m, 3H), 1.98-1.86 (m, 2H); **^{13}C NMR (125 MHz, $CDCl_3$) δ;** 142.6, 142.0, 141.9, 130.4-129.5 (m), 127.7, 127.7, 127.4, 127.3, 125.6-125.1 (m), 122.9, 96.9, 82.0, 80.4, 77.8, 74.6, 74.1, 72.7, 72.2, 70.3, 68.8, 64.9, 48.2, 28.8; **^{19}F NMR (471 MHz, $CDCl_3$) δ;** -63.08 (s, 3F), -63.09 (s, 3F), -63.10 (s, 3F), -63.15 (s, 3F); **HRMS (ESI) m/z:** [M+Na]+ Calcd. for $C_{41}H_{37}F_{12}N_3O_6Na$ 918.2383; found 918.2373. **$[\alpha]_D^{25}$ =** +13.0° (*c* 1, CH_2Cl_2). **IR (cm^{-1})** 2925, 2098, 1324, 1161, 1120, 1066, 101, 822 (*p*-disubstituted benzene), 594.

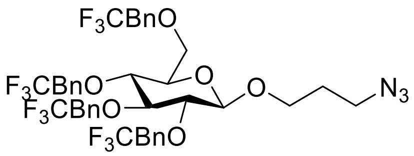

18b
beta

Data for *beta* anomer 18b: **^{1}H NMR (500 MHz, $CDCl_3$) δ;** 7.61-7.19 (m, 16H), 4.95 (d, *J* = 12.0 Hz, 1H), 4.89 (d, *J* = 12.1 Hz, 1H), 4.82-4.54 (m, 6H), 4.41(d, *J* = 7.8 Hz, 1H), 4.00 (dt, *J* = 10.4, 6.0 Hz, 1H), 3.73-3.74 (m, 2H), 3.68-3.60 (m, 3H), 3.51-3.36 (m, 4H), 1.95-1.83 (m, 2H); **^{13}C NMR (125 MHz, $CDCl_3$) δ** 142.3, 142.3, 142.1, 141.9, 130.2-129.4 (m), 127.7, 127.6, 127.4, 127.3, 125.5-125.1 (m), 123.0, 103.56, 84.7, 82.3, 77.9, 74.7, 74.6, 74.0, 73.8, 72.8, 69.0, 66.7, 48.2, 29.3; **^{19}F NMR (471 MHz, $CDCl_3$) δ** -63.06 (s, 3F), -63.09 (s. 3F), -63.12 (s, 3F), -63.15 (s, 3F) **HRMS (ESI) m/z:** [M+Na]+ Calcd. for $C_{41}H_{37}F_{12}N_3O_6Na$ 918.2383; found 918.2373. **$[\alpha]_D^{25}$ =** -16.0° (*c* 1, CH_2Cl_2). **IR (cm^{-1})** 2874, 2099, 1326, 1162, 1326, 1162, 1121, 1066, 1018, 822 (*p*-disubstituted benzene), 593.

Glycosylation using acceptor 18 and donor 11c

Following the general procedure **A**, 2.0 ml of dichloromethane was used, 189.8 mg (0.1511 mmol) of 2,3,4,6-tetra-*O*-3,5-*bis*(trifluoromethyl)benzyl-D-glucopyranose *N*-phenyl trifluoroacetimidate (**11c**), 10.5 mg (0.104 mmol) of acceptor **18,** 255.3 mg of triphenylphosphine oxide (0.9174 mmol, 6.07 equiv.) and 22.5 µL (0.158 mmol) of TMSI (1.05 equiv.) were used. Purified by flash column chromatography (5-10% EtOAc/hexanes) to give 118.8 mg of a pale-yellow oil **18c**, yield 97%, *alpha*:*beta* ratio 34:1.

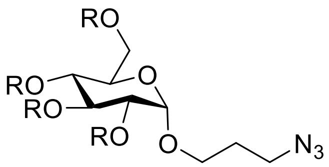

18c
alpha

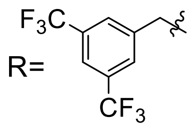

Data for 18c *alpha* anomer: ^{1}H NMR (500 MHz, CDCl3) δ 7.79 (s, 4H), 7.73 (s, 2H), 7.71 (s, 2H) 7.63 (d, *J* = 11.9 Hz, 4H), 5.04 (d, *J* = 12.9 Hz, 1H), 4.97 (d, *J* = 3.4 Hz, 1H), 4.89-4.79 (m, 2H), 4.77-4.71 (m, 4H), 4.66 (s, 1H), 4.04 (t, *J* = 9.2 Hz, 1H) 3.87 (m, 3H), 3.72 (t, *J* = 9.4 Hz, 2H), 3.66 (dd, *J* = 9.6 Hz, *J* = 3.5 Hz, 1H), 3.53 (m, 1H), 3.45 (t, *J* = 6.5 Hz, 2H), 1.94 (m, 2H). **^{13}C NMR (125 MHz, CDCl3) δ** 141.0, 140.6, 140.6, 140.5, 132.0, 131.9, 131.9, 131.8, 131.7, 131.7, 131.6, 130.9, 128.9, 127.3, 127.0, 126.4, 124.4, 124.2, 122.2, 122.1, 121.9, 121.6, 119.9, 96.5, 82.2, 80.9, 78.2, 73.9, 73.4, 72.1, 71.2, 70.2, 69.3, 65.0, 53.4, 48.2. **^{19}F NMR (471 MHz, CDCl3) δ** -63.51 (s, 6F), -63.60 (s, 6F), -63.62 (s, 6F) -63.71 (s, 6F) **$[\alpha]_D^{25}$ =** -312.7° (*c* 0.41, CH_2Cl_2). **IR (cm^{-1})** 2923, 2853, 2100 (azide), 1463, 1357, 1277, 1171, 1128, 884, 843, 801, 735, 705, 682 (*m*-disubstituted aromatic).

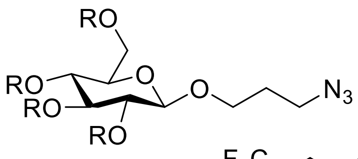

18c
beta

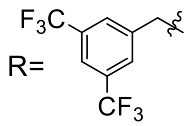

Data for *beta* anomer 18c: **^{1}H NMR (500 MHz, $CDCl_3$) δ** 7.80 (s, 3H), 7.79 (d, *J* = 9.45 Hz, 2H), 7.69 (s, 3H) 7.59 (d, *J* = 10.4 Hz, 4H), 5.06 (d, *J* = 12.6 Hz, 1H), 4.97 (d, *J* = 12.9 Hz, 1H), 4.86-4.66 (m, 6H), 4.48 (d, *J* = 7.8 Hz, 1H), 4.02 (m, 1H), 3.88-3.79 (m, 2H), 3.73 (m, 2H), 3.67 (m, 1H), 3.56 m 1H), 3.49 (t, *J* = 7.6 Hz, 1H), 3.36 (m, 2H), 1.88 (m, 2H). **^{13}C NMR (125 MHz, $CDCl_3$) δ** 140.8, 140.8, 140.7, 140.5, 132.2, 132.0, 131.9, 131.9, 131.9, 131.7, 131.7, 131.6, 131.6, 127.3, 126.9, 126.5, 126.2, 124.4, 124.3, 124.2, 122.2, 122.1, 122.0, 121.6, 120.0, 103.3, 85.0, 82.7, 78.2, 74.5, 73.9, 73.3, 73.0, 72.2, 69.3, 66.8, 48.2, 29.2. **^{19}F NMR (471 MHz, $CDCl_3$) δ -**63.47 (s, 6F), -63.61 (s, 6F), -63.63 (s, 6F) -63.75 (s, 6F) **$[\alpha]_D^{25}$ =** -202.6° (*c* 0.66, CH_2Cl_2). **IR (cm^{-1})** 2922, 2852, 2100 (azide), 1463, 1377, 1355, 1276, 1169, 1125, 884, 843, 800, 734, 705, 682 (*m*-disubstituted aromatic).

Glycosylation using cholesterol (19) as the acceptor and 11b as the donor.

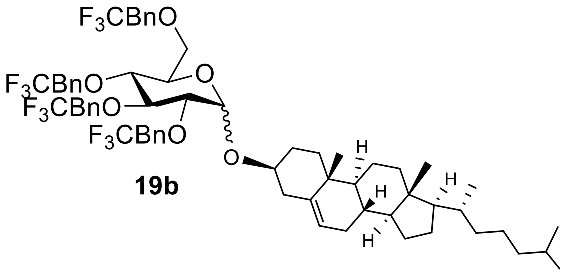

19b

Following the general procedure **A**, 2.0 ml of dichloromethane was used, 148.6 mg (0.1511 mmol) of 2,3,4,6-tetra-*O*-(4-trifluoromethylbenzyl)-D-glucopyranose *N*-phenyl trifluoroacetimidate donor (**11b**), 40.8 mg (0.106 mmol) of acceptor **19,** 251.8 mg of triphenylphosphine oxide (0.9048 mmol, 5.99 equiv.) and 22.5 µL (0.158 mmol) of TMSI (1.05 equiv.) were used. Purified by flash column chromatography (36-60% DE/hexanes (where DE= 3:1 DCM/EtOAc)) to give 117.5 mg of a colorless oil **19b**, yield 94%, *alpha*:*beta* ratio 15:1. ^{1}H NMR spectra of both anomeric products matched the previously published spectra.[6]

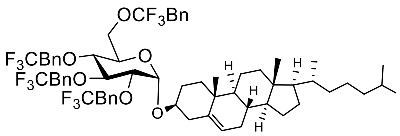

19b *alpha*

Data for *alpha* anomer 19b: ^{1}H NMR (500 MHz, $CDCl_3$) δ 7.56-7.50 (m, 8H), 7.43-7.40 (m, 4H), 7.35 (d, *J* = 8.0 Hz, 2H), 7.22 (d, *J* = 8.0 Hz, 2H), 5.29-5.27 (m, 1H), 5.05 (d, *J* = 3.7 Hz, 1H), 5.00 (d, *J* = 12.0 Hz, 1H), 4.81 (t, *J* = 11.9 Hz, 2H), 4.71 (s, 2H), 4.65 (d, *J* = 12.7 Hz, 1H), 4.56 (d, *J* = 11.9 Hz, 1H), 4.50 (d, *J* = 12.7 Hz, 1H), 4.00 (t, *J* = 9.2 Hz, 1H), 3.92 (m, 1H), 3.75 (dd, *J* = 10.7 Hz, 3.8 Hz 1H), 3.69-3.61 (m, 2H), 3.55 (dd, *J* = 9.6 Hz, 3.7 Hz, 1H), 3.46 (m, 1H), 2.44 (t, *J* = 13.3 Hz, 1H), 2.31 (ddd, *J* = 13.2 Hz, 5.0 Hz, 2.0 Hz, 1H), 2.02 (dt, *J* = 16.2 Hz, 3.5 Hz, 1H), 1.99-1.92 (m, 1H), 1.91-1.79 (m, 3H), 1.62-0.96 (m, 20H), 1.01 (s, 3H), 0.92 (d, *J* = 6.6 Hz, 4H), 0.87 (d, *J* = 6.6 Hz, 3H), 0.87 (d, *J* = 6.6 Hz, 3H), 0.68 (s, 3H); **^{13}C NMR (125 MHz, $CDCl_3$)** δ 142.7, 142.0, 141.9, 140.6, 130.1, 129.8, 127.9, 127.7, 127.3, 125.4-125.0 (m), 122.0, 94.5, 82.1, 80.3, 78.1, 74.6, 74.1, 72.7, 71.9, 70.0, 69.0, 56.8, 56.2, 50.1, 42.3, 40.0, 39.8, 39.5, 37.1, 36.8, 36.2, 35.8, 31.9, 31.9, 28.2, 28.0, 27.7, 24.3, 23.8, 22.8, 22.6, 21.1, 19.4, 18.7, 11.9**; ^{19}F NMR** (471 MHz, $CDCl_3$) δ -63.07 (s, 3F), -63.09 (s, 3F), -63.10 (s, 3F), -63.17 (s, 3F), **HRMS (ESI) m/z:** [M+Cl]- Calcd. for $C_{65}H_{76,}F_{12}O_6Cl$ 1215.5144; found 1215.5188. **$[\alpha]_D^{25}$ =** +26.6° (*c* 1, CH_2Cl_2). **IR (cm^{-1})** 2935, 2868, 1620, 1324, 1210, 1109, 1063, 1016, 822 (*p*-disubstituted benzene), 738, 592.

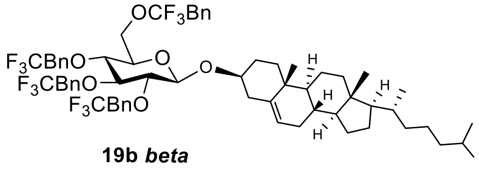

19b *beta*

Data for *beta* anomer 19b: ^{1}H NMR (500 MHz, $CDCl_3$) δ 7.56 (d, *J* = 8.0 Hz, 2H), 7.53-7.48 (m, 6H), 7.43 (d, *J* = 8.0 Hz, 2H), 7.39 (d, *J* = 8.0 Hz, 2H), 7.28 (d, *J* = 8.0 Hz, 2H), 7.24 (d, *J* = 8.0 Hz, 2H), 5.33 (d, *J* = 5.4 Hz, 1H), 5.01 (d, *J* = 12.0 Hz, 1H), 4.89 (d, *J* = 12.2 Hz, 1H), 4.80 (d, *J* = 12.0 Hz 1H), 4.76 (d, *J* = 12.1 Hz, 1H), 4.72 (d, *J* = 12.0 Hz, 1H), 4.67-4.57 (m, 3H), 4.52 (d, *J*

= 7.8 Hz, 1H), 3.71 (m, 2H), 3.64-3.55 (m, 3H), 3.47-3.41 (m, 2H), 2.36-2.25 (m, 2H), 2.04-1.95 (m, 3H), 1.88-1.79 (m, 2H), 1.68-0.95 (m, 20H), 1.01 (s, 3H), 0.92 (d, *J* = 6.5 Hz, 4H), 0.87 (d, *J* = 6.7 Hz, 3H), 0.86 (d, *J* = 6.7 Hz, 3H), 0.68 (s, 3H); **^{13}C NMR (125 MHz, $CDCl_3$)** δ 142.4, 142.2, 142.0, 130.1-129.7 (m), 127.9, 127.5, 127.4, 127.3, 125.3, 125.3, 125.3, 125.3, 125.2, 123.0, 122.9, 122.2, 102.1, 84.8, 82.3, 79.8, 78.1, 74.6, 74.0, 73.8, 72.6, 69.2, 56.8, 56.2, 53.4, 50.2, 42.3, 39.8, 39.5, 39.1, 37.3, 36.8, 36.2, 35.8, 31.9, 31.9, 30.0, 29.7, 28.2, 28.0, 24.3, 23.8, 22.8, 22.7, 22.7, 22.6, 21.1, 19.4, 18.7, 14.1, 11.9**; ^{19}F NMR** (471 MHz, $CDCl_3$) δ -63.04 (s, 3F), -63.08 (s, 3F) -63.13 (s, 3F), -63.16 (s, 3F) **HRMS (ESI) m/z:** [M+Cl]- Calcd. for $C_{65}H_{76,}F_{12}O_6Cl$ 1215.5144; found 1215.5186. **$[\alpha]_D^{25}$ =** +34.5° (*c* 1, CH_2Cl_2). **IR (cm^{-1})** 2936, 1325**,** 1264, 1165, 1123, 1065, 1018, 823 (*p*-disubstituted benzene), 735, 704.

Glycosylation using cholesterol (19) as the acceptor and 11c as the donor

Following the general procedure **A**, 2.0 ml of dichloromethane was used, 188.6 mg (0.1502 mmol) of 2,3,4,6-tetra-*O*-3,5-*bis*(trifluoromethyl)benzyl-D-glucopyranose *N*-phenyl trifluoroacetimidate (**11c**), 40.8 mg (0.106 mmol) of acceptor **19,** 259.8 mg of triphenylphosphine oxide (0.9336 mmol, 6.21 equiv.) and 22.5 µL (0.158 mmol) of TMSI (1.05 equiv.) were used. Purified by flash column chromatography (1-5% ethyl acetate/hexanes (thin-layer chromatography conditions 20% DE/hexanes (where DE= 3:1 DCM/EtOAc)) to give 141.9 mg of a yellow oil **19c**, yield 93%, *alpha*:*beta* ratio 23:1.

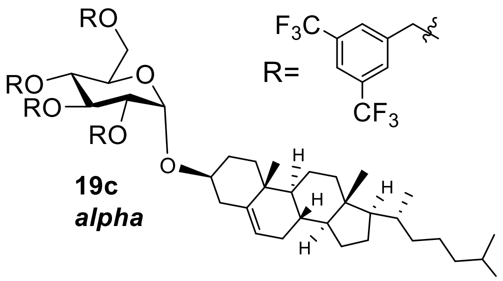

19c
alpha

Data for *alpha* anomer 19c: ^{1}H NMR (500 MHz, $CDCl_3$) δ 7.79 (s, 4H), 7.74 (m, 4H), 7.63 (d, *J* = 10.8 Hz, 4H), 5.29 (d, *J* = 5.1 Hz, 1H), 5.15 (d, *J* = 3.6 Hz, 1H), 5.05 (d, *J* = 12.9 Hz, 1H), 4.88-4.78 (m, 3H), 4.75-4.69 (m, 4H), 4.64 (d, *J* = 13.0 Hz, 1H), 4.07 (t, *J* = 9.2 Hz, 1H), 3.99 (d, *J* = 9.4 Hz, 1H), 3.88 (dd, *J* = 11.0 Hz, *J* = 3.4 Hz, 1H), 3.74-3.70 (m, 2H), 3.64 (dd, *J* = 9.6 Hz, *J* = 3.7 Hz, 1H), 4.97 (m, 1H), 2.48 (m, 1H), 2.34 (m, 1H), 2.04-1.93 (m, 2H), 1.89-1.78 (m, 3H), 1.52-1.26 (m, 10H), 1.23-1.04 (m, 9H), 1.02 (s, 3H),1.00-0.97 (m, 1H), 0.93 (d, *J* = 6.5 Hz, 3H), 0.88 (d, *J* = 1.7 Hz, 3H), 0.86 (d, *J* = 1.7 Hz, 3H), 0.68 (s, 3H). **^{13}C NMR (125 MHz, CDCl3) δ** 141.1, 140.6, 140.6, 140.5, 140.3, 132.0, 131.9, 131.9, 131.8, 131.7, 131.7, 131.6, 131.5, 127.4, 127.1, 126.4, 124.3, 124.2, 122.2, 122.1, 94.3, 82.3, 80.7, 78.3, 78.0, 73.8, 73.4, 72.1, 70.9, 70.0, 69.3, 56.7, 56.2, 50.1, 42.3, 40.0, 39.8, 39.5, 37.0, 36.7, 36.2, 35.8, 31.9, 31.9, 28.2, 28.0, 27.9, 24.3, 23.8, 22.8, 22.6, 21.1, 19.3, 18.7, 11.9. **^{19}F NMR (471 MHz, $CDCl_3$) δ -**63.47 (s, 6F), -63.57 (s, 6F), -63.60, -63.70 (s, 6F) **HRMS (ESI) m/z:** [M+Na]+ Calcd. $C_{69}H_{72}O_6F_{24}$ 1475.4843; found 1475.4811. **$[\alpha]_D^{25}$ = +**49.3° (*c* 1.0, CH_2Cl_2) **IR (cm^{-1})** 2930, 1624, 1464, 1356, 1276, 1169, 1127, 1036, 908, 884, 843, 733, 705, 682 (*m*-disubstituted aromatic).

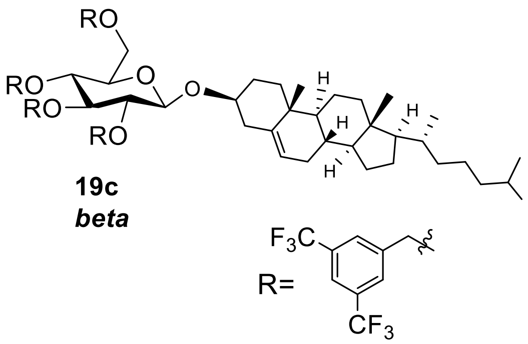

19c
beta

Data for *beta* anomer 19c: ^{1}H NMR (500 MHz, $CDCl_3$) δ 7.80 (d, *J* = 10.3 Hz, 3H), 7.75 (d, *J* = 7.9 Hz, 2H), 7.69 (s, 3H), 7.59 (d, *J* = 10.9 Hz, 4H) 5.31 (d, *J* = 5.5 Hz, 1H), 5.13 (d, *J* = 12.7 Hz, 1H), 4.97 (d, *J* = 12.9 Hz, 1H), 4.86 (m, 2H), 4.77 (d, *J* = 5.2 Hz, 1H), 4.75-4.67 (m, 4H), 4.59 (d, *J* = 7.8 Hz, 1H), 3.83 (m, 2H), 3.73 (m, 2H), 3.60 (m, 2H), 3.47 (t, *J* = 8.4 Hz, 1H), 2.30-2.19 (m,

2H), 2.03-1.94 (m, 3H), 1.87-1.79 (m, 2H), 1.66-1.57 (m, 2H), 1.53-1.29 (m, 8H), 1.19-1.01 (m, 9H), 0.97 (s, 3H), 0.93 (m, 4H), 0.87 (d, *J* = 6.6 Hz, 3H), 0.86 (d, *J* = 6.6 Hz, 3H), 0.68 (s, 3H). **^{13}C NMR (125 MHz, CDCl3) δ** 141.0, 140.8, 140.8, 140.5, 140.0, 131.9, 131.9, 131.8, 131.7, 131.6, 131.6, 127.3, 127.0, 126.5, 126.2, 124.4, 124.3, 124.2, 124.2, 122.3, 122.2, 122.1, 122.0, 121.5, 101.9, 85.1, 82.6, 80.0, 78.3, 74.5, 73.9, 73.3, 72.9, 72.3, 69.5, 56.8, 56.2, 50.2, 42.3, 39.8, 39.5, 39.1, 37.1, 36.7, 36.2, 35.8, 31.9, 31.8, 30.0, 28.3, 28.0, 24.3, 23.8, 22.8, 22.6, 21.0, 19.3, 18.7, 11.9. **^{19}F NMR (471 MHz, $CDCl_3$) δ** -63.41 (s, 6F), -63.56 (s, 6F), -63.62 (s, 6F), -63.74 (s, 6F) **HRMS (ESI) m/z:** [M+K]+ Calcd. for $C_{69}H_{72}F_{24}O_6$ 1491.4583; found 1491.4586. **$[\alpha]_D^{25}$ =** -207.5° (*c* 0.66, CH_2Cl_2) **IR (cm^{-1})** 2934, 1624, 1465, 1377, 1354, 1277, 1171, 1129, 884, 843, 802, 733, 706, 682 (*m*-disubstituted aromatic).

Glycosylation using acceptor 20 with donor 11b

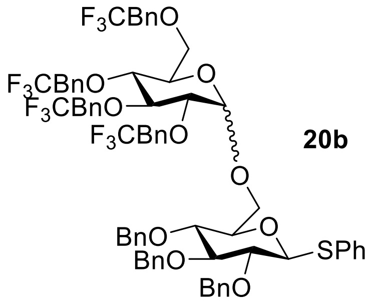

20b

Following the general procedure **A**, 2.0 ml of dichloromethane was used, 148.7 mg (0.1512 mmol) of 2,3,4,6-tetra-*O*-(4-trifluoromethylbenzyl)-D-glucopyranose *N*-phenyl trifluoroacetimidate donor (**11b**), 57.3 mg (0.106 mmol) of acceptor **20,** 251.6 mg of triphenylphosphine oxide (0.9041 mmol, 5.98 equiv.) and 22.5 µL (0.158 mmol) of TMSI (1.04 equiv.) were used. Purified by flash column chromatography (28-36% DE/hexanes (where DE = 3:1 DCM/EtOAc)) to give 132.3 mg of a colorless oil **20b**, yield 93%, *alpha*:*beta* ratio 25:1. ^{1}H NMR spectra of both anomeric products matched the previously published spectra.[6]

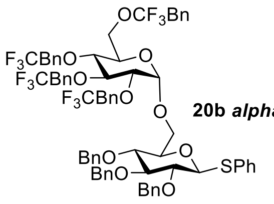

20b *alpha*

Data for *alpha* anomer 20b: ^{1}H NMR (500 MHz, $CDCl_3$) δ 7.57-7.16 (m, 36H), 5.16 (d, *J* = 3.5 Hz, 1H), 4.98 (d, *J* = 4.2 Hz, 1H), 4.94-4.89 (m, 2H), 4.86 (d, *J* = 10.9 Hz, 1H), 4.84-4.76 (m, 3H), 4.72-4.61 (m, 5H), 4.58-4.51 (m, 2H), 4.48 (d, *J* = 12.7 Hz, 1H), 3.97 (t, *J* = 9.2 Hz, 1H), 3.93-3.79 (m, 3H), 3.74-3.55 (m, 6H), 3.47 (m, 1H). 3.09 (t, *J* = 8.6Hz, 1H), **^{13}C NMR (125 MHz, $CDCl_3$)** δ 142.6, 142.2, 141.9, 138.3, 138.2, 137.9, 136.9, 134.2, 131.6, 130.3-129.4 (m), 129.0, 128.5, 128.4, 128.4, 128.0, 127.9, 127.7, 127.7, 127.7, 127.4, 127.3, 127.2, 127.1, 125.5-125.1 (m), 97.1, 88.2, 86.6, 81.8, 81.2, 80.4, 78.8, 77.8, 75.8, 75.4, 74.9, 74.6, 73.9, 72.6, 71.3, 70.1, 68.8, 66.1, **^{19}F NMR** (471 MHz, $CDCl_3$) δ -62.94 (s, 3F), -63.09 (s, 3F), -63.11 (s, 3F), -63.15 (s, 3F). **HRMS (ESI) m/z:** [M+Na]+ Calcd. for $C_{71}H_{64,}F_{12}O_{10}SNa$ 1359.3921; found 1359.3868. **$[\alpha]_D^{25}$ =** -49.5 (*c* 0.5, CH_2Cl_2). **IR (cm^{-1})** 3032, 2922, 2185, 1741, 1620, 1326, 1261, 1162, 1122, 1066, 1018, 821 (*p*-disubstituted benzene), 745, 698, 643.

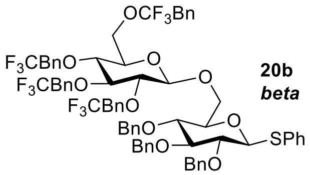

20b *beta*

Data for *beta* anomer 20b: ^{1}H NMR (500 MHz, $CDCl_3$) *δ* 7.64-7.19 (m, 36H), 5.02 (d, *J* = 12.3 Hz, 1H), 4.96-4.90 (m, 3H), 4.87-4.54 (m, 11H), 4.45 (d, *J* = 7.8 Hz, 1H), 4.21 (d, *J* = 11.0 Hz, 1H), 3.83-3.42 (m, 11H). **^{13}C NMR (125 MHz, $CDCl_3$)** δ 142.4, 142.2, 142.1, 142.0, 138.2, 137.9, 134.1, 131.1, 130.3-129.5 (m), 128.9, 128.5, 128.5, 128.4, 128.4, 128.2, 128.0, 128.0, 127.9, 127.9, 127.8, 127.8, 127.8, 127.7, 127.6, 127.6, 127.3, 127.3, 127.2, 125.4-125.1 (m), 123.0, 123.0, 103.7, 87.3, 86.7, 84.6, 82.1, 80.9, 78.7, 78.1, 78.0, 75.8, 75.5, 74.9, 74.7, 74.6, 74.0, 73.7,

72.8, 69.1, 68.9; **^{19}F NMR** (471 MHz, $CDCl_3$) δ -63.03 (s, 6F), -63.14 (s, 3F), -63.16 (s, 3F). **HRMS (ESI) m/z:** [M+Na]+ Calcd. for $C_{71}H_{64}F_{12}O_{10}SNa$ 1359.3926; found 1359.3930. **$[\alpha]_D^{25}$ =** -61.6 (*c* 1, CH_2Cl_2). **IR (cm^{-1})** 3033, 2869, 1621, 1326, 1210, 1162, 1122, 1066, 1019, 823 (*p*-disubstituted benzene), 739, 698.

Glycosylation using acceptor 21 and donor 11b

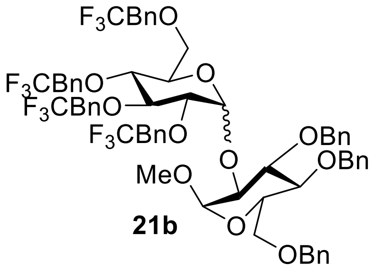

21b

Following the general procedure **A**, 2.0 ml of dichloromethane was used, 148.2 mg (0.1506 mmol) of 2,3,4,6-tetra-*O*-(4-trifluoromethylbenzyl)-D-glucopyranose *N*-phenyl trifluoroacetimidate donor (**11b**), 49.2 mg (0.106 mmol) of acceptor **21,** 252.3 mg of triphenylphosphine oxide (0.9066 mmol, 6.02 equiv.) and 22.5 µL (0.158 mmol) of TMSI (1.05 equiv.) were used. Purified by flash column chromatography (44-48% DE/hexanes (where DE = 3:1 DCM/EtOAc)) to give 73.6 mg of a colorless oil **21b**, yield 55%, *alpha*:*beta* ratio 19:1. ^{1}H NMR spectra of both anomeric products matched the previously published spectra.[6]

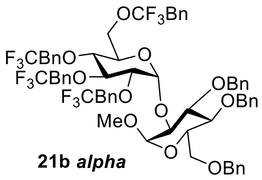

21b *alpha*

Data for *alpha* anomer 21b: ^{1}H NMR (400 MHz, $CDCl_3$) δ 7.57-7.07 (m, 31H), 5.09 (d, *J* = 3.6 Hz, 1H), 4.98-4.95 (m, 2H), 4.91 (d, *J* = 10.7 Hz, 1H), 4.84-4.69 (m, 6H), 4.61 (m, 2H), 4.50 (m, 3H), 4.39 (d, *J* = 12.7 Hz, 1H), 4.08-4.01 (m, 3H), 3.89 (dd, *J* = 9.8 Hz, 3.4 Hz, 1H), 3.80-3.74 (m, 2H), 3.70-3.64 (m, 3H), 3.59 (dd, *J* = 9.5 Hz, 3.6 Hz, 1H), 3.50 (d, *J* = 2.4 Hz, 2H), 3.41 (s, 3H);

^{13}C NMR (125 MHz, $CDCl_3$) δ 142.5, 142.3, 142.1, 141.9, 138.3, 138.1, 137.8, 130.3-129.6 (m), 128.4, 128.4, 128.3, 128.3, 128.0, 127.9, 127.8, 127.8, 127.8, 127.7, 127.7, 127.6, 127.5, 127.1, 125.4-125.1 (m), 123.0, 123.0, 123.0, 96.3, 93.4, 82.0, 80.7, 79.5, 77.9, 77.6, 75.9, 75.1, 74.7, 74.6, 73.9, 73.6, 72.6, 71.5, 70.3, 70.0, 68.4, 54.9; **^{19}F NMR** (471 MHz, $CDCl_3$) δ -63.12 (s, 3F), -63.15 (s, 3F), -63.17 (s, 3F), - 63.18 (s, 3F) **HRMS (ESI) m/z:** [M+K]+ Calcd. for $C_{66}H_{62,}F_{12}O_{11}K$ 1297.3738; found 1297.3714. **$[\alpha]_D^{25}$ =** +45.2° (*c* 1, CH_2Cl_2). **IR (cm^{-1})** 3032, 2914, 1324,1162, 1120,1064, 1018, 851, 822 (*p*-disubstituted benzene), 736, 699.

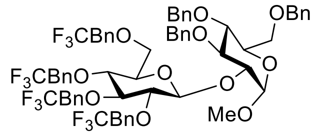

21b *beta*

Data for *beta* anomer 21b: ^{1}H NMR (400 MHz, $CDCl_3$) δ 7.58 (d, *J* = 8.0 Hz, 2H), 7.51-7.13 (m, 27H), 7.06 (m, 2H), 5.03 (d, *J* = 12.3 Hz, 1H), 4.95 (d, *J* = 3.5 Hz, 1H), 4.85 (d, *J* = 12.0 Hz, 1H), 4.78-4.44 (m, 13H), 4.02 (t, *J* = 9.8 Hz, 1H), 3.85 (dd, *J* = 9.8 Hz, 3.5 Hz, 1H), 3.82-3.65 (m, 6H), 3.64-3.57 (m, 2H), 3.51 (t, *J* = 8.1 Hz, 1H), 3.44 (m, 1H), 3.40 (s, 3H); **^{13}C NMR (125 MHz, $CDCl_3$)** δ 142.3, 142.1, 141.9, 141.9, 138.4, 138.0, 137.8, 130.1-129.4 (m), 128.4, 128.4, 128.3, 128.0, 127.8, 127.8, 127.7, 127.7, 127.6, 127.5, 127.4, 127.3, 127.2, 125.4-125.0 (m), 123.0, 104.1, 99.7, 84.8, 82.0, 81.6, 78.9, 78.3, 77.9, 75.1, 75.0, 74.6, 74.5, 74.0, 73.7, 73.6, 72.8, 70.0, 69.1, 68.4, 55.1; **^{19}F NMR** (471 MHz, $CDCl_3$) δ - 63.13 (s, 3F), -63.17 (s, 3F), -63.22 (s, 3F), -63.24 (s, 3F) **HRMS (ESI) m/z**: [M+K]+ Calcd. for $C_{66}H_{62,}F_{12}O_{11}K$ 1297.3738; found 1297.3718. **$[\alpha]_D^{25}$ =** +27.2° (*c* 1, DCM). **IR (cm^{-1})** 3054, 2927, 1325, 1264, 1165, 1018, 895, 823 (*p*-disubstituted benzene), 734, 703.

Glycosylation using acceptor 22 and donor 11b

Following the general procedure **A**, 2.0 ml of dichloromethane was used, 148.2 mg (0.1506 mmol) of 2,3,4,6-tetra-*O*-(4-trifluoromethylbenzyl)-D-glucopyranose *N*-phenyl trifluoroacetimidate donor

(**11b**), 28.7 mg (0.110 mmol) of acceptor **22,** 251.0 mg of triphenylphosphine oxide (0.9020 mmol, 5.99 equiv.) and 22.5 µL (0.158 mmol) of TMSI (1.05 equiv.) were used. Purified by flash column chromatography (36-50% DE/hexanes (where DE = 3:1 DCM/EtOAc)) to give 87.2 mg of a colorless oil **22b**, yield 75%, *alpha*:*beta* ratio 24:1.

22b
alpha

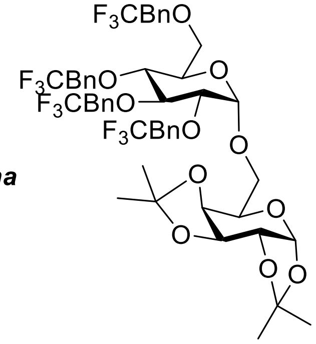

Data for *alpha* anomer 22b: **^{1}H NMR (400 MHz, $CDCl_3$) δ;** 7.59-7.19 (m, 16H), 5.54 (d, *J* = 5.0 Hz, 1H), 5.06 (d, *J* = 3.6 Hz, 1H), 4.98 (d, *J* = 12.1 Hz, 1H), 4.84-4.77 (m, 3H), 4.68 (s, 1H), 4.65 (s, 1H), 4.61 (dd, *J* = 7.9, 2.4 Hz, 1H), 4.57 (d, *J* = 12.0 Hz, 1H), 4.51 (d, *J* = 12.7 Hz, 1H), 4.34 (dd, *J* = 5.0, 2.5 Hz, 1H), 4.30 (dd, *J* = 7.9, 2.0 Hz, 1H), 4.06 (td, *J* = 6.7, 2.0 Hz, 1H), 4.00 (t, *J* = 9.2 Hz, 1H), 3.93-3.82 (m, 2H), 3.80-3.72 (m, 2H), 3.71-3.64 (m, 2H), 3.59 (dd, *J* = 9.5, 3.6 Hz, 1H), 1.54 (s, 3H), 1.45 (s, 3H), 1.34 (s, 3H), 1.28 (s, 3H); **^{13}C NMR (125 MHz, $CDCl_3$) δ** 142.7, 142.2, 142.0, 130.3-129.4 (m), 127.7, 127.6, 127.4, 127.2, 125.6-125.0 (m), 123.0, 109.4, 108.6, 96.4, 81.9, 80.1, 77.7, 74.5, 73.9, 72.7, 71.3, 71.0, 70.7, 70.6, 70.1, 68.7, 66.4, 65.7, 30.9, 26.1, 26.0, 24.9, 24.6. **^{19}F NMR (471 MHz, $CDCl_3$) δ** -63.08 (s, 3F), -63.09 (s, 6F), -63.15 (s, 3F). **HRMS (ESI) m/z:** [M+Na]+ Calcd. for $C_{50}H_{50}F_{12}O_{11}Na$ 1077.3054; found 1077.3060. **$[\alpha]_D^{25}$ =** +0.4° (*c* 1, CH_2Cl_2). **IR (cm^{-1})**, 2934, 1325, 1264, 1164, 1122, 1066, 1018, 896, 823 (*p*-disubstituted benzene), 735, 704.

Glycosylation using acceptor 23 and donor 11b

Following the general procedure **A**, 2.0 ml of dichloromethane was used, 147.9 mg (0.1503 mmol) of 2,3,4,6-tetra-*O*-(4-trifluoromethylbenzyl)-D-glucopyranose *N*-phenyl trifluoroacetimidate donor (**11b**), 17.1 mg (0.109 mmol) of acceptor **23,** 251.1 mg of triphenylphosphine oxide (0.9023 mmol, 6.00 equiv.) and 22.5 µL (0.158 mmol) of TMSI (1.05 equiv.) were used. Purified by flash column chromatography (24-32% DE/hexanes (Where DE= 3:1 DCM/EtOAc)) to give 103.6 mg of a colorless oil **23b**, yield >99 %, *alpha*:*beta* ratio 28:1.

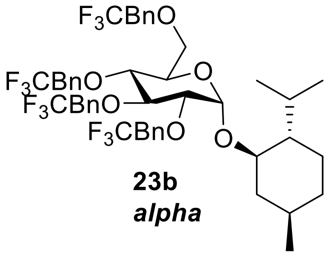

23b
alpha

Data for *alpha* anomer 23b: ^{1}H NMR (500 MHz, $CDCl_3$) δ; 7.59-7.19 (m, 16H), 5.08 (d, *J* = 3.6 Hz, 1H), 4.95 (d, *J* = 12.1 Hz, 1H), 4.86-4.77 (m, 2H), 4.73 (d, *J* = 12.3 Hz, 1H), 4.70-4.63 (m, 2H), 4.57-4.48 (m, 2H), 4.05-3.96 (m, 2H), 3.76 (dd, *J* = 10.6, 3.8 Hz, 1H), 3.68-3.60 (m, 2H), 3.53 (dd, *J* = 9.7, 3.6 Hz, 1H), 3.35 (td, *J* = 10.7, 4.4 Hz, 1H), 2.42-2.32 (m, 1H), 2.18-2.12 (m, 1H), 1.69-1.58 (m, 2H), 1.45-1.24 (m, 2H), 1.13-0.88 (m, 3H), 0.85 (d, *J* = 6.6 Hz, 3H), 0.83 (d, *J* = 7.0 Hz, 3H), 0.69 (d, *J* = 6.9 Hz, 3H). **^{13}C NMR (125 MHz, $CDCl_3$) δ;** 142.7, 142.1, 142.0, 130.4-129.4 (m), 127.7, 127.4, 127.3, 127.3, 125.5-125.1, 123.0, 98.4, 81.9, 81.9, 80.9, 78.3, 74.5, 74.0, 72.7, 72.1, 70.2, 69.1, 48.7, 43.1, 34.2, 31.7, 24.7, 23.0, 22.3, 21.0,16.1. **^{19}F NMR (471 MHz, $CDCl_3$) δ -**63.07 (s, 3F), -63.09 (s, 3F), -63.10 (s, 3F), -63.16 (s, 3F). **HRMS (ESI) m/z:** [M+Na]+ Calcd. for $C_{48}H_{50}F_{12}O_6Na$ 973.3308 found 973.3312. **$[\alpha]_D^{25}$ =** +3.0° (*c* 1, CH_2Cl_2). **IR (cm^{-1})**, 2925, 1324, 1162, 1122, 1066, 1018, 822 (*p*-disubstituted benzene), 593.

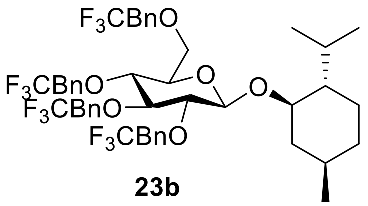

23b
beta

Data for *beta* anomer 23b: ^{1}H NMR (500 MHz, $CDCl_3$) δ; 7.62-7.20 (m, 16H), 4.99 (d, *J* = 12.0 Hz, 1H), 4.90 (d, *J* = 12.2 Hz, 1H), 4.83-4.75 (m, 2H), 4.72-4.63 (m, 3H), 4.58 (d, *J* = 12.8 Hz, 1H), 4.50 (d, *J* = 7.8 Hz, 1H), 3.79-3.74 (m, 1H), 3.70 (dd, *J* = 11.1, 1.9 Hz, 1H), 3.68-3.59 (m, 2H), 3.50 (td, *J* = 10.7, 4.2 Hz, 1H), 3.46-3.38 (m, 2H), 2.31 (m, 1H), 2.12-2.07 (m, 1H), 1.70-1.64 (m, 2H) 1.35 (m, 1H), 1.28-1.17 (m, 1H), 1.06-0.75 (m, 3H), 0.92 (d, *J* = 7.1 Hz, 3H), 0.89 (d, *J* = 6.7 Hz, 3H), 0.81 (d, *J* = 6.9 Hz, 3H) ; **^{13}C NMR (125 MHz, $CDCl_3$) δ;** 142.6, 142.5, 142.4, 142.1, 130.1-129.4 (m), 128.0, 127.5, 127.5, 127.2, 125.5-125.0 (m), 123.1, 100.5, 84.9, 82.2, 78.0, 77.8, 74.7, 74.5, 74.0, 73.7, 73.0, 69.4, 48.1, 40.8, 34.4, 31.5, 25.4, 23.3, 22.2, 21.0, 16.1; **^{19}F NMR (471 MHz, $CDCl_3$) δ -**63.05 (s, 3F), -63.08 (s, 3F), -63.11 (s, 3F), -63.14 (s, 3F) **HRMS (ESI) m/z:** [M+Na]+ Calcd. for $C_{48}H_{50}F_{12}O_6Na$ 973.3308; found 973.3309. **$[\alpha]_D^{25}$ =** -40.0° (*c* 1, CH_2Cl_2). **IR (cm^{-1})**, 2923, 1324, 1162, 1122, 1066, 1019, 822 (*p*-disubstituted benzene), 594.

Hydrogenolysis of 3,5-*bis*-trifluoromethylbenzyl groups

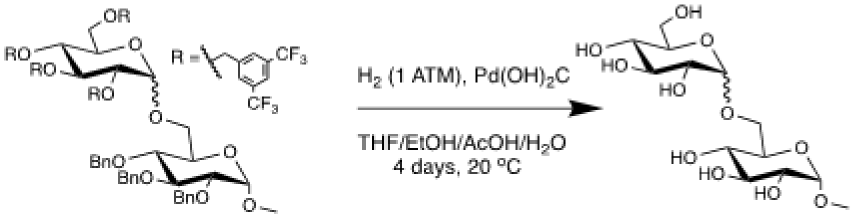

13c **24**

To a vial containing **13c** (40:1 α/β) (0.0538 mmol, 82.4 mg), was added tetrahydrofuran (1.5 mL), deionized water (0.50 mL), and ethanol (2.2 mL) at 20 °C. Then, 20% wt. $Pd(OH)_2$ on

carbon (176.5 mg) was added followed by the addition of acetic acid (0.50 mL). The reaction vessel was then capped with a septum and attached with a H_2-filled balloon through a needle. The reaction mixture was stirred at 20 ^{o}C for four days and replaced with a new H_2-filled balloon every 24 hours or when the balloon deflated. After four days, the balloon was removed and the reaction mixture was filtered through a cotton plug using methanol as a rinsing solvent. The resulting mixture was concentrated down by blowing nitrogen on the mixture for several hours until solvent evaporated. The crude concentrate was purified by flash column chromatography (1-10% MeOH/CH_2Cl_2) to give 9.9 mg (82%) of a colorless gum (methyl α-D-glucopyranosyl-(1→6)-α-D-glucopyranoside, **24**).

The ^{1}HNMR and ^{13}C NMR match the detailed spectral data reported in the literature reference 15.

1 mmol-Scale Preparation Procedure

To a flame-dried 100 mL round-bottom flask was added a stir bar, 2,3,4,6-tetra-*O*-3,5-bis-(trifluoromethyl)benzyl-D-glucopyranose glucosyl *N*-phenyltrifluoroacetimidate donor **11c** (1.79 g, 1.43 mmol, 1 equiv.), alcohol acceptor **12** (0.464 g, 1.00 mmol, 0.7 equiv.), and triphenylphosphine oxide (2.39 g, 8.59 mmol, 6 equiv.). The reaction vessel was capped with a rubber septum followed by the addition 19.0 mL of anhydrous dichloromethane using a syringe and subjected to vacuum-purge-backfill with N_2 three times. After all the contents dissolved, TMSI was added dropwise with a syringe at 18 ^{o}C. The reaction was allowed to stir for five days after which TLC indicated full consumption of the donor. The reaction mixture was diluted with 20 mL of dichloromethane and transferred to a separation funnel where it was washed vigorously with 80 mL of 1M NaOH. The organic layer was separated, dried over Na_2SO_4, filtered, and concentrated under vacuum in a rotary evaporator. A ^{1}H NMR of the crude mixture in $CDCl_3$ was taken before purification indicating an α:β ratio of 52:1 using the reducing-end methyl aglycone singlets. The product was purified through flash column chromatography using

a gradient of 0-15% of ethyl acetate in hexanes to give 1.40 g of **13c** methyl (2,3,4,6-tetra-O-3,5-bis-(trifluoromethyl)benzyl-D-glucopyranosyl)-(1→6)-2,3,4-tri-O-benzyl-D-glucopyranoside in a 91% yield with an α:β ratio of 48:1.

NMR Study Procedure (*in-situ* generation of glycosyl iodide a-25 from PTFAI donor 11b)

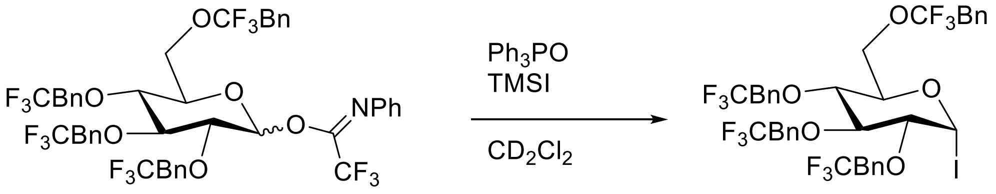

11b **α-25 (CF_3Bn)**

To 37.90 mg (0.0385 mmol) of 2,3,4,6-tetra-*O*-(4-trifluoromethylbenzyl)-D-glucopyranose glucosyl donor **11b** in an oven dried vial was added 63.4 mg (0.228 mmol) of triphenylphosphine oxide followed by addition of 0.6 ml CD_2Cl_2. The vial was capped with a septum then flushed with nitrogen gas for less than ten seconds. TMS-I (7.5 µL, 0.0527mmol) was then added dropwise and the contents were immediately transferred to an NMR tube using a syringe. ^{1}H NMR data of the resulting mixture were acquired after 3 min., 38 min., and 165 min. from the time TMS-I was added.

References

1.) Mandal, S. S.; Ganesh, N. V.; Sadowska, J. M.; Bundle, D. R. *Organic & Biomolecular Chemistry* **2017**, *15* (18), 3874.
2.) Nigudkar, S.S.; Stine, K.J.; Demchenko, A.V. *Journal of the American Chemical Society* **2014**, *136* (3), 921.
3.) Dieskau, A. P.; Plietker, B. *Organic Letters* **2011**, *13* (20), 5544.

4.) Kitowski, A.; Jiménez-Moreno, E.; Salvadó, M.; Mestre, J.; Castillón, S.; Jiménez-Osés, G.; Boutureira, O.; Bernardes, G. J. *Organic Letters* **2017**, *19* (19), 5490.

5.) Wang, Z.; Li, T.; Zhao, J.; Shi, X.; Jiao, D.; Zheng, H.; Chen, C.; Zhu, B. *Organic Letters* **2018**, 20 (21), 6640.

6.) Njeri, D. K.; Pertuit, C. J.; Ragains, J. R. *Organic & Biomolecular Chemistry* **2020**, 18 (13), 2405.

7.) König, S. G.; Öz, S.; Krämer, R. *Chemical Communications* **2015**, *51* (34), 7360.

8.) Wolf, N.; Kersting, L.; Herok, C.; Mihm, C.; Seibel, J. *The Journal of Organic Chemistry* **2020**, *85* (15), 9751.

9.) Xia, M.-jie; Yao, W.; Meng, X.-bao; Lou, Q.-hua; Li, Z.-jun. *Tetrahedron Letters* **2017**, *58* (24), 2389.

10.) Lecourt, T.; Herault, A.; Pearce, A. J.; Sollogoub, M.; Sinaÿ, P. *Chemistry - A European Journal* **2004**, *10* (12), 2960.

11.) Serra, F.; Coutrot, P.; Estève-Quelquejeu, M.; Herson, P.; Olszewski, T. K.; Grison, C. *European Journal of Organic Chemistry* **2011**, *2011* (10), 1841.

12.) Garcia, B. A.; Gin, D. Y. *Journal of the American Chemical Society* **2000**, *122* (18), 4269.

13.) Kim, H.-H.; Yang, H.; Khot, V.; Whitfield, D.; Boons, G.-J. *Eur. J. Org. Chem.* **2006**, 5007–5028.

14.) Kitowski, A.; Jiménez-Moreno, E.; Salvadó, M.; Mestre, J.; Castillón, S.; Jiménez-Osés, G.; Boutureira, O.; Bernardes, G. J. *Organic Letters* **2017**, *19* (19), 5490.

15.) Gouasmat, A.; Lemétais, A.; Solles, J.; Bourdreux, Y.; Beau, J.-M. *Eur. J. Org. Chem.* **2017**, 3355.

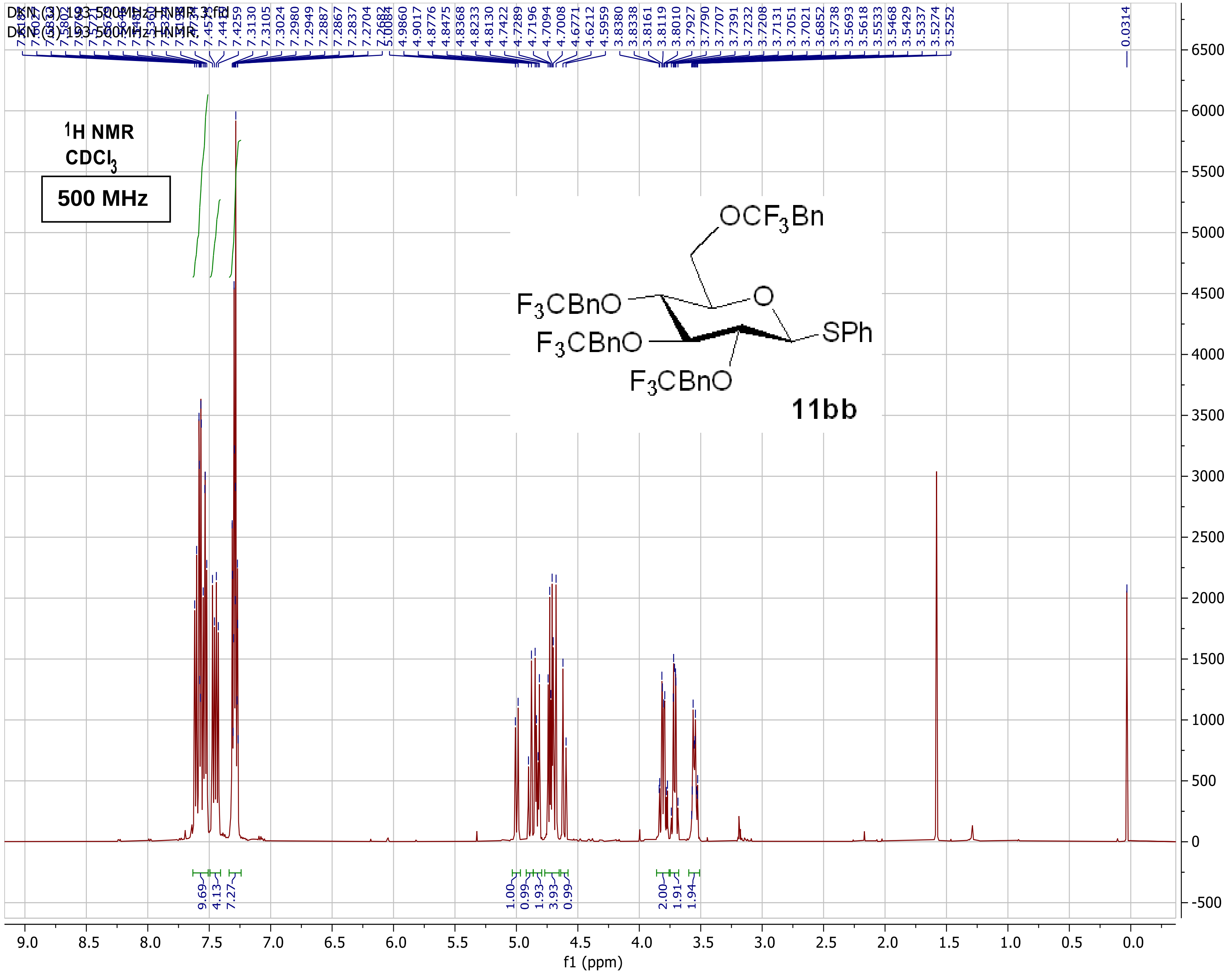

1H NMR
CDCl3
500 MHz
OCF3Bn
F3CBnO
F3CBnO
O
SPh
F3CBnO
11bb
9.69
4.13
7.27
1.00
0.99
1.93
3.93
0.99
2.00
1.91
1.94
f1 (ppm)

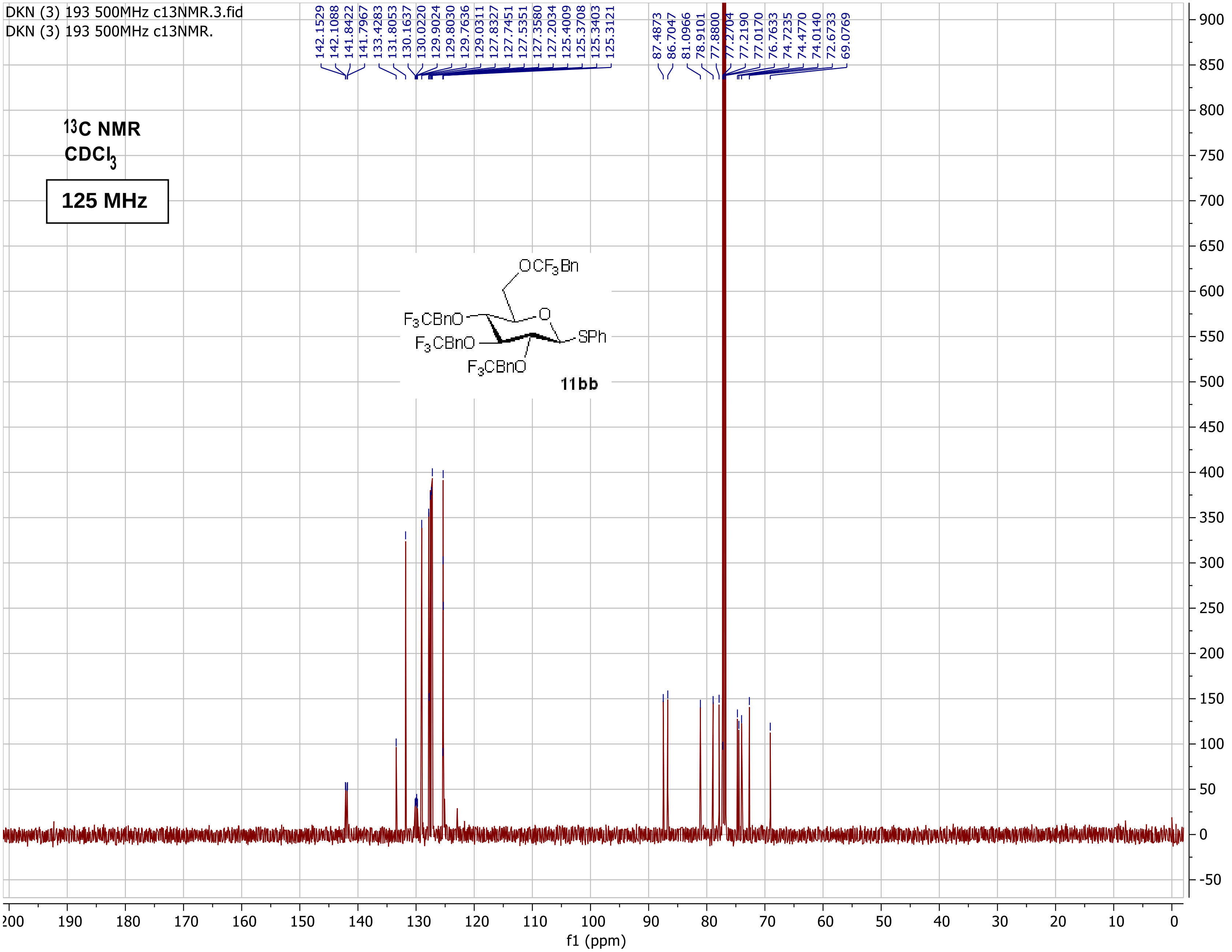
DKN (3) 193 500MHz c13NMR.3.fid
DKN (3) 193 500MHz c13NMR.
142.1529 142.1088 141.8422 141.7967 133.4283 131.8053 130.1637 130.0220 129.9024 129.8030 129.7636 129.0311 127.8327 127.7451 127.5351 127.3580 127.2034 125.4009 125.3708 125.3403 125.3121
87.4873 86.7047 81.0966 78.9101 77.8800 77.2704 77.2190 77.0170 76.7633 74.7235 74.4770 74.0140 72.6733 69.0769
13C NMR
CDCl3
125 MHz
OCF3Bn
F3CBnO
O
F3CBnO
SPh
F3CBnO
11bb
f1 (ppm)

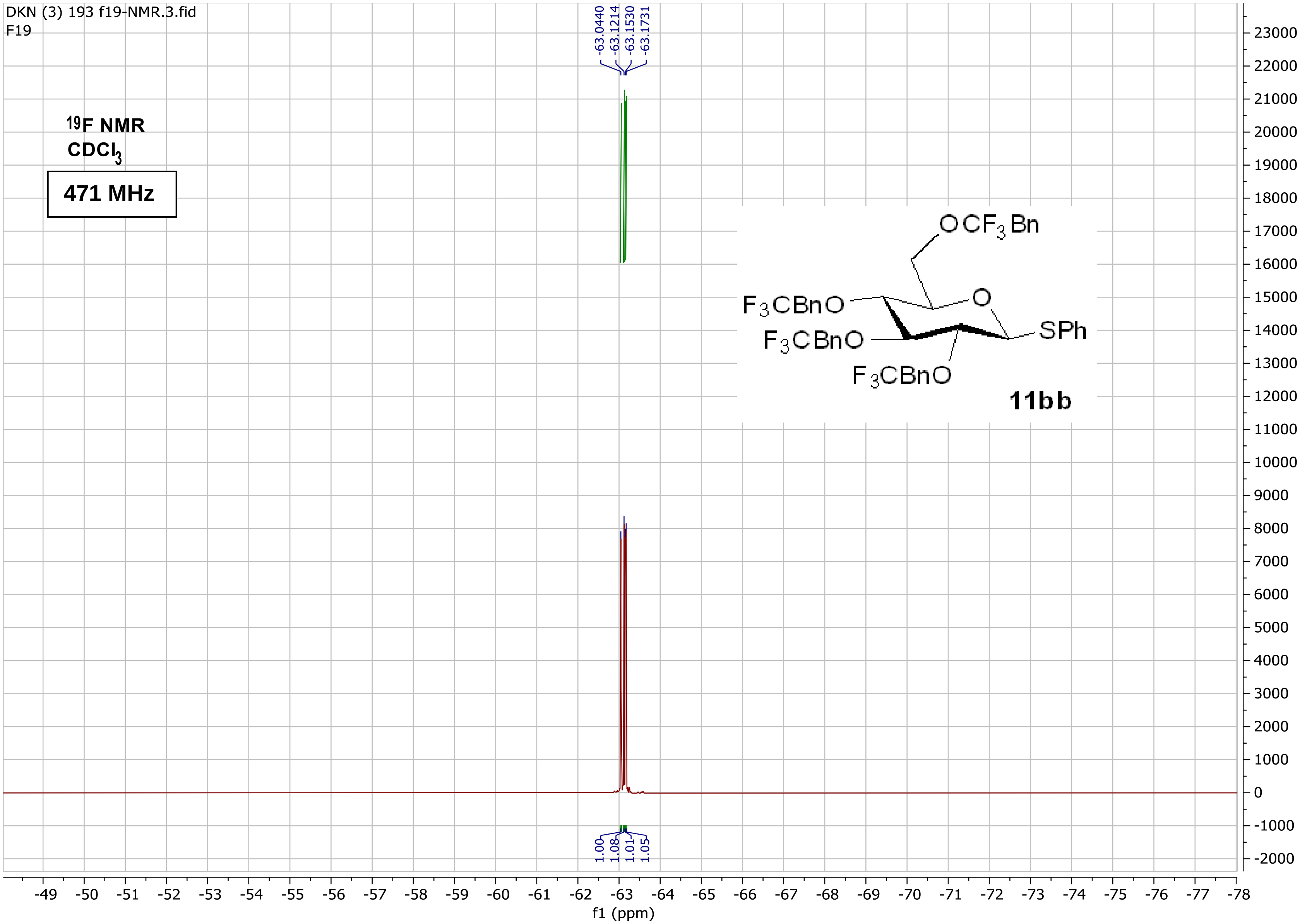

DKN (3) 193 f19-NMR.3.fid
F19
-63.0440
-63.1214
-63.1530
-63.1731
19F NMR
CDCl3
471 MHz
OCF3Bn
F3CBnO
O
SPh
F3CBnO
F3CBnO
11bb
1.00
1.08
1.01
1.05
f1 (ppm)

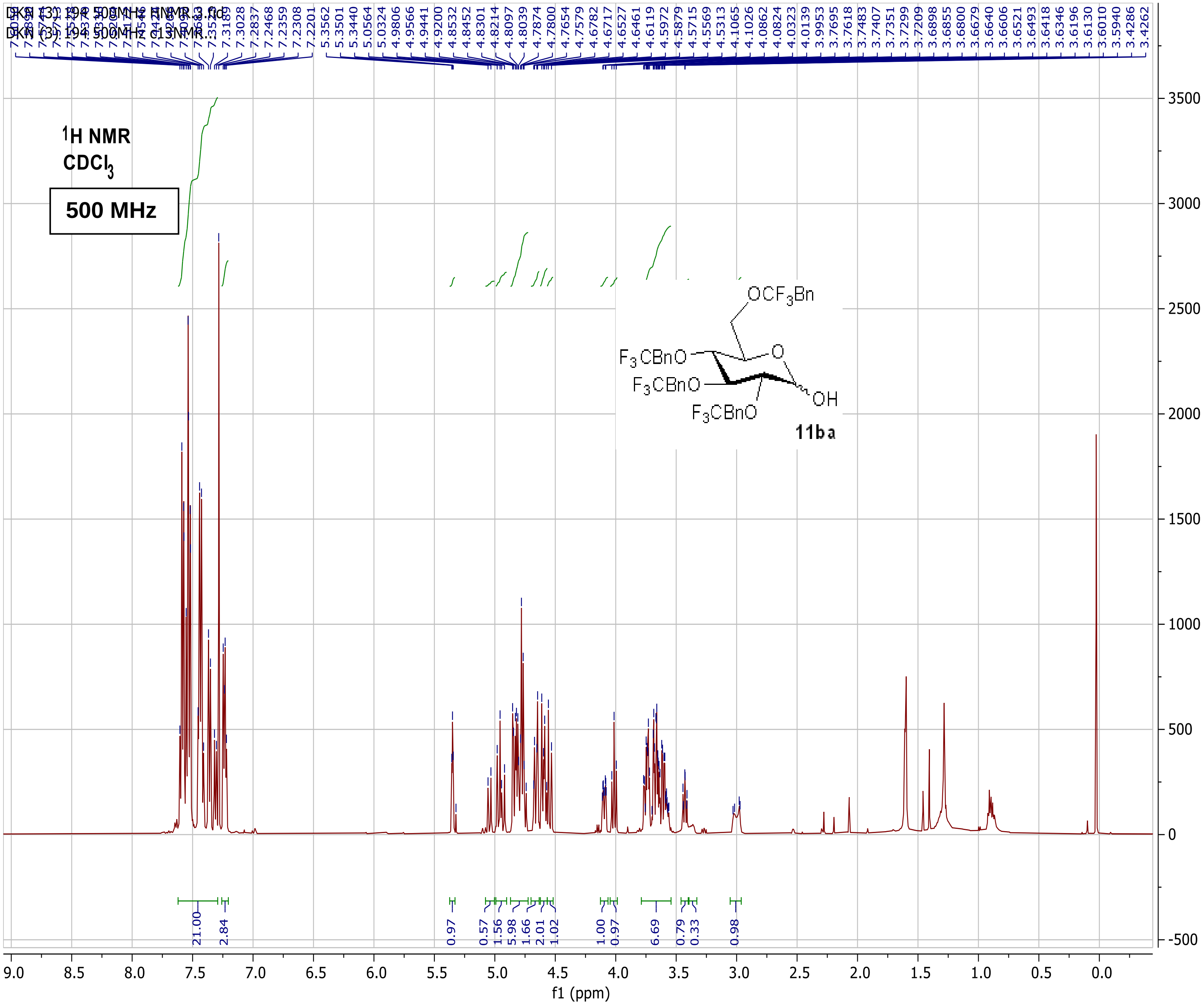

¹H NMR
CDCl3
500 MHz
OCF3Bn
F3CBnO
F3CBnO
F3CBnO
O
OH
11ba
f1 (ppm)

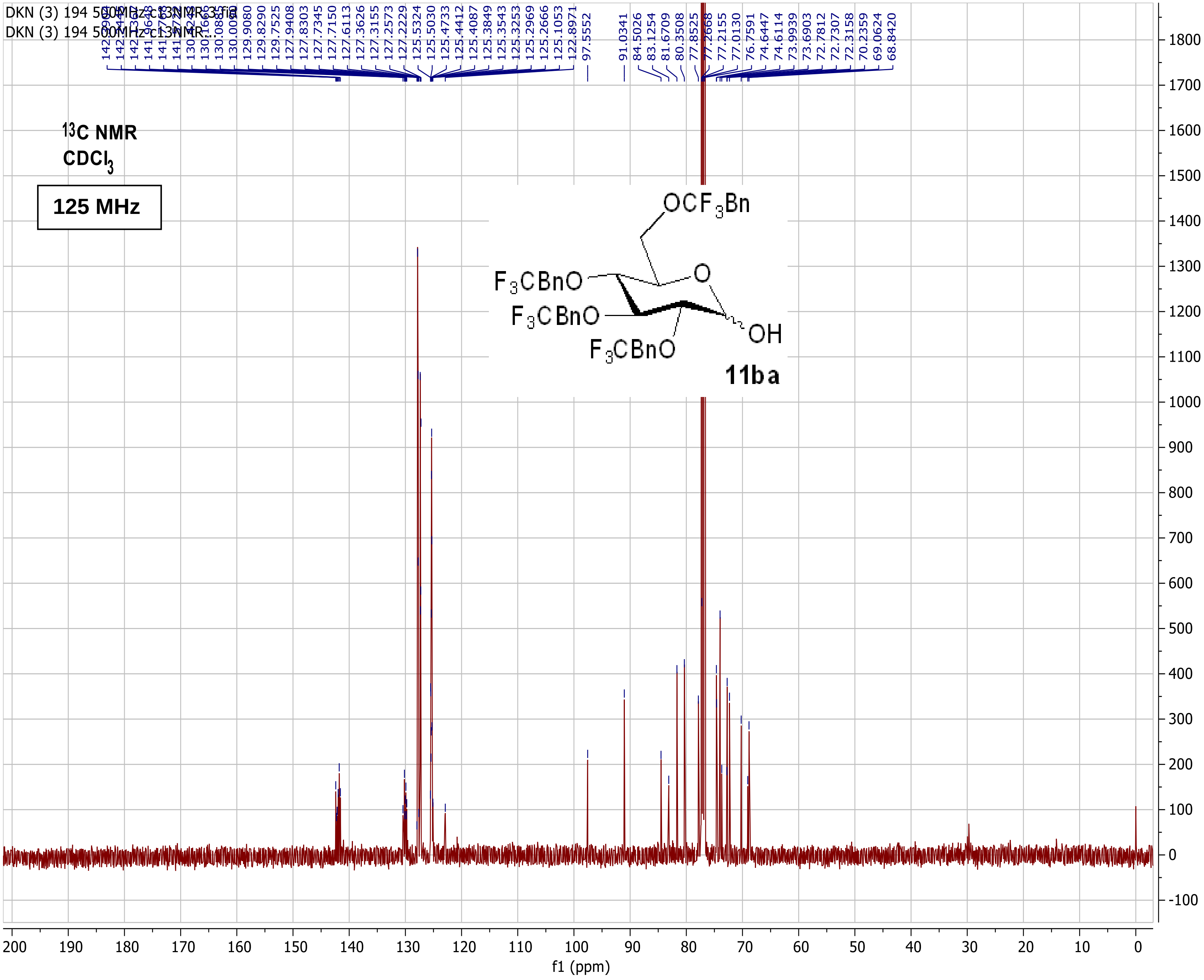

DKN (3) 194 500MHz-c13NMR
13C NMR
CDCl3
125 MHz
OCF3Bn
F3CBnO
F3CBnO
F3CBnO
O
OH
11ba
91.0341
84.5026
83.1254
81.6709
80.3508
77.8525
77.2155
77.0130
76.7591
74.6447
74.6114
73.9939
73.6903
72.7812
72.7307
72.3158
70.2359
69.0624
68.8420
97.5552
122.8971
125.1053
125.2666
125.2969
125.3253
125.3543
125.3849
125.4087
125.4412
125.4733
125.5030
125.5324
127.2229
127.2573
127.3155
127.3626
127.6113
127.7150
127.7345
127.8303
127.9408
129.7525
129.8290
129.9080
f1 (ppm)

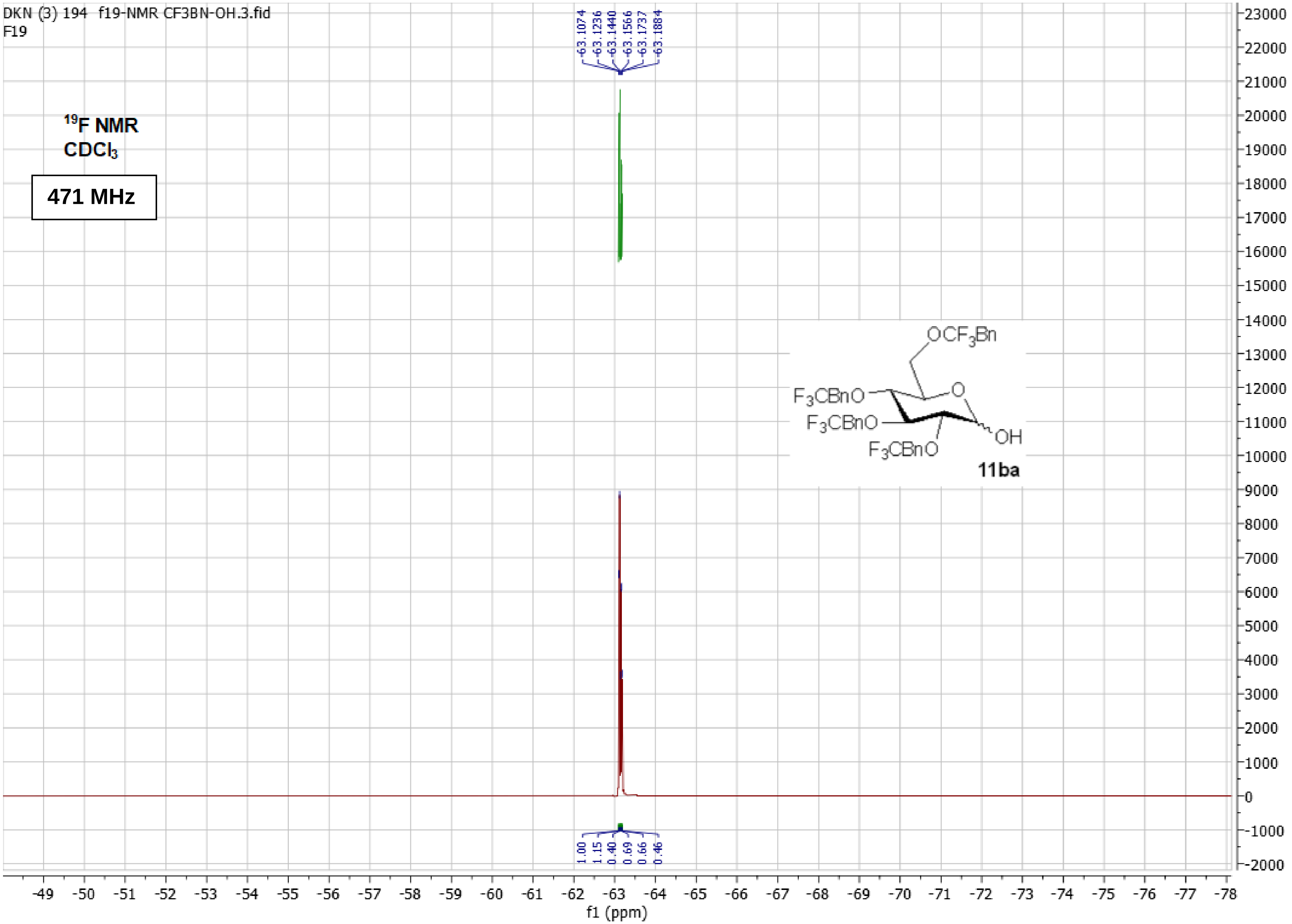
DKN (3) 194 f19-NMR CF3BN-OH.3.fid
F19
19F NMR
CDCl3
471 MHz
-63.1074
-63.1236
-63.1440
-63.1566
-63.1737
-63.1884
OCF3Bn
F3CBnO
F3CBnO
F3CBnO
O
OH
11ba
1.00
1.15
0.40
0.69
0.66
0.46
f1 (ppm)

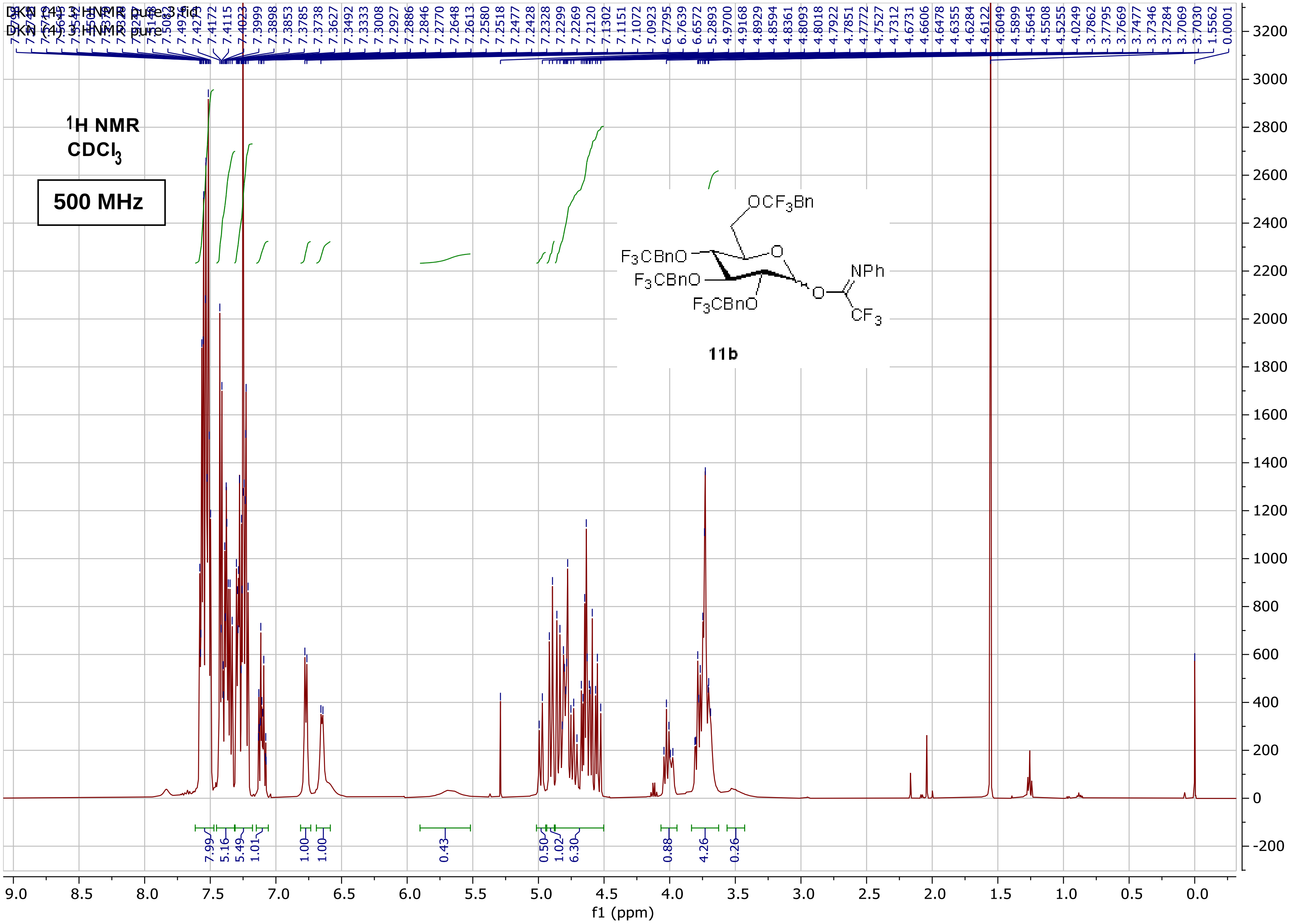

1H NMR
CDCl3
500 MHz
OCF3Bn
F3CBnO
F3CBnO
F3CBnO
O
NPh
CF3
11b
f1 (ppm)

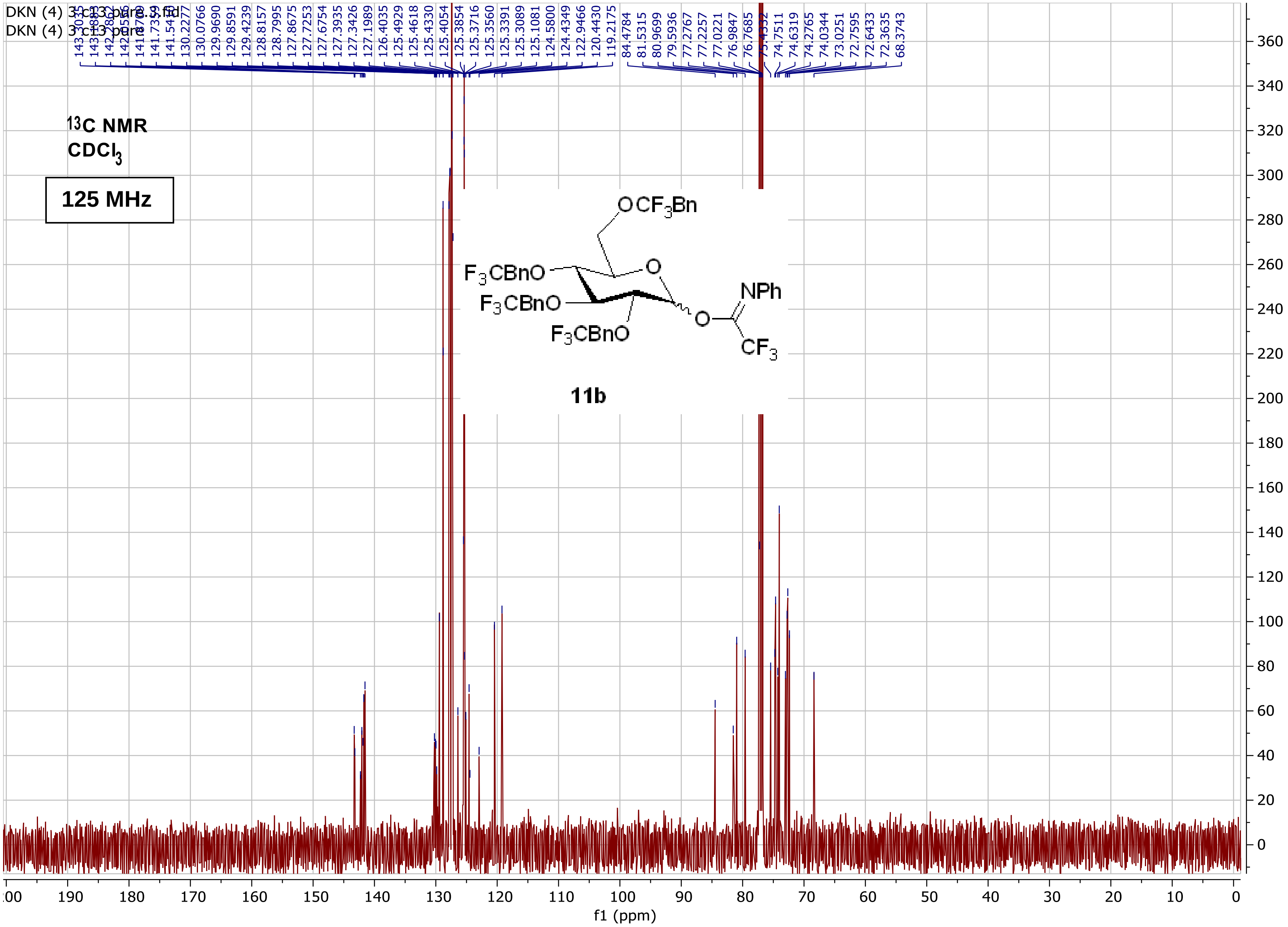
13C NMR
CDCl3
125 MHz
11b
OCF3Bn
F3CBnO
F3CBnO
F3CBnO
O
NPh
CF3
130.2277
130.0766
129.9690
129.8591
129.4239
128.8157
128.7995
127.8675
127.7253
127.6754
127.3935
127.3426
127.1989
126.4035
125.4929
125.4618
125.4330
125.4054
125.3854
125.3716
125.3560
125.3391
125.3089
125.1081
124.5800
124.4349
122.9466
120.4430
119.2175
84.4784
81.5315
80.9699
79.5936
77.2767
77.2257
77.0221
76.9847
76.7685
74.7511
74.6319
74.2765
74.0344
73.0251
72.7595
72.6433
72.3635
68.3743
f1 (ppm)

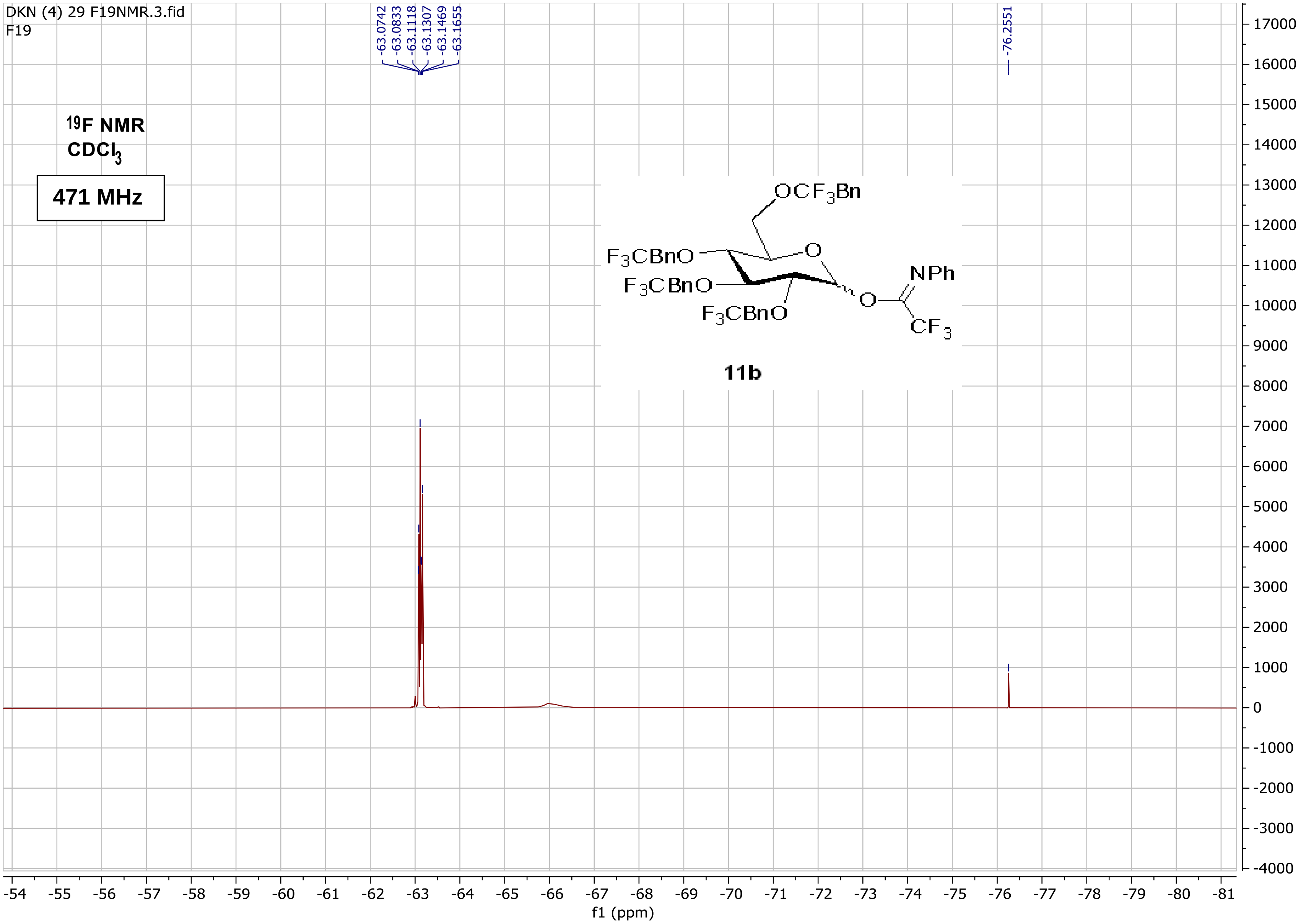

DKN (4) 29 F19NMR.3.fid
F19
-63.0742
-63.0833
-63.1118
-63.1307
-63.1469
-63.1655
-76.2551
19F NMR
CDCl3
471 MHz
OCF3Bn
F3CBnO
F3CBnO
F3CBnO
O
NPh
O
CF3
11b
f1 (ppm)

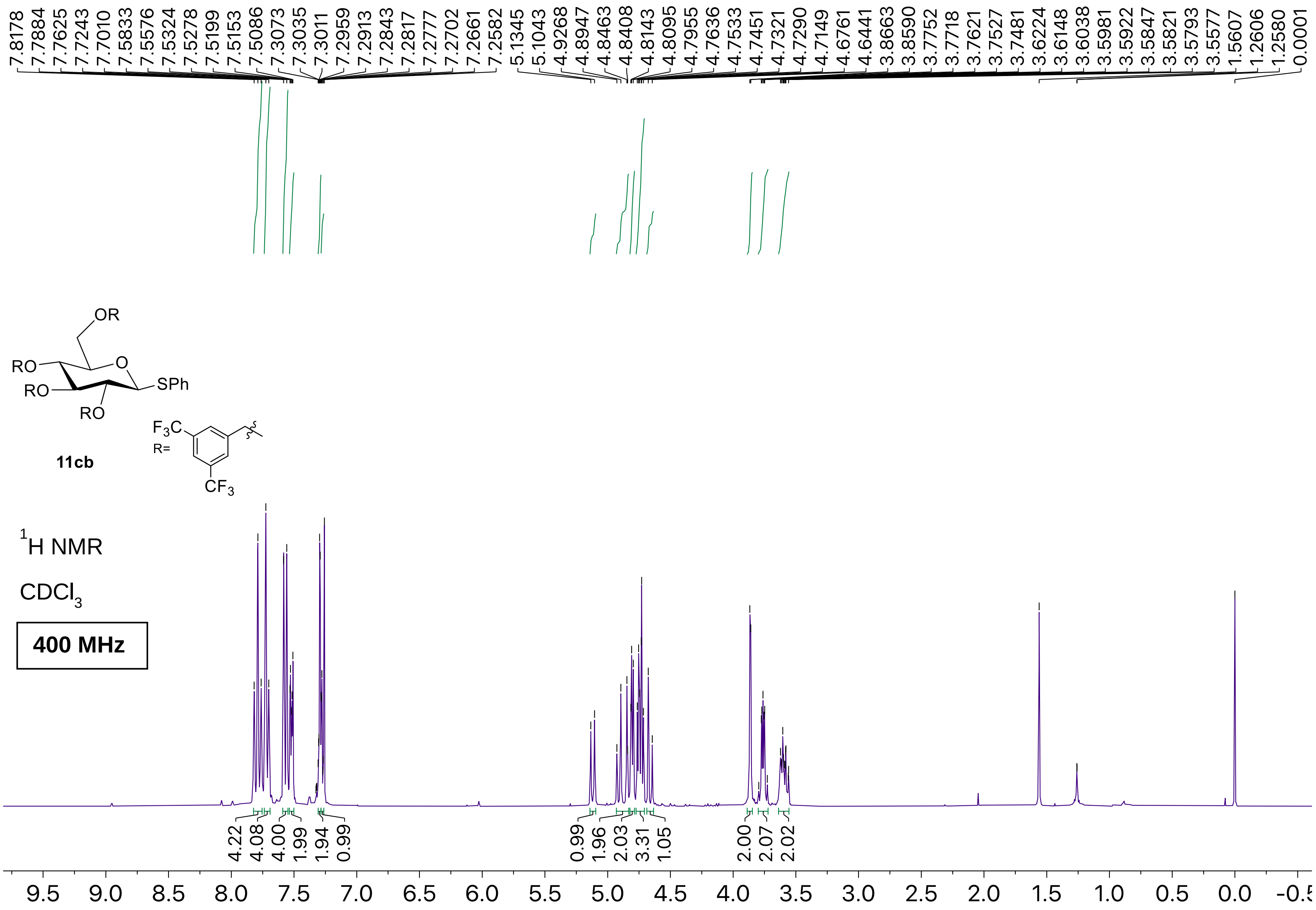

7.8178 7.7884 7.7625 7.7243 7.7010 7.5833 7.5576 7.5324 7.5278 7.5199 7.5153 7.5086 7.3073 7.3035 7.3011 7.2959 7.2913 7.2843 7.2817 7.2777 7.2702 7.2661 7.2582 5.1345 5.1043 4.9268 4.8947 4.8463 4.8408 4.8143 4.8095 4.7955 4.7636 4.7533 4.7451 4.7321 4.7290 4.7149 4.6761 4.6441 3.8663 3.8590 3.7752 3.7718 3.7621 3.7527 3.7481 3.6224 3.6148 3.6038 3.5981 3.5922 3.5847 3.5821 3.5793 3.5577 1.5607 1.2606 1.2580 0.0001
OR
RO
O
RO
SPh
RO
F3C
R=
CF3
11cb
1H NMR
CDCl3
400 MHz
4.22 4.08 4.00 1.99 1.94 0.99
0.99 1.96 2.03 3.31 1.05
2.00 2.07 2.02
9.5 9.0 8.5 8.0 7.5 7.0 6.5 6.0 5.5 5.0 4.5 4.0 3.5 3.0 2.5 2.0 1.5 1.0 0.5 0.0 -0.5

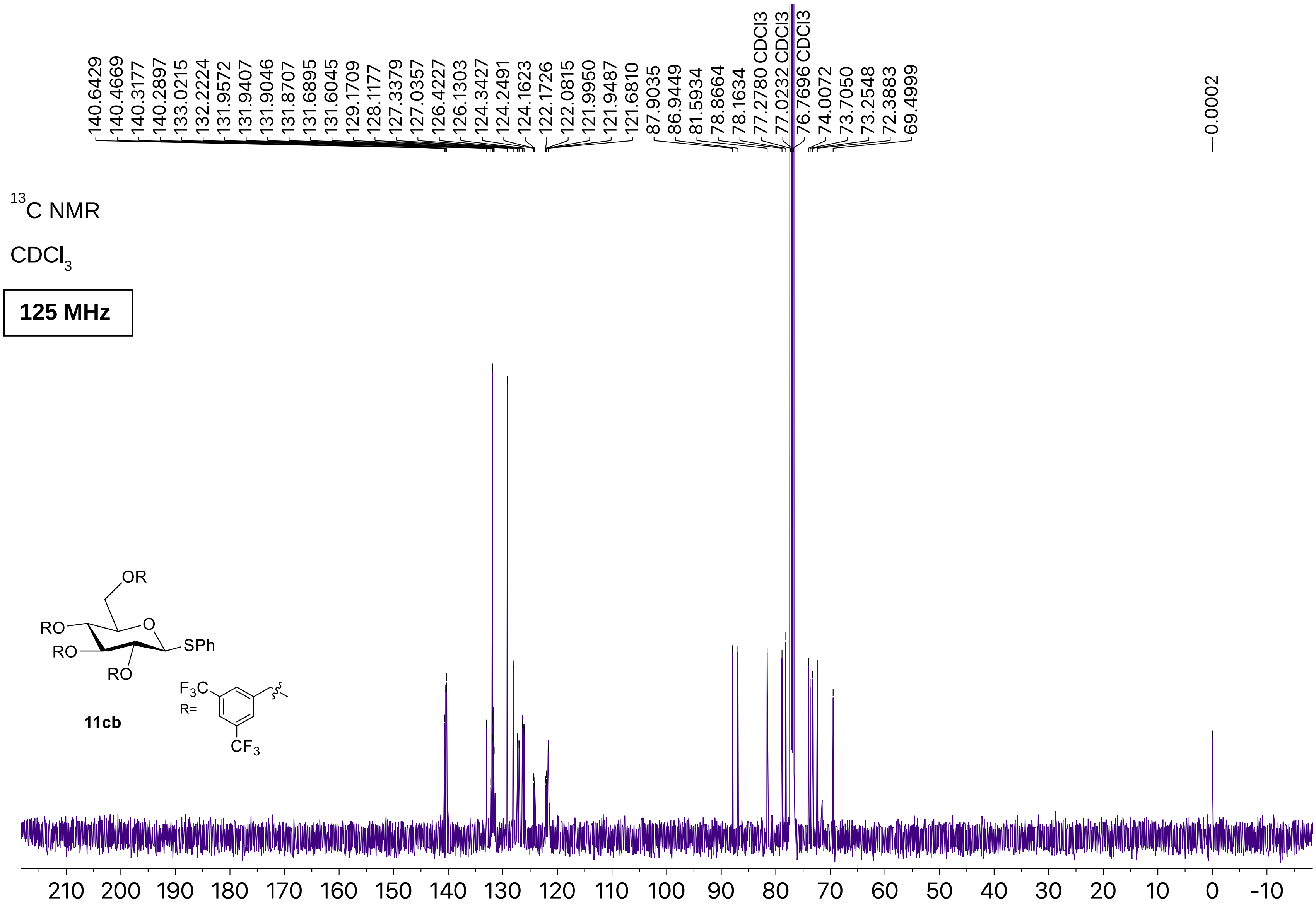

^{13}C NMR

$CDCl_3$

125 MHz

11cb

^{19}F NMR

$CDCl_3$

471 MHz

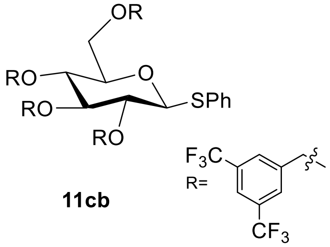

11cb

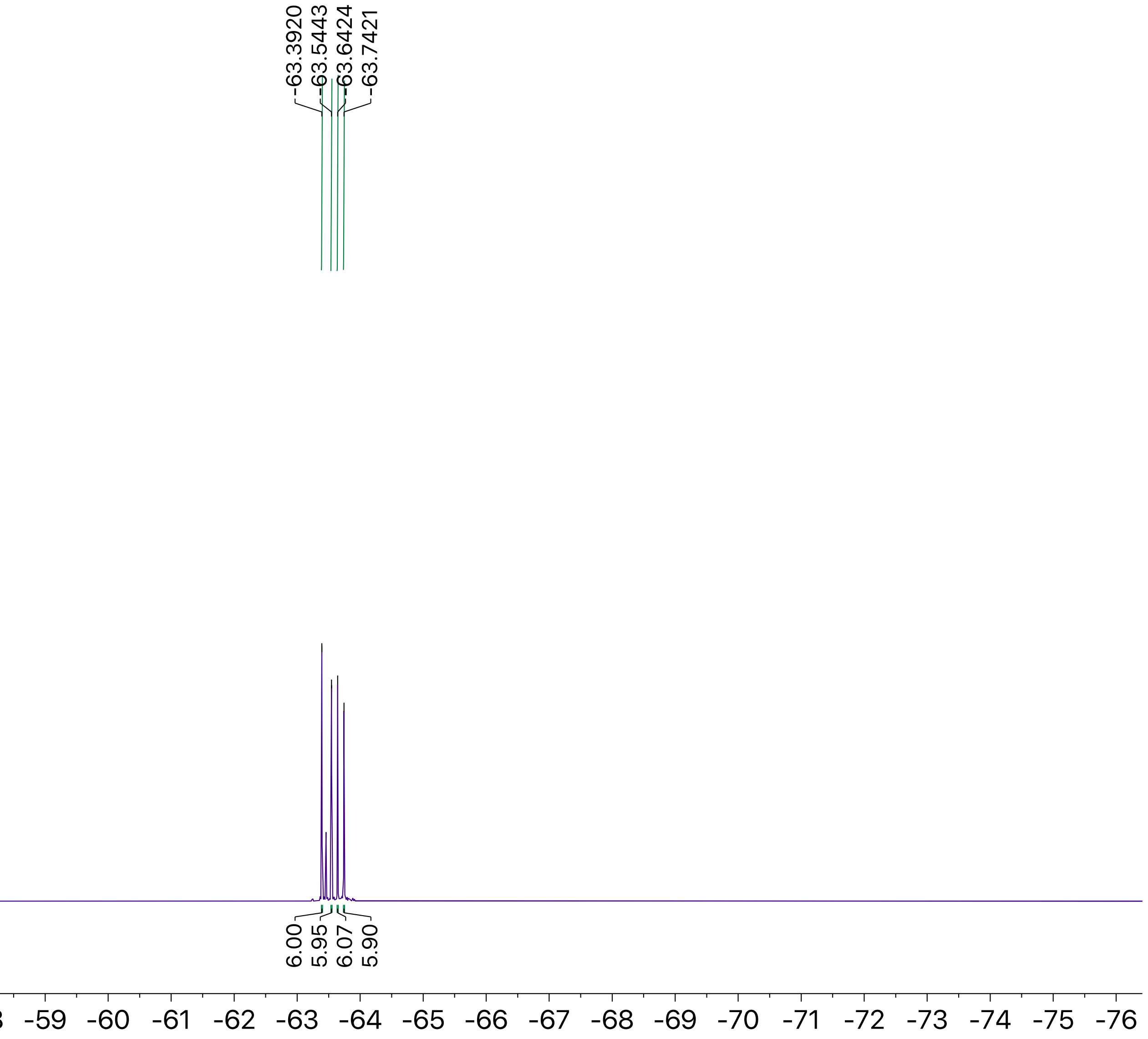

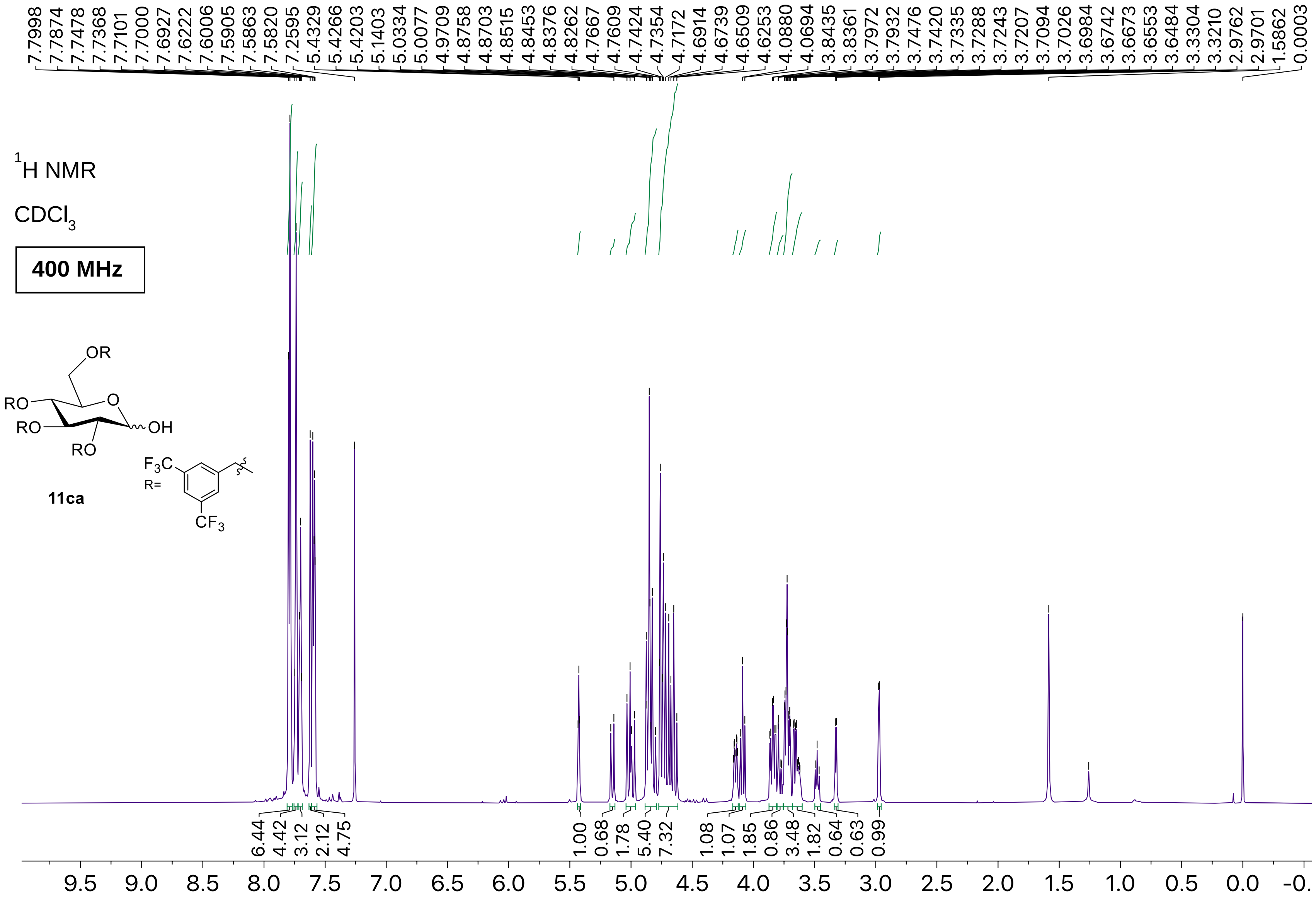
1H NMR
CDCl3
400 MHz
OR
RO
O
OH
RO
RO
F3C
R=
CF3
11ca

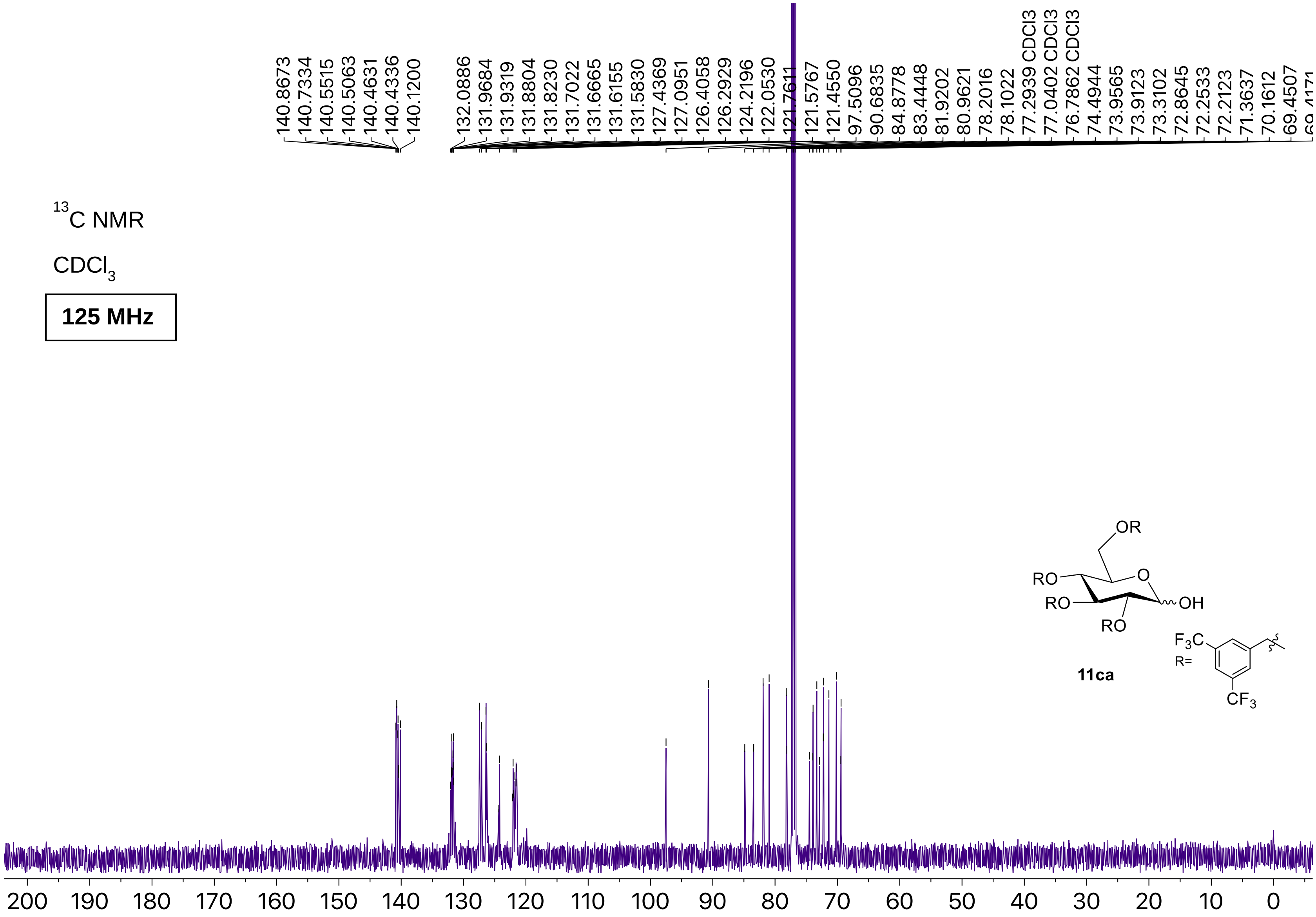

140.8673
140.7334
140.5515
140.5063
140.4631
140.4336
140.1200
132.0886
131.9684
131.9319
131.8804
131.8230
131.7022
131.6665
131.6155
131.5830
127.4369
127.0951
126.4058
126.2929
124.2196
122.0530
121.7611
121.5767
121.4550
97.5096
90.6835
84.8778
83.4448
81.9202
80.9621
78.2016
78.1022
77.2939 CDCl3
77.0402 CDCl3
76.7862 CDCl3
74.4944
73.9565
73.9123
73.3102
72.8645
72.2533
72.2123
71.3637
70.1612
69.4507
^{13}C NMR
$CDCl_3$
125 MHz
OR
O
RO
RO
OH
RO
F_3C
R=
CF_3
11ca
200
190
180
170
160
150
140
130
120
110
100
90
80
70
60
50
40
30
20
10
0

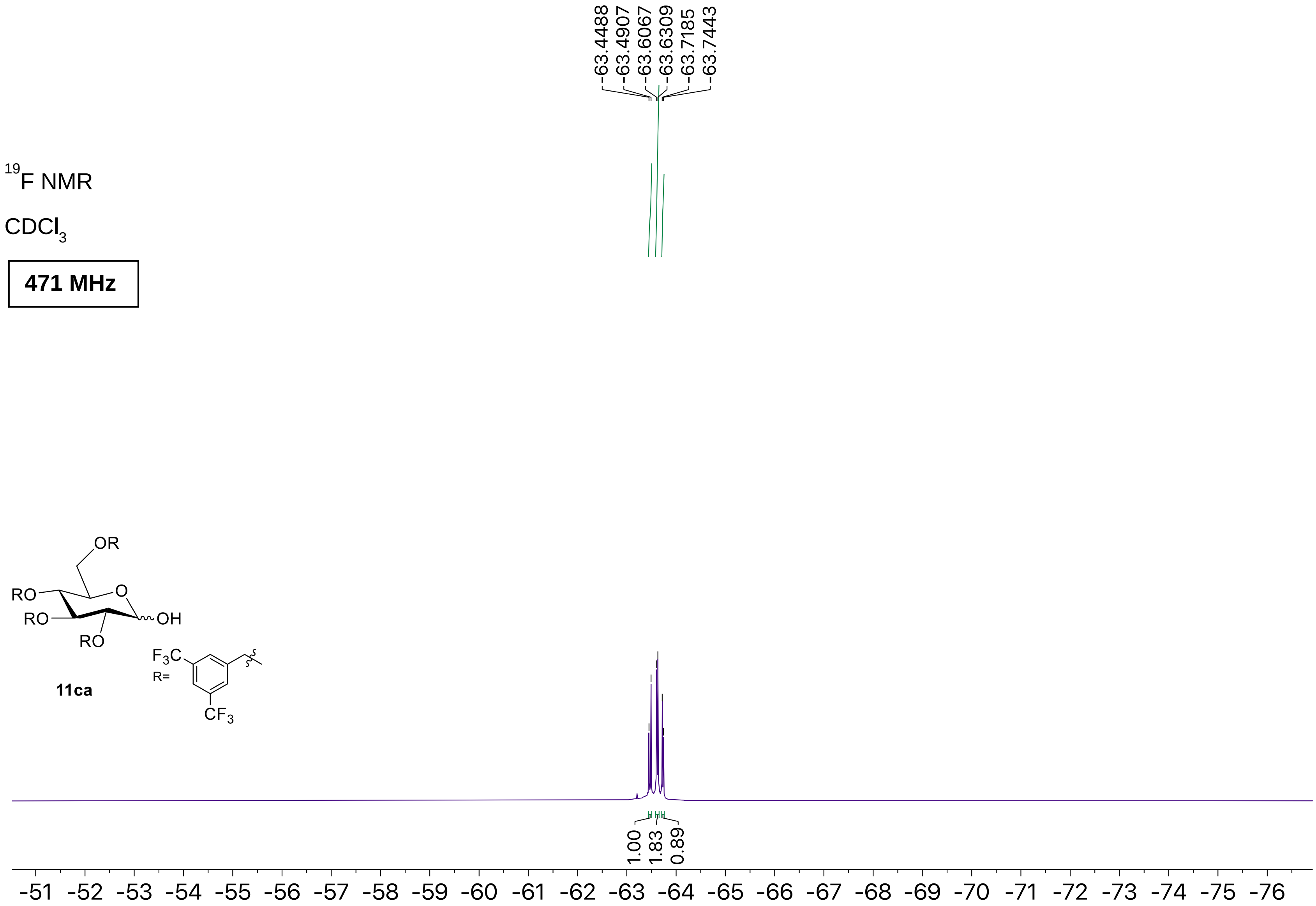

-63.4488
-63.4907
-63.6067
-63.6309
-63.7185
-63.7443
^{19}F NMR
CDCl$_3$
471 MHz
1.00
1.83
0.89
-51 -52 -53 -54 -55 -56 -57 -58 -59 -60 -61 -62 -63 -64 -65 -66 -67 -68 -69 -70 -71 -72 -73 -74 -75 -76
OR
RO
O
RO
OH
RO
F3C
R=
CF3

11ca

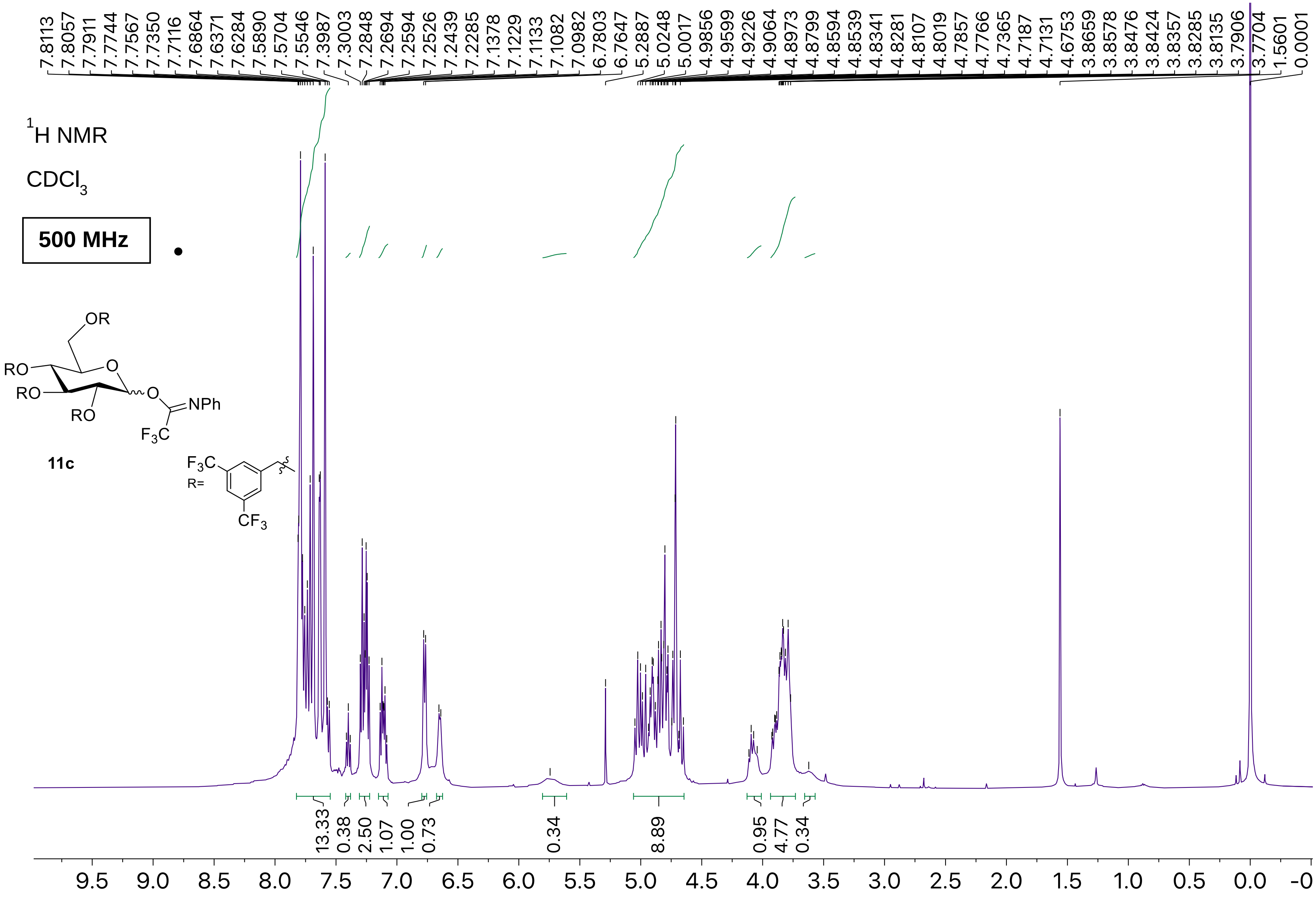

1H NMR
CDCl3
500 MHz
11c
R=
7.8113
7.8057
7.7911
7.7744
7.7567
7.7350
7.7116
7.6864
7.6371
7.6284
7.5890
7.5704
7.5546
7.3987
7.3003
7.2848
7.2694
7.2594
7.2526
7.2439
7.2285
7.1378
7.1229
7.1133
7.1082
7.0982
6.7803
6.7647
5.2887
5.0248
5.0017
4.9856
4.9599
4.9226
4.9064
4.8973
4.8799
4.8594
4.8539
4.8341
4.8281
4.8107
4.8019
4.7857
4.7766
4.7365
4.7187
4.7131
4.6753
3.8659
3.8578
3.8476
3.8424
3.8357
3.8285
3.8135
3.7906
3.7704
1.5601
0.0001
13.33
0.38
2.50
1.07
1.00
0.73
0.34
8.89
0.95
4.77
0.34
9.5
9.0
8.5
8.0
7.5
7.0
6.5
6.0
5.5
5.0
4.5
4.0
3.5
3.0
2.5
2.0
1.5
1.0
0.5
0.0
-0

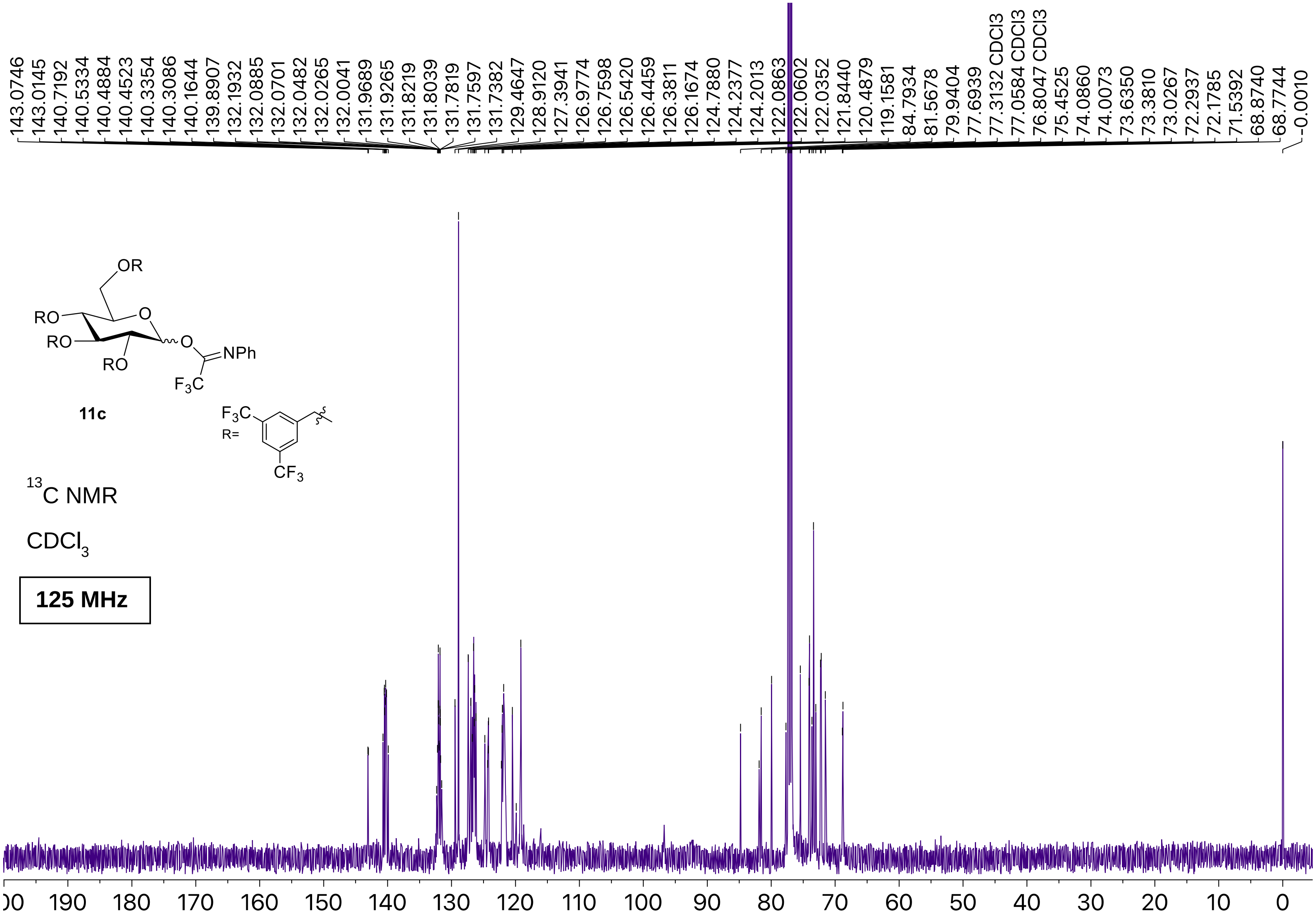
143.0746
143.0145
140.7192
140.5334
140.4884
140.4523
140.3354
140.3086
140.1644
139.8907
132.1932
132.0885
132.0701
132.0482
132.0265
132.0041
131.9689
131.9265
131.8219
131.8039
131.7819
131.7597
131.7382
129.4647
128.9120
127.3941
126.9774
126.7598
126.5420
126.4459
126.3811
126.1674
124.7880
124.2377
124.2013
122.0863
122.0602
122.0352
121.8440
120.4879
119.1581
84.7934
81.5678
79.9404
77.6939
77.3132 CDCl3
77.0584 CDCl3
76.8047 CDCl3
75.4525
74.0860
74.0073
73.6350
73.3810
73.0267
72.2937
72.1785
71.5392
68.8740
68.7744
-0.0010
OR
RO
O
RO
O
NPh
RO
F3C
11c
F3C
R=
CF3
13C NMR
CDCl3
125 MHz
00 190 180 170 160 150 140 130 120 110 100 90 80 70 60 50 40 30 20 10 0

^{19}F NMR

$CDCl_3$

471 MHz

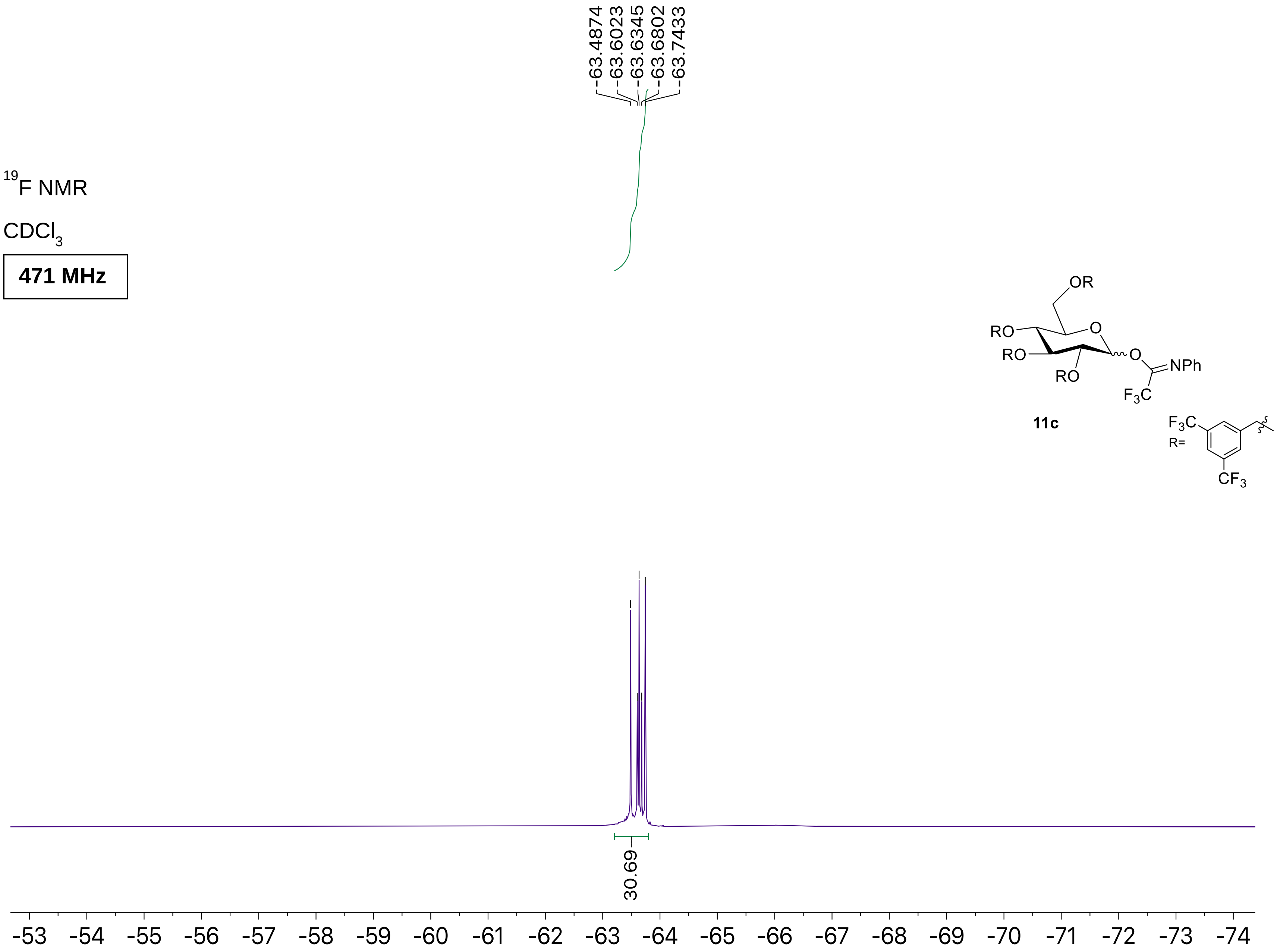

11c

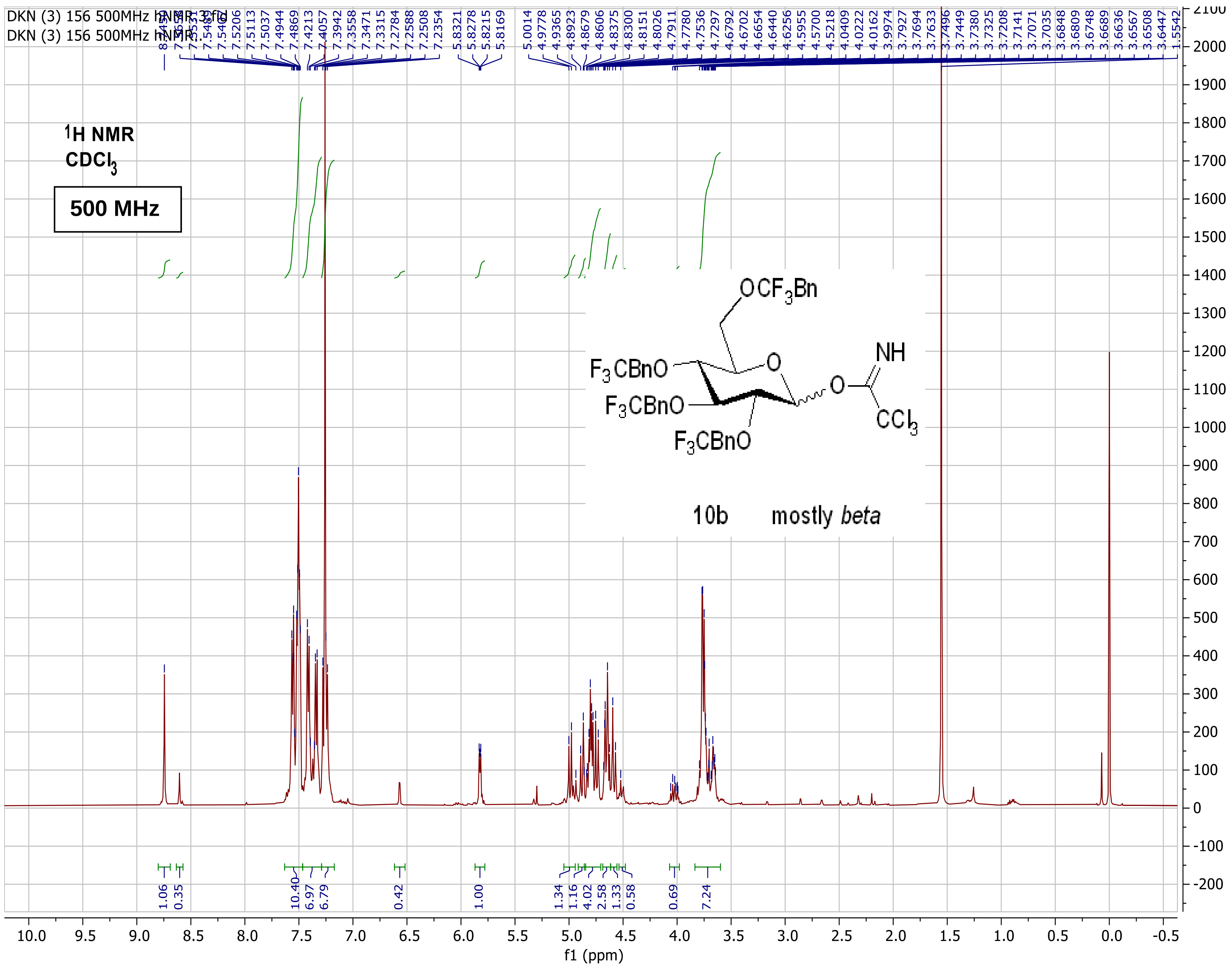

DKN (3) 156 500MHz hNMR 3.fid
1H NMR
CDCl3
500 MHz
OCF3Bn
F3CBnO
F3CBnO
F3CBnO
O
NH
CCl3
10b mostly beta
f1 (ppm)

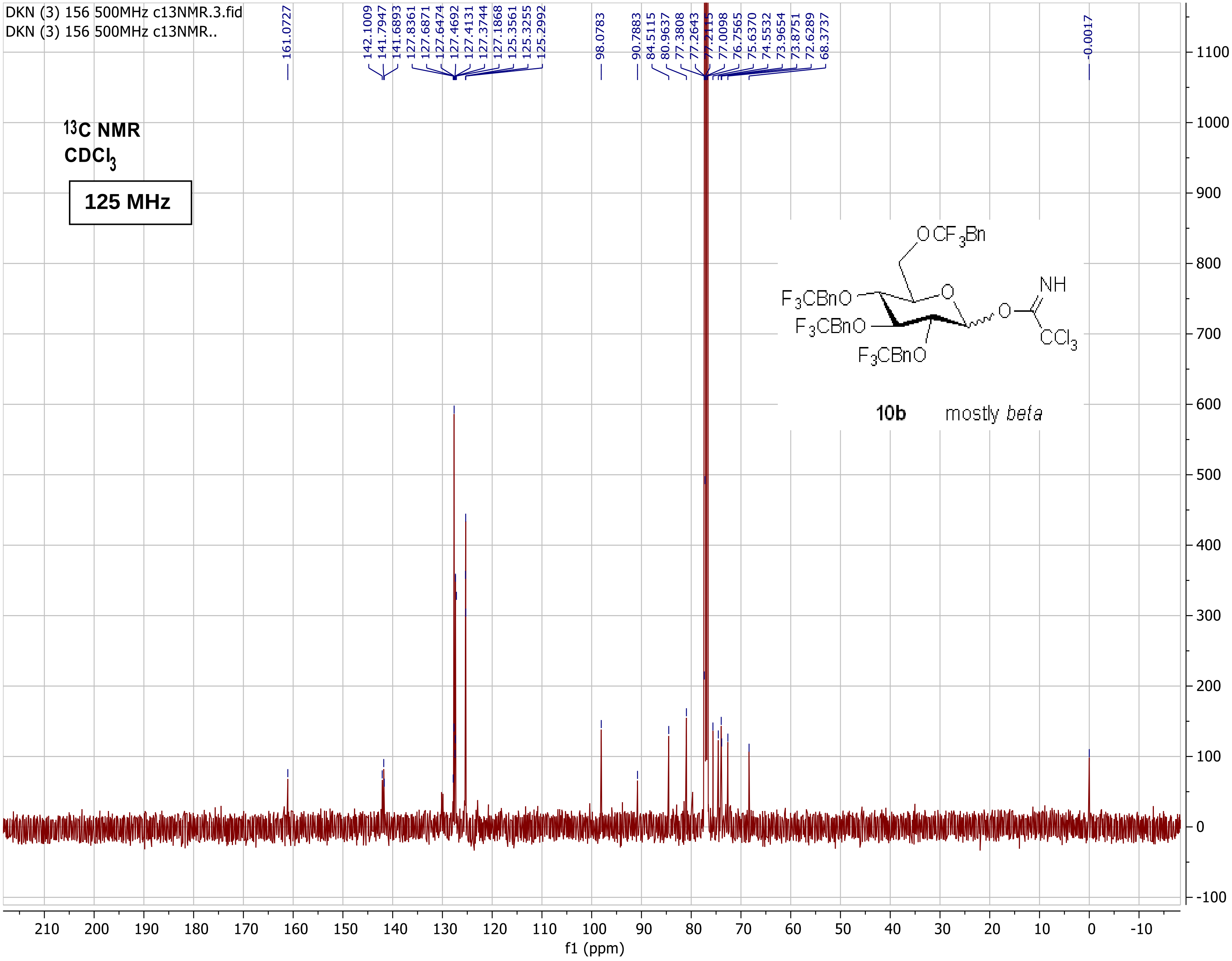
DKN (3) 156 500MHz c13NMR.3.fid
DKN (3) 156 500MHz c13NMR..
161.0727
142.1009
141.7947
141.6893
127.8361
127.6871
127.6474
127.4692
127.4131
127.3744
127.1868
125.3561
125.3255
125.2992
98.0783
90.7883
84.5115
80.9637
77.3808
77.2643
77.2115
77.0098
76.7565
75.6370
74.5532
73.9654
73.8751
72.6289
68.3737
-0.0017
13C NMR
CDCl3
125 MHz
OCF3Bn
F3CBnO
F3CBnO
F3CBnO
O
O
NH
CCl3
10b
mostly beta
f1 (ppm)

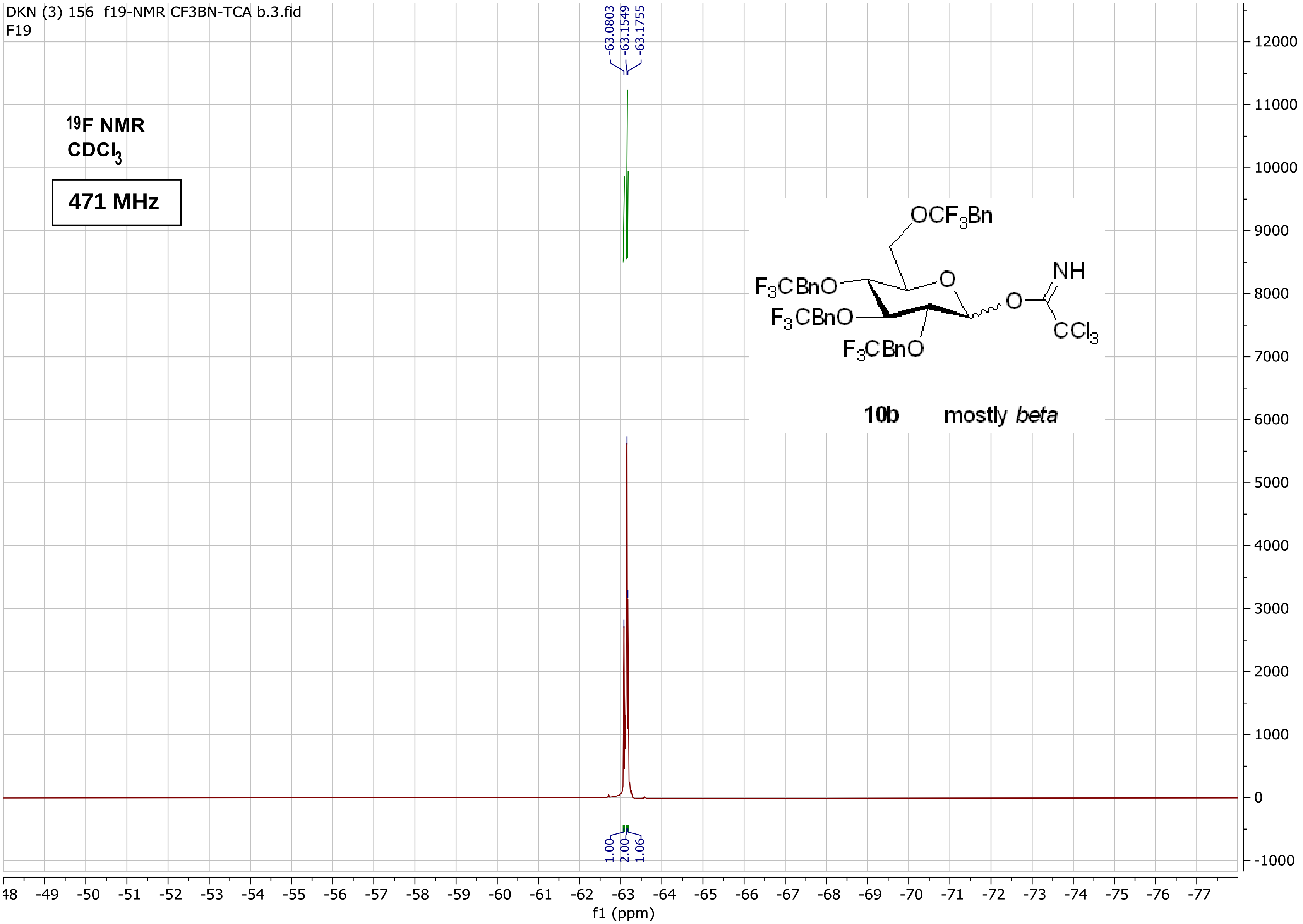
DKN (3) 156 f19-NMR CF3BN-TCA b.3.fid
F19
19F NMR
CDCl3
471 MHz
-63.0803
-63.1549
-63.1755
OCF3Bn
F3CBnO
F3CBnO
F3CBnO
O
NH
O
CCl3
10b mostly beta
1.00
2.00
1.06
f1 (ppm)

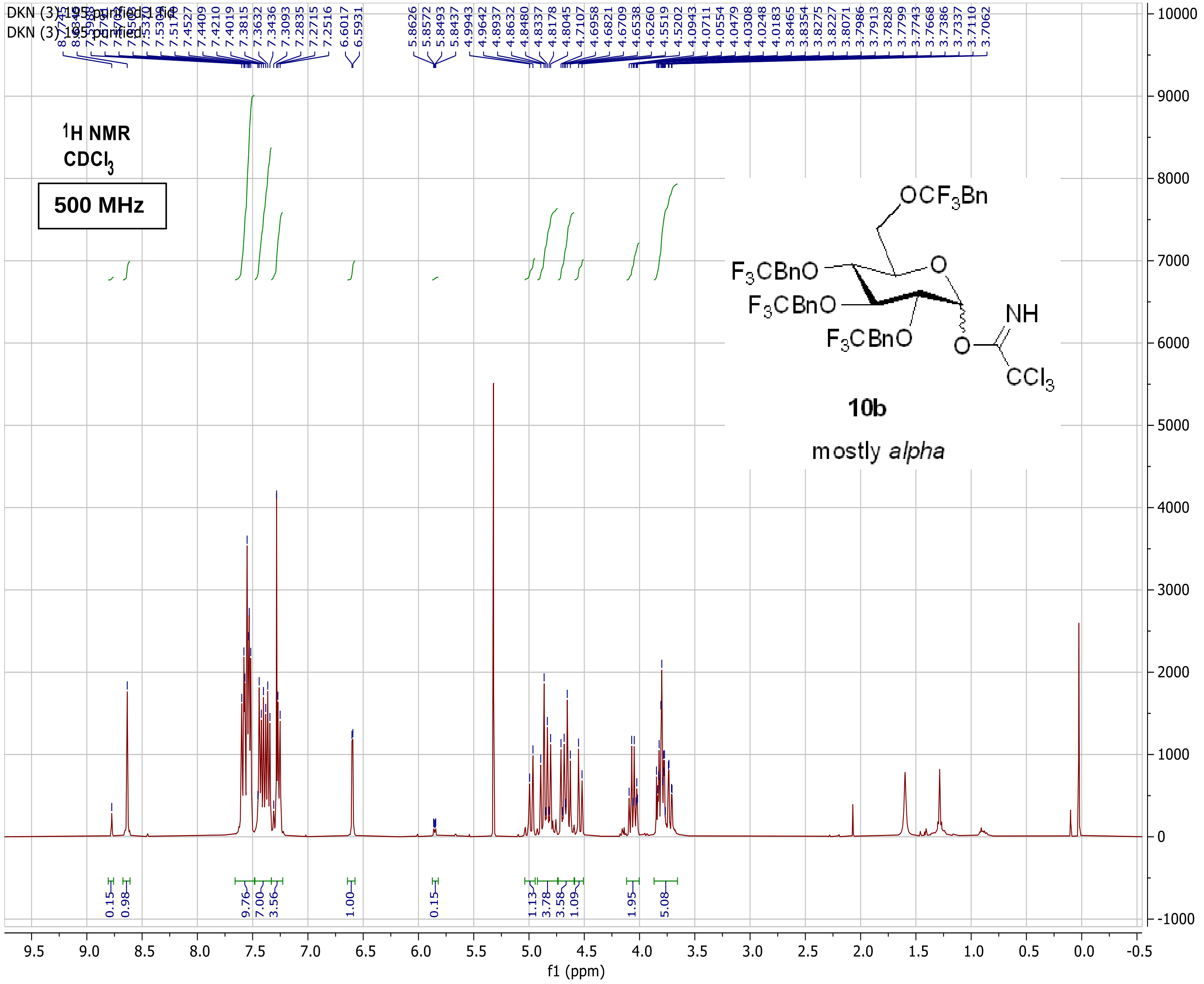

DKN (3) 195 purified.1.fid
DKN (3) 195 purified
1H NMR
CDCl3
500 MHz
OCF3Bn
F3CBnO
F3CBnO
F3CBnO
O
NH
O
CCl3
10b
mostly alpha
f1 (ppm)

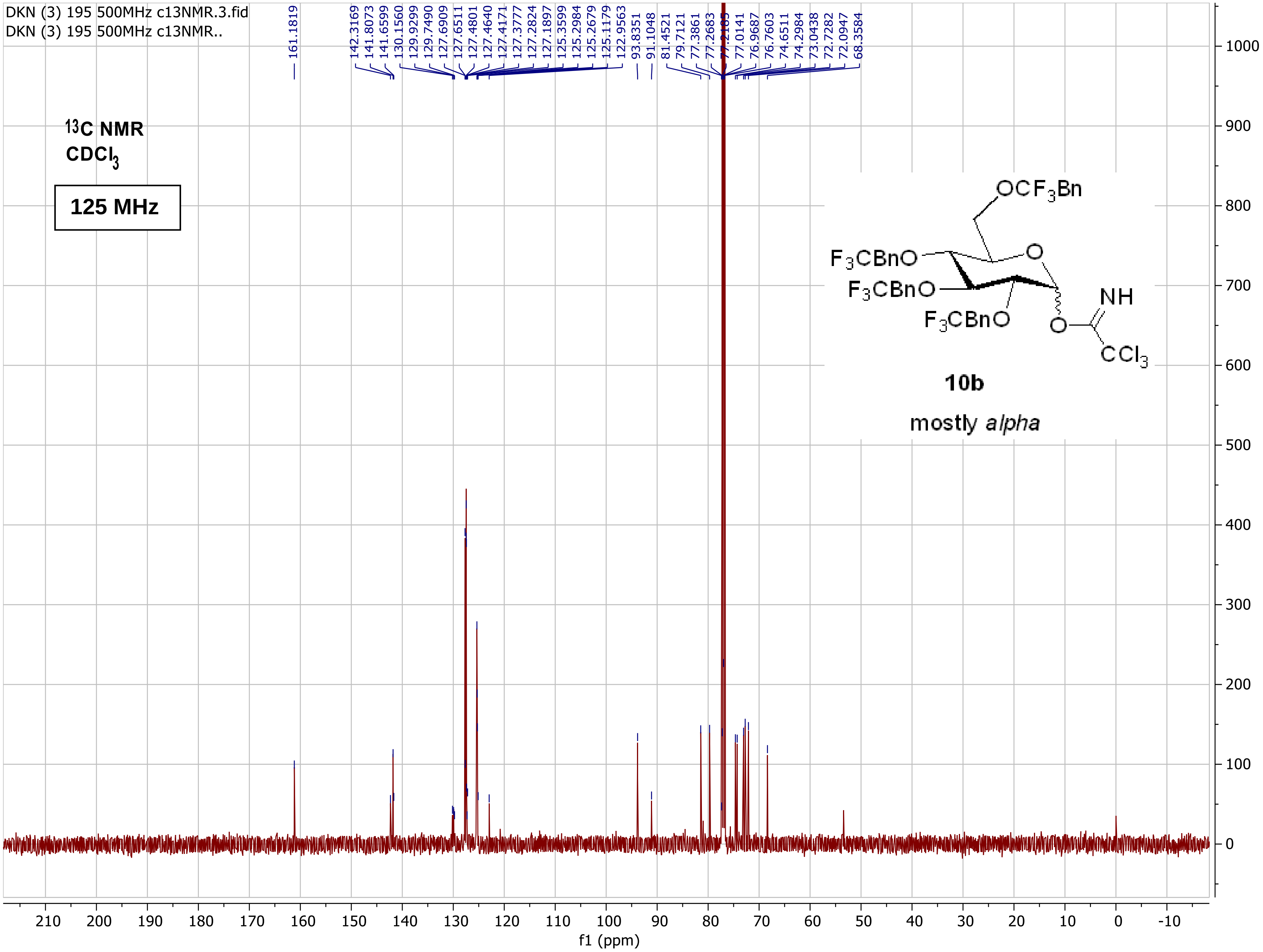

DKN (3) 195 500MHz c13NMR.3.fid
DKN (3) 195 500MHz c13NMR..
161.1819
142.3169
141.8073
141.6599
130.1560
129.9299
129.7490
127.6909
127.6511
127.4801
127.4640
127.4171
127.3777
127.2824
127.1897
125.3599
125.2984
125.2679
125.1179
122.9563
93.8351
91.1048
81.4521
79.7121
77.3861
77.2683
77.0141
76.9687
76.7603
74.6511
74.2984
73.0438
72.7282
72.0947
68.3584
13C NMR
CDCl3
125 MHz
OCF3Bn
F3CBnO
F3CBnO
F3CBnO
O
NH
O
CCl3
10b
mostly alpha
f1 (ppm)

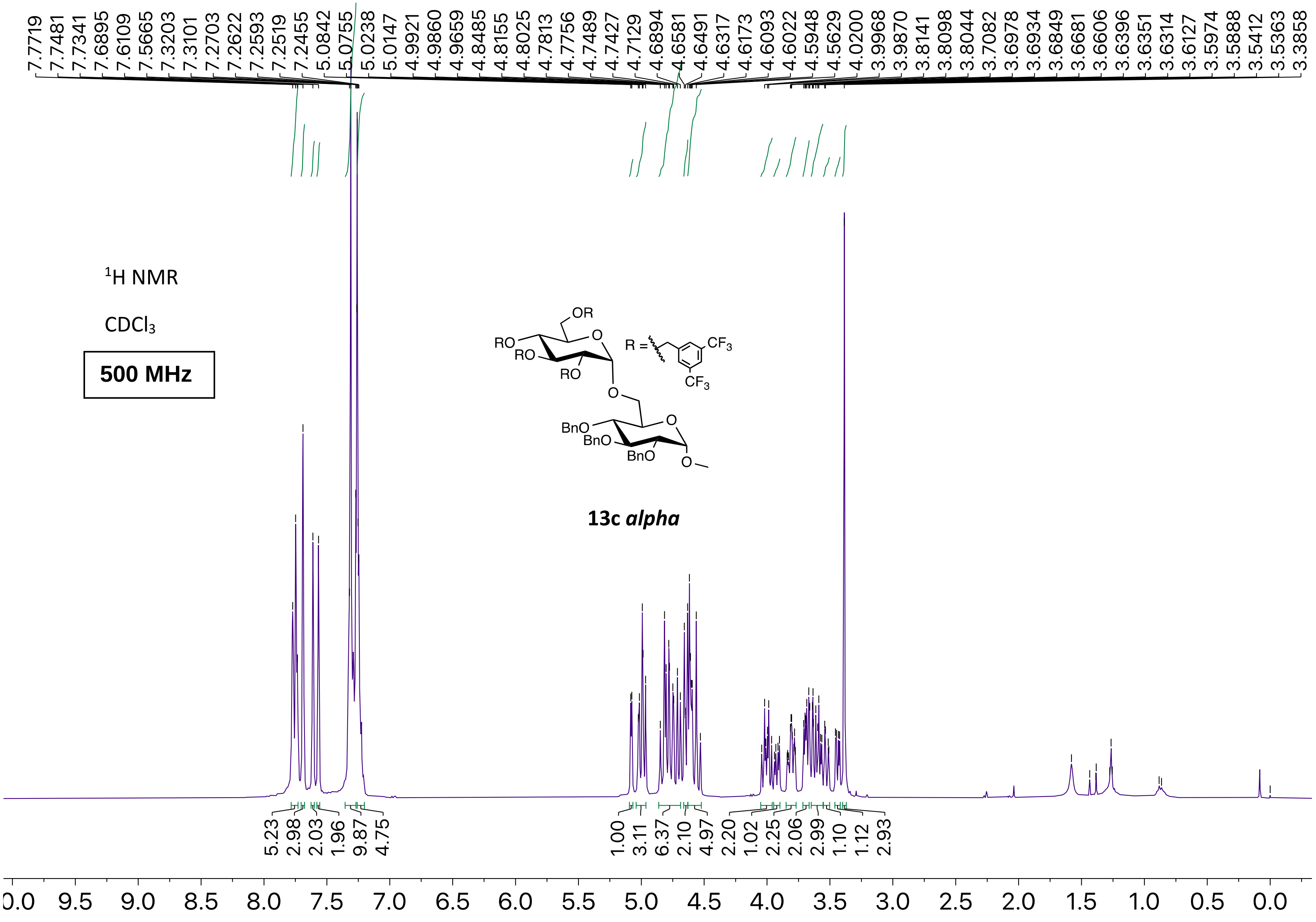

7.7719
7.7481
7.7341
7.6895
7.6109
7.5665
7.3203
7.3101
7.2703
7.2622
7.2593
7.2519
7.2455
5.0842
5.0755
5.0238
5.0147
4.9921
4.9860
4.9659
4.8485
4.8155
4.8025
4.7813
4.7756
4.7489
4.7427
4.7129
4.6894
4.6581
4.6491
4.6317
4.6173
4.6093
4.6022
4.5948
4.5629
4.0200
3.9968
3.9870
3.8141
3.8098
3.8044
3.7082
3.6978
3.6934
3.6849
3.6681
3.6606
3.6396
3.6351
3.6314
3.6127
3.5974
3.5888
3.5412
3.5363
3.3858
^{1}H NMR
$CDCl_3$
500 MHz
OR
RO
RO
RO
O
R =
CF_3
CF_3
O
BnO
BnO
BnO
O
O
13c *alpha*
5.23
2.98
2.03
1.96
9.87
4.75
1.00
3.11
6.37
2.10
4.97
2.20
1.02
2.25
2.06
2.99
1.10
1.12
2.93
0.0
9.5
9.0
8.5
8.0
7.5
7.0
6.5
6.0
5.5
5.0
4.5
4.0
3.5
3.0
2.5
2.0
1.5
1.0
0.5
0.0

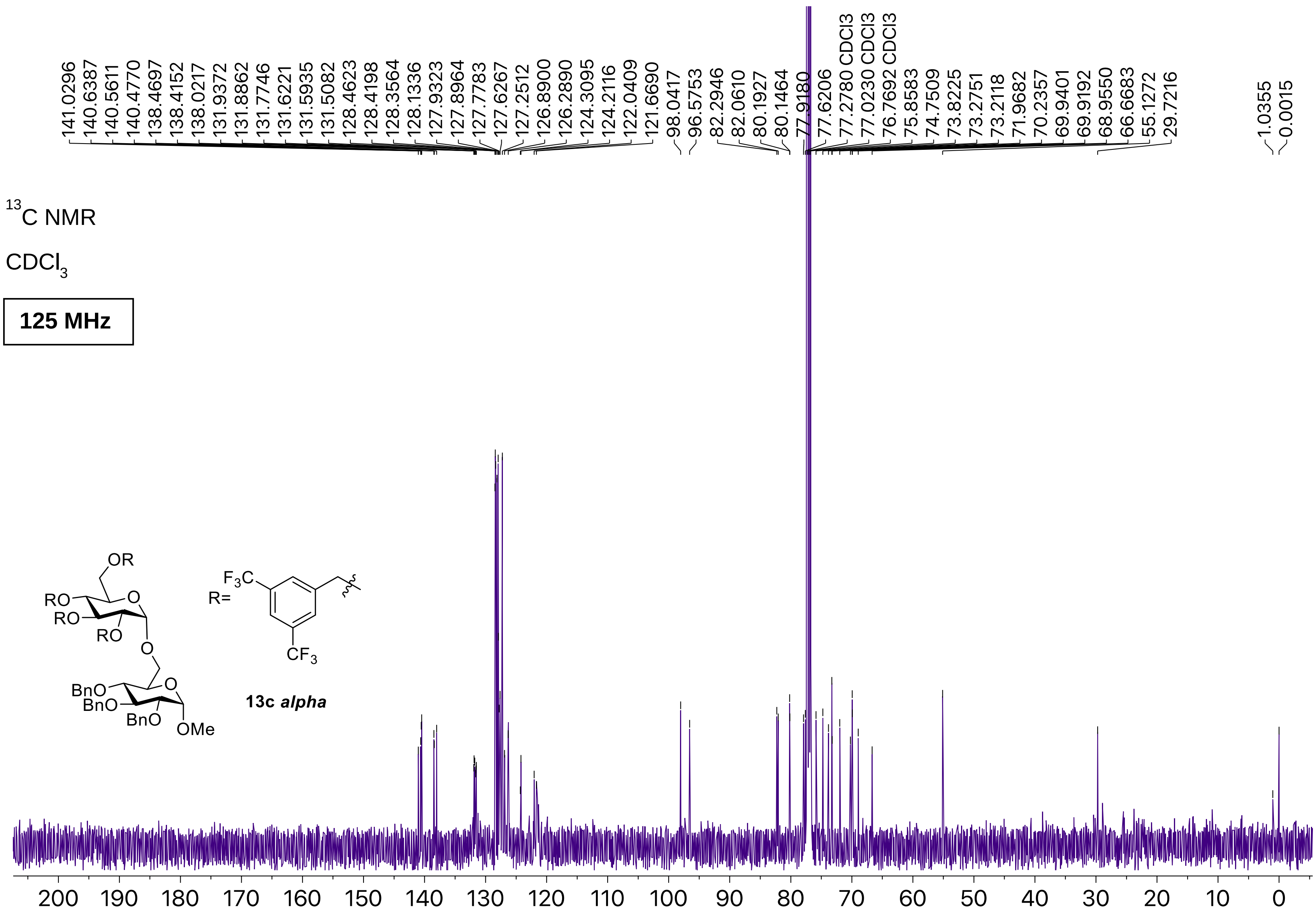
141.0296
140.6387
140.5611
140.4770
138.4697
138.4152
138.0217
131.9372
131.8862
131.7746
131.6221
131.5935
131.5082
128.4623
128.4198
128.3564
128.1336
127.9323
127.8964
127.7783
127.6267
127.2512
126.8900
126.2890
124.3095
124.2116
122.0409
121.6690
98.0417
96.5753
82.2946
82.0610
80.1927
80.1464
77.9180
77.6206
77.2780 CDCl3
77.0230 CDCl3
76.7692 CDCl3
75.8583
74.7509
73.8225
73.2751
73.2118
71.9682
70.2357
69.9401
69.9192
68.9550
66.6683
55.1272
29.7216
1.0355
0.0015
^{13}C NMR
$CDCl_3$
125 MHz
OR
RO
RO
RO
O
O
BnO
BnO
BnO
O
OMe
F_3C
R=
CF_3
200 190 180 170 160 150 140 130 120 110 100 90 80 70 60 50 40 30 20 10 0
13c *alpha*

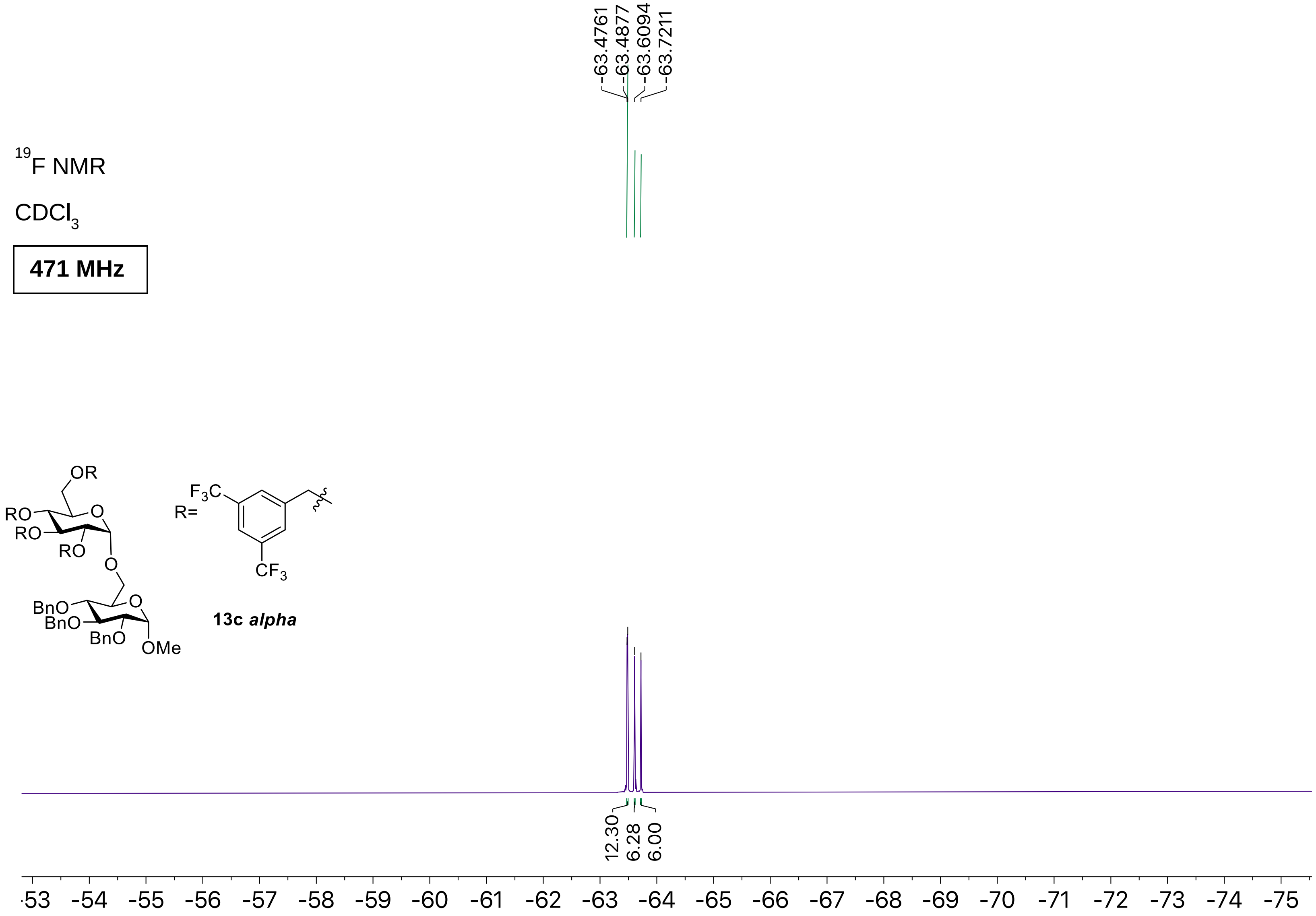

-63.4761
-63.4877
-63.6094
-63.7211
19F NMR
CDCl3
471 MHz
OR
RO
RO
RO
O
O
BnO
BnO
BnO
O
OMe
R=
F3C
CF3
13c alpha
12.30
6.28
6.00
-53 -54 -55 -56 -57 -58 -59 -60 -61 -62 -63 -64 -65 -66 -67 -68 -69 -70 -71 -72 -73 -74 -75

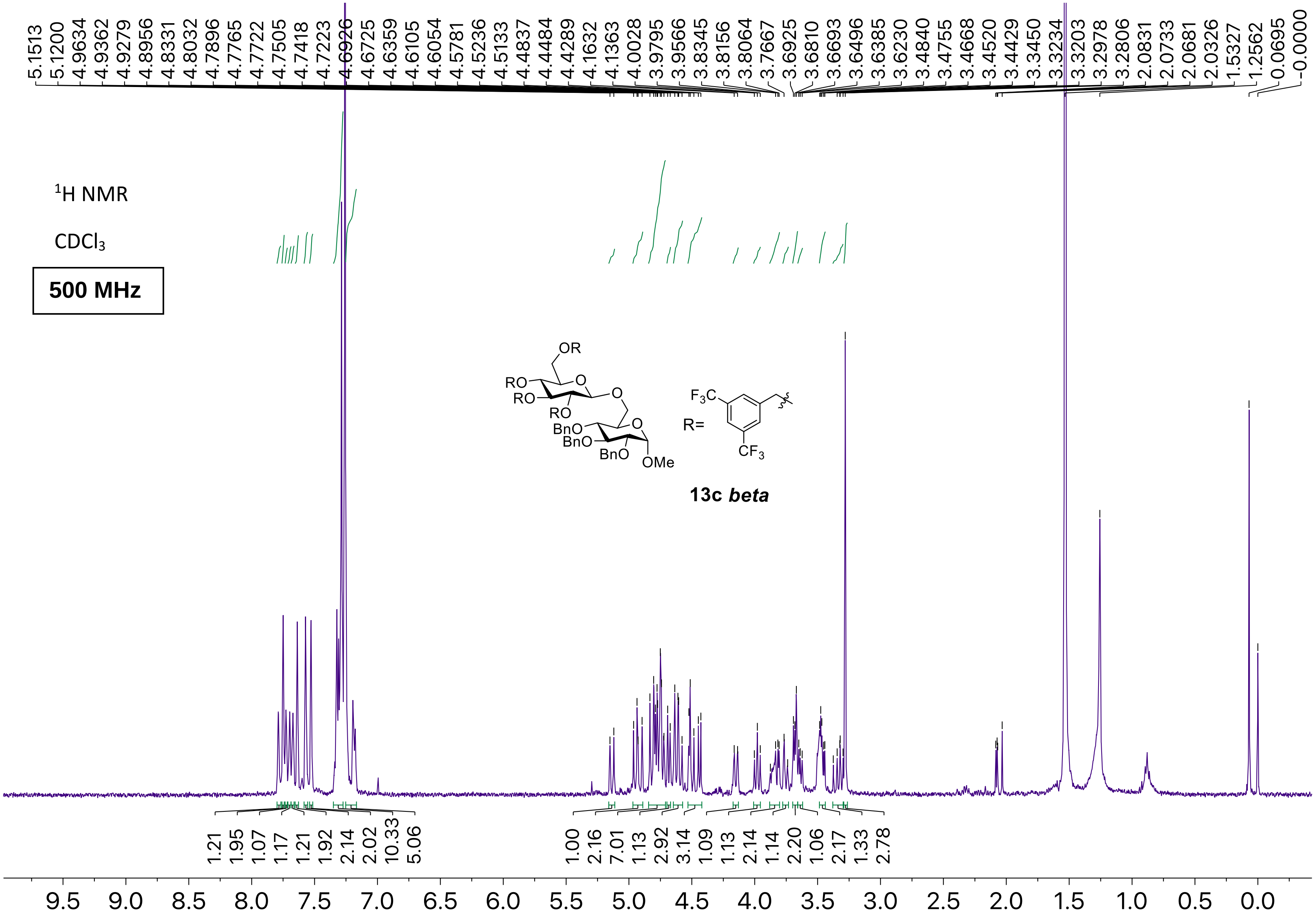
5.1513
5.1200
4.9634
4.9362
4.9279
4.8956
4.8331
4.8032
4.7896
4.7765
4.7722
4.7505
4.7418
4.7223
4.6926
4.6725
4.6359
4.6105
4.6054
4.5781
4.5236
4.5133
4.4837
4.4484
4.4289
4.1632
4.1363
4.0028
3.9795
3.9566
3.8345
3.8156
3.8064
3.7667
3.6925
3.6810
3.6693
3.6496
3.6385
3.6230
3.4840
3.4755
3.4668
3.4520
3.4429
3.3450
3.3234
3.3203
3.2978
3.2806
2.0831
2.0733
2.0681
2.0326
1.5327
1.2562
0.0695
-0.0000
1H NMR
CDCl3
500 MHz
OR
RO
RO
RO
O
O
O
BnO
BnO
BnO
OMe
R=
F3C
CF3
13c *beta*
1.21
1.95
1.07
1.17
1.21
1.92
2.14
2.02
10.33
5.06
1.00
2.16
7.01
1.13
2.92
3.14
1.09
1.13
2.14
1.14
2.20
1.06
2.17
1.33
2.78
9.5
9.0
8.5
8.0
7.5
7.0
6.5
6.0
5.5
5.0
4.5
4.0
3.5
3.0
2.5
2.0
1.5
1.0
0.5
0.0

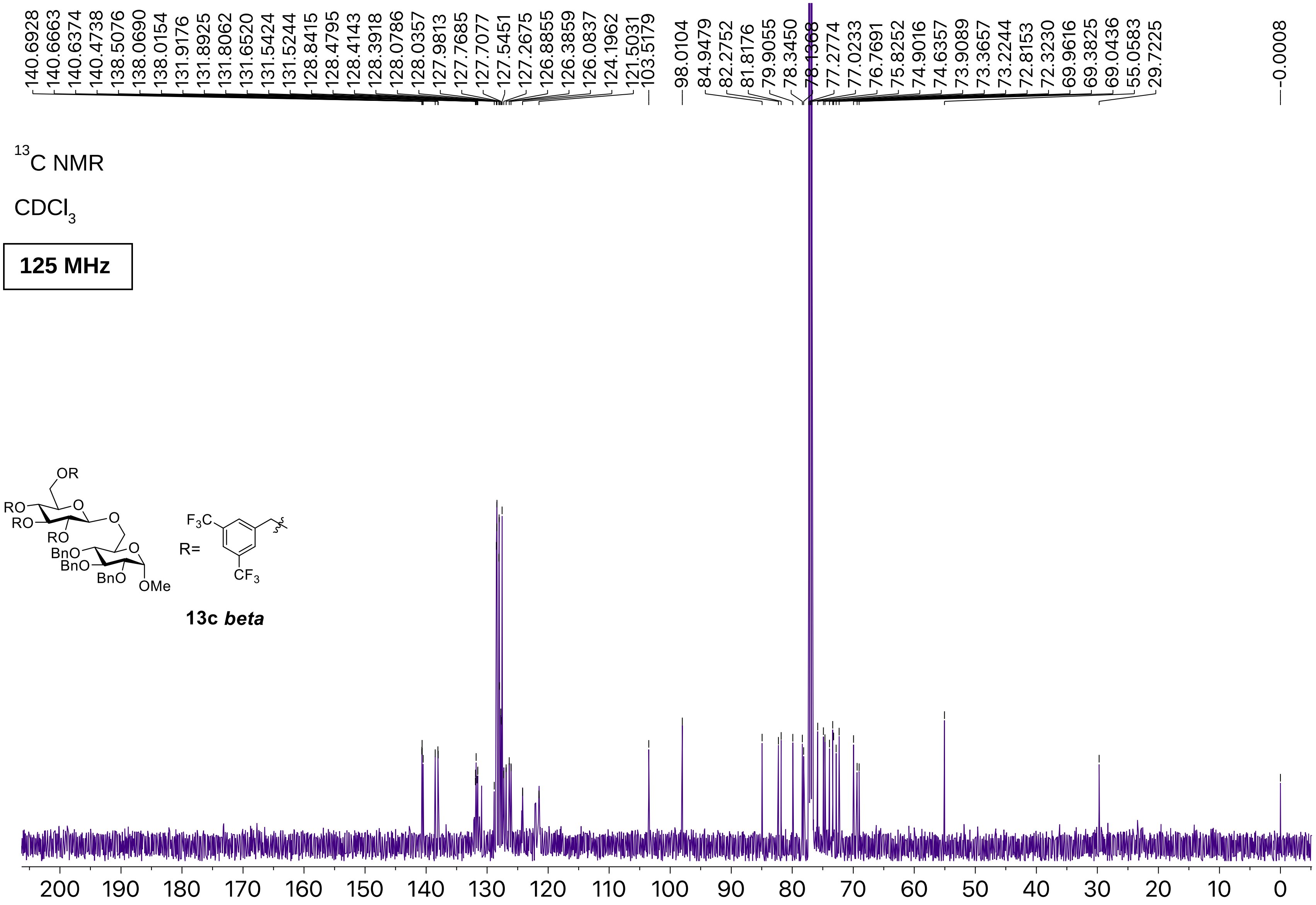
13C NMR
CDCl3
125 MHz
140.6928
140.6663
140.6374
140.4738
138.5076
138.0690
138.0154
131.9176
131.8925
131.8062
131.6520
131.5424
131.5244
128.8415
128.4795
128.4143
128.3918
128.0786
128.0357
127.9813
127.7685
127.7077
127.5451
127.2675
126.8855
126.3859
126.0837
124.1962
121.5031
103.5179
98.0104
84.9479
82.2752
81.8176
79.9055
78.3450
77.2774
77.0233
76.7691
75.8252
74.9016
74.6357
73.9089
73.3657
73.2244
72.8153
72.3230
69.9616
69.3825
69.0436
55.0583
29.7225
-0.0008
OR
RO
O
O
RO
RO
BnO
O
BnO
BnO
OMe
R=
F3C
CF3
13c beta
200
190
180
170
160
150
140
130
120
110
100
90
80
70
60
50
40
30
20
10
0

^{19}F NMR

$CDCl_3$

471 MHz

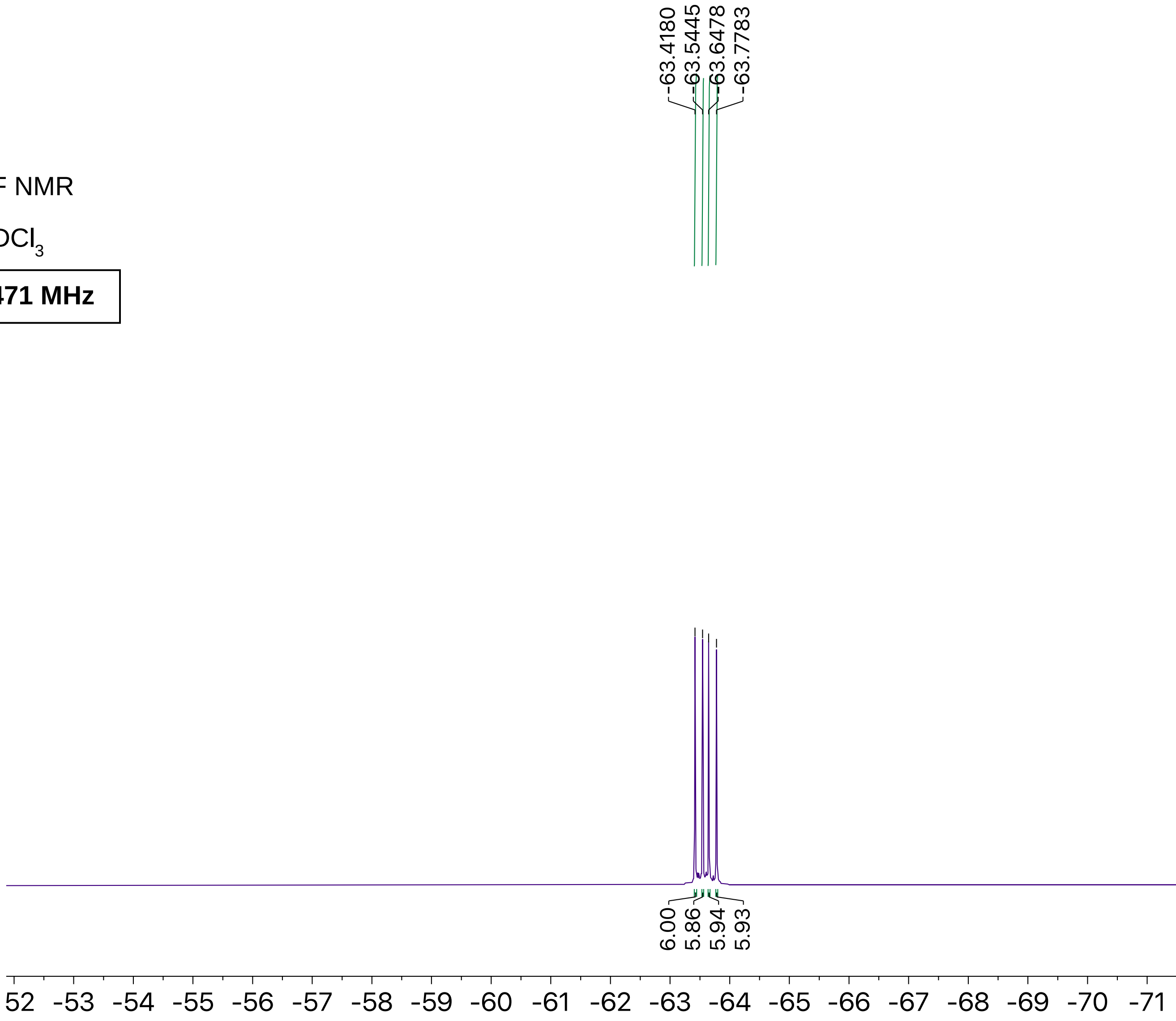

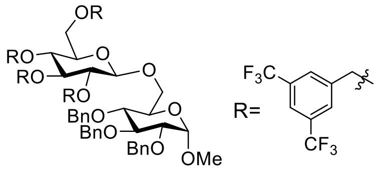

13c *beta*

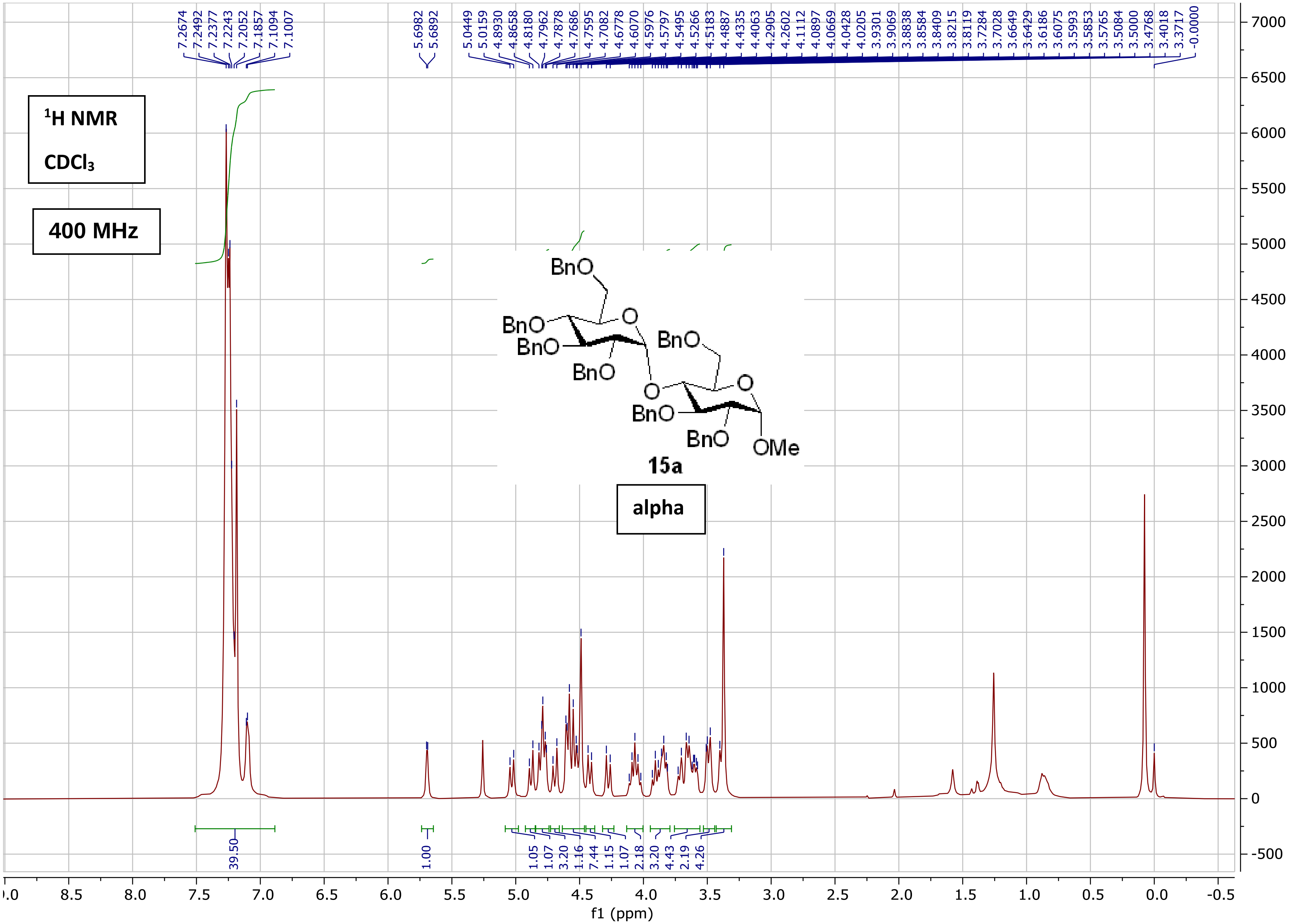

¹H NMR
CDCl₃
400 MHz
15a
alpha
f1 (ppm)

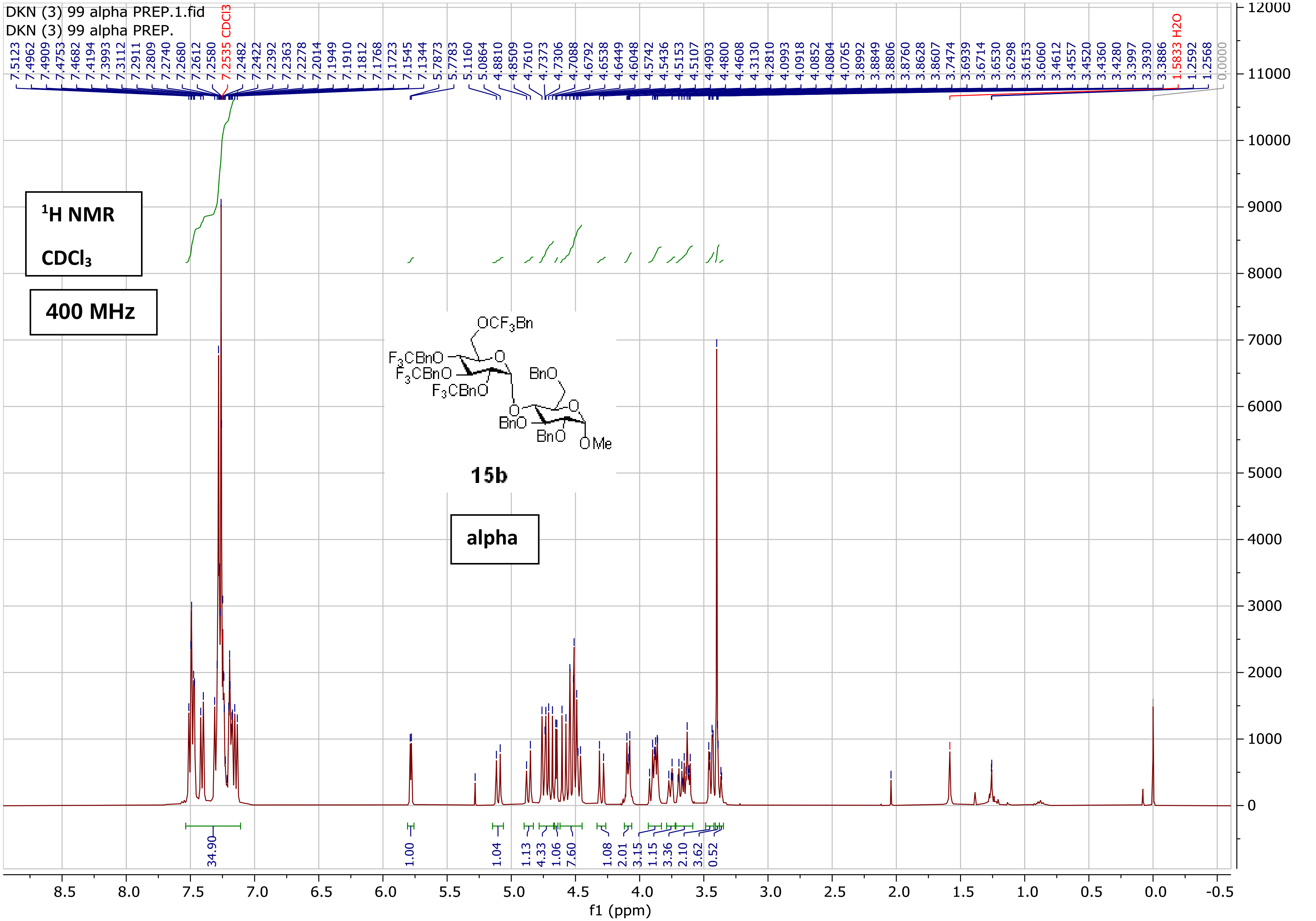

DKN (3) 99 alpha PREP.1.fid
DKN (3) 99 alpha PREP.
1H NMR
CDCl3
400 MHz
OCF3Bn
F3CBnO
F3CBnO
F3CBnO
BnO
O
BnO
BnO
OMe
15b
alpha
7.2535 CDCl3
1.5833 H2O
f1 (ppm)

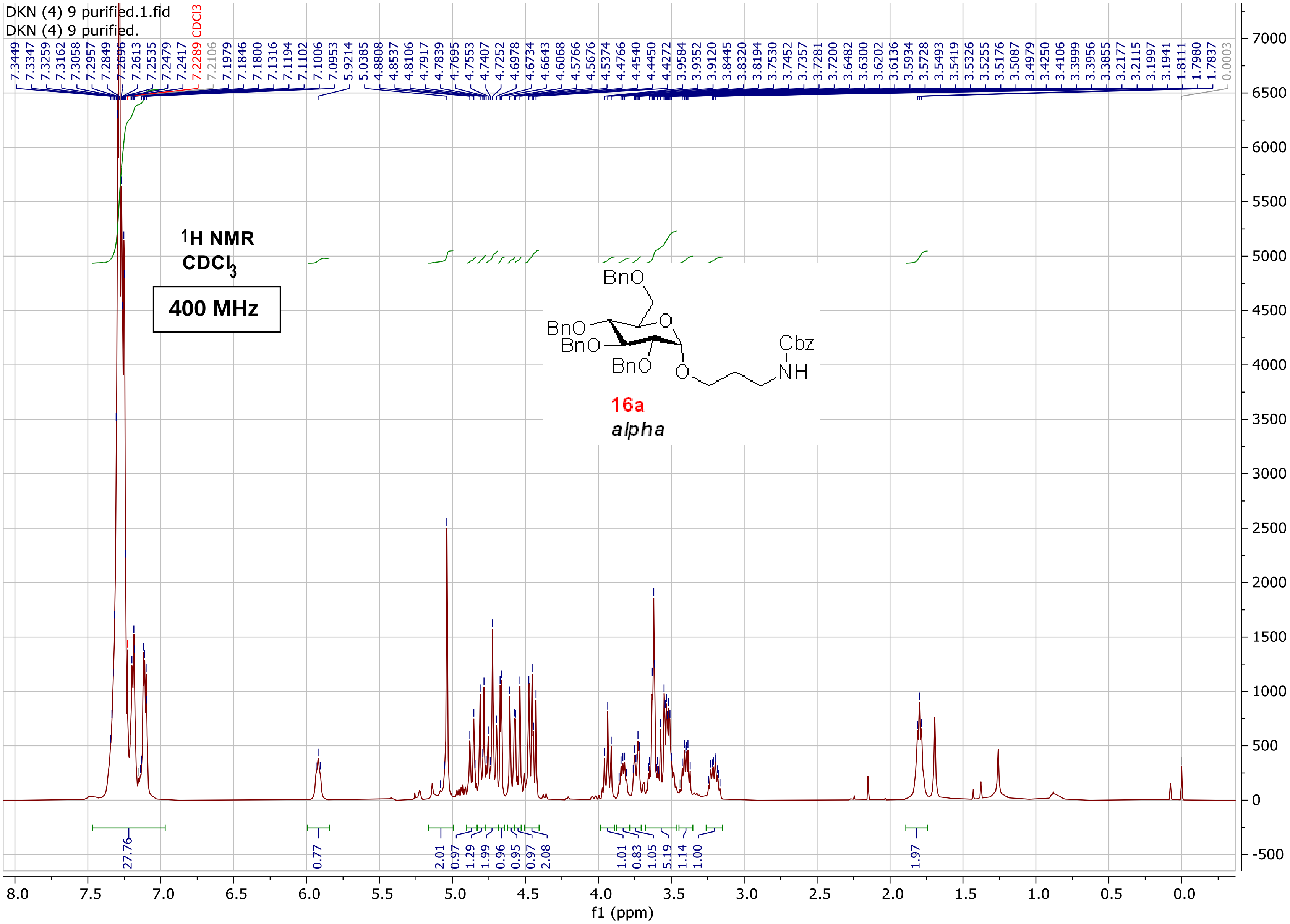
DKN (4) 9 purified.1.fid
DKN (4) 9 purified.
1H NMR
CDCl3
400 MHz
BnO
BnO
BnO
BnO
O
O
Cbz
NH
16a
alpha
f1 (ppm)

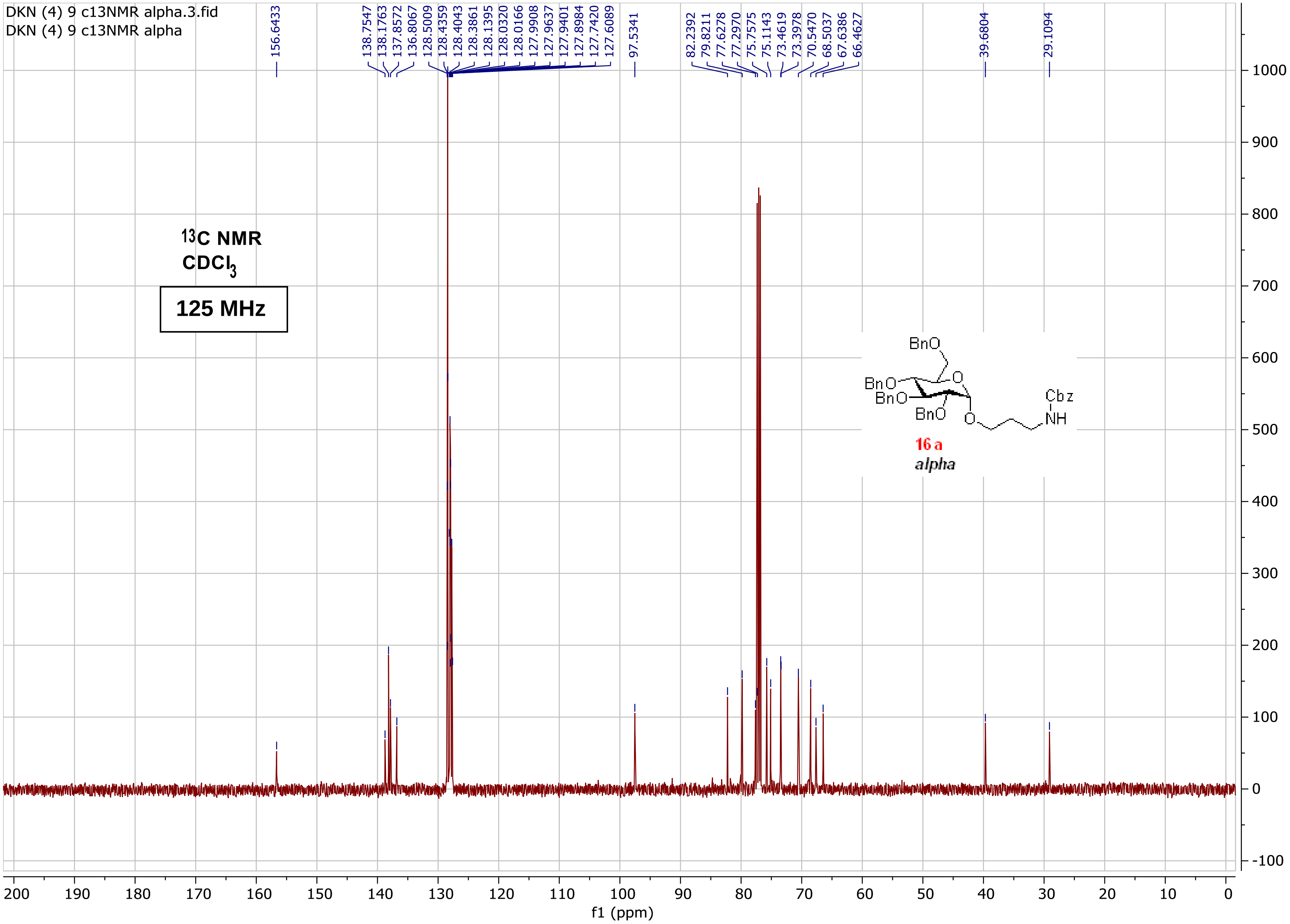
DKN (4) 9 c13NMR alpha.3.fid
DKN (4) 9 c13NMR alpha
156.6433
138.7547
138.1763
137.8572
136.8067
128.5009
128.4359
128.4043
128.3861
128.1395
128.0320
128.0166
127.9908
127.9637
127.9401
127.8984
127.7420
127.6089
97.5341
82.2392
79.8211
77.6278
77.2970
75.7575
75.1143
73.4619
73.3978
70.5470
68.5037
67.6386
66.4627
39.6804
29.1094
13C NMR
CDCl3
125 MHz
BnO
BnO
BnO
BnO
O
O
Cbz
NH
16 a
alpha
f1 (ppm)

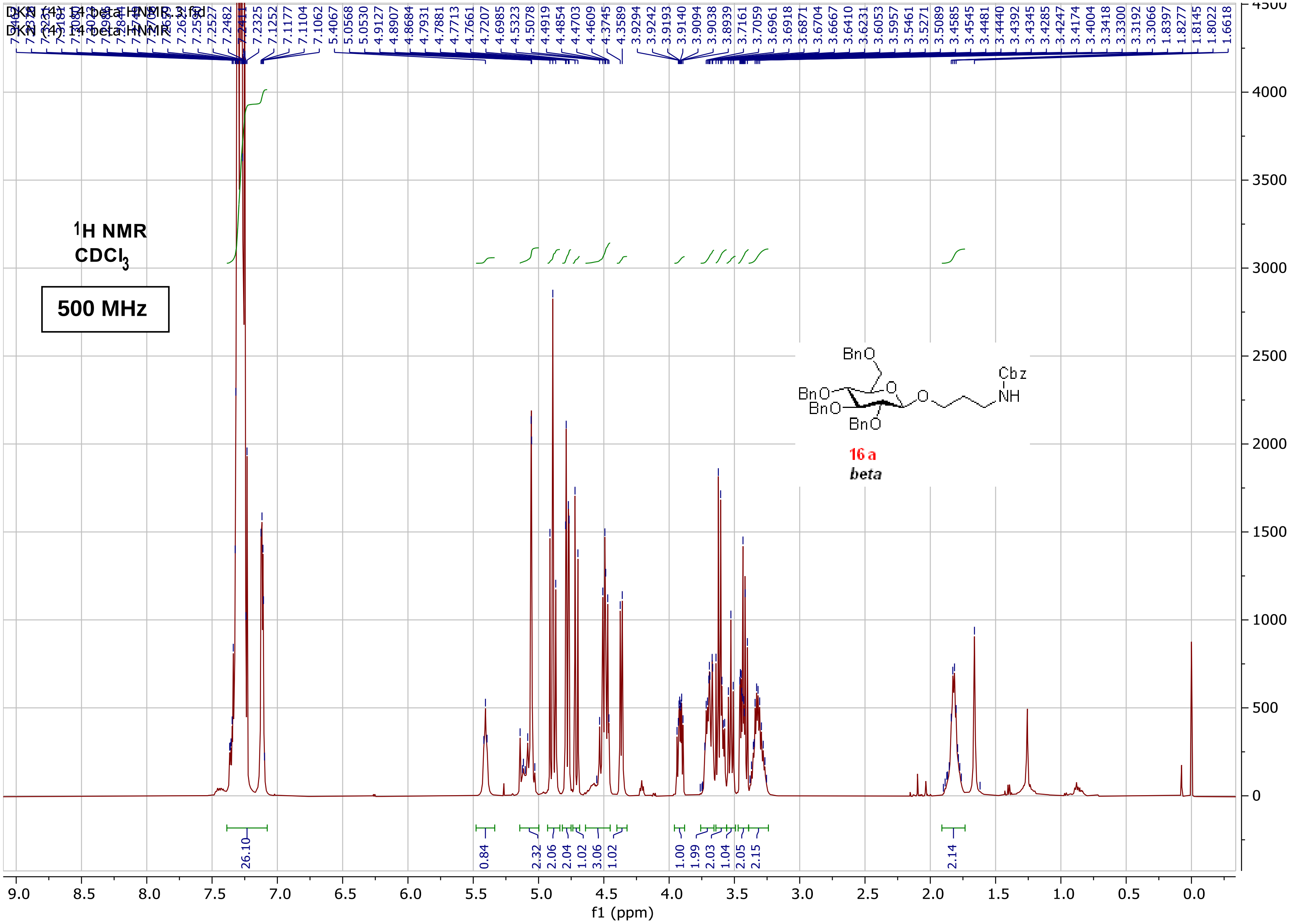

1H NMR
CDCl3
500 MHz
16 a
beta
f1 (ppm)

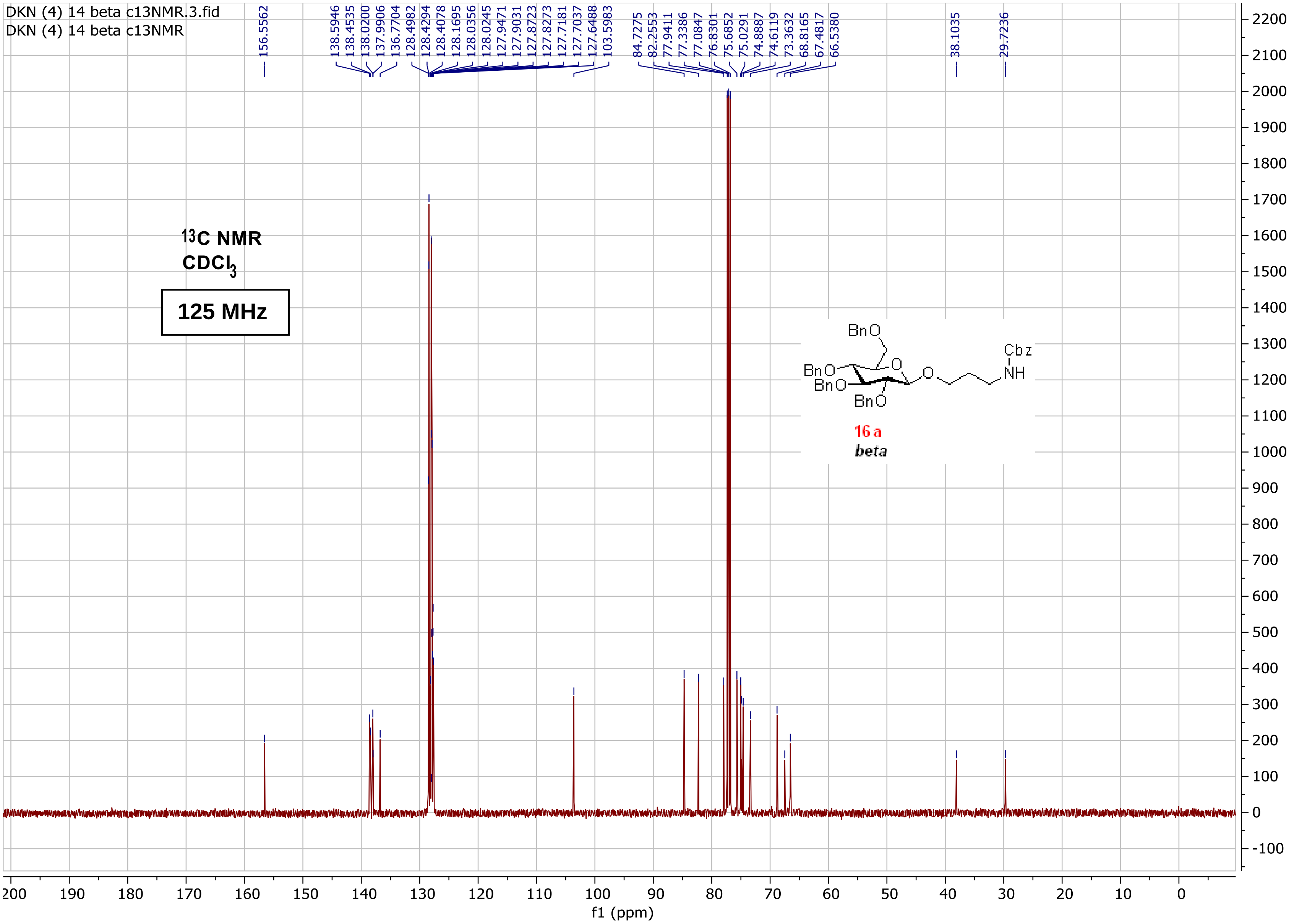
DKN (4) 14 beta c13NMR.3.fid
DKN (4) 14 beta c13NMR
156.5562
138.5946
138.4535
138.0200
137.9906
136.7704
128.4982
128.4294
128.4078
128.1695
128.0356
128.0245
127.9471
127.9031
127.8723
127.8273
127.7181
127.7037
127.6488
103.5983
84.7275
82.2553
77.9411
77.3386
77.0847
76.8301
75.6852
75.0291
74.8887
74.6119
73.3632
68.8165
67.4817
66.5380
38.1035
29.7236
13C NMR
CDCl3
125 MHz
BnO
BnO
BnO
BnO
O
O
Cbz
NH
16 a
beta
f1 (ppm)

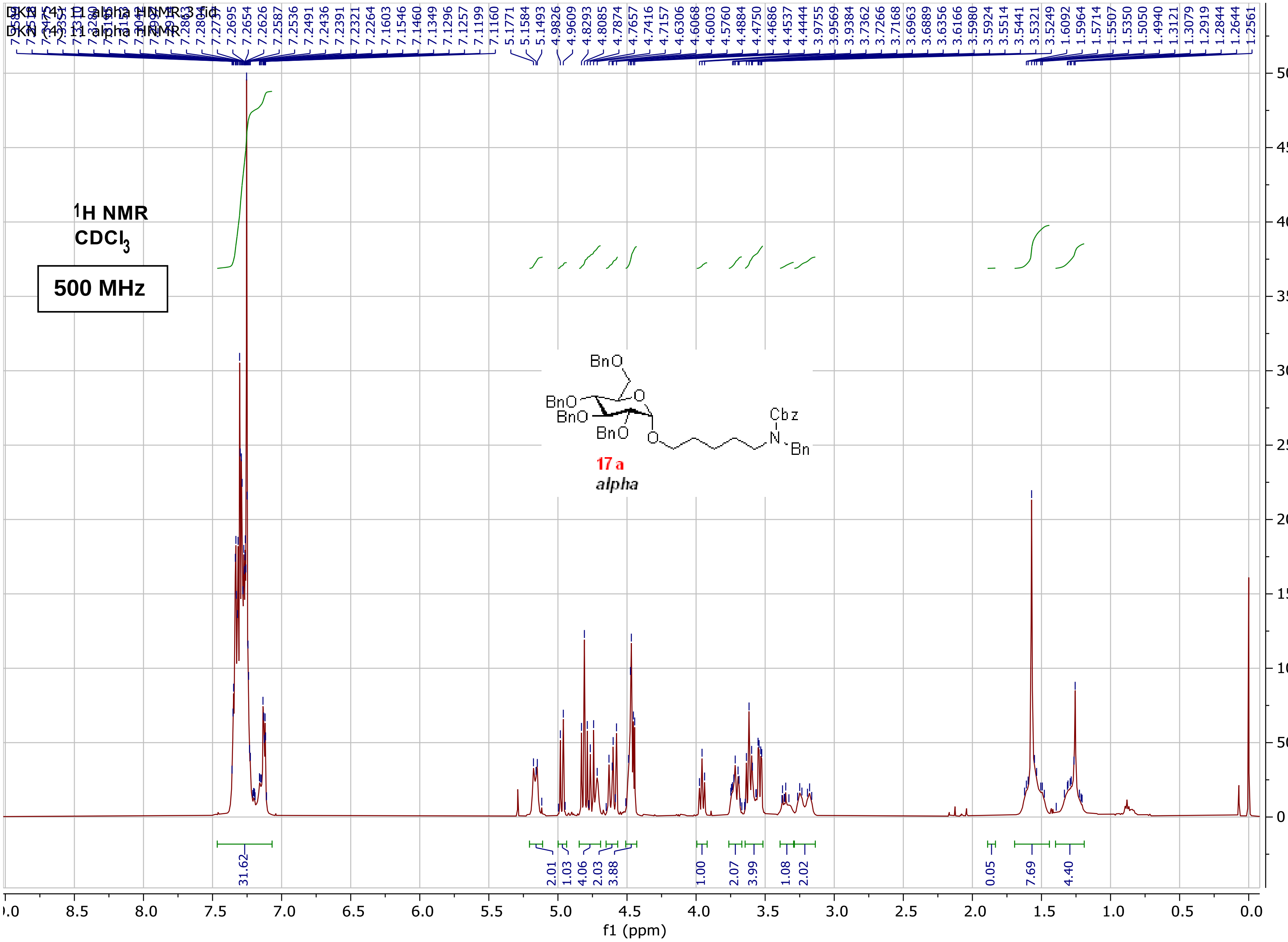

1H NMR
CDCl3
500 MHz
BnO
BnO
BnO
BnO
Cbz
N
Bn
17a
alpha
f1 (ppm)

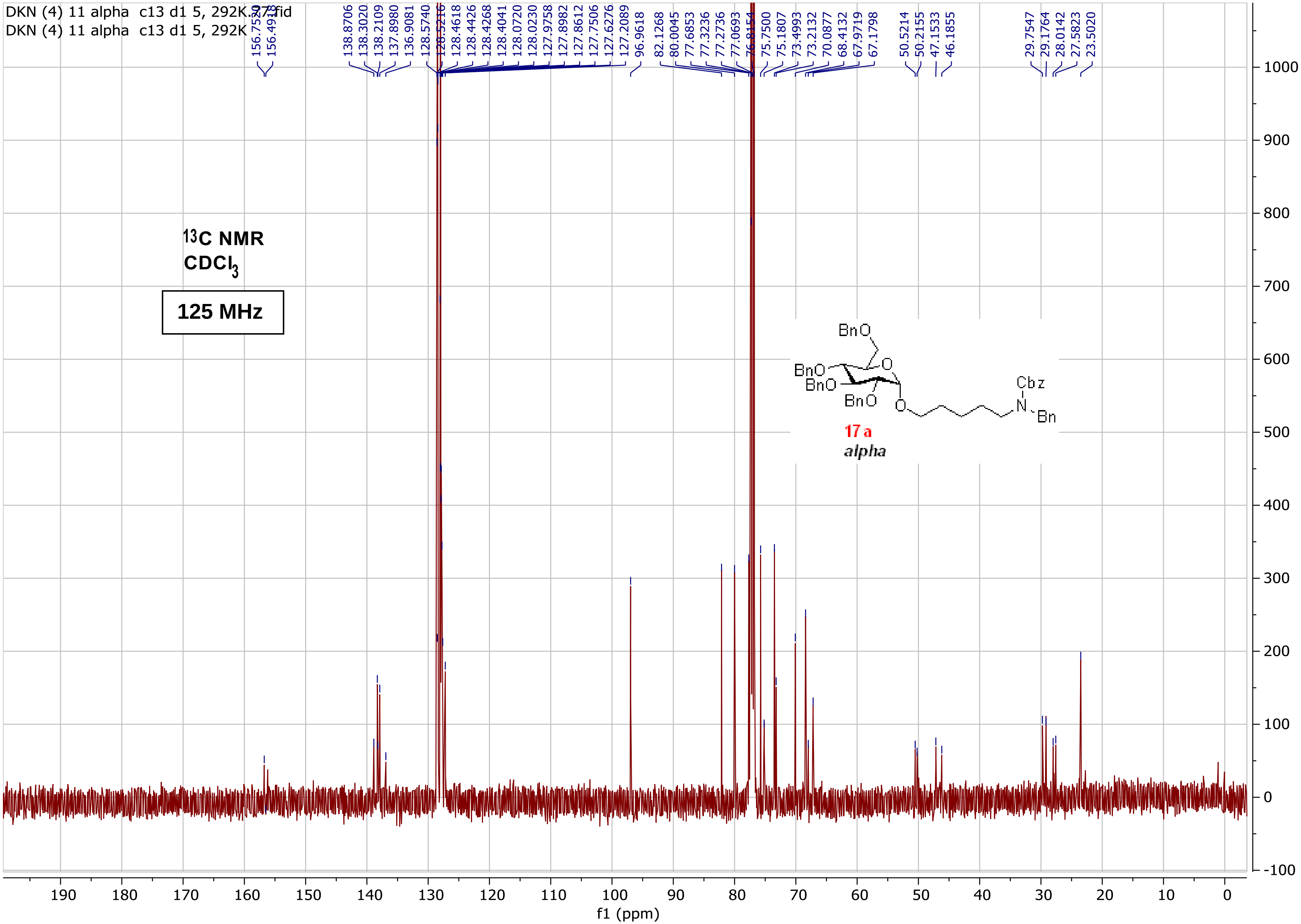
DKN (4) 11 alpha c13 d1 5, 292K.97.fid
DKN (4) 11 alpha c13 d1 5, 292K
156.7529
156.4918
138.8706
138.3020
138.2109
137.8980
136.9081
128.5740
128.5216
128.4618
128.4426
128.4268
128.4041
128.0720
128.0230
127.9758
127.8982
127.8612
127.7506
127.6276
127.2089
96.9618
82.1268
80.0045
77.6853
77.3236
77.2736
77.0693
76.8154
75.7500
75.1807
73.4993
73.2132
70.0877
68.4132
67.9719
67.1798
50.5214
50.2155
47.1533
46.1855
29.7547
29.1764
28.0142
27.5823
23.5020
13C NMR
CDCl3
125 MHz
BnO
BnO
BnO
O
BnO
O
Cbz
N
Bn
17 a
alpha
f1 (ppm)

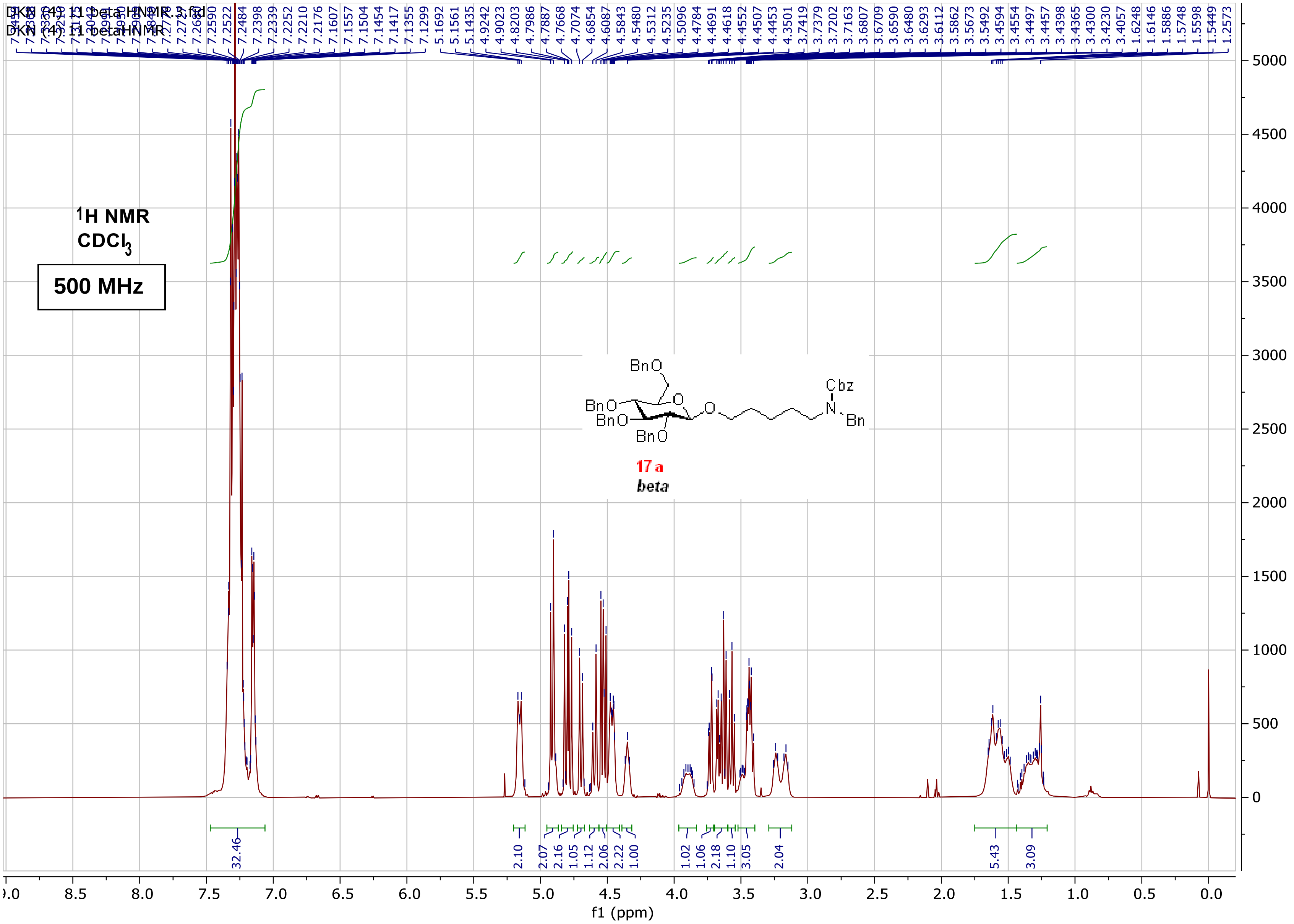

1H NMR
CDCl3
500 MHz
BnO
BnO
BnO
BnO
O
O
Cbz
N
Bn
17a
beta
f1 (ppm)
32.46
2.10
2.07
2.16
1.05
1.12
2.06
2.22
1.00
1.02
1.06
2.18
1.10
3.05
2.04
5.43
3.09

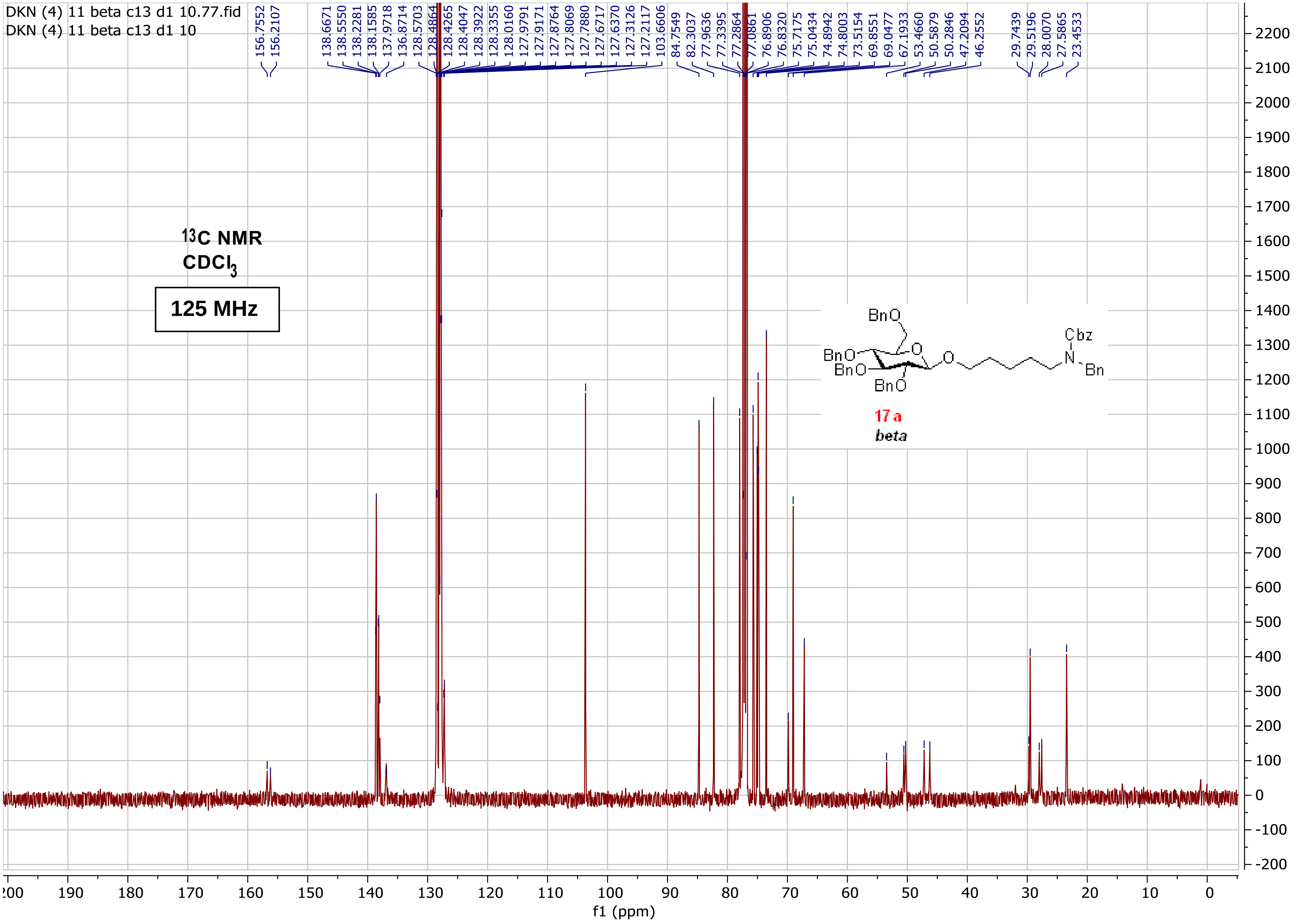
DKN (4) 11 beta c13 d1 10.77.fid
DKN (4) 11 beta c13 d1 10
156.7552
156.2107
138.6671
138.5550
138.2281
138.1585
137.9718
136.8714
128.5703
128.4864
128.4265
128.4047
128.3922
128.3355
128.0160
127.9791
127.9171
127.8764
127.8069
127.7880
127.6717
127.6370
127.3126
127.2117
103.6606
84.7549
82.3037
77.9636
77.3395
77.2864
77.0861
76.8906
76.8320
75.7175
75.0434
74.8942
74.8003
73.5154
69.8551
69.0477
67.1933
53.4660
50.5879
50.2846
47.2094
46.2552
29.7439
29.5196
28.0070
27.5865
23.4533
13C NMR
CDCl3
125 MHz
BnO
BnO
BnO
BnO
O
O
Cbz
N
Bn
17 a
beta
f1 (ppm)

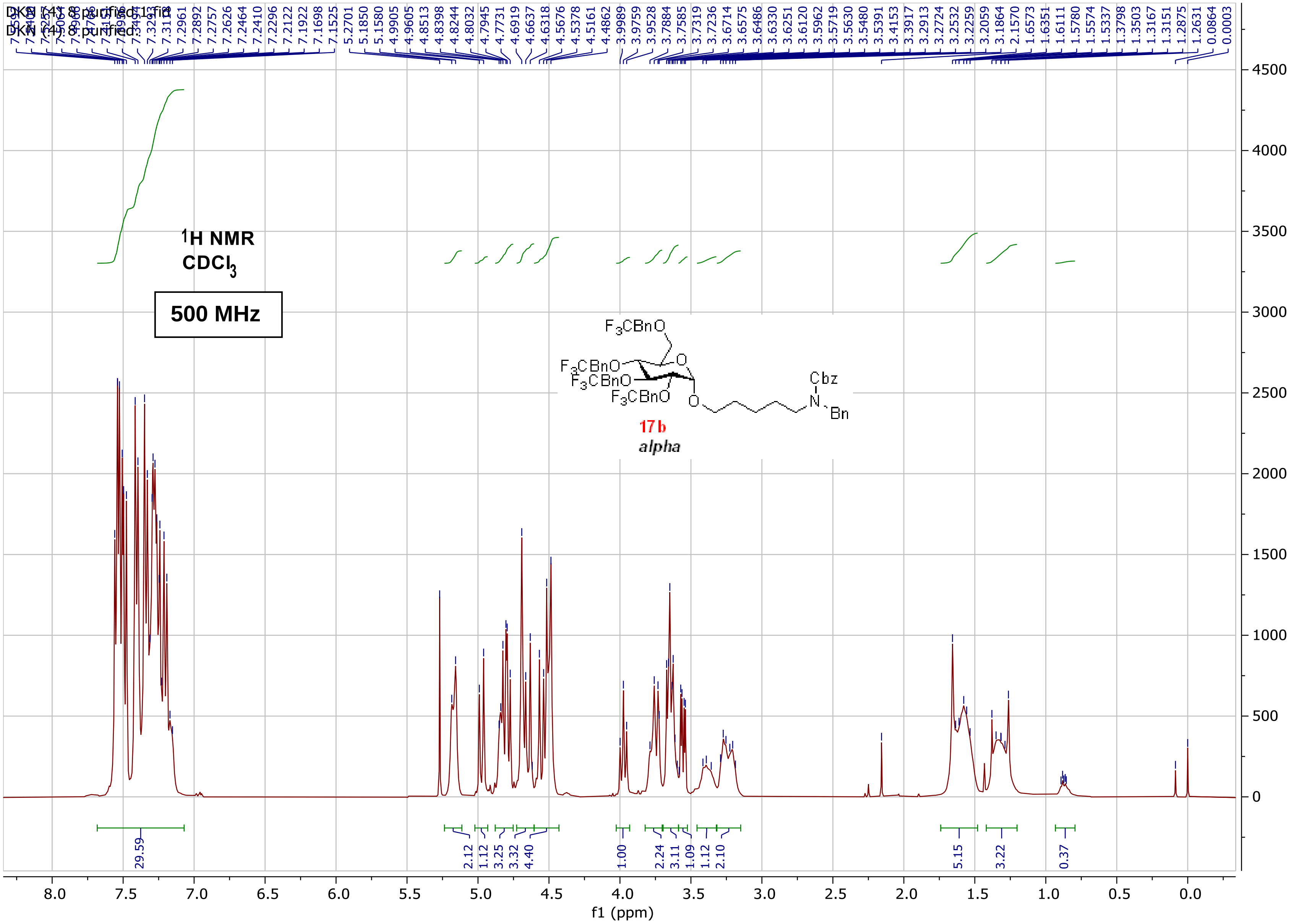

DKN 74 8 purified 1 fid
DKN (4) 8 purified
1H NMR
CDCl3
500 MHz
F3CBnO
F3CBnO
F3CBnO
F3CBnO
O
O
Cbz
N
Bn
17b
alpha
f1 (ppm)

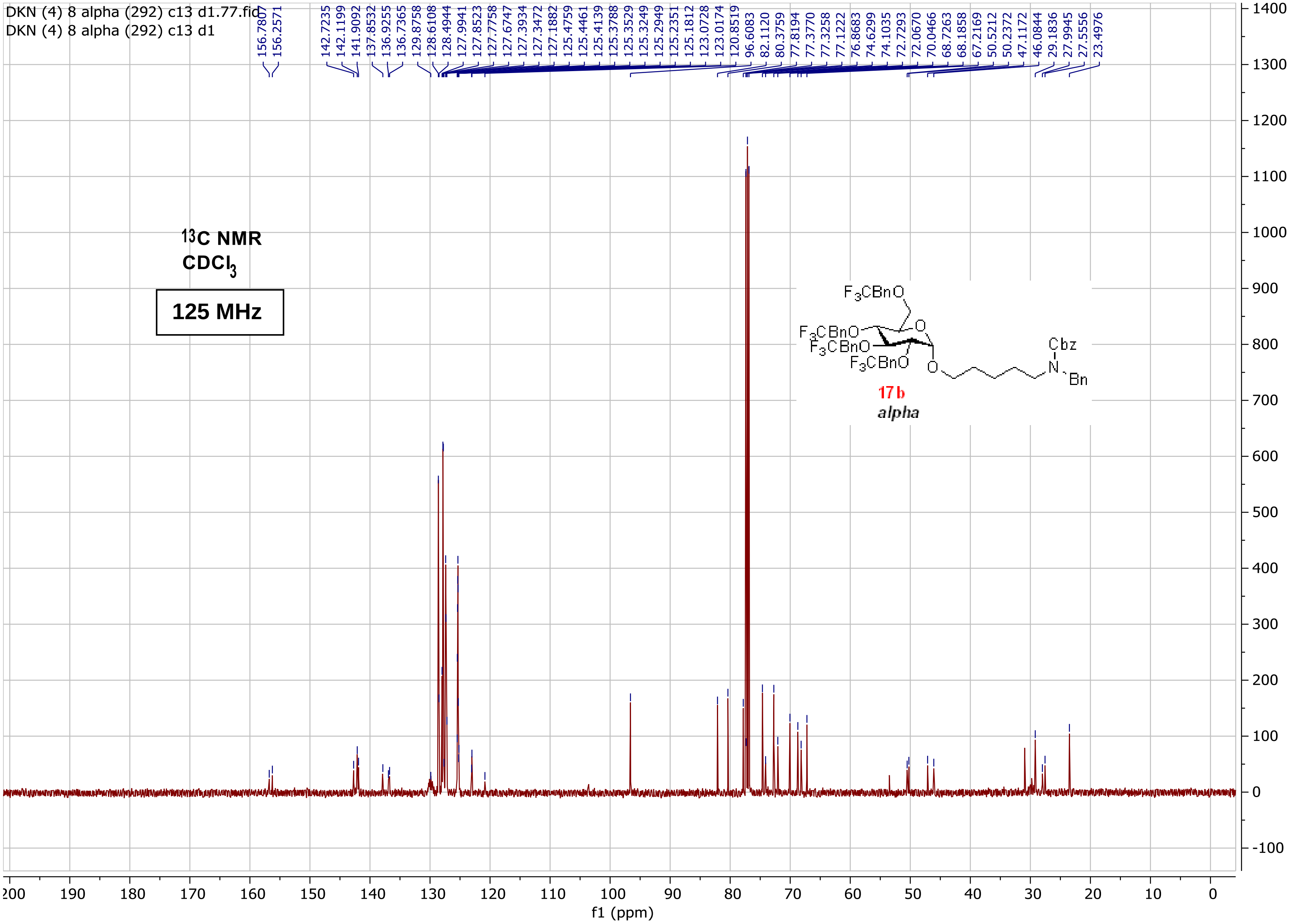

DKN (4) 8 alpha (292) c13 d1.77.fid
DKN (4) 8 alpha (292) c13 d1
156.7807
156.2571
142.7235
142.1199
141.9092
137.8532
136.9255
136.7365
129.8758
128.6108
128.4944
127.9941
127.8523
127.7758
127.6747
127.3934
127.3472
127.1882
125.4759
125.4461
125.4139
125.3788
125.3529
125.3249
125.2949
125.2351
125.1812
123.0728
123.0174
120.8519
96.6083
82.1120
80.3759
77.8194
77.3770
77.3258
77.1222
76.8683
74.6299
74.1035
72.7293
72.0670
70.0466
68.7263
68.1858
67.2169
50.5212
50.2372
47.1172
46.0844
29.1836
27.9945
27.5556
23.4976
13C NMR
CDCl3
125 MHz
F3CBnO
F3CBnO
F3CBnO
F3CBnO
O
O
Cbz
N
Bn
17b
alpha
f1 (ppm)

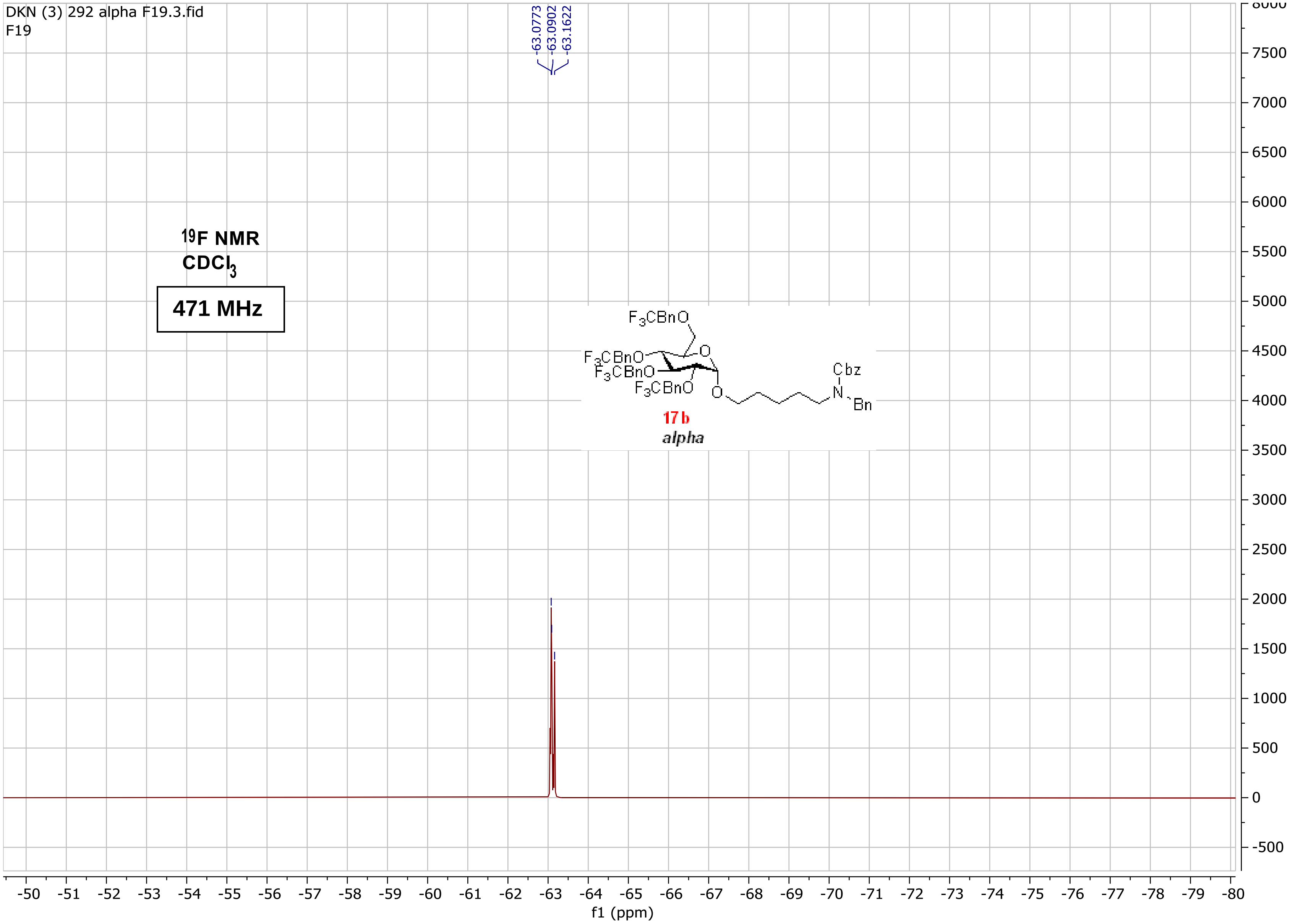
DKN (3) 292 alpha F19.3.fid
F19
-63.0773
-63.0902
-63.1622
19F NMR
CDCl3
471 MHz
F3CBnO
F3CBnO
F3CBnO
F3CBnO
O
O
Cbz
N
Bn
17b
alpha
f1 (ppm)

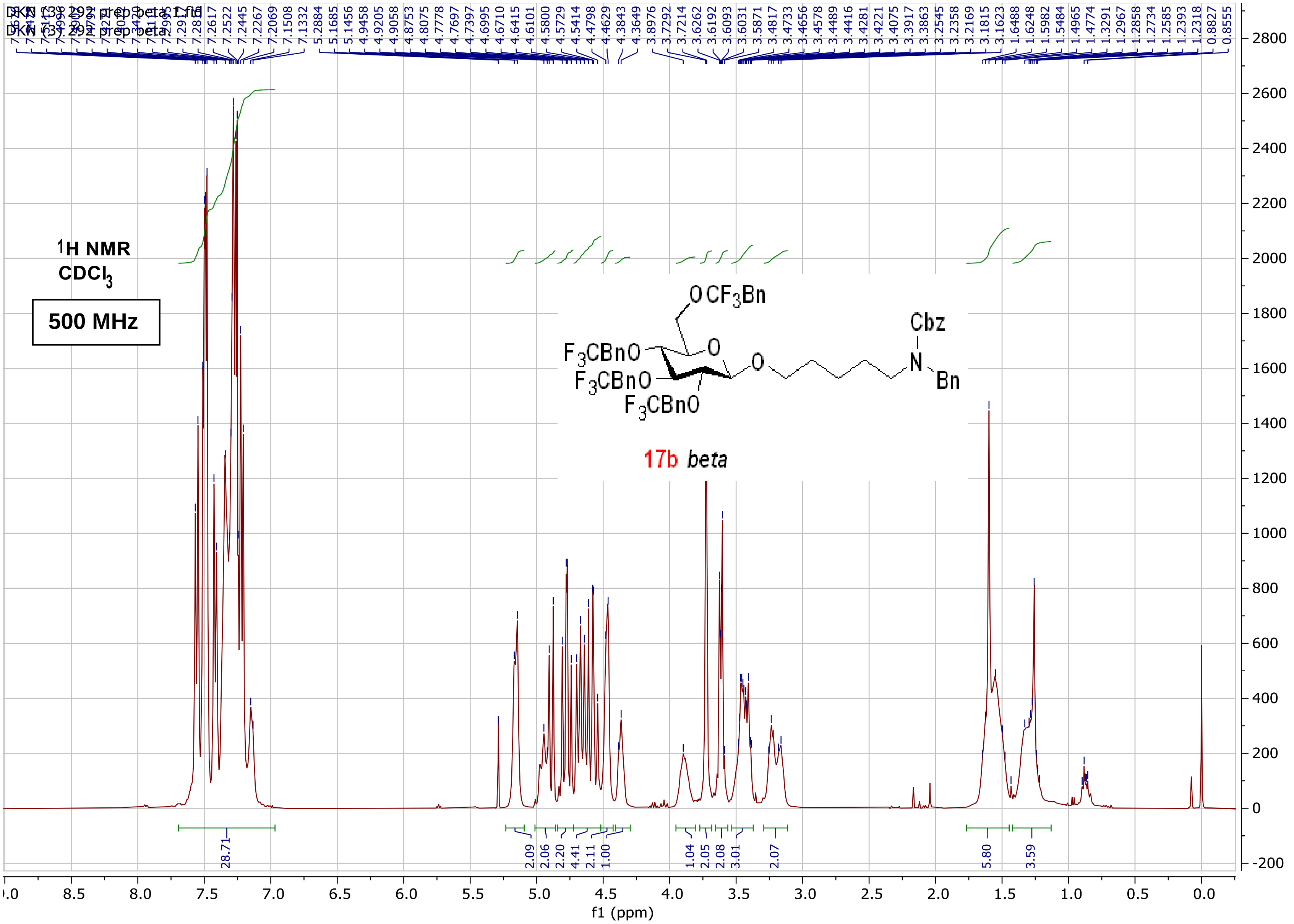

DKN (3) 292 prep beta
1H NMR
CDCl3
500 MHz
OCF3Bn
F3CBnO
F3CBnO
F3CBnO
O
O
Cbz
N
Bn
17b beta
28.71
2.09
2.06
2.20
4.41
2.11
1.00
1.04
2.05
2.08
3.01
2.07
5.80
3.59
f1 (ppm)

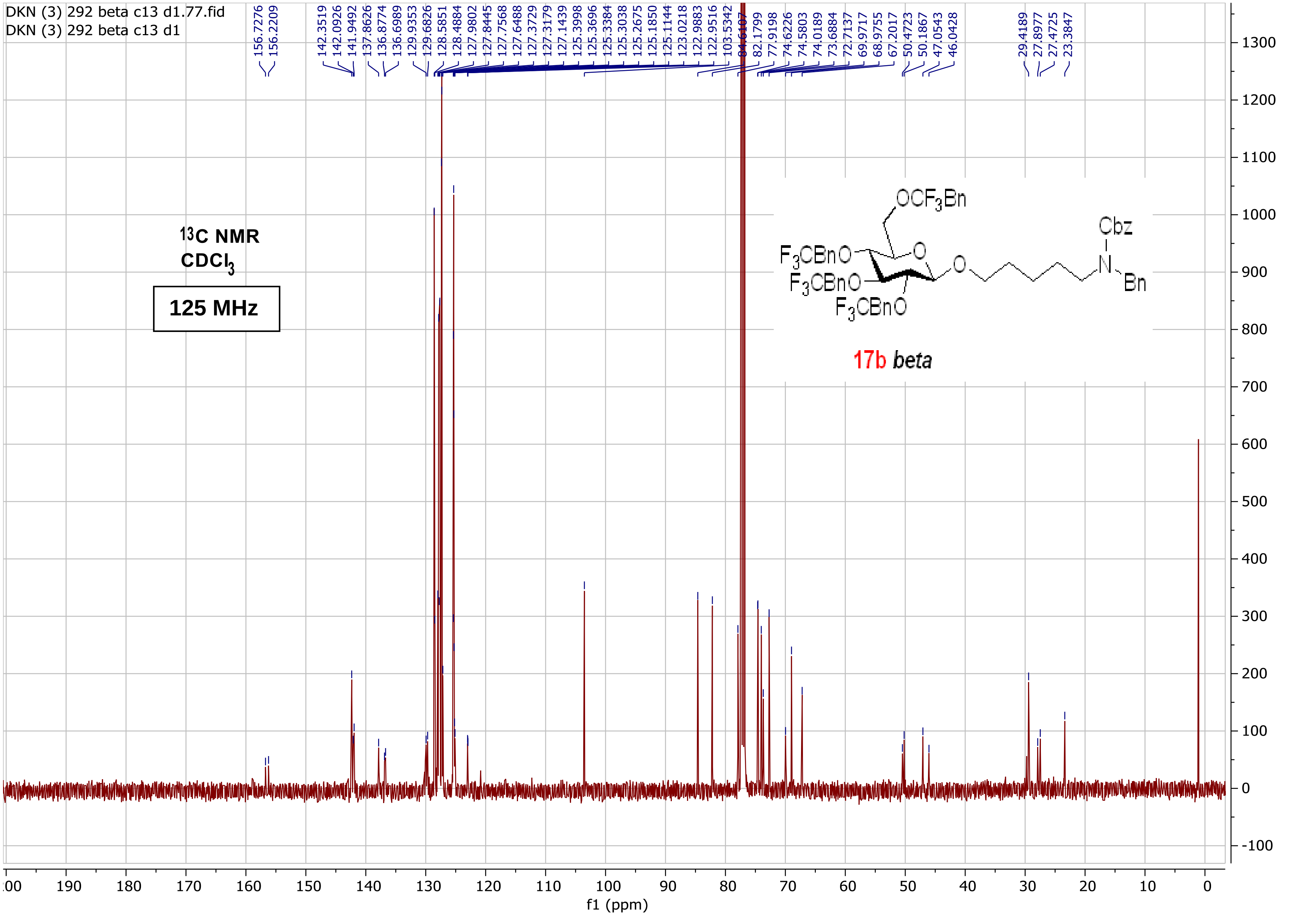

DKN (3) 292 beta c13 d1.77.fid
DKN (3) 292 beta c13 d1
156.7276
156.2209
142.3519
142.0926
141.9492
137.8626
136.8774
136.6989
129.9353
129.6826
128.5851
128.4884
127.9802
127.8445
127.7568
127.6488
127.3729
127.3179
127.1439
125.3998
125.3696
125.3384
125.3038
125.2675
125.1850
125.1144
123.0218
122.9883
122.9516
103.5342
84.6107
82.1799
77.9198
74.6226
74.5803
74.0189
73.6884
72.7137
69.9717
68.9755
67.2017
50.4723
50.1867
47.0543
46.0428
29.4189
27.8977
27.4725
23.3847
^{13}C NMR
$CDCl_3$
125 MHz
17b beta
f1 (ppm)

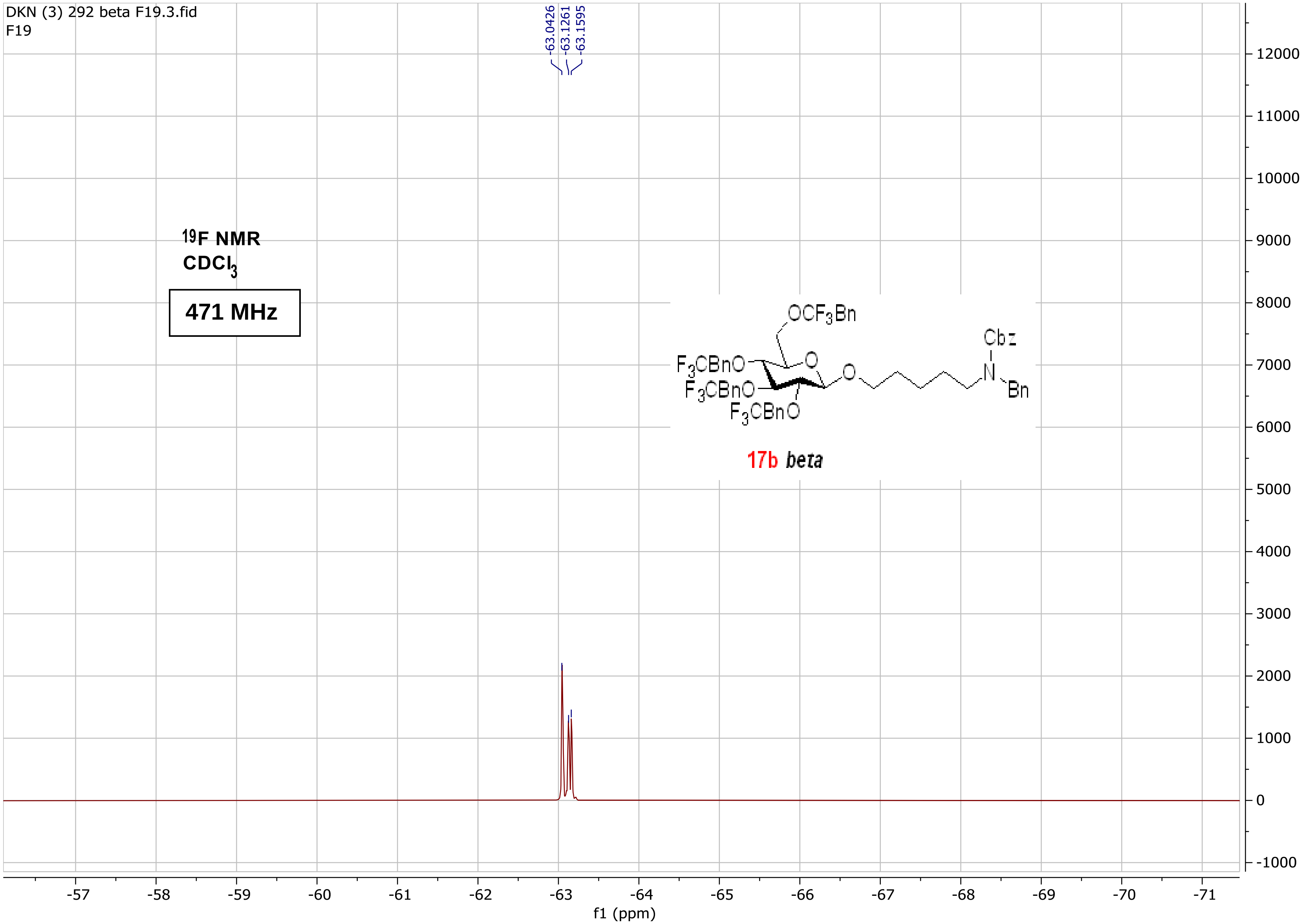
DKN (3) 292 beta F19.3.fid
F19
-63.0426
-63.1261
-63.1595
^{19}F NMR
$CDCl_3$
471 MHz
OCF3Bn
F3CBnO
F3CBnO
F3CBnO
O
O
Cbz
N
Bn
17b beta
f1 (ppm)

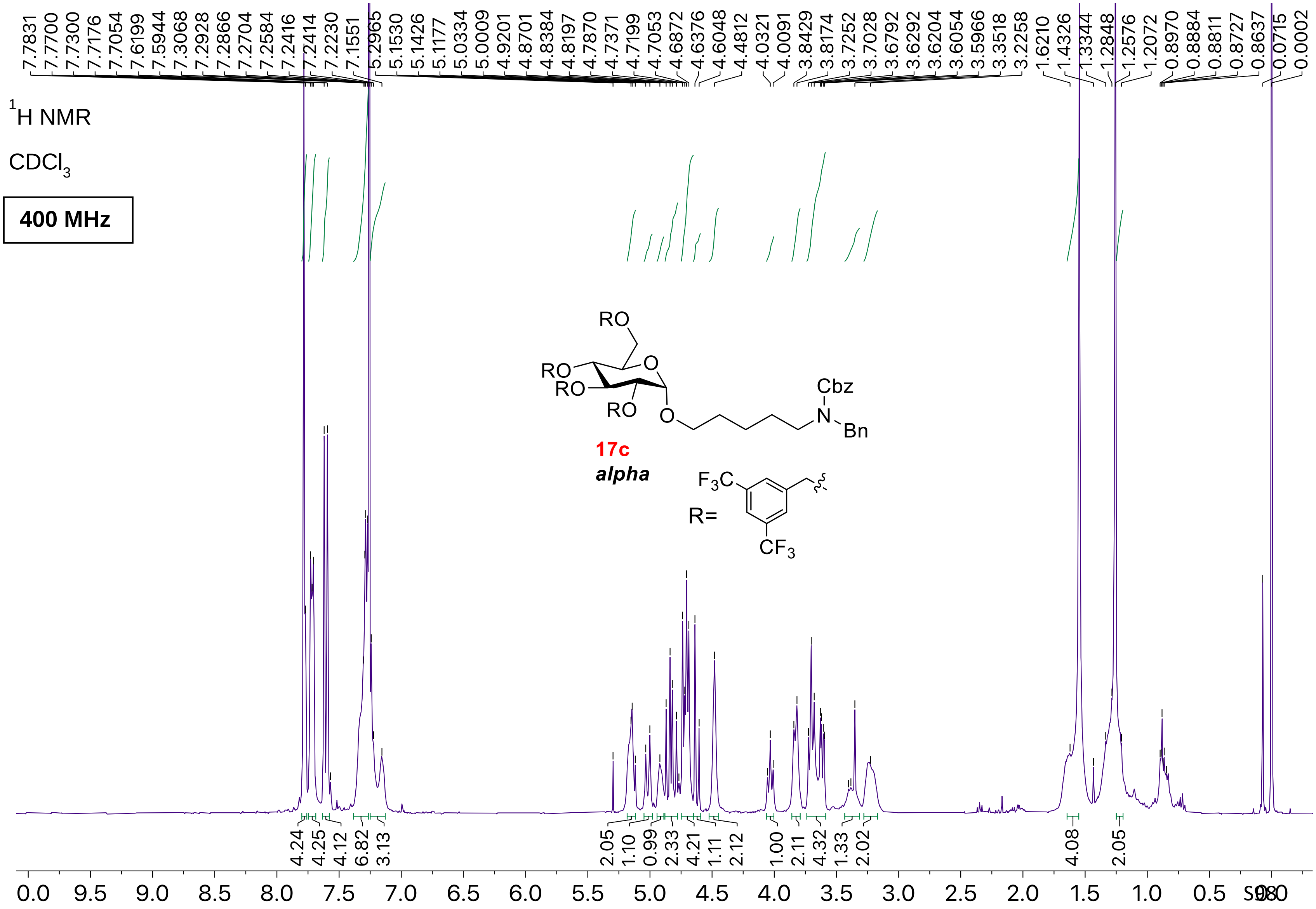

^{1}H NMR
$CDCl_3$
400 MHz
17c
alpha
R=
Cbz
Bn

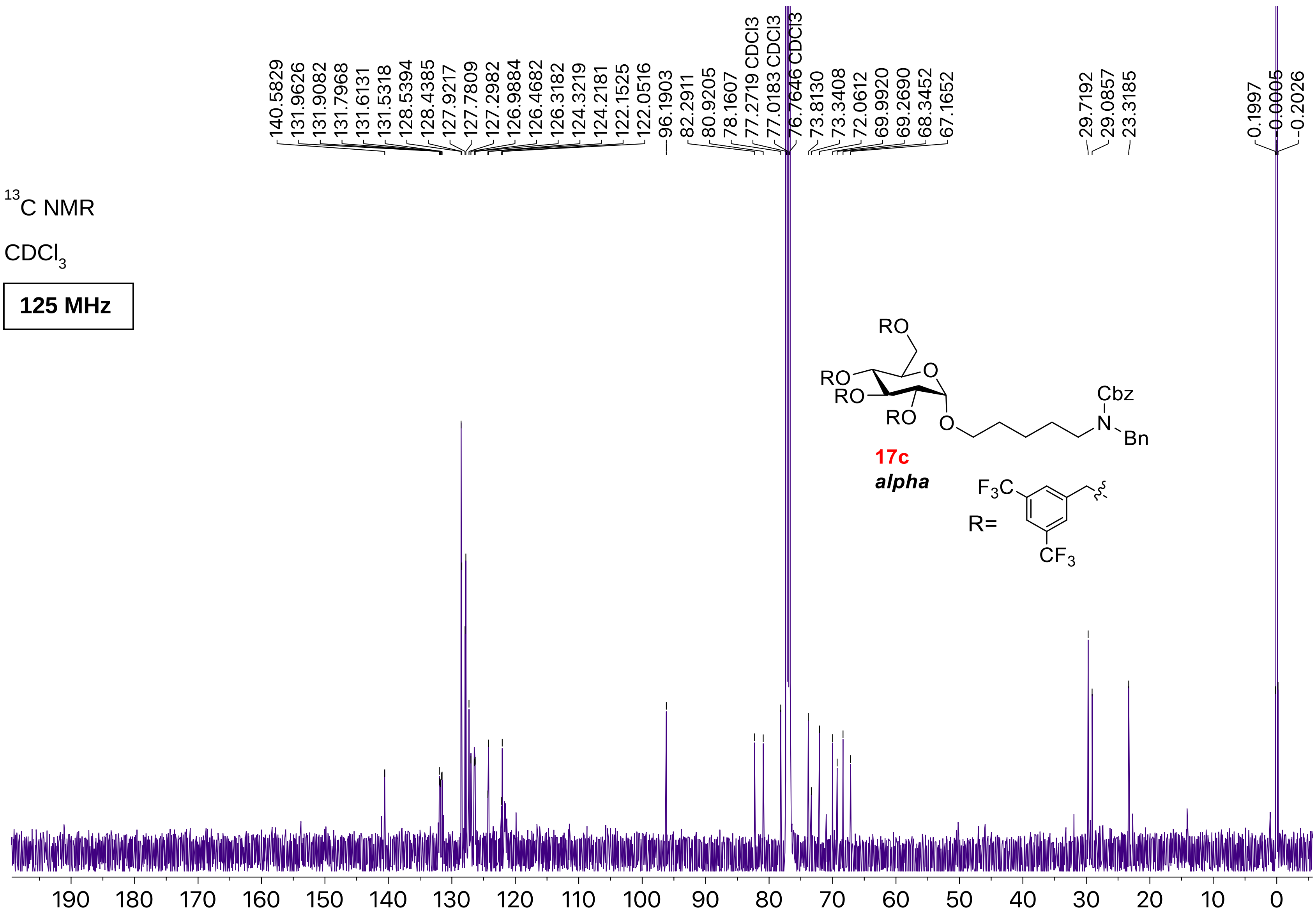

13C NMR
CDCl3
125 MHz
140.5829
131.9626
131.9082
131.7968
131.6131
131.5318
128.5394
128.4385
127.9217
127.7809
127.2982
126.9884
126.4682
126.3182
124.3219
124.2181
122.1525
122.0516
96.1903
82.2911
80.9205
78.1607
77.2719 CDCl3
77.0183 CDCl3
76.7646 CDCl3
73.8130
73.3408
72.0612
69.9920
69.2690
68.3452
67.1652
29.7192
29.0857
23.3185
0.1997
-0.0005
-0.2026
RO
O
RO
RO
RO
O
Cbz
N
Bn
17c
alpha
F3C
R=
CF3
190
180
170
160
150
140
130
120
110
100
90
80
70
60
50
40
30
20
10
0

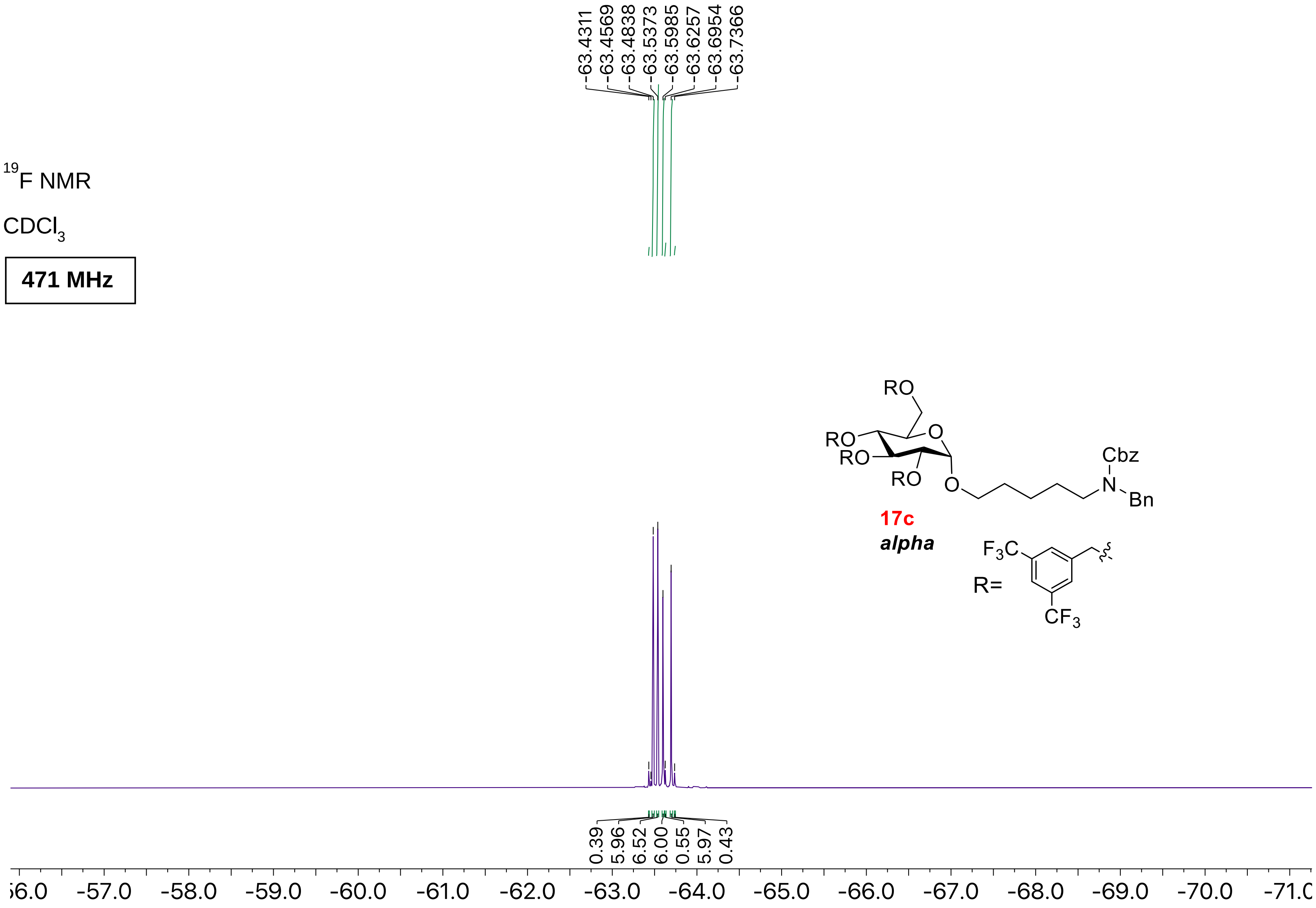

19F NMR
CDCl3
471 MHz
-63.4311
-63.4569
-63.4838
-63.5373
-63.5985
-63.6257
-63.6954
-63.7366
RO
RO
RO
RO
O
O
Cbz
N
Bn
17c
alpha
R=
F3C
CF3
0.39
5.96
6.52
6.00
0.55
5.97
0.43
-57.0
-58.0
-59.0
-60.0
-61.0
-62.0
-63.0
-64.0
-65.0
-66.0
-67.0
-68.0
-69.0
-70.0

^{1}H NMR

$CDCl_3$

500 MHz

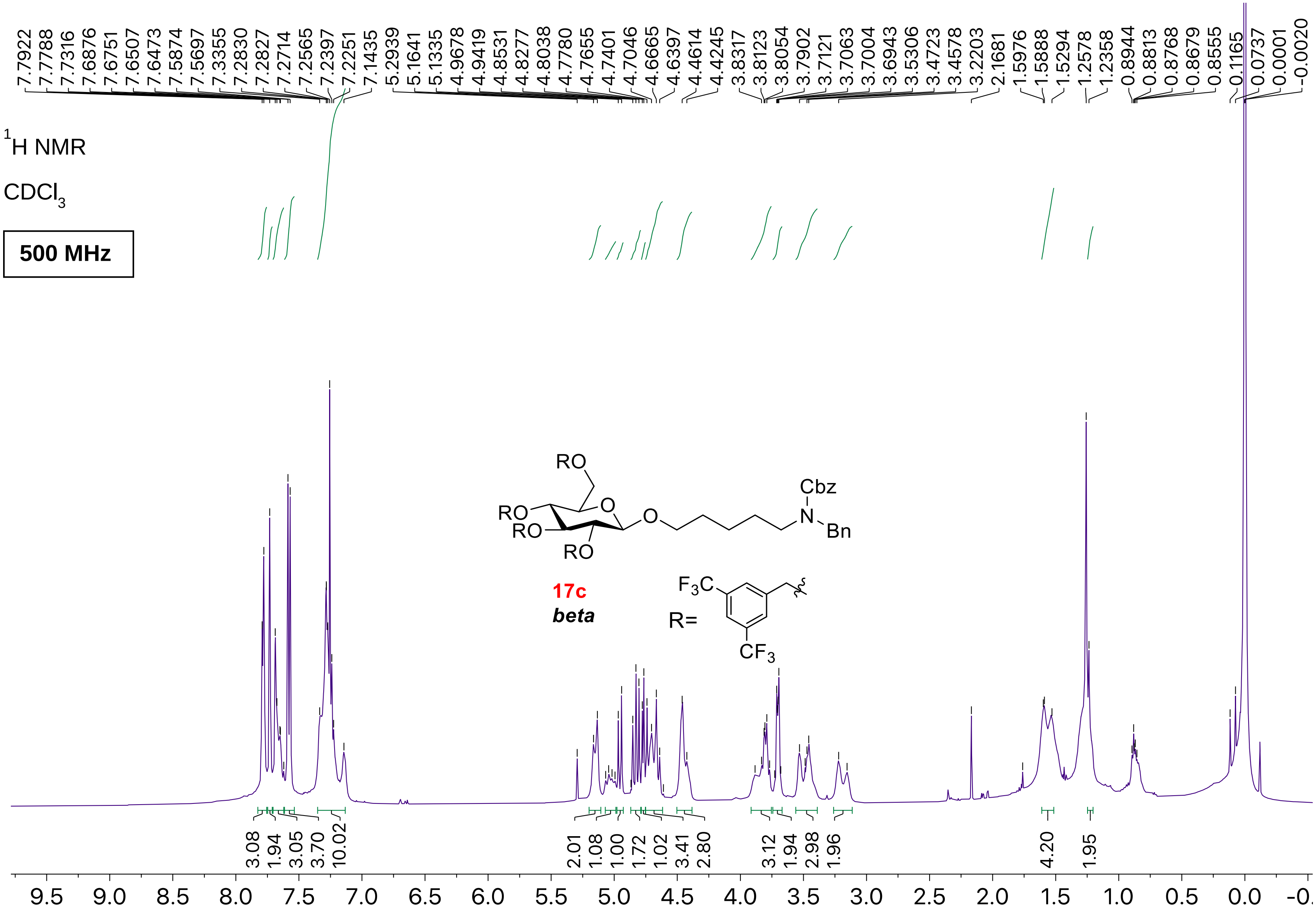

17c
beta

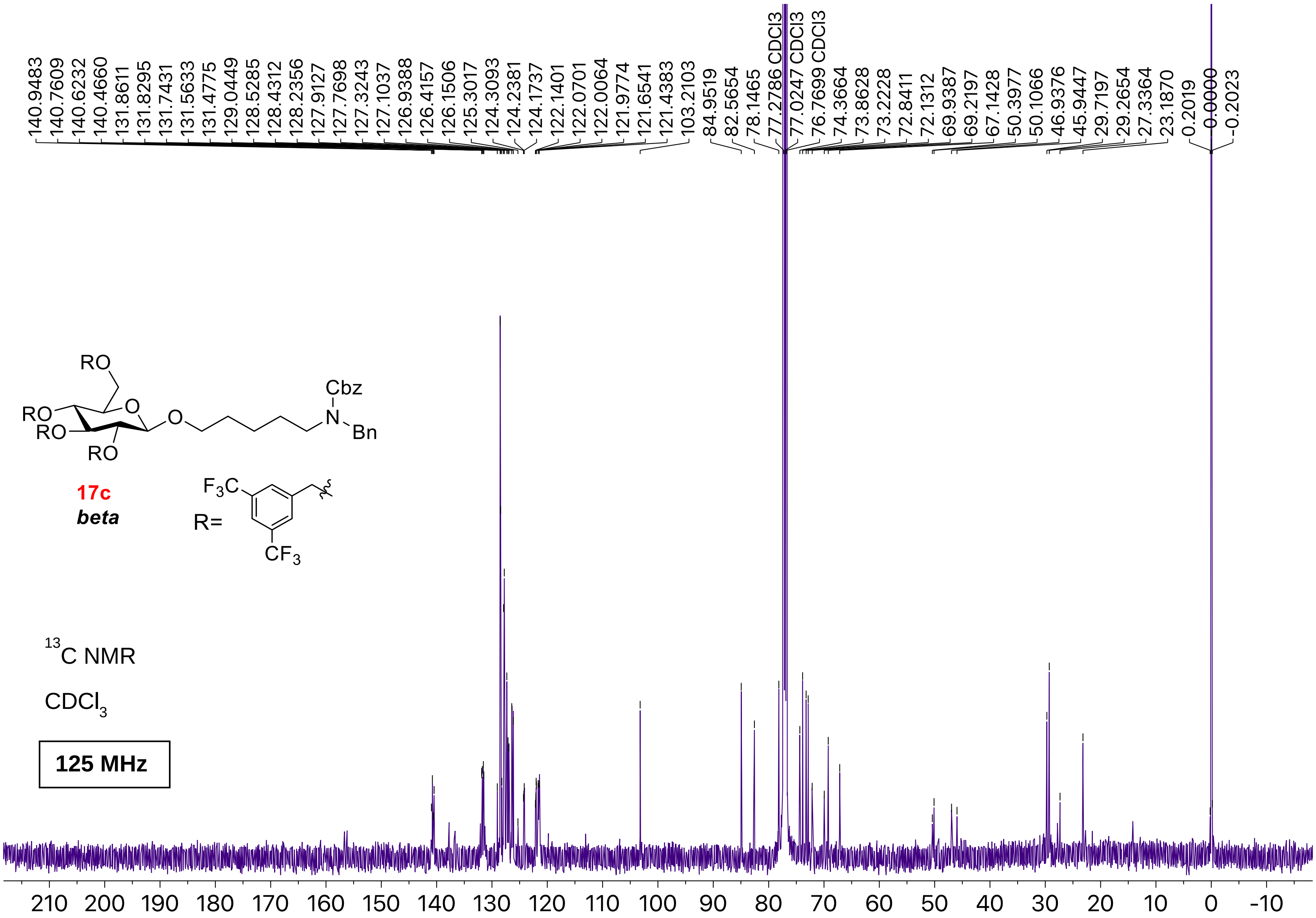
140.9483
140.7609
140.6232
140.4660
131.8611
131.8295
131.7431
131.5633
131.4775
129.0449
128.5285
128.4312
128.2356
127.9127
127.7698
127.3243
127.1037
126.9388
126.4157
126.1506
125.3017
124.3093
124.2381
124.1737
122.1401
122.0701
122.0064
121.9774
121.6541
121.4383
103.2103
84.9519
82.5654
78.1465
77.2786 CDCl3
77.0247 CDCl3
76.7699 CDCl3
74.3664
73.8628
73.2228
72.8411
72.1312
69.9387
69.2197
67.1428
50.3977
50.1066
46.9376
45.9447
29.7197
29.2654
27.3364
23.1870
0.2019
0.0000
-0.2023
RO
RO
RO
RO
O
O
Cbz
N
Bn
17c
beta
R=
F3C
CF3
13C NMR
CDCl3
125 MHz
210 200 190 180 170 160 150 140 130 120 110 100 90 80 70 60 50 40 30 20 10 0 -10

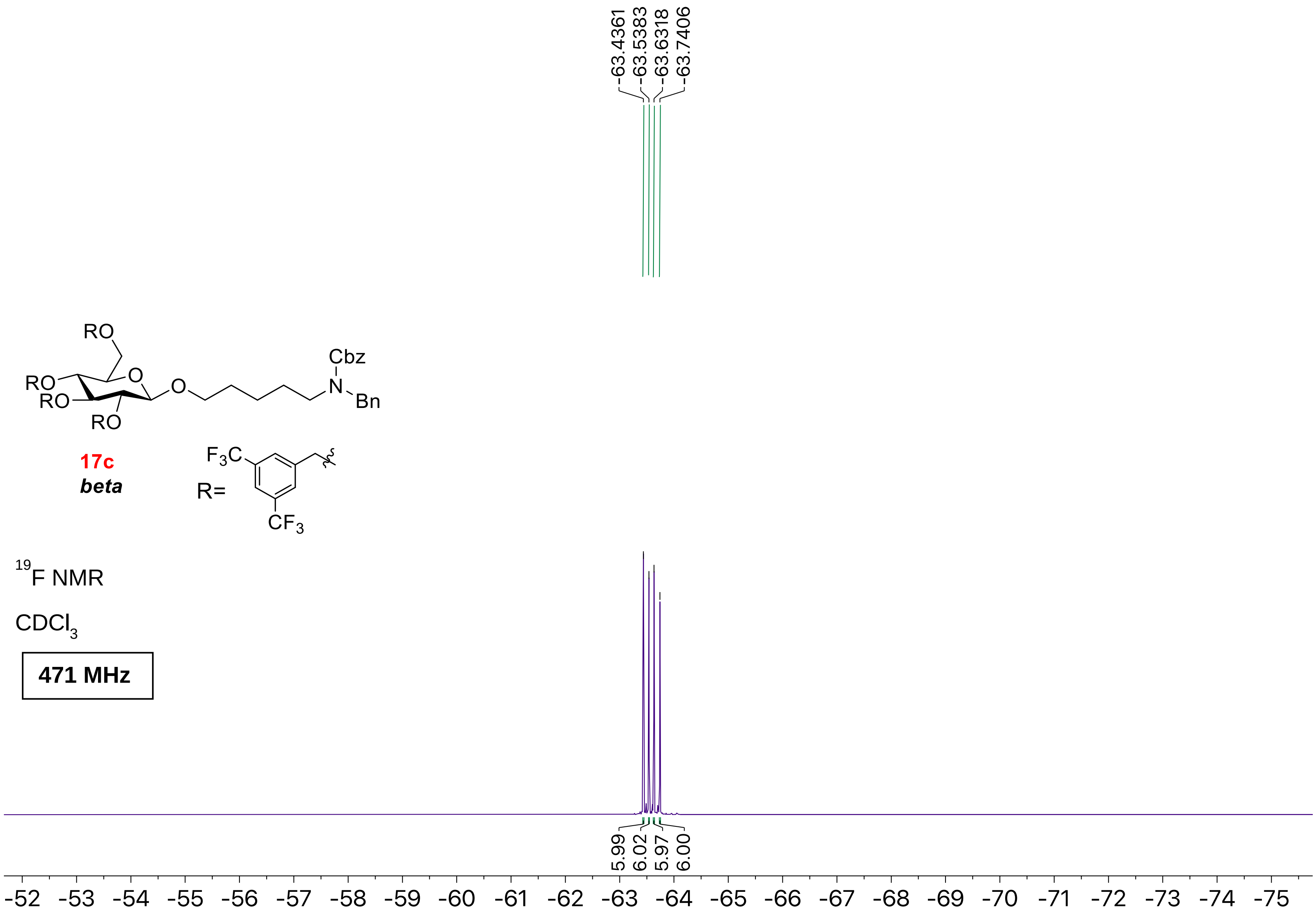

17c
beta

^{19}F NMR

$CDCl_3$

471 MHz

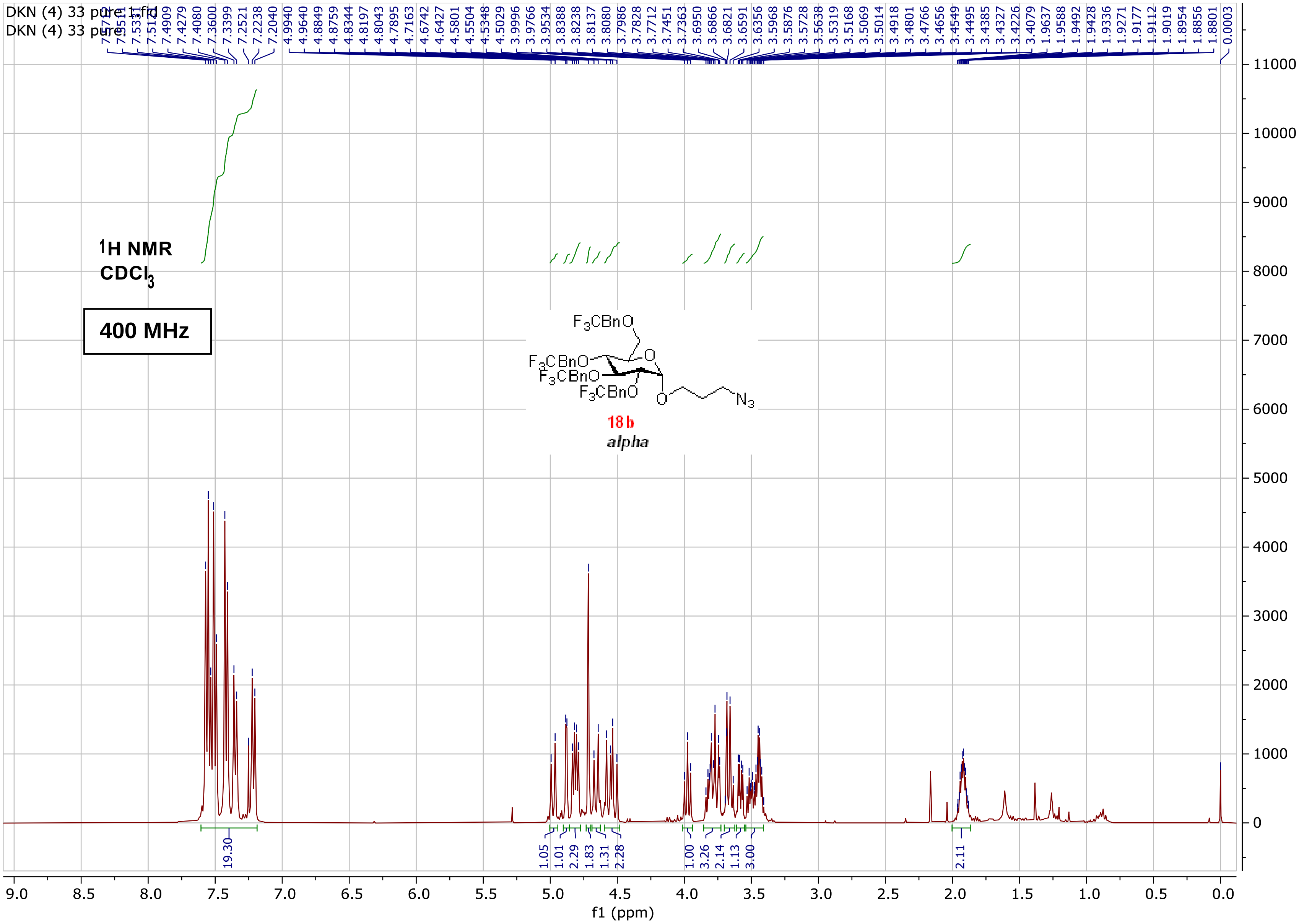

DKN (4) 33 pure 1.fid
DKN (4) 33 pure
1H NMR
CDCl3
400 MHz
F3CBnO
F3CBnO
F3CBnO
F3CBnO
O
O
N3
18b
alpha
f1 (ppm)

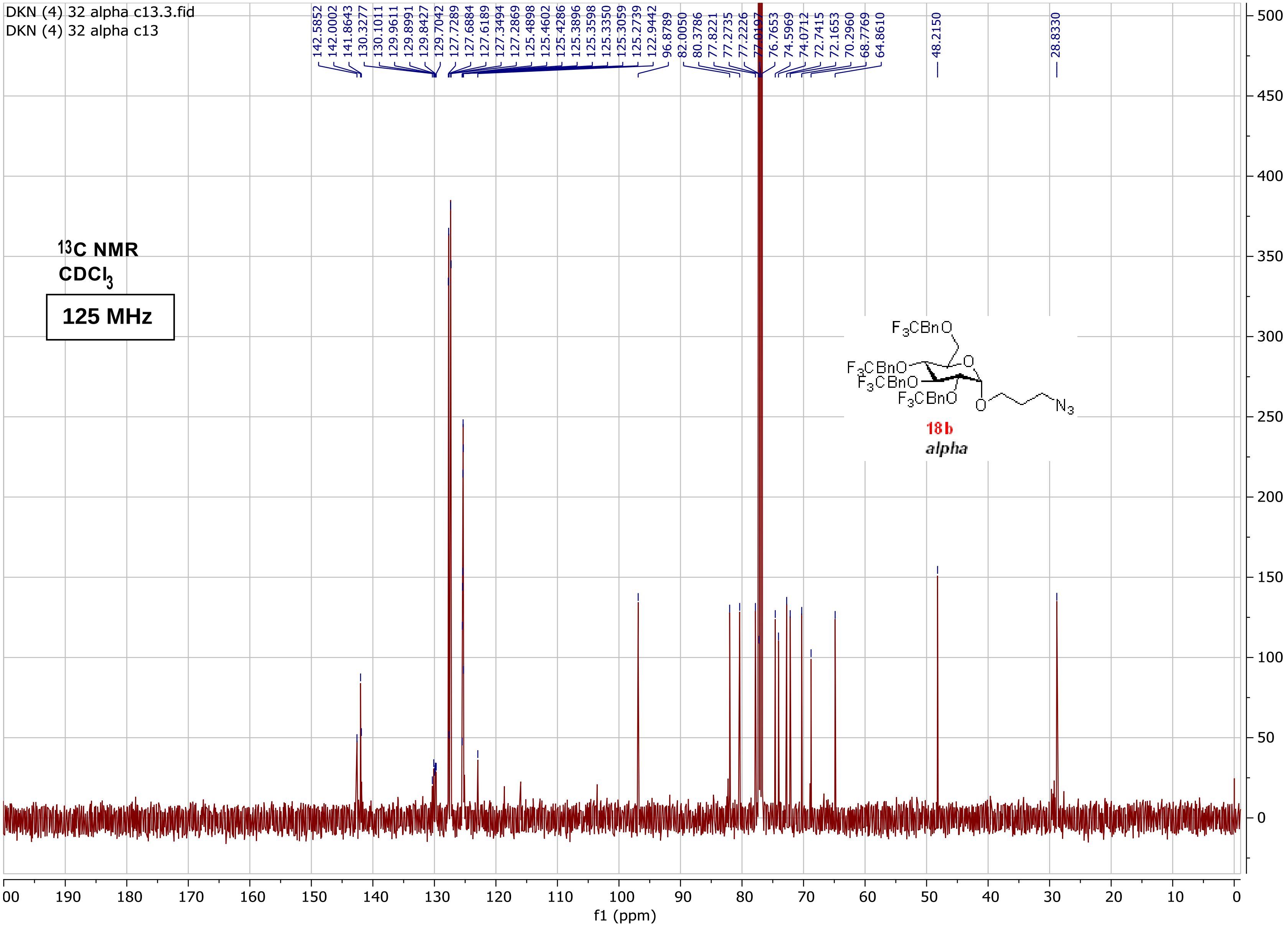
DKN (4) 32 alpha c13.3.fid
DKN (4) 32 alpha c13
142.5852
142.0002
141.8643
130.3277
130.1011
129.9611
129.8991
129.8427
129.7042
127.7289
127.6884
127.6189
127.3494
127.2869
125.4898
125.4602
125.4286
125.3896
125.3598
125.3350
125.3059
125.2739
122.9442
96.8789
82.0050
80.3786
77.8221
77.2735
77.2226
76.7653
74.5969
74.0712
72.7415
72.1653
70.2960
68.7769
64.8610
48.2150
28.8330
^{13}C NMR
$CDCl_3$
125 MHz
F_3CBnO
F_3CBnO
F_3CBnO
F_3CBnO
O
O
N_3
18b
alpha
f1 (ppm)

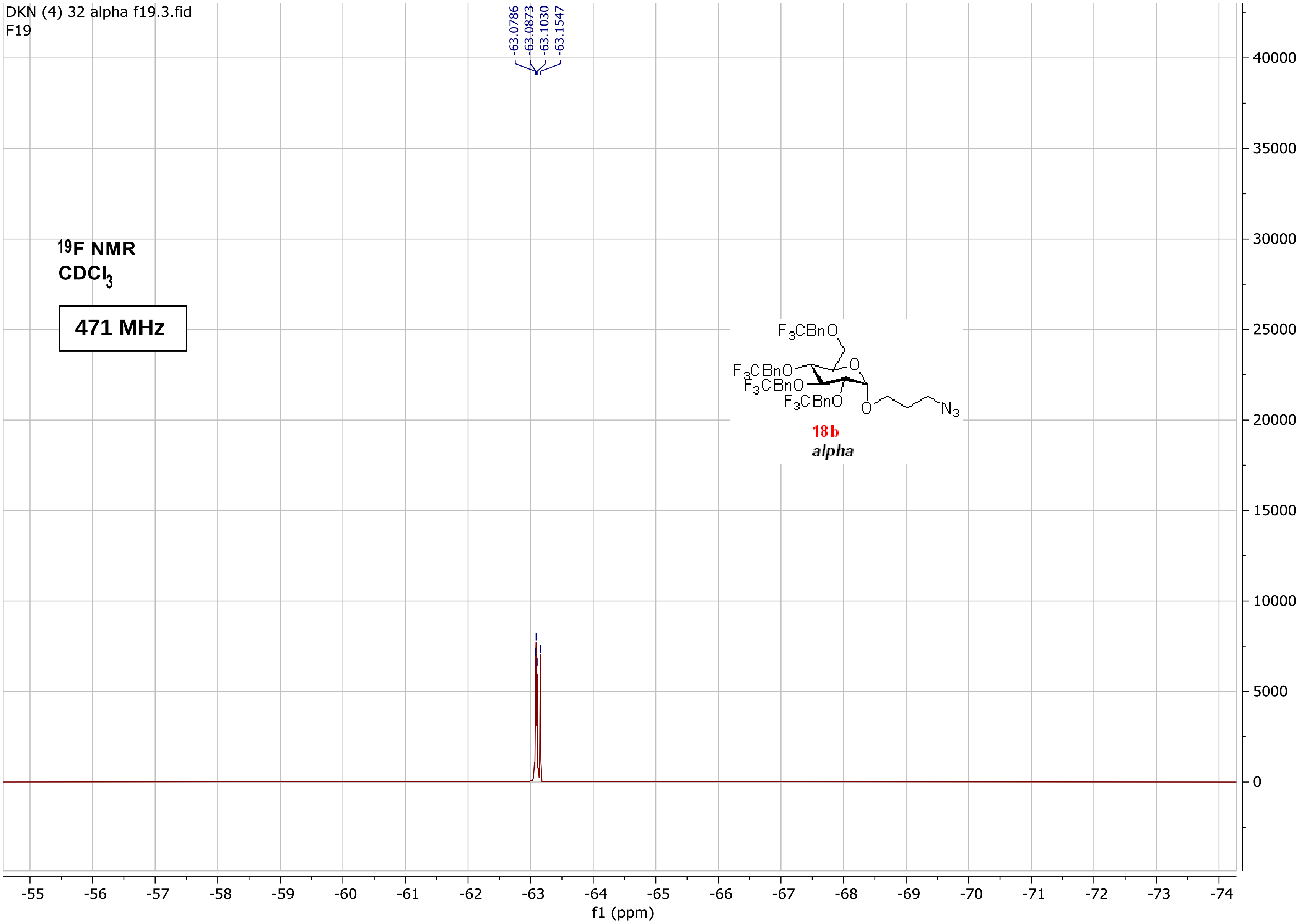

DKN (4) 32 alpha f19.3.fid
F19
-63.0786
-63.0873
-63.1030
-63.1547
19F NMR
CDCl3
471 MHz
F3CBnO
F3CBnO
F3CBnO
F3CBnO
O
O
N3
18b
alpha
40000
35000
30000
25000
20000
15000
10000
5000
0
-55
-56
-57
-58
-59
-60
-61
-62
-63
-64
-65
-66
-67
-68
-69
-70
-71
-72
-73
-74
f1 (ppm)

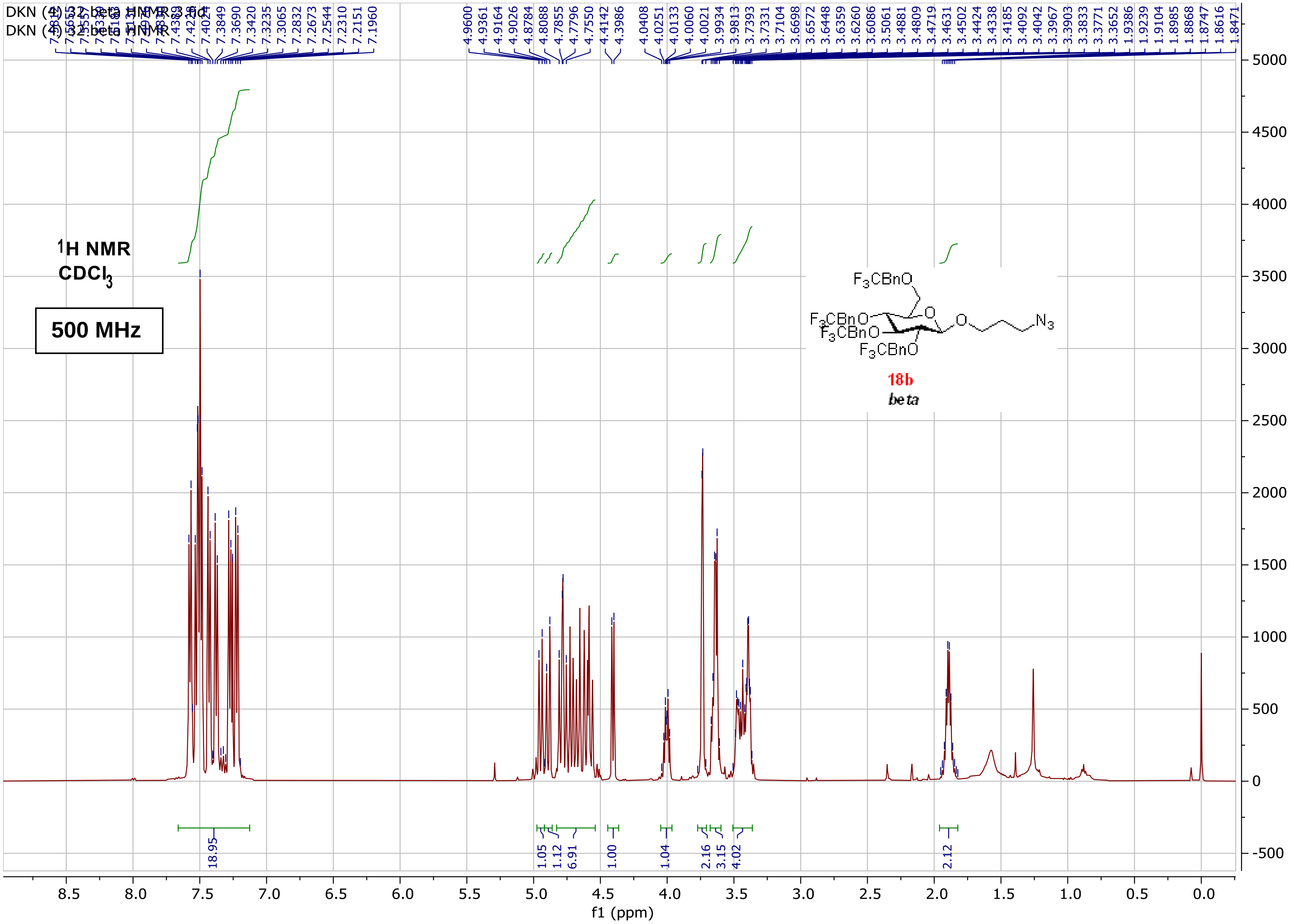

DKN (4) 32 beta HNMR 3.fid
DKN (4) 32 beta HNMR
1H NMR
CDCl3
500 MHz
F3CBnO
O
O
N3
F3CBnO
F3CBnO
F3CBnO
18b
beta
f1 (ppm)

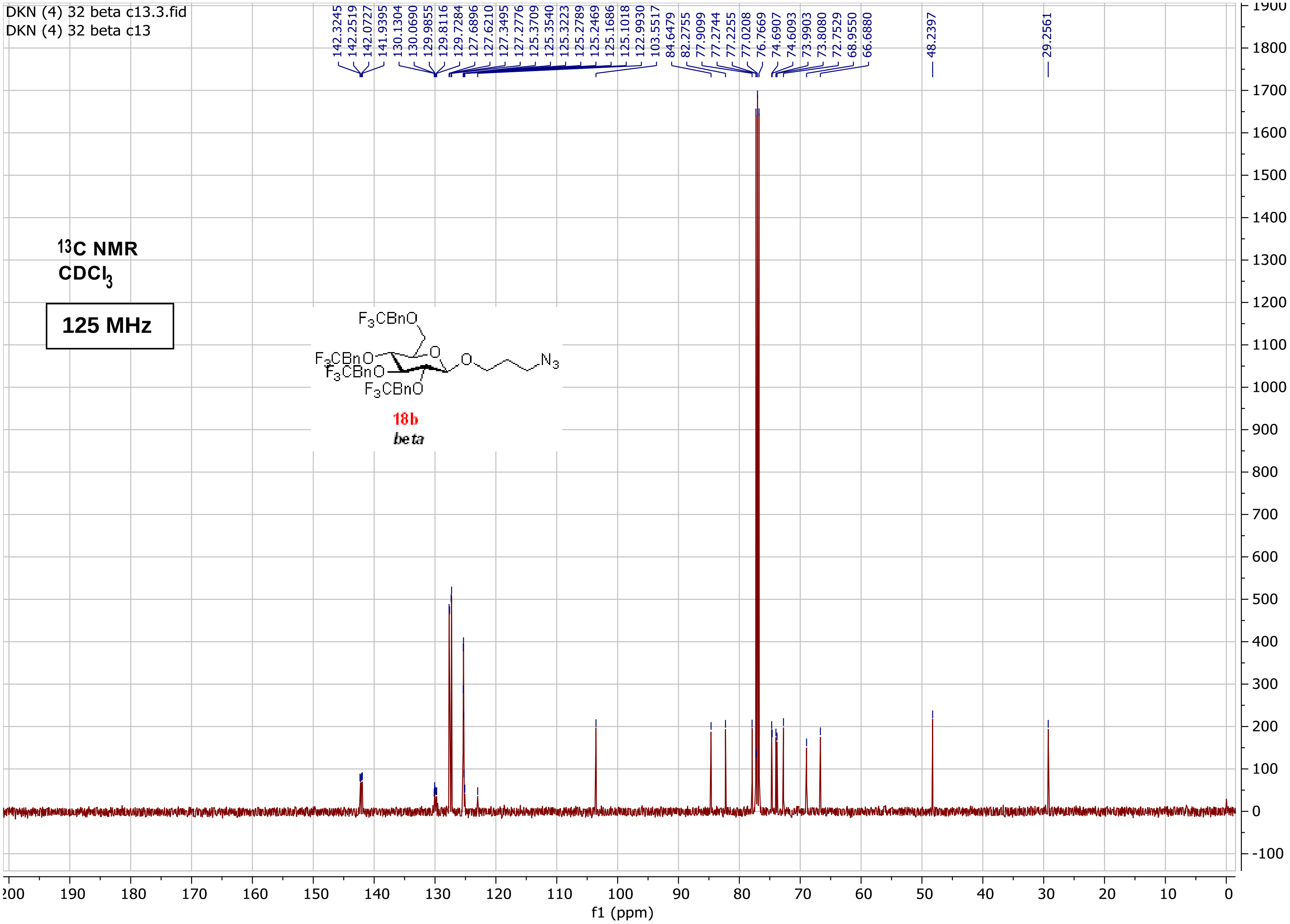
DKN (4) 32 beta c13.3.fid
DKN (4) 32 beta c13
142.3245
142.2519
142.0727
141.9395
130.1304
130.0690
129.9855
129.8116
129.7284
127.6896
127.6210
127.3495
127.2776
125.3709
125.3540
125.3223
125.2789
125.2469
125.1686
125.1018
122.9930
103.5517
84.6479
82.2755
77.9099
77.2744
77.2255
77.0208
76.7669
74.6907
74.6093
73.9903
73.8080
72.7529
68.9550
66.6880
48.2397
29.2561
13C NMR
CDCl3
125 MHz
F3CBnO
F3CBnO
F3CBnO
F3CBnO
O
O
N3
18b
beta
f1 (ppm)

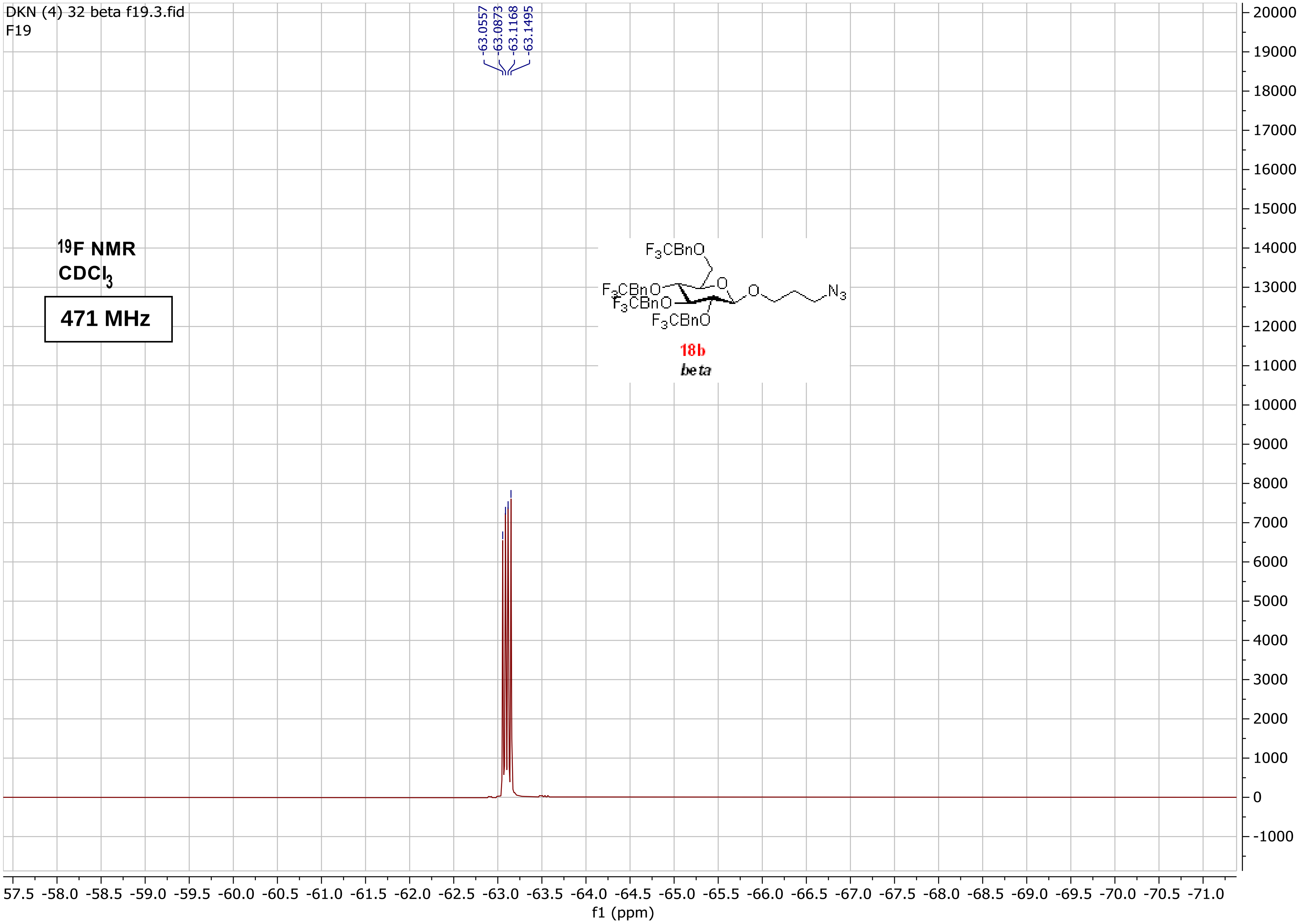

DKN (4) 32 beta f19.3.fid
F19
-63.0557
-63.0873
-63.1168
-63.1495
19F NMR
CDCl3
471 MHz
F3CBnO
F3CBnO
F3CBnO
F3CBnO
O
O
N3
18b
beta
f1 (ppm)

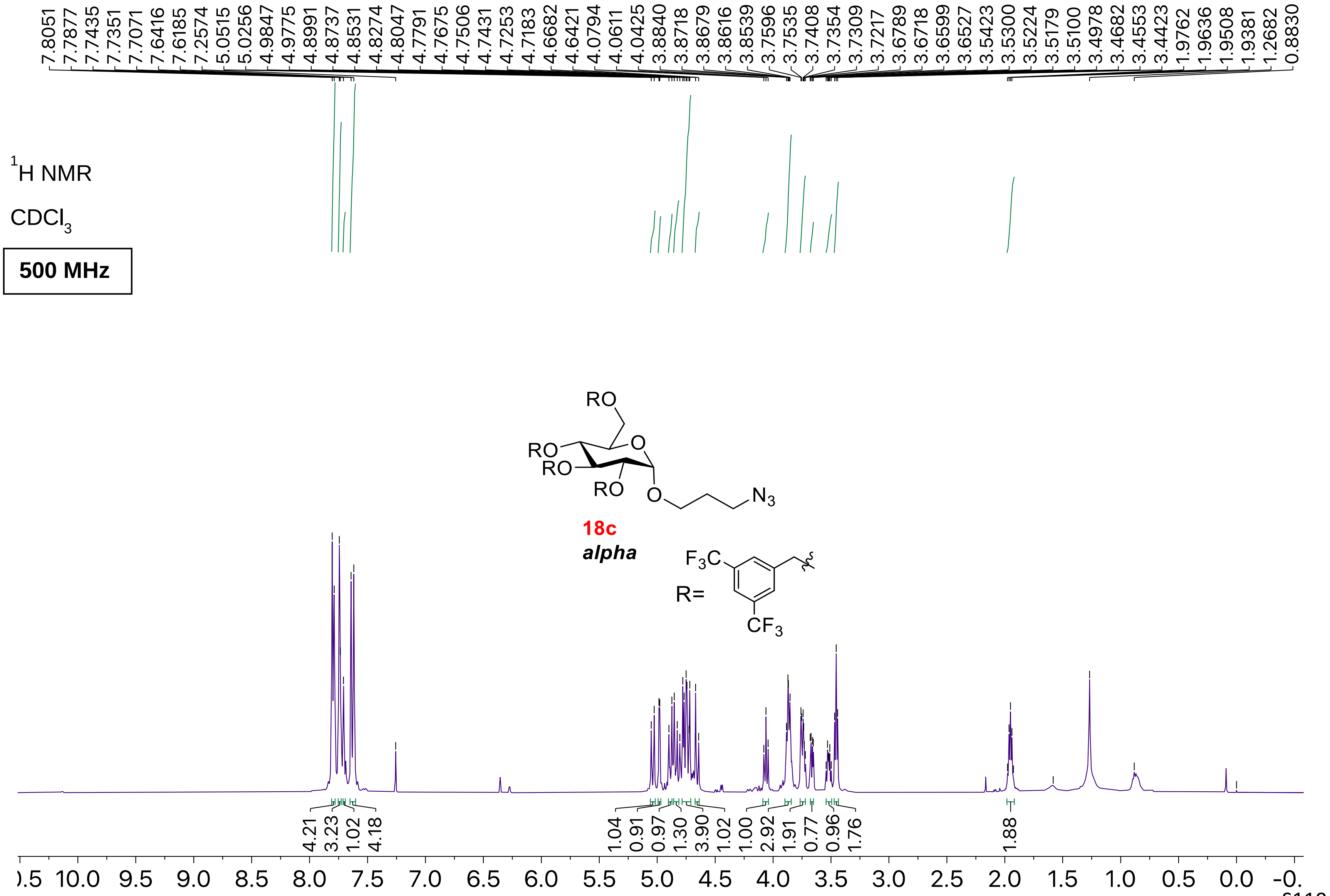

1H NMR
CDCl3
500 MHz
18c
alpha
R=

^{13}C NMR

$CDCl_3$

125 MHz

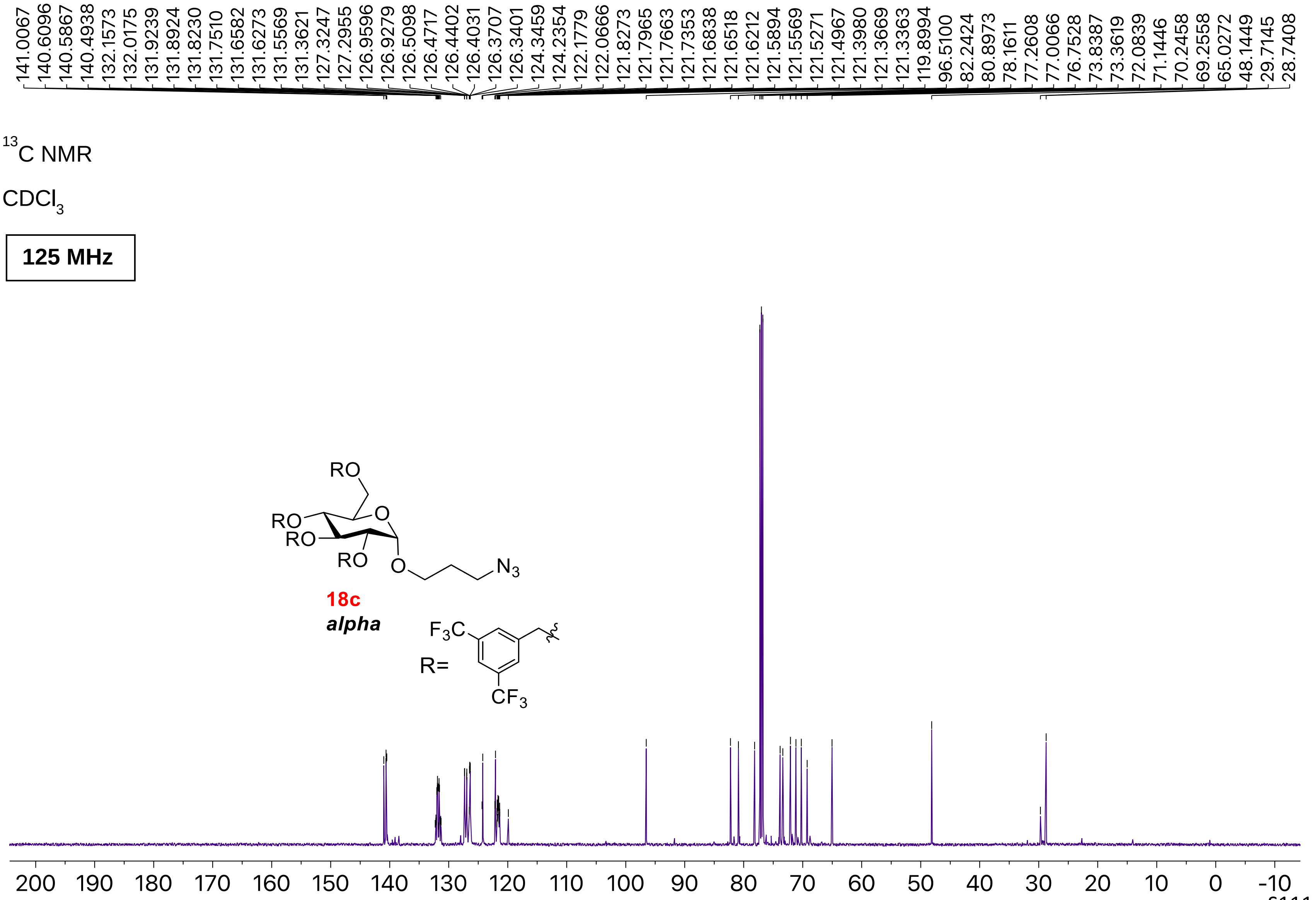

^{19}F NMR

$CDCl_3$

471 MHz

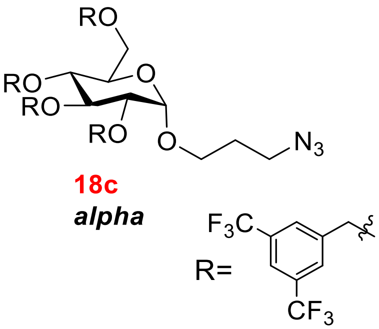

18c

alpha

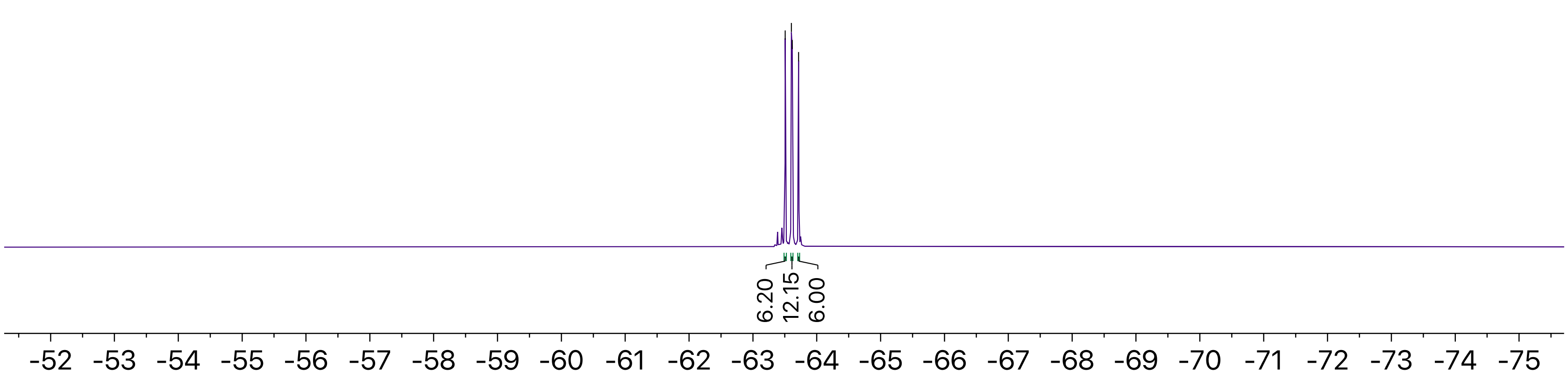

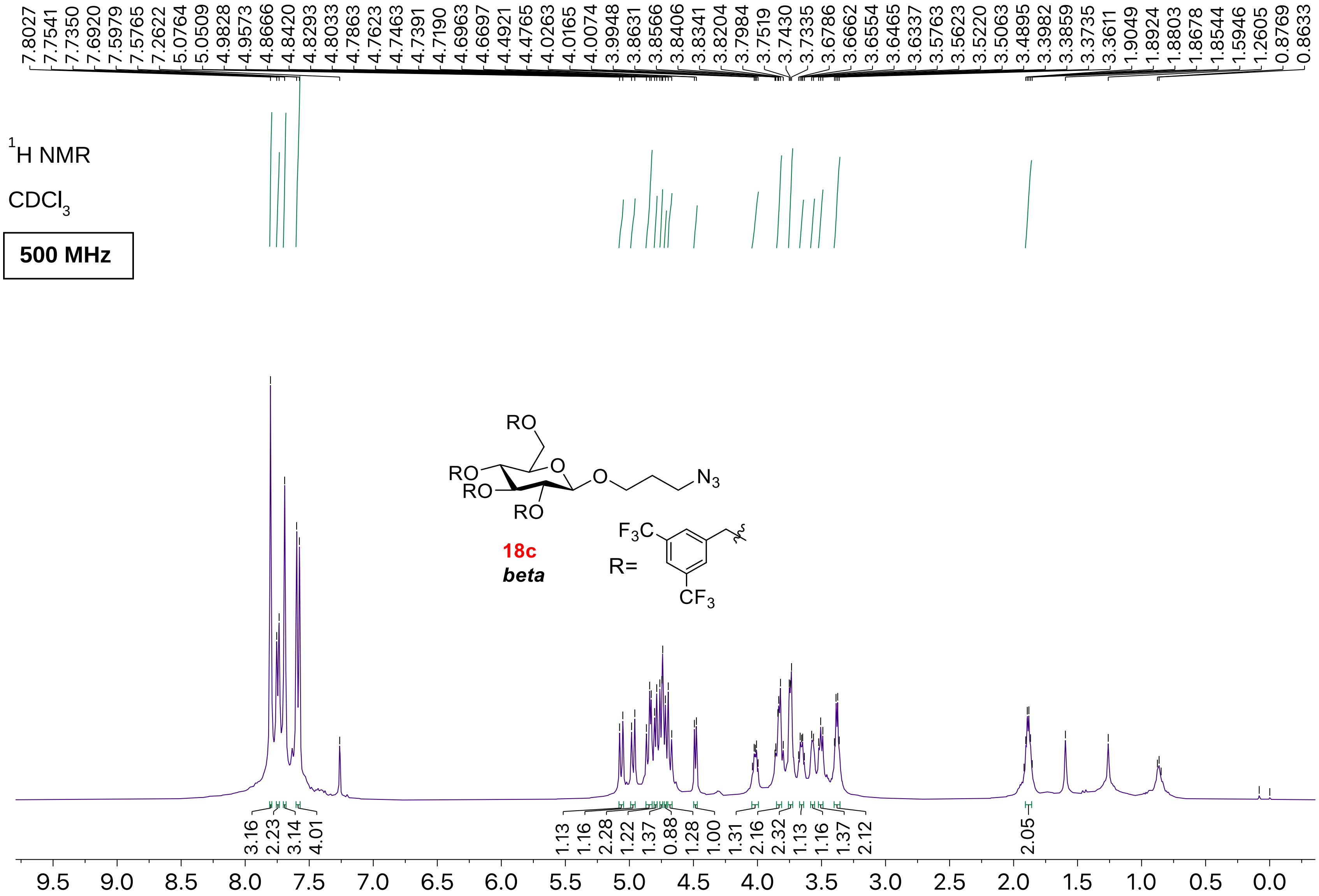
7.8027
7.7541
7.7350
7.6920
7.5979
7.5765
7.2622
5.0764
5.0509
4.9828
4.9573
4.8666
4.8420
4.8293
4.8033
4.7863
4.7623
4.7463
4.7391
4.7190
4.6963
4.6697
4.4921
4.4765
4.0263
4.0165
4.0074
3.9948
3.8631
3.8566
3.8406
3.8341
3.8204
3.7984
3.7519
3.7430
3.7335
3.6786
3.6662
3.6554
3.6465
3.6337
3.5763
3.5623
3.5220
3.5063
3.4895
3.3982
3.3859
3.3735
3.3611
1.9049
1.8924
1.8803
1.8678
1.8544
1.5946
1.2605
0.8769
0.8633
^{1}H NMR
$CDCl_3$
500 MHz
RO
RO
RO
O
O
N_3
RO
18c
beta
F_3C
R=
CF_3
3.16
2.23
3.14
4.01
1.13
1.16
2.28
1.22
1.37
0.88
1.28
1.00
1.31
2.16
2.32
1.13
1.16
1.37
2.12
2.05
9.5
9.0
8.5
8.0
7.5
7.0
6.5
6.0
5.5
5.0
4.5
4.0
3.5
3.0
2.5
2.0
1.5
1.0
0.5
0.0

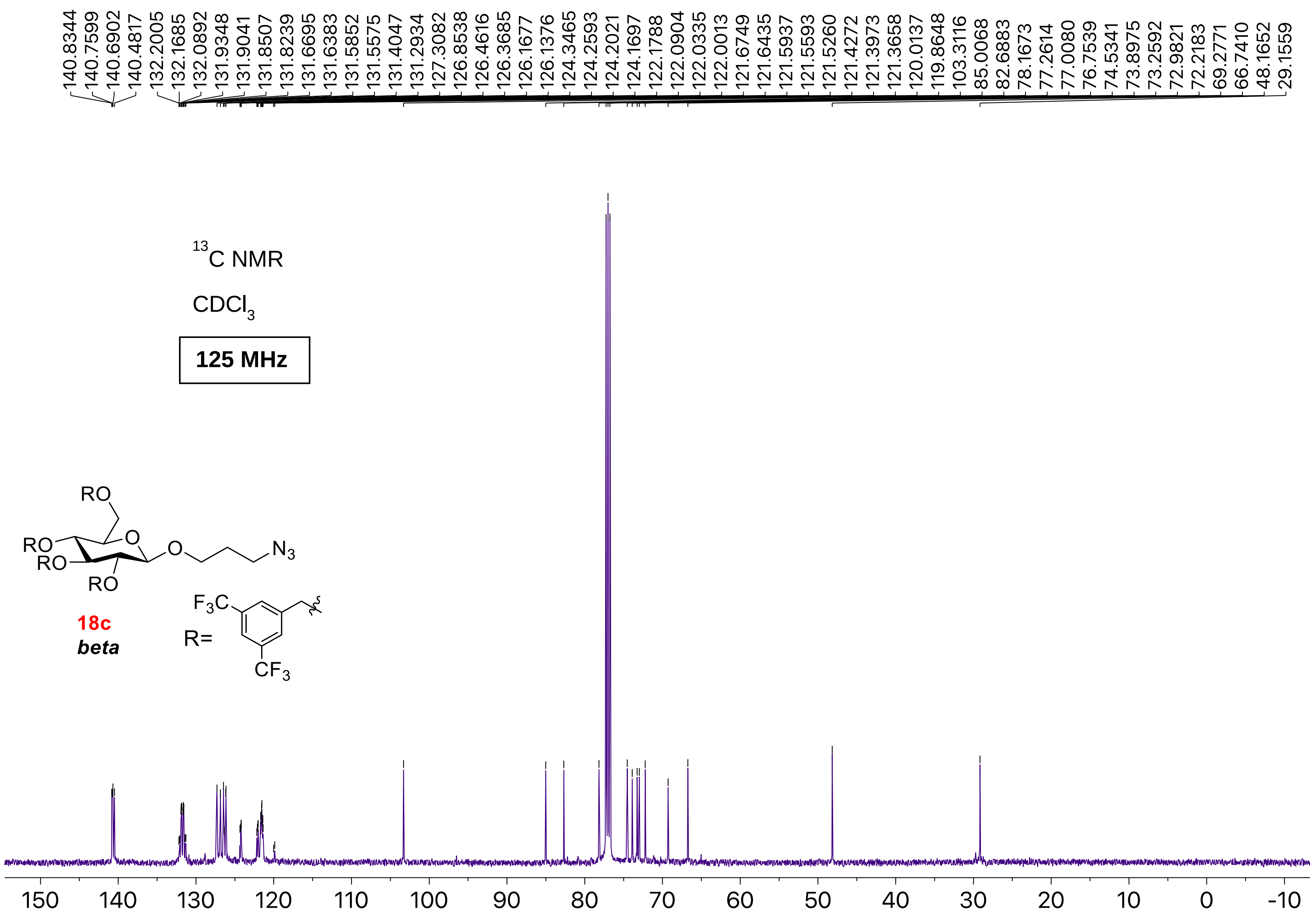
140.8344
140.7599
140.6902
140.4817
132.2005
132.1685
132.0892
131.9348
131.9041
131.8507
131.8239
131.6695
131.6383
131.5852
131.5575
131.4047
131.2934
127.3082
126.8538
126.4616
126.3685
126.1677
126.1376
124.3465
124.2593
124.2021
124.1697
122.1788
122.0904
122.0335
122.0013
121.6749
121.6435
121.5937
121.5593
121.5260
121.4272
121.3973
121.3658
120.0137
119.8648
103.3116
85.0068
82.6883
78.1673
77.2614
77.0080
76.7539
74.5341
73.8975
73.2592
72.9821
72.2183
69.2771
66.7410
48.1652
29.1559
13C NMR
CDCl3
125 MHz
RO
O
N3
F3C
R=
CF3
18c
beta
150
140
130
120
110
100
90
80
70
60
50
40
30
20
10
0
-10

^{19}F NMR

$CDCl_3$

471 MHz

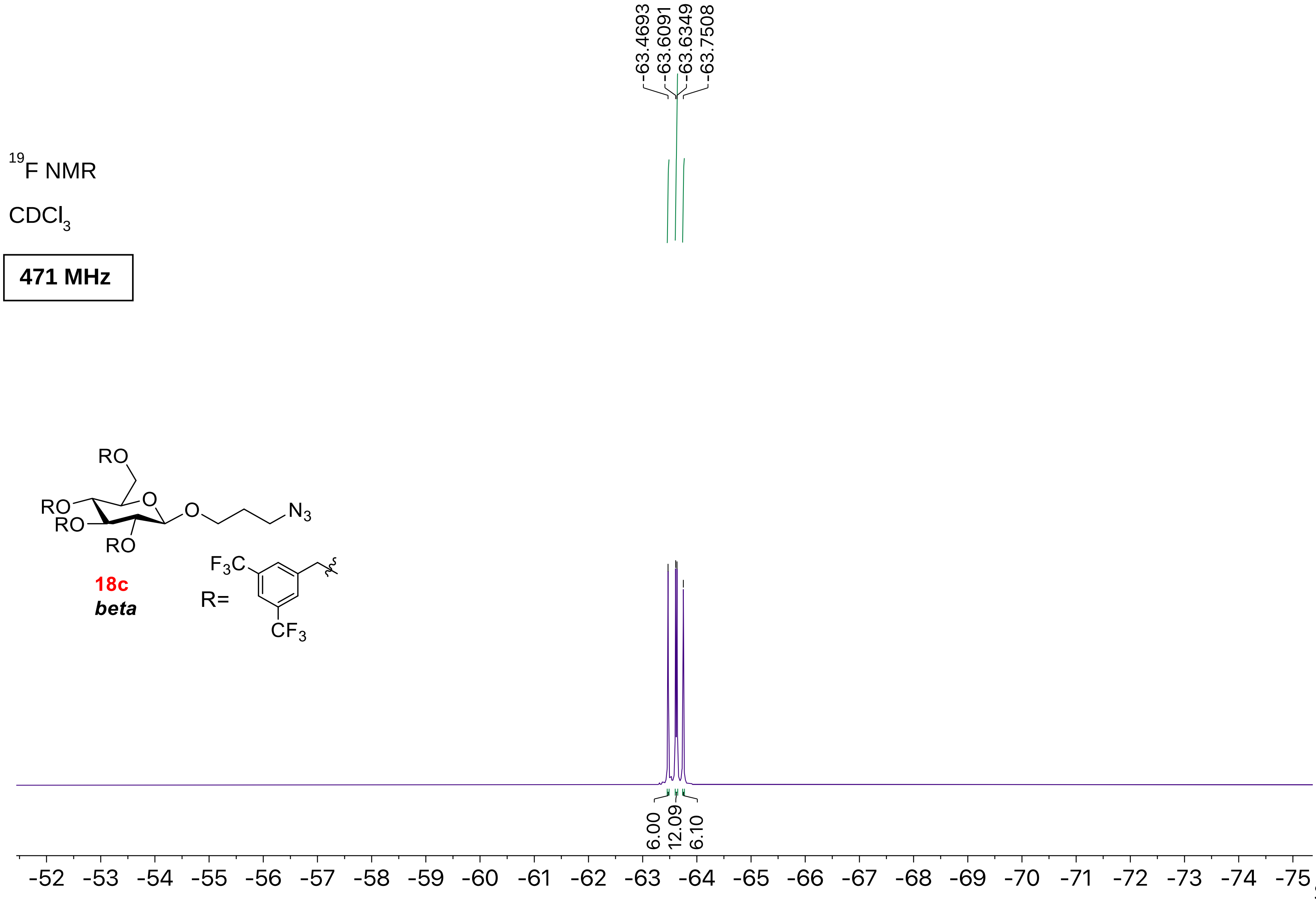

18c
beta

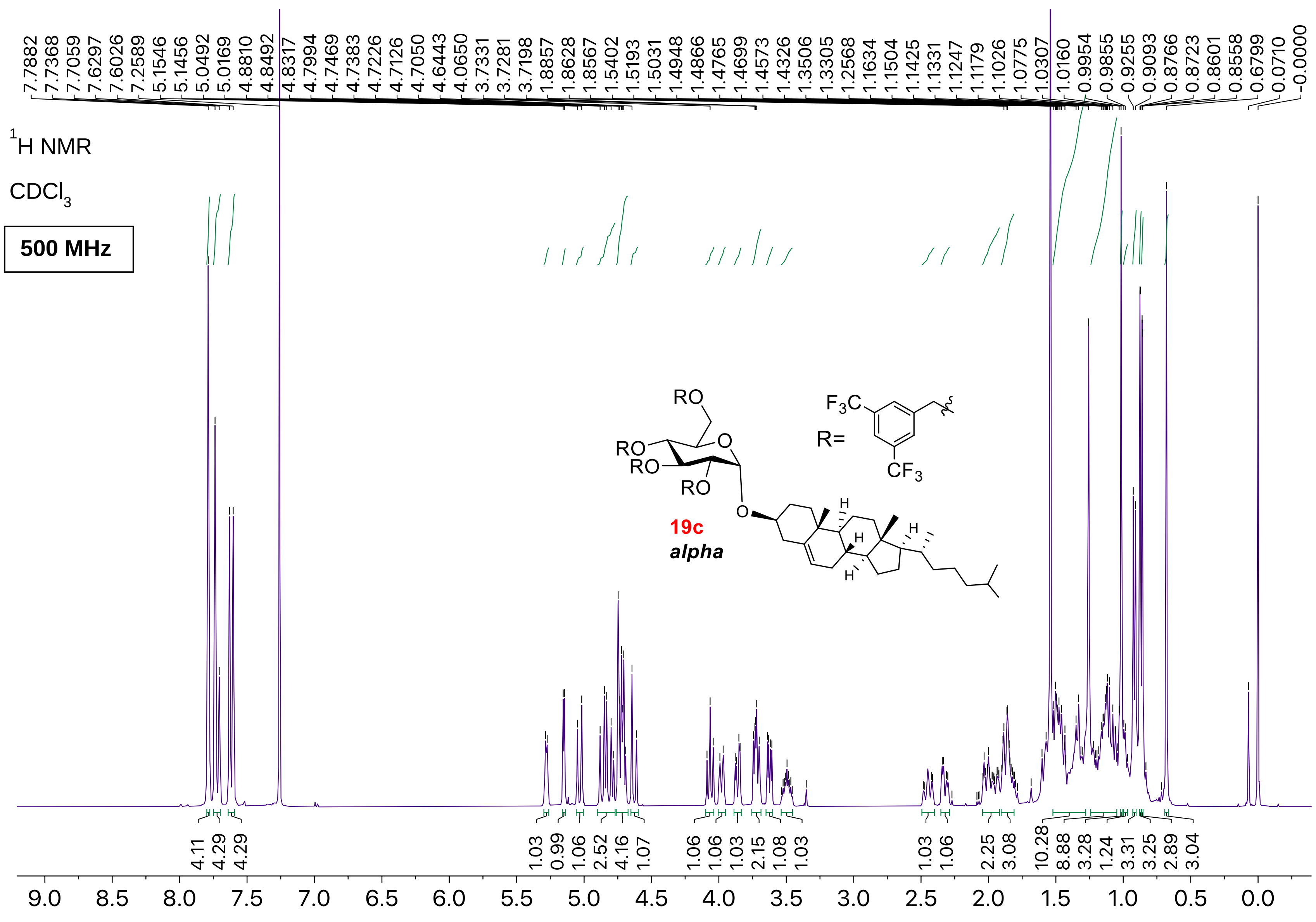

1H NMR
CDCl3
500 MHz
19c
alpha
R=

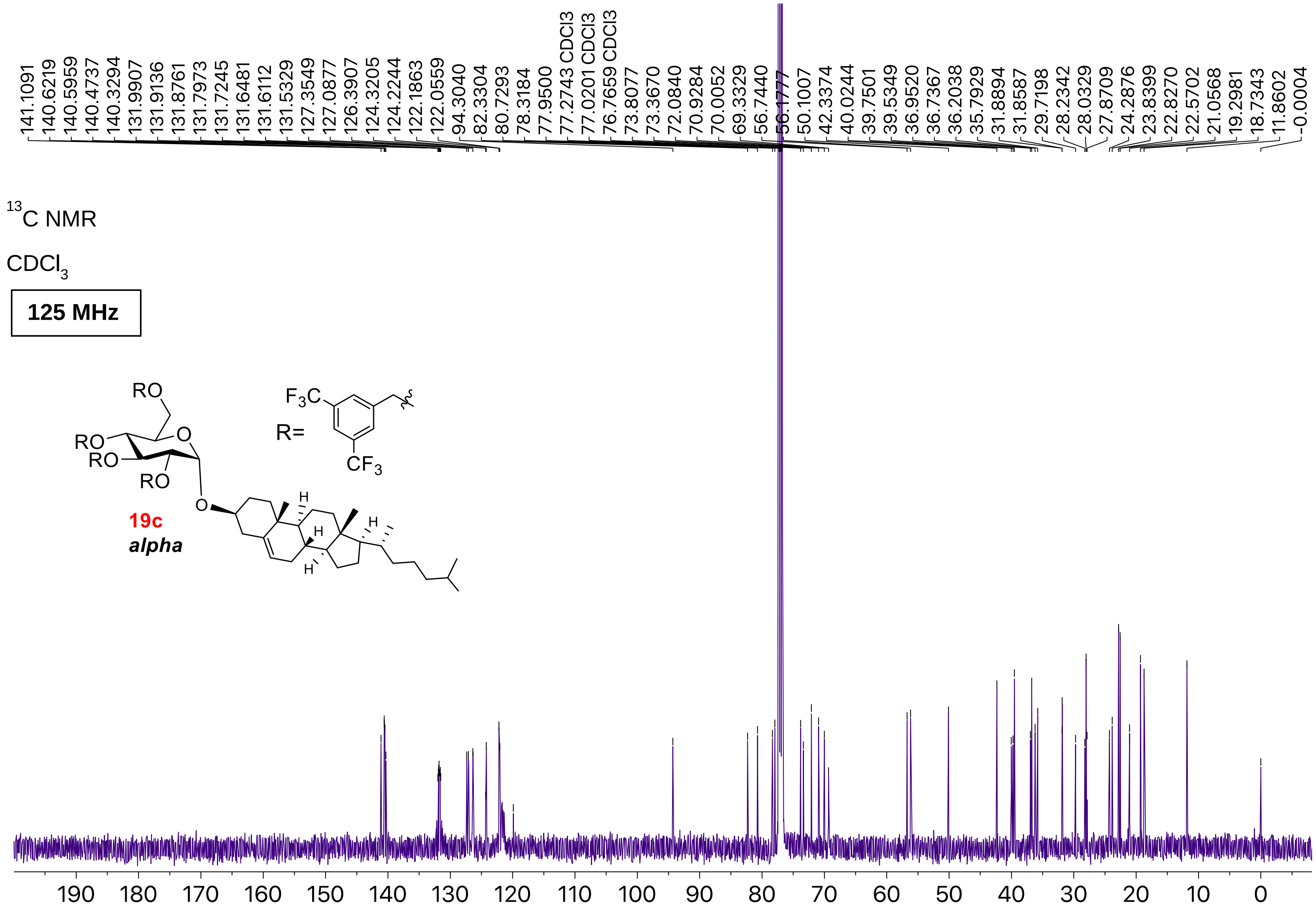
141.1091
140.6219
140.5959
140.4737
140.3294
131.9907
131.9136
131.8761
131.7973
131.7245
131.6481
131.6112
131.5329
127.3549
127.0877
126.3907
124.3205
124.2244
122.1863
122.0559
94.3040
82.3304
80.7293
78.3184
77.9500
77.2743 CDCl3
77.0201 CDCl3
76.7659 CDCl3
73.8077
73.3670
72.0840
70.9284
70.0052
69.3329
56.7440
56.1777
50.1007
42.3374
40.0244
39.7501
39.5349
36.9520
36.7367
36.2038
35.7929
31.8894
31.8587
29.7198
28.2342
28.0329
27.8709
24.2876
23.8399
22.8270
22.5702
21.0568
19.2981
18.7343
11.8602
-0.0004
13C NMR
CDCl3
125 MHz
RO
RO
RO
RO
O
O
H
H
H
H
R=
F3C
CF3
19c
alpha
190
180
170
160
150
140
130
120
110
100
90
80
70
60
50
40
30
20
10
0

^{19}F NMR

$CDCl_3$

471 MHz

19c
alpha

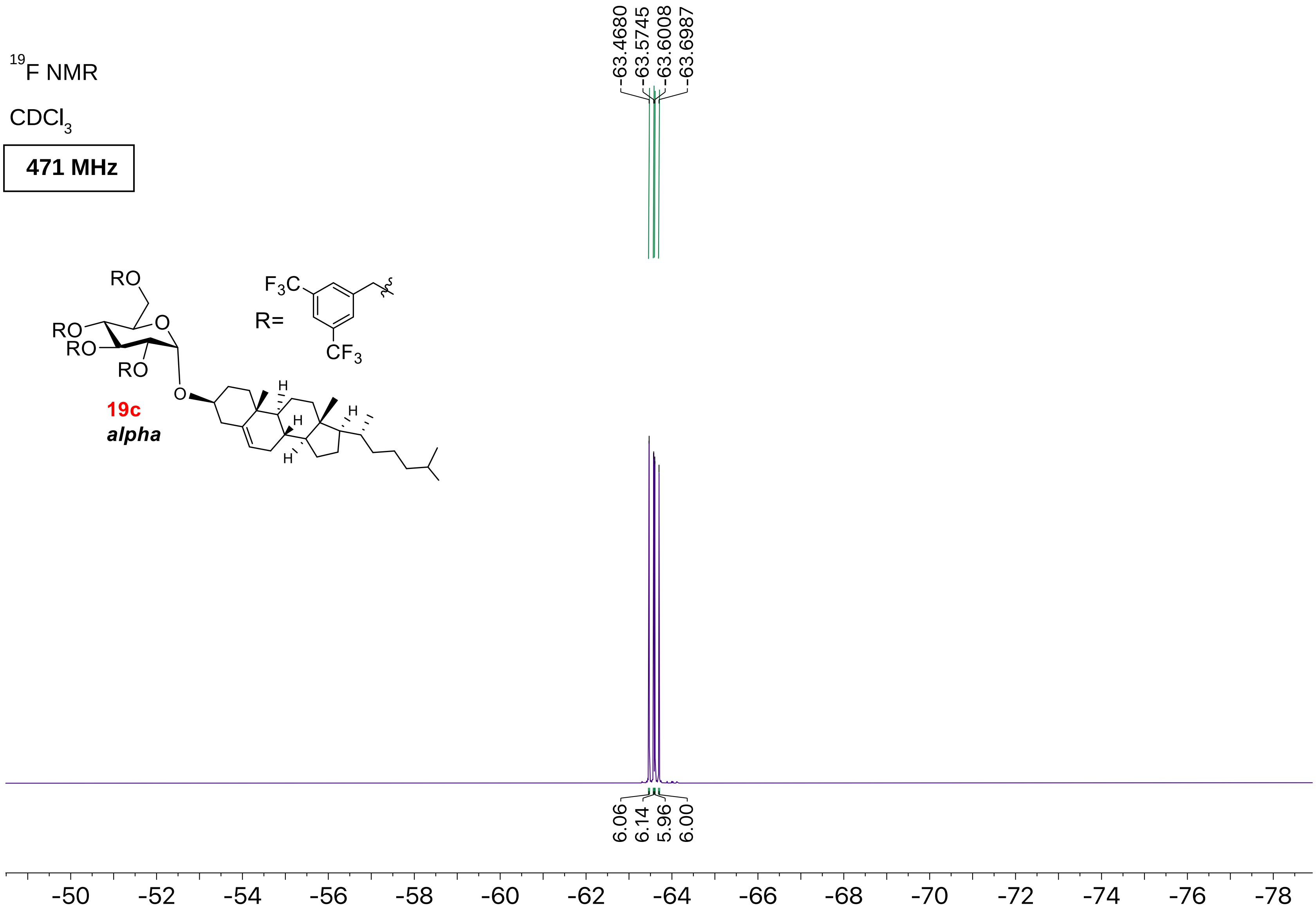

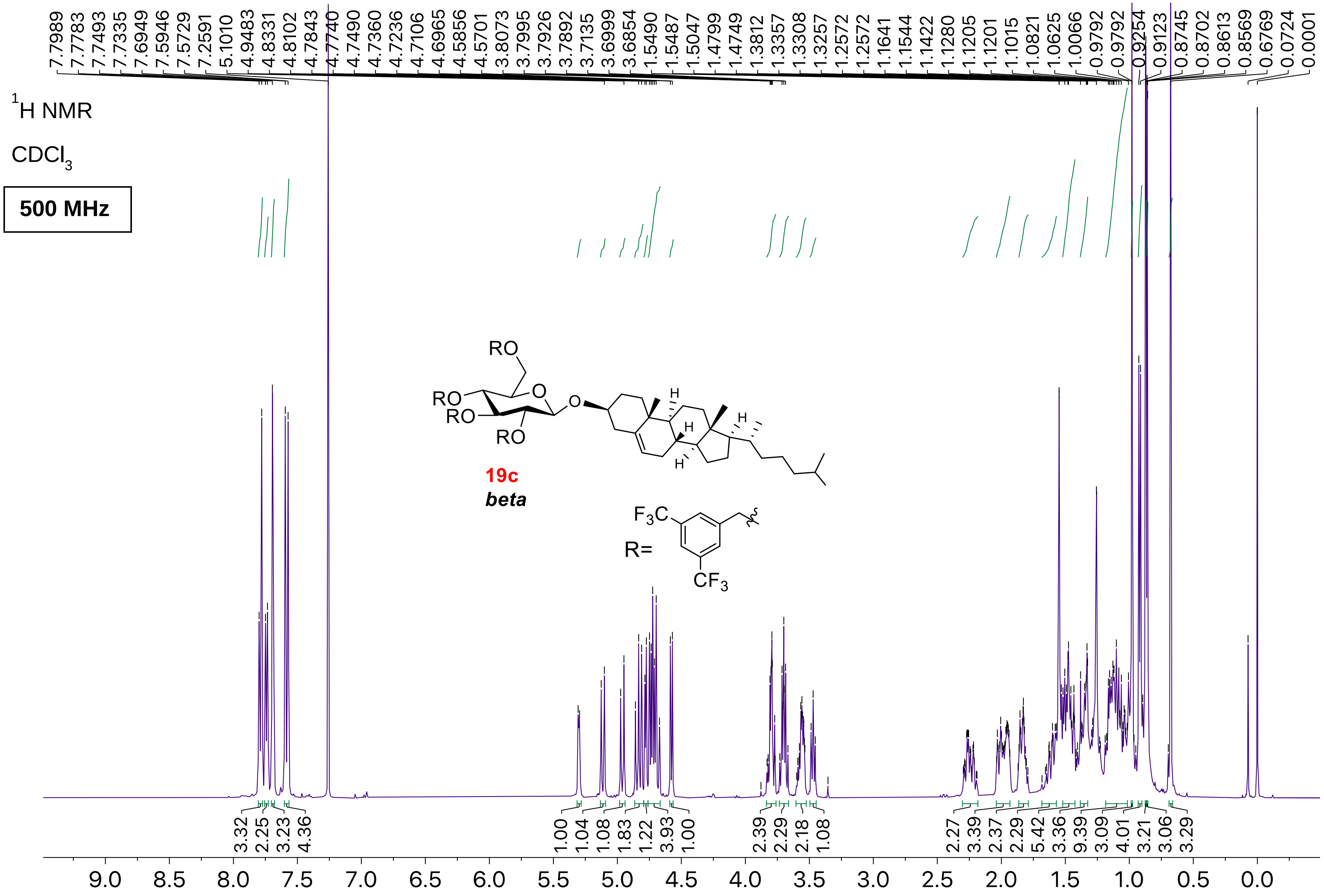

7.7989
7.7783
7.7493
7.7335
7.6949
7.5946
7.5729
7.2591
5.1010
4.9483
4.8331
4.8102
4.7843
4.7740
4.7490
4.7360
4.7236
4.7106
4.6965
4.5856
4.5701
3.8073
3.7995
3.7926
3.7892
3.7135
3.6999
3.6854
1.5490
1.5487
1.5047
1.4799
1.4749
1.3812
1.3357
1.3308
1.3257
1.2572
1.2572
1.1641
1.1544
1.1422
1.1280
1.1205
1.1201
1.1015
1.0821
1.0625
1.0066
0.9792
0.9792
0.9254
0.9123
0.8745
0.8702
0.8613
0.8569
0.6769
0.0724
0.0001
1H NMR
CDCl3
500 MHz
RO
RO
RO
O
O
RO
H
H
H
H
19c
beta
F3C
R=
CF3
3.32
2.25
3.23
4.36
1.00
1.04
1.08
1.83
1.22
3.93
1.00
2.39
2.29
2.18
1.08
2.27
3.39
2.37
2.29
5.42
3.36
9.39
3.09
4.01
3.21
3.06
3.29
9.0
8.5
8.0
7.5
7.0
6.5
6.0
5.5
5.0
4.5
4.0
3.5
3.0
2.5
2.0
1.5
1.0
0.5
0.0

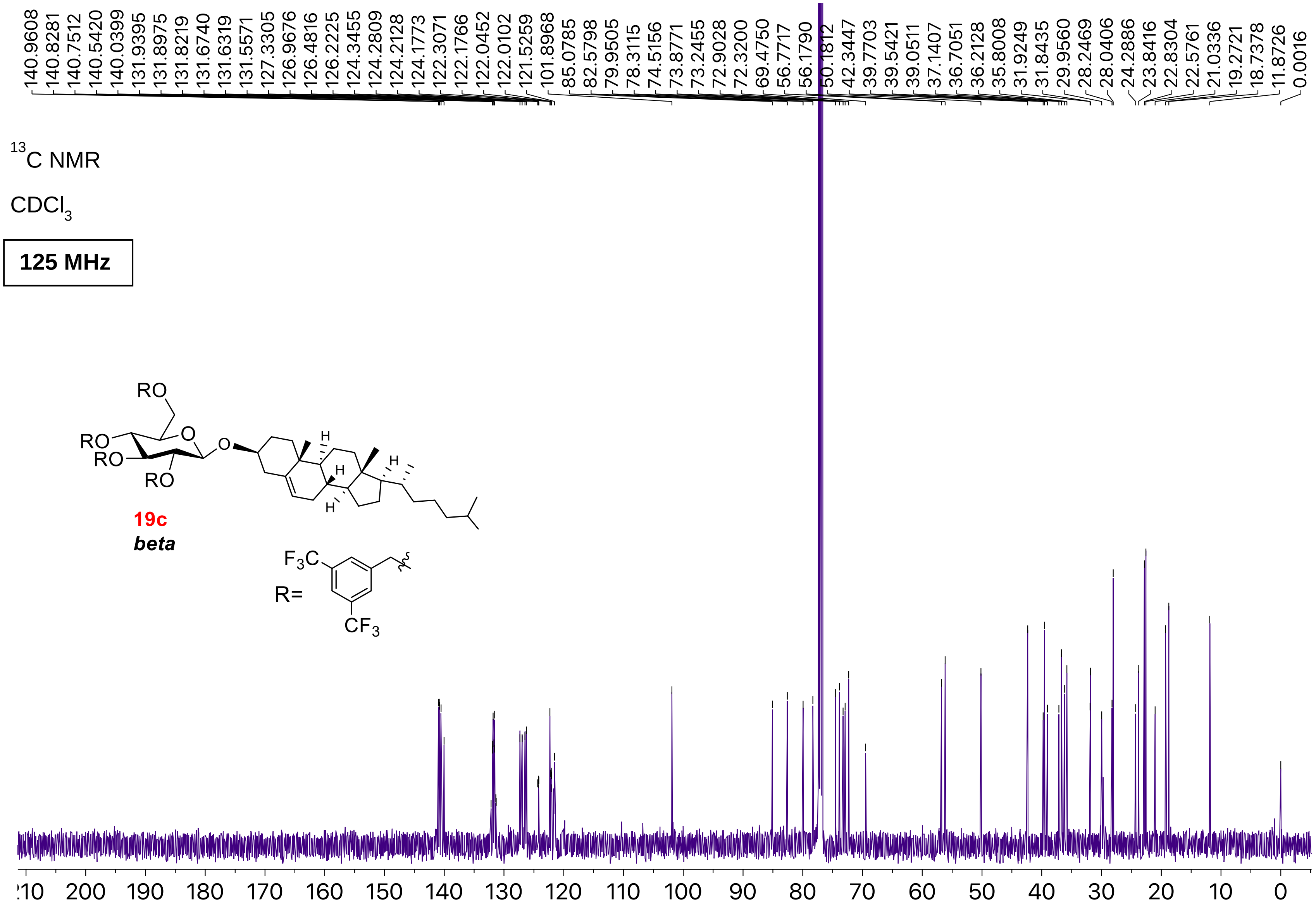

13C NMR
CDCl3
125 MHz
19c
beta
R=

^{19}F NMR

$CDCl_3$

471 MHz

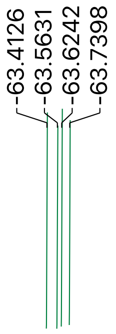

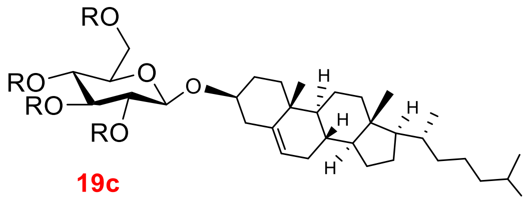

19c
beta

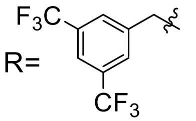

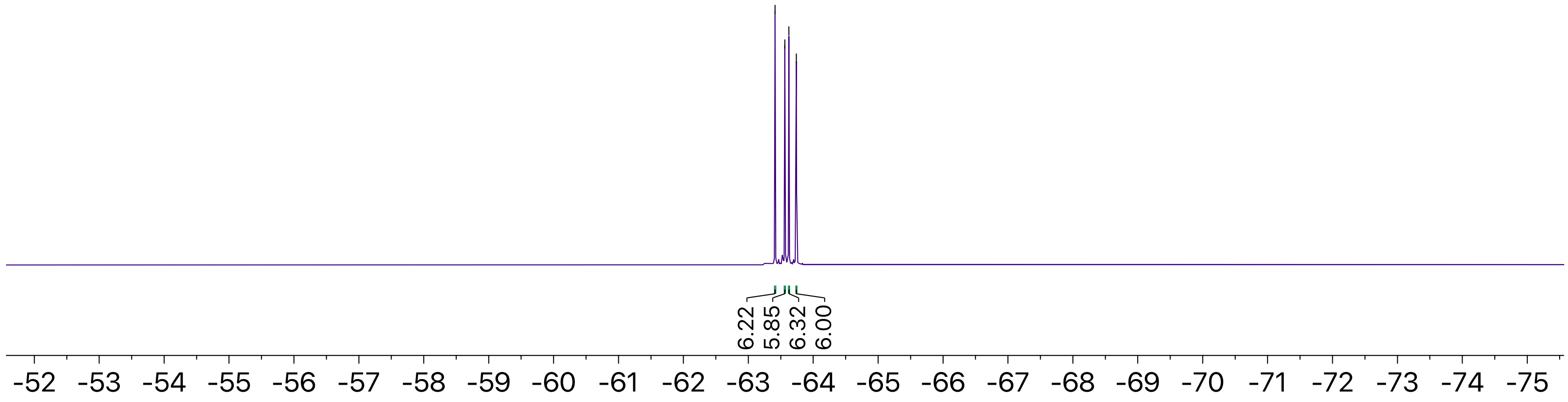

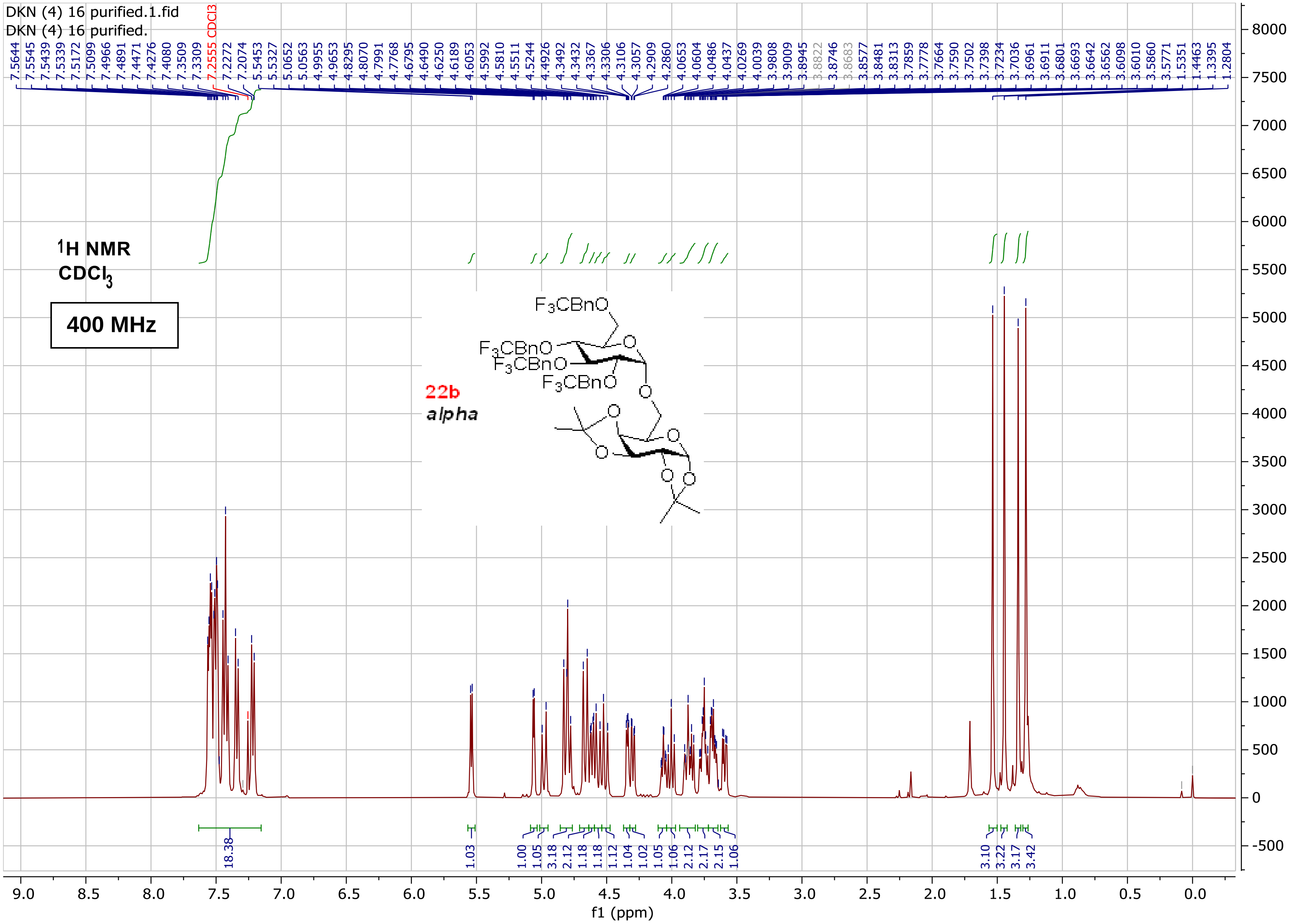
DKN (4) 16 purified.1.fid
DKN (4) 16 purified.
1H NMR
CDCl3
400 MHz
22b
alpha
F3CBnO
F3CBnO
F3CBnO
F3CBnO
f1 (ppm)

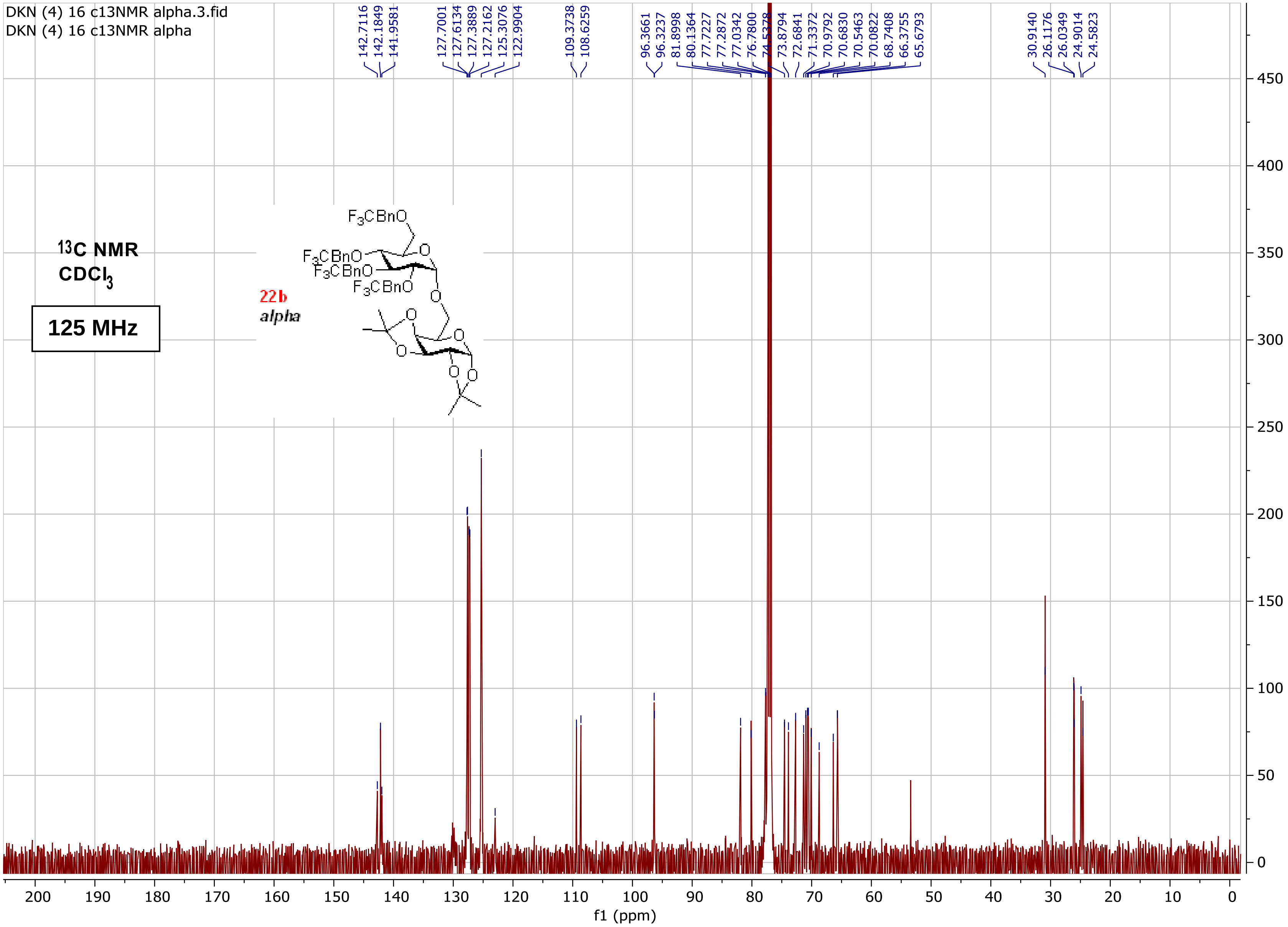

DKN (4) 16 c13NMR alpha.3.fid
DKN (4) 16 c13NMR alpha
142.7116
142.1849
141.9581
127.7001
127.6134
127.3889
127.2162
125.3076
122.9904
109.3738
108.6259
96.3661
96.3237
81.8998
80.1364
77.7227
77.2872
77.0342
76.7800
74.5378
73.8794
72.6841
71.3372
70.9792
70.6830
70.5463
70.0822
68.7408
66.3755
65.6793
30.9140
26.1176
26.0349
24.9014
24.5823
13C NMR
CDCl3
125 MHz
22b
alpha
F3CBnO
F3CBnO
F3CBnO
F3CBnO
O
O
O
O
O
O
O
f1 (ppm)
200
190
180
170
160
150
140
130
120
110
100
90
80
70
60
50
40
30
20
10
0
450
400
350
300
250
200
150
100
50
0

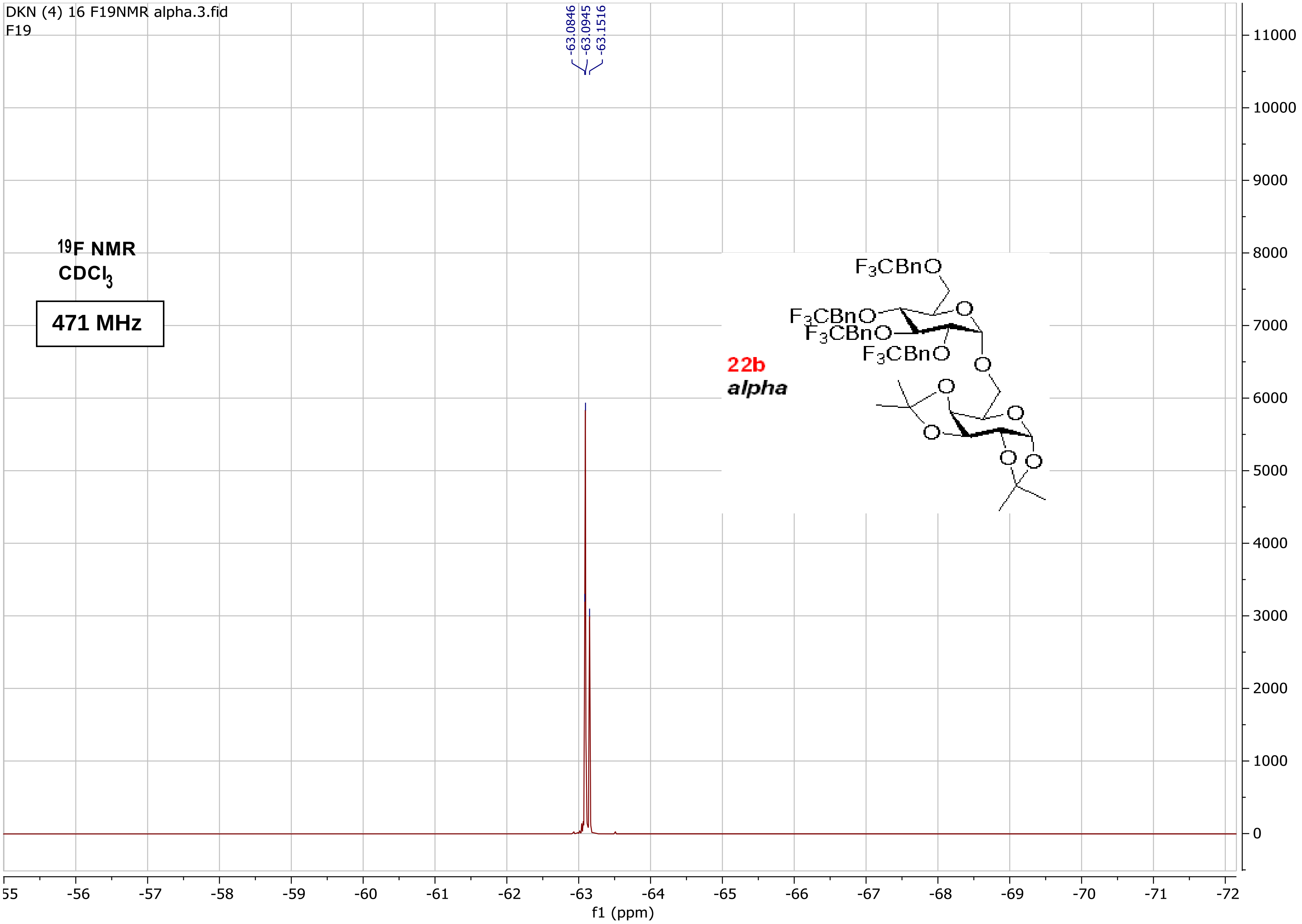

DKN (4) 16 F19NMR alpha.3.fid
F19
-63.0846
-63.0945
-63.1516
19F NMR
CDCl3
471 MHz
F3CBnO
F3CBnO
F3CBnO
F3CBnO
22b
alpha
f1 (ppm)

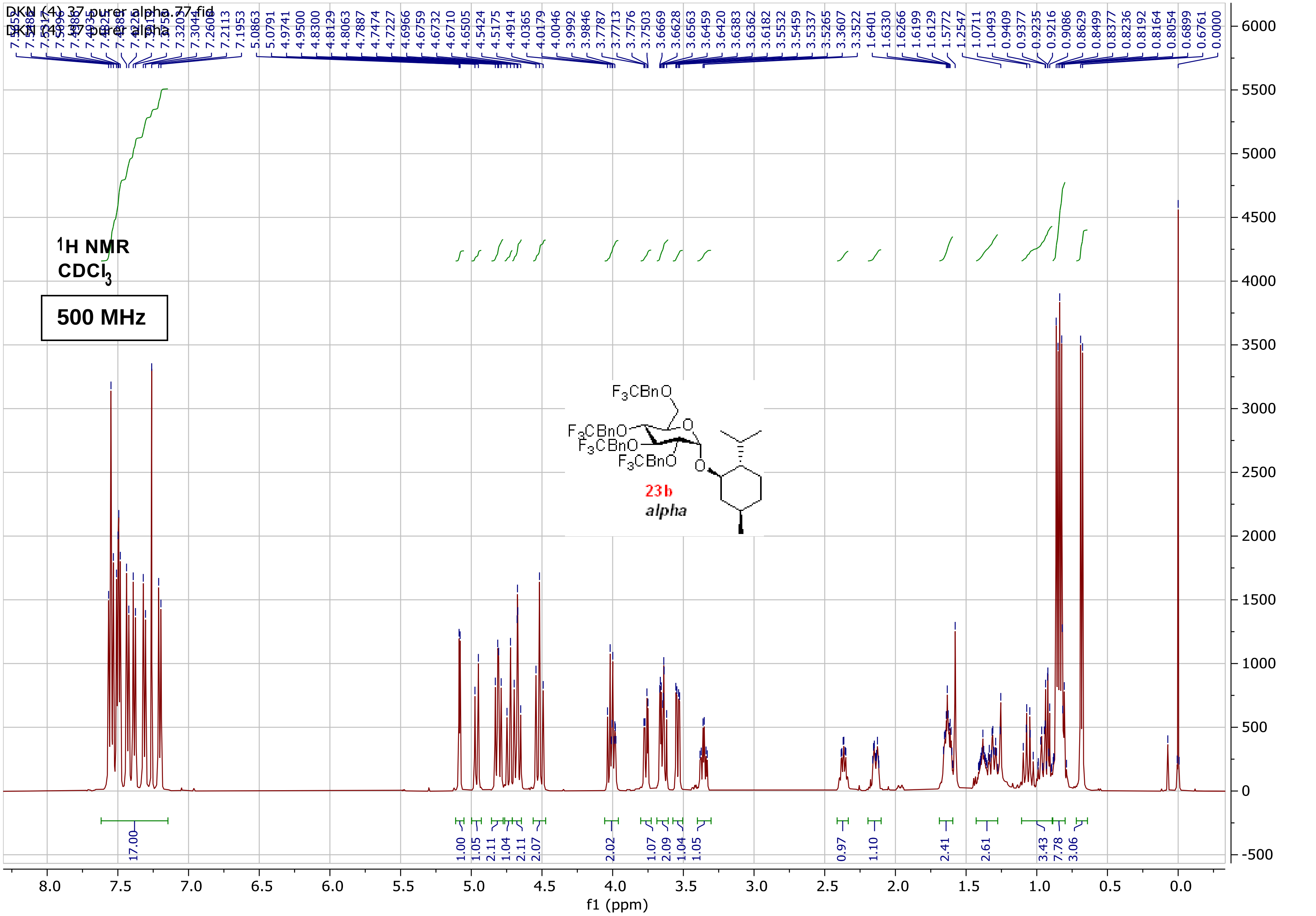

DKN (4) 37 purer alpha.77.fid
1H NMR
CDCl3
500 MHz
F3CBnO
F3CBnO
F3CBnO
F3CBnO
O
O
23b
alpha
f1 (ppm)

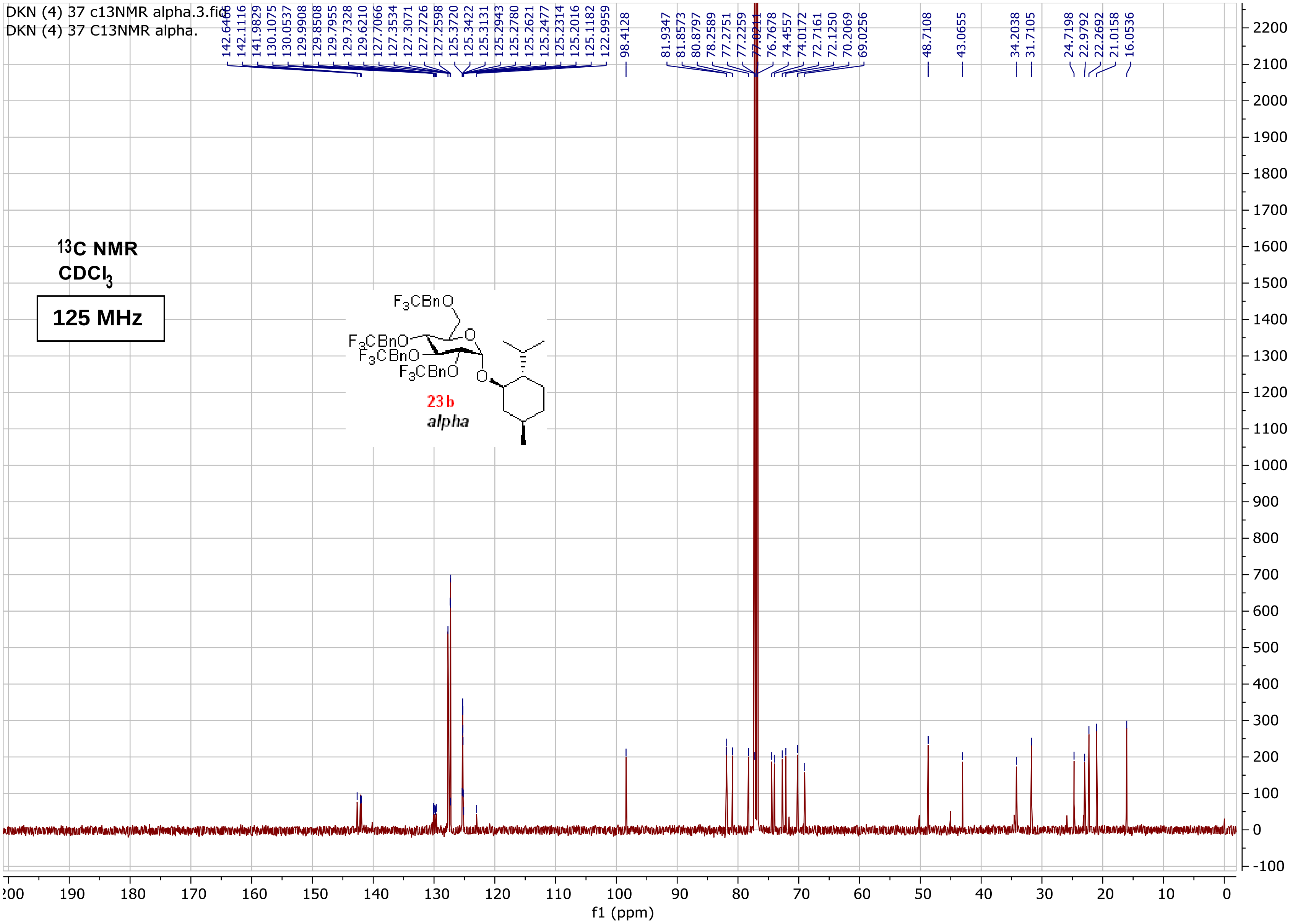

DKN (4) 37 c13NMR alpha.3.fid
DKN (4) 37 C13NMR alpha.
13C NMR
CDCl3
125 MHz
F3CBnO
F3CBnO
F3CBnO
F3CBnO
O
O
23b
alpha
f1 (ppm)

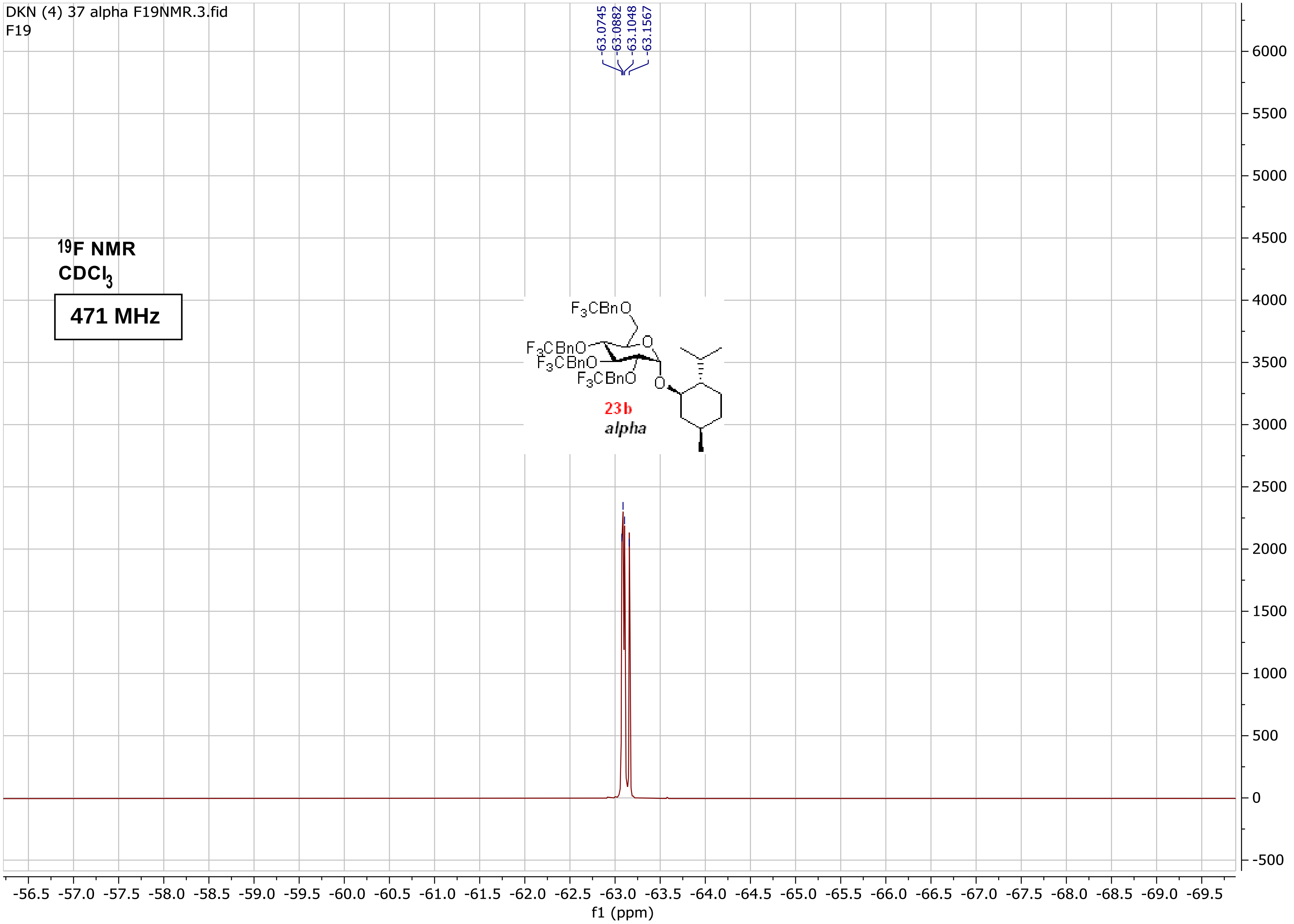

DKN (4) 37 alpha F19NMR.3.fid
F19
-63.0745
-63.0882
-63.1048
-63.1567
19F NMR
CDCl3
471 MHz
F3CBnO
F3CBnO
F3CBnO
F3CBnO
O
O
23b
alpha
f1 (ppm)

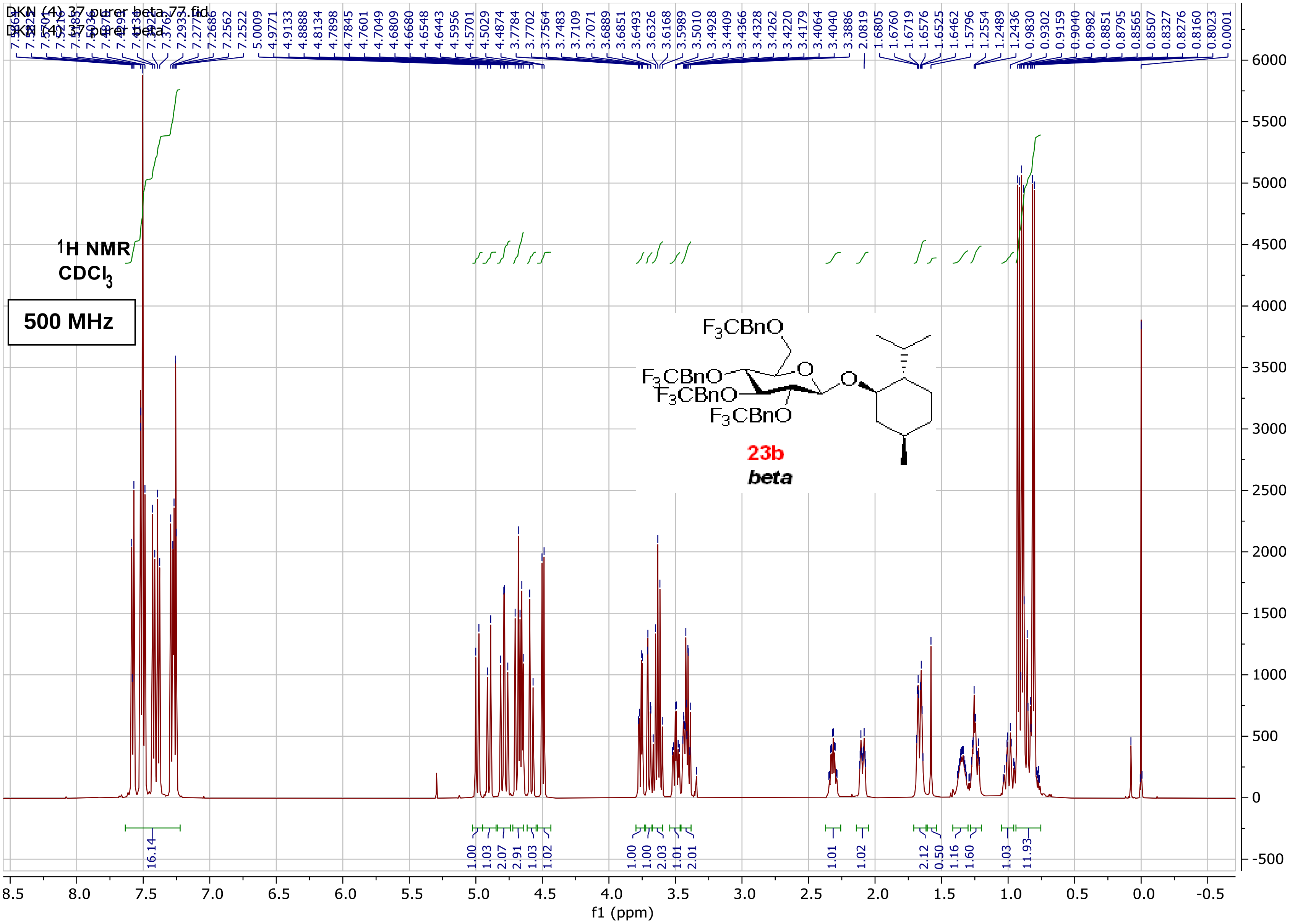

DKN (4) 37 purer beta 77.fid
1H NMR
CDCl3
500 MHz
F3CBnO
F3CBnO
F3CBnO
F3CBnO
23b
beta
f1 (ppm)

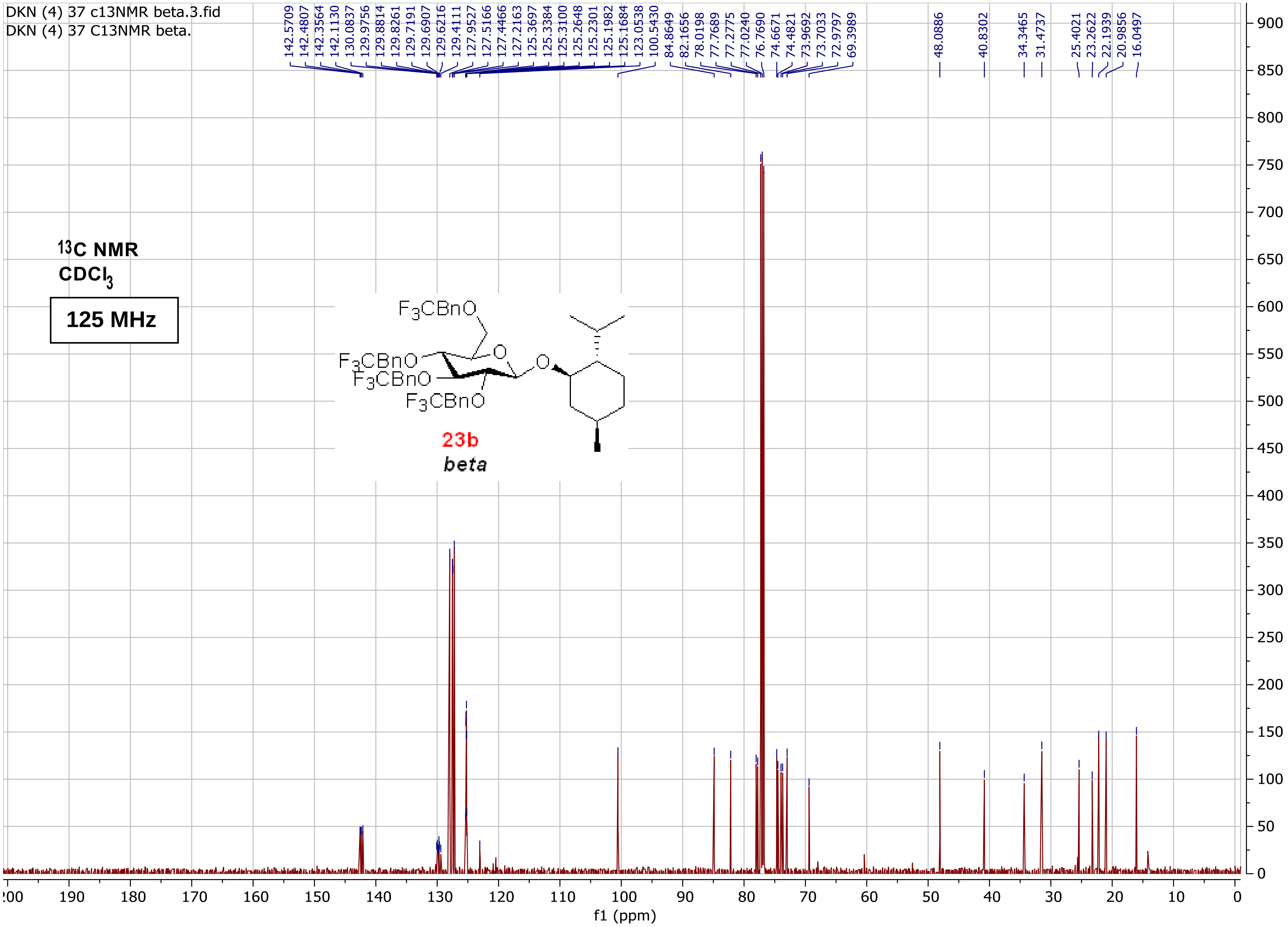
DKN (4) 37 c13NMR beta.3.fid
DKN (4) 37 C13NMR beta.
142.5709
142.4807
142.3564
142.1130
130.0837
129.9756
129.8814
129.8261
129.7191
129.6907
129.6216
129.4111
127.9527
127.5166
127.4466
127.2163
125.3697
125.3384
125.3100
125.2648
125.2301
125.1982
125.1684
123.0538
100.5430
84.8649
82.1656
78.0198
77.7689
77.2775
77.0240
76.7690
74.6671
74.4821
73.9692
73.7033
72.9797
69.3989
48.0886
40.8302
34.3465
31.4737
25.4021
23.2622
22.1939
20.9856
16.0497
13C NMR
CDCl3
125 MHz
F3CBnO
F3CBnO
F3CBnO
F3CBnO
O
O
23b
beta
f1 (ppm)

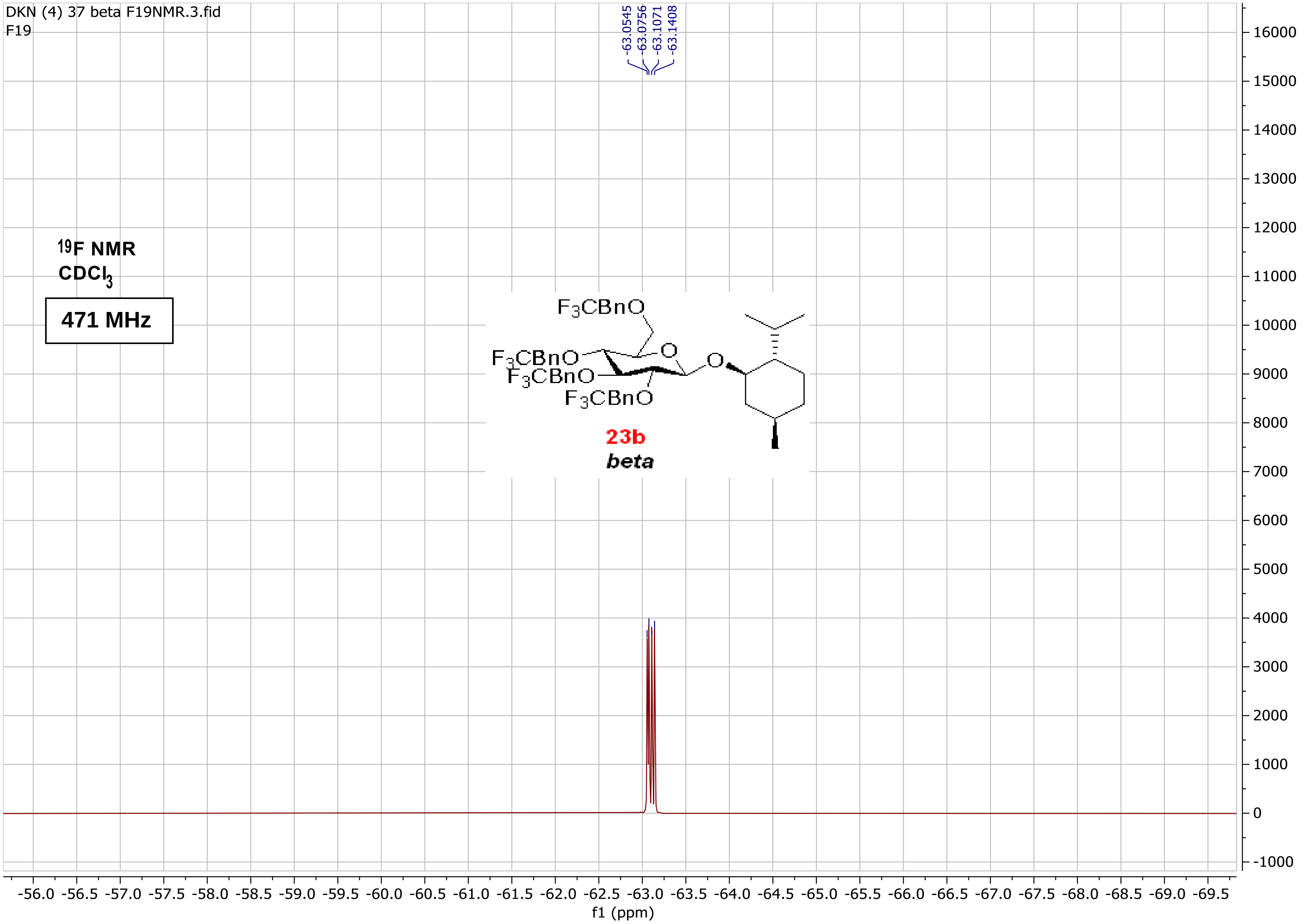
DKN (4) 37 beta F19NMR.3.fid
F19
-63.0545
-63.0756
-63.1071
-63.1408
19F NMR
CDCl3
471 MHz
F3CBnO
F3CBnO
F3CBnO
F3CBnO
O
O
23b
beta
16000
15000
14000
13000
12000
11000
10000
9000
8000
7000
6000
5000
4000
3000
2000
1000
0
-1000
-56.0 -56.5 -57.0 -57.5 -58.0 -58.5 -59.0 -59.5 -60.0 -60.5 -61.0 -61.5 -62.0 -62.5 -63.0 -63.5 -64.0 -64.5 -65.0 -65.5 -66.0 -66.5 -67.0 -67.5 -68.0 -68.5 -69.0 -69.5
f1 (ppm)

^{1}H NMR (400 MHz, D_2O)

^{1}H NMR
D_2O

400MHz

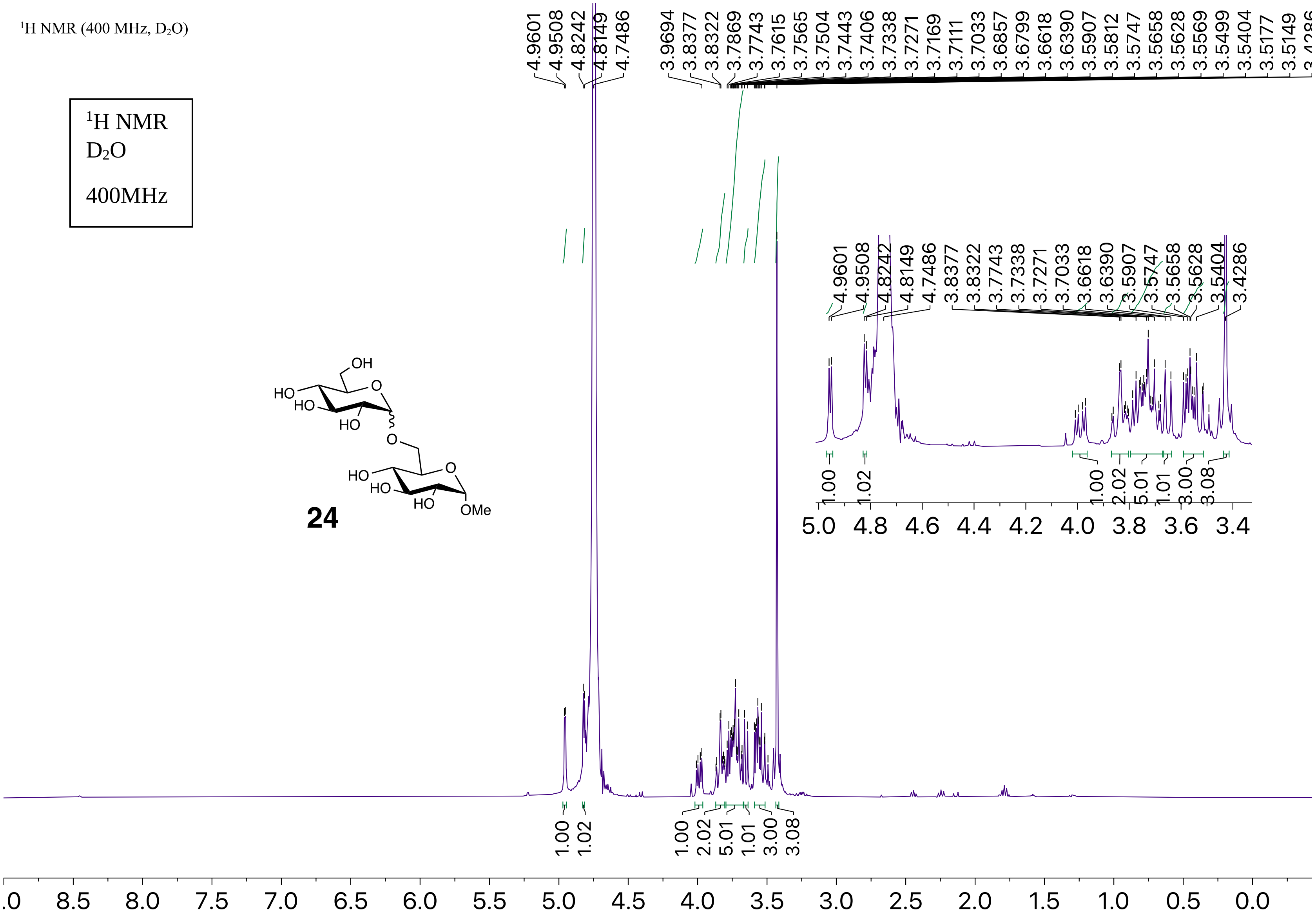

Anomeric Ratios Determination

Note: Entries refer to Table 1 of the SI document, not Table 1 of the manuscript.

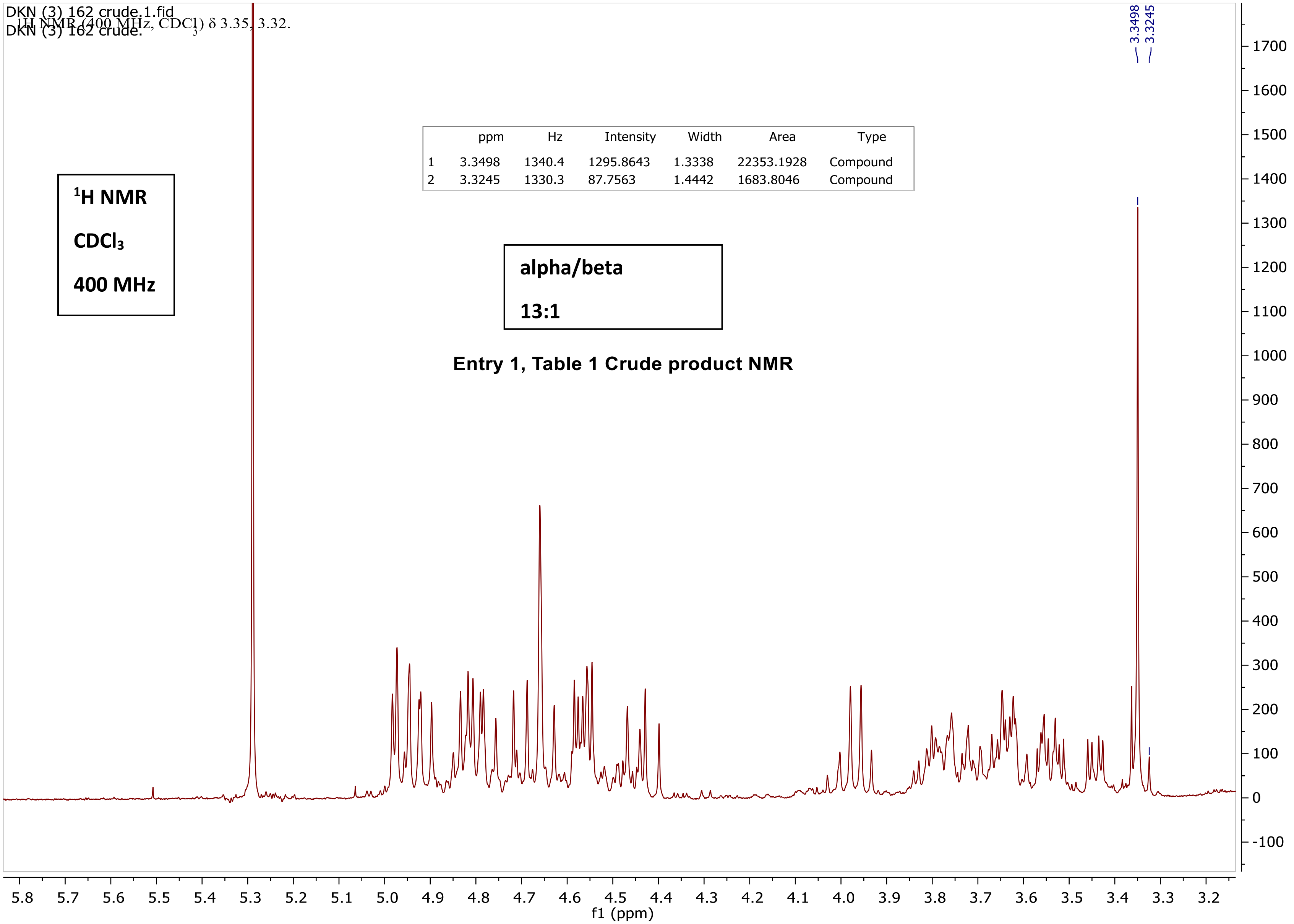

	ppm	Hz	Intensity	Width	Area	Type
1	3.3498	1340.4	1295.8643	1.3338	22353.1928	Compound
2	3.3245	1330.3	87.7563	1.4442	1683.8046	Compound

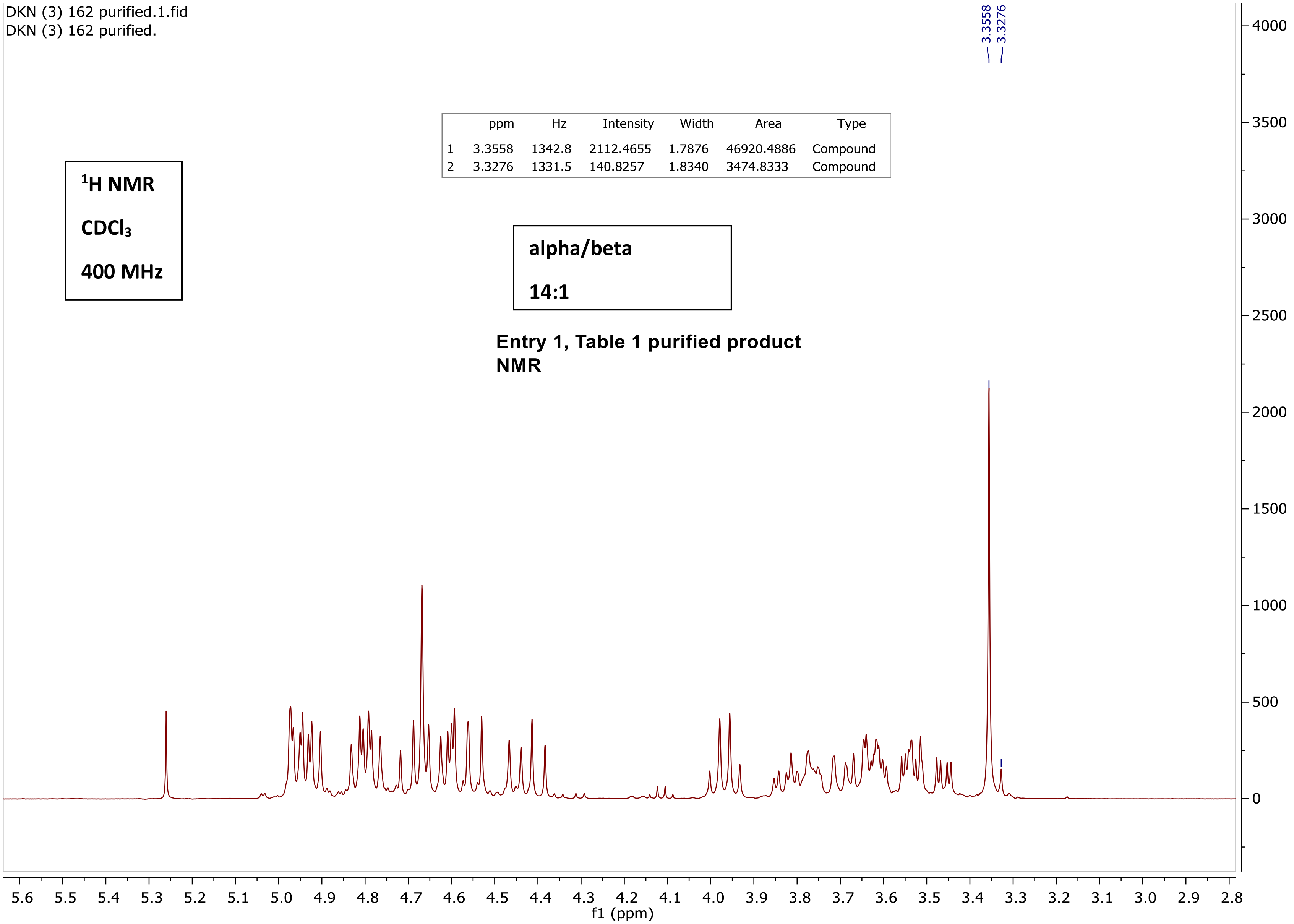
DKN (3) 162 purified.1.fid
DKN (3) 162 purified.
3.3558
3.3276
ppm Hz Intensity Width Area Type
1 3.3558 1342.8 2112.4655 1.7876 46920.4886 Compound
2 3.3276 1331.5 140.8257 1.8340 3474.8333 Compound
¹H NMR
CDCl₃
400 MHz
alpha/beta
14:1
Entry 1, Table 1 purified product NMR
5.6 5.5 5.4 5.3 5.2 5.1 5.0 4.9 4.8 4.7 4.6 4.5 4.4 4.3 4.2 4.1 4.0 3.9 3.8 3.7 3.6 3.5 3.4 3.3 3.2 3.1 3.0 2.9 2.8
f1 (ppm)
4000
3500
3000
2500
2000
1500
1000
500
0

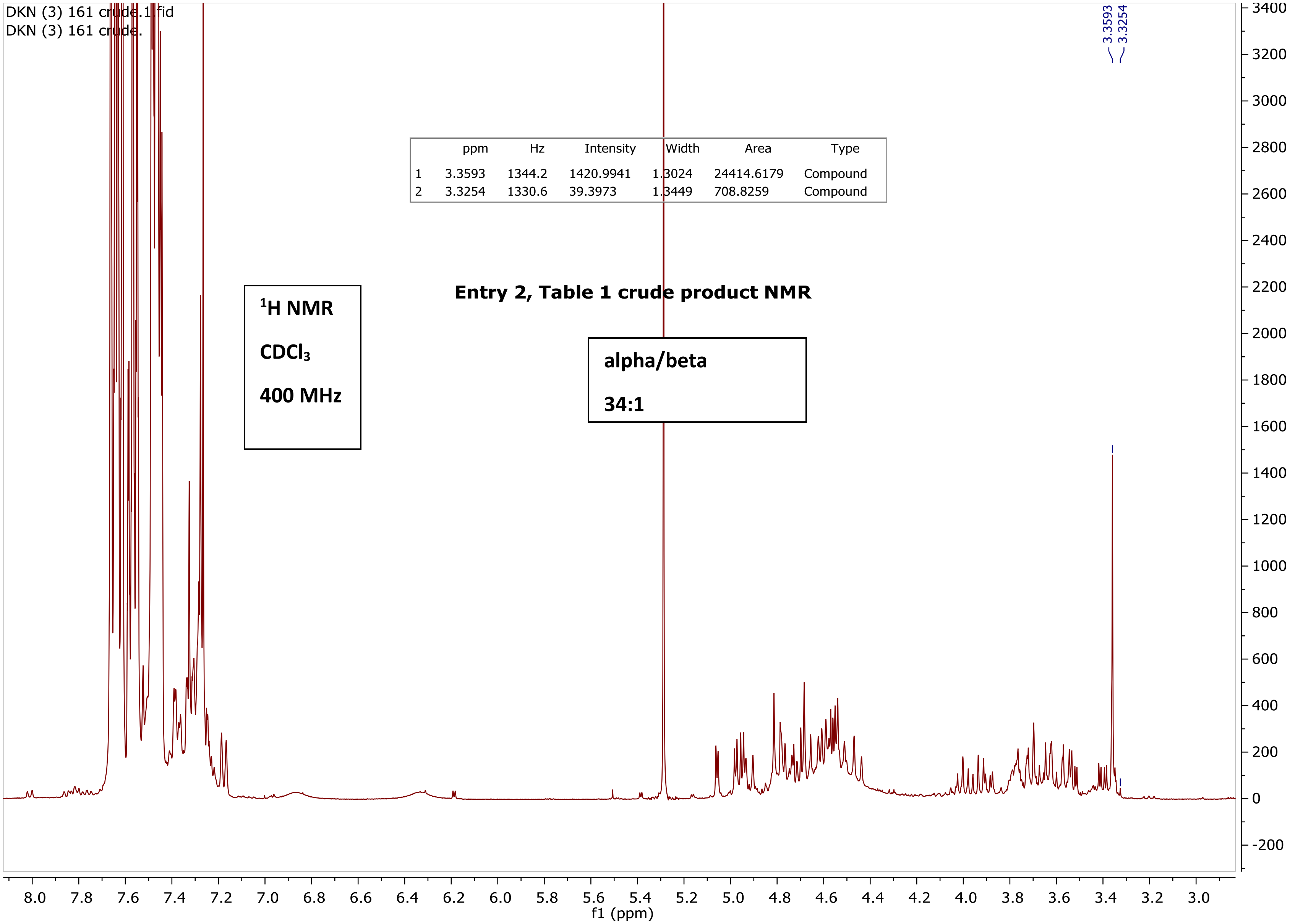

	ppm	Hz	Intensity	Width	Area	Type
1	3.3593	1344.2	1420.9941	1.3024	24414.6179	Compound
2	3.3254	1330.6	39.3973	1.3449	708.8259	Compound

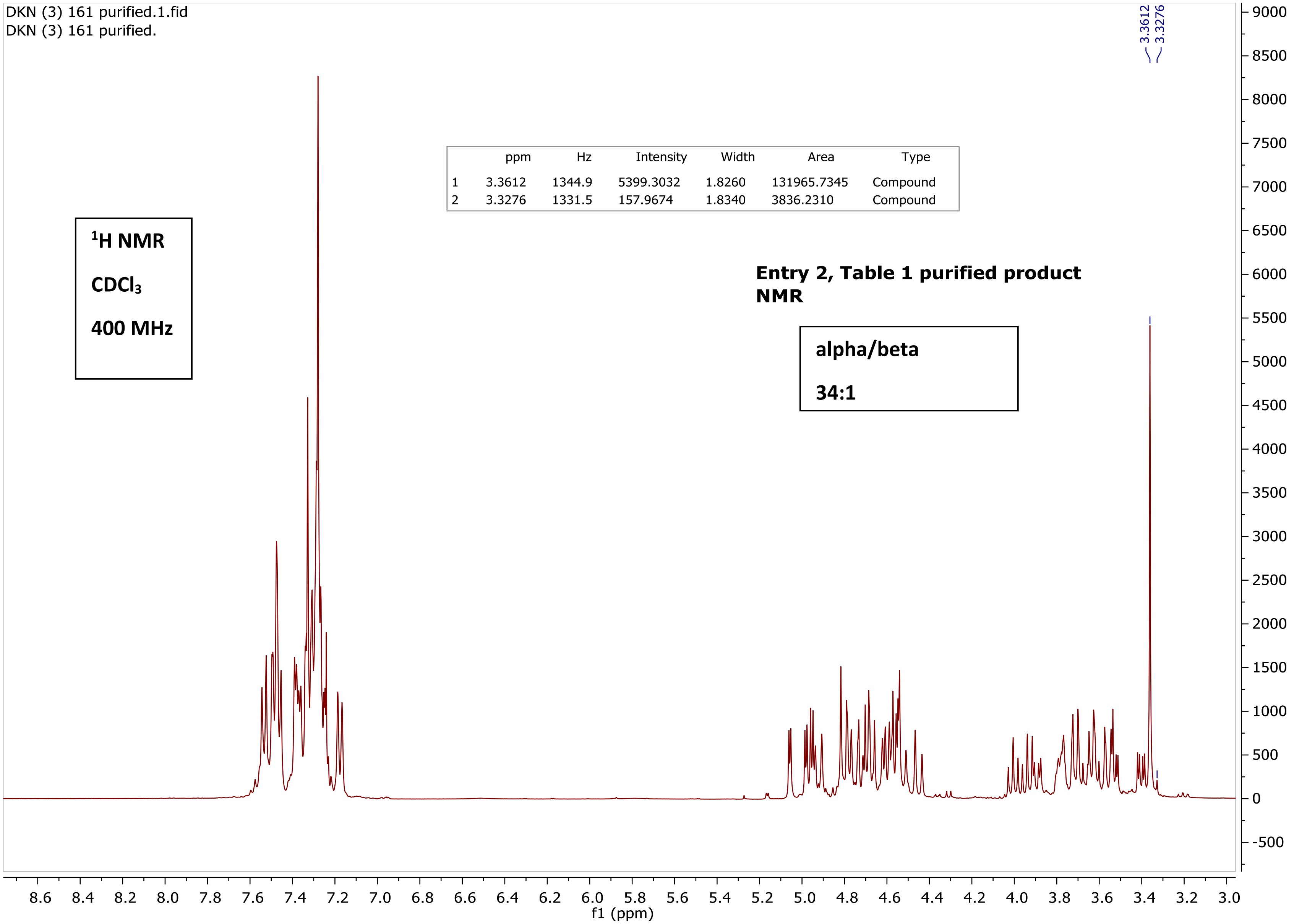

	ppm	Hz	Intensity	Width	Area	Type
1	3.3612	1344.9	5399.3032	1.8260	131965.7345	Compound
2	3.3276	1331.5	157.9674	1.8340	3836.2310	Compound

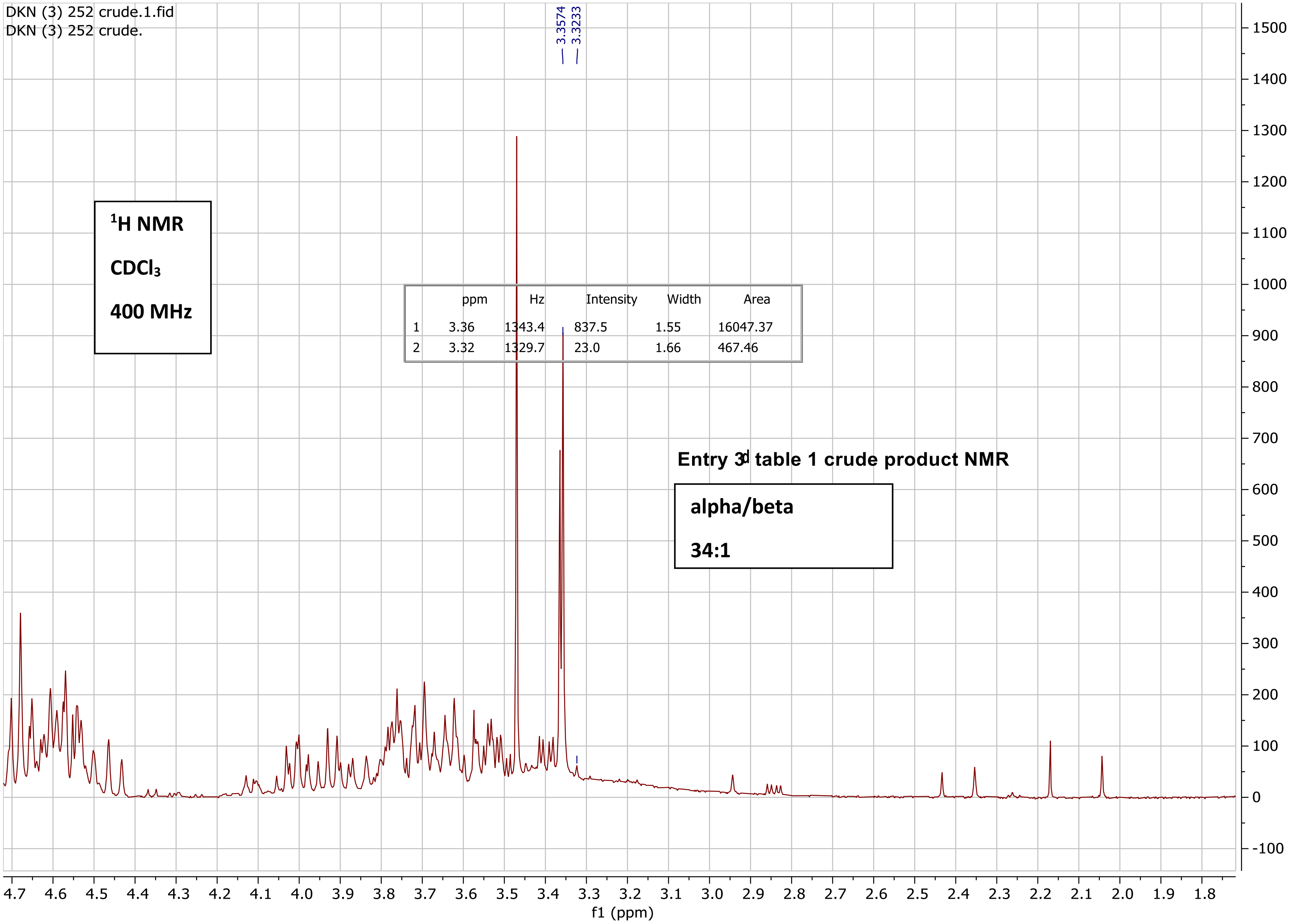
DKN (3) 252 crude.1.fid
DKN (3) 252 crude.
3.3574
3.3233
1H NMR
CDCl3
400 MHz
ppm Hz Intensity Width Area
1 3.36 1343.4 837.5 1.55 16047.37
2 3.32 1329.7 23.0 1.66 467.46
Entry 3d table 1 crude product NMR
alpha/beta
34:1
f1 (ppm)

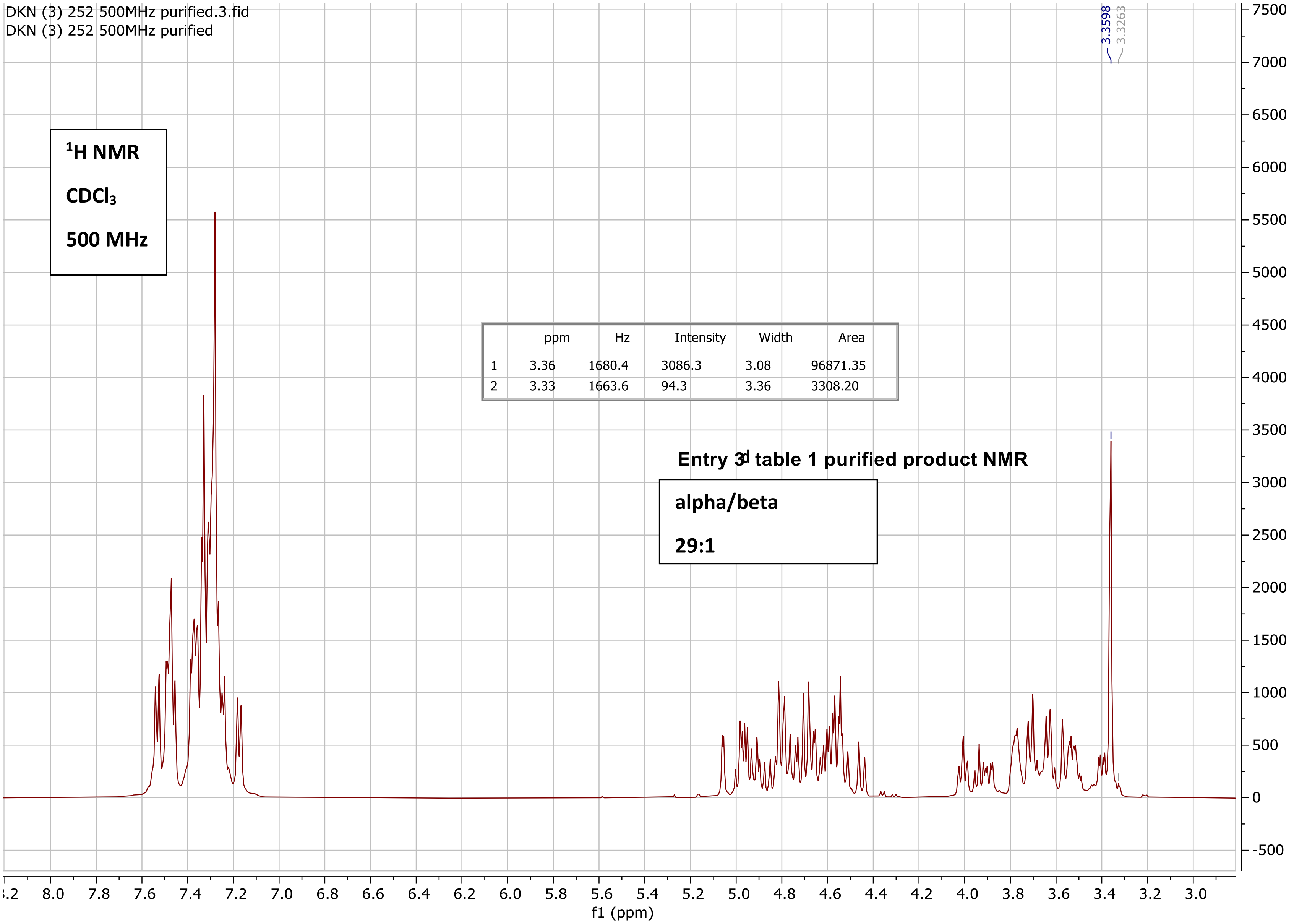
DKN (3) 252 500MHz purified.3.fid
DKN (3) 252 500MHz purified
1H NMR
CDCl3
500 MHz
ppm Hz Intensity Width Area
1 3.36 1680.4 3086.3 3.08 96871.35
2 3.33 1663.6 94.3 3.36 3308.20
Entry 3d table 1 purified product NMR
alpha/beta
29:1
3.3598
3.3263
f1 (ppm)

DKN (3) 272 crude.1.fid
DKN (3) 272 crude.

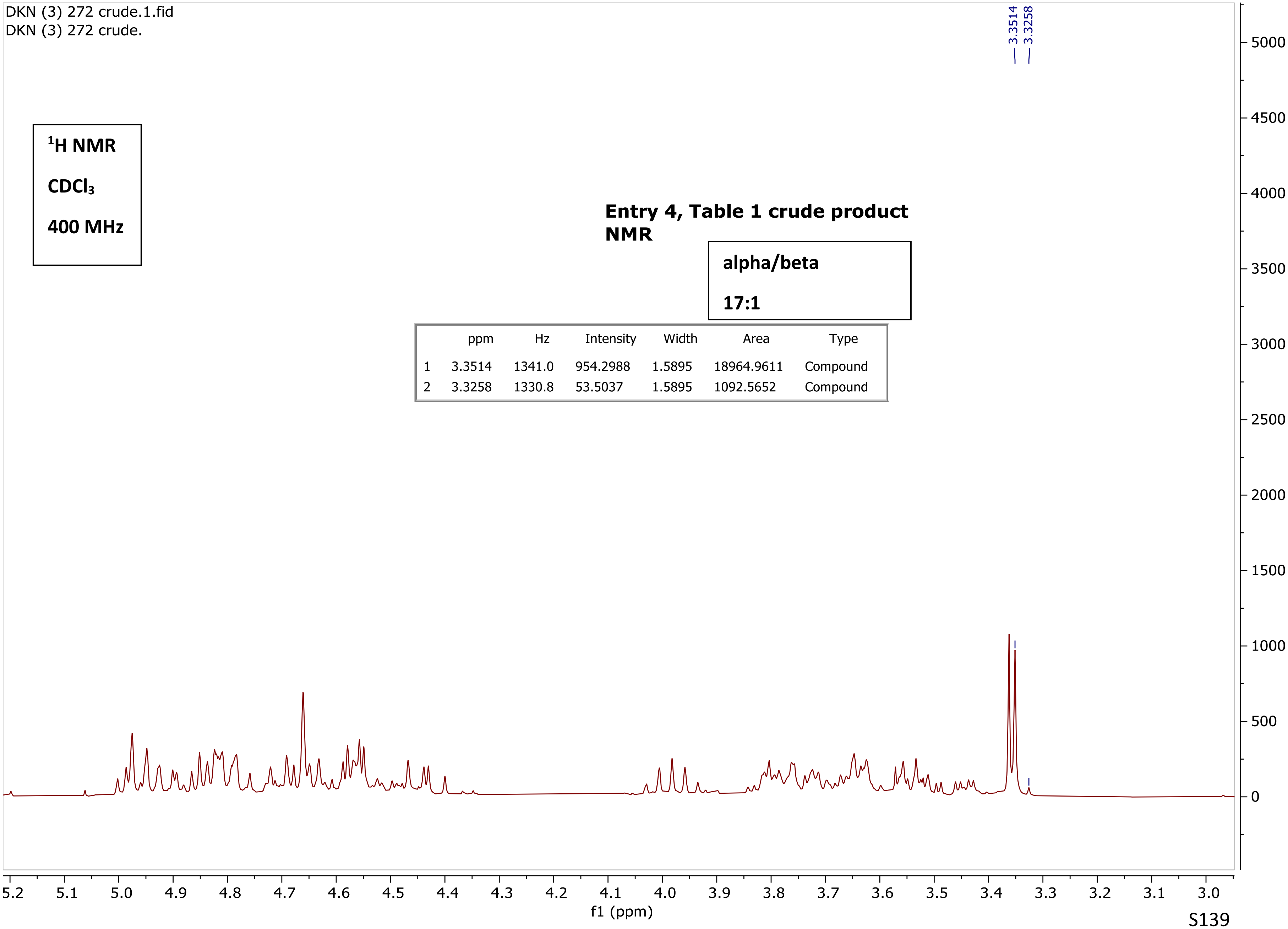

^{1}H NMR

$CDCl_3$

400 MHz

Entry 4, Table 1 crude product NMR

alpha/beta

17:1

	ppm	Hz	Intensity	Width	Area	Type
1	3.3514	1341.0	954.2988	1.5895	18964.9611	Compound
2	3.3258	1330.8	53.5037	1.5895	1092.5652	Compound

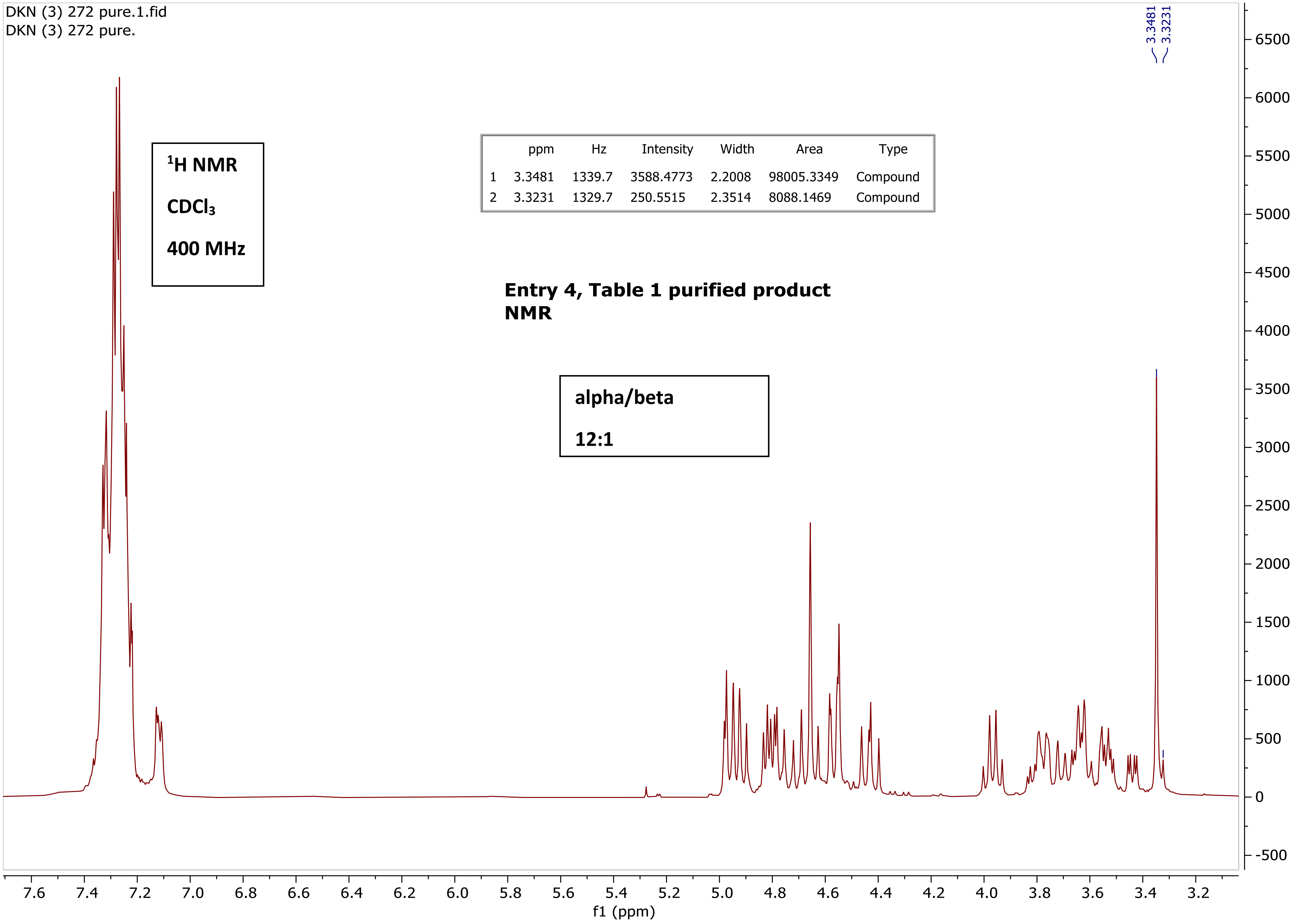

	ppm	Hz	Intensity	Width	Area	Type
1	3.3481	1339.7	3588.4773	2.2008	98005.3349	Compound
2	3.3231	1329.7	250.5515	2.3514	8088.1469	Compound

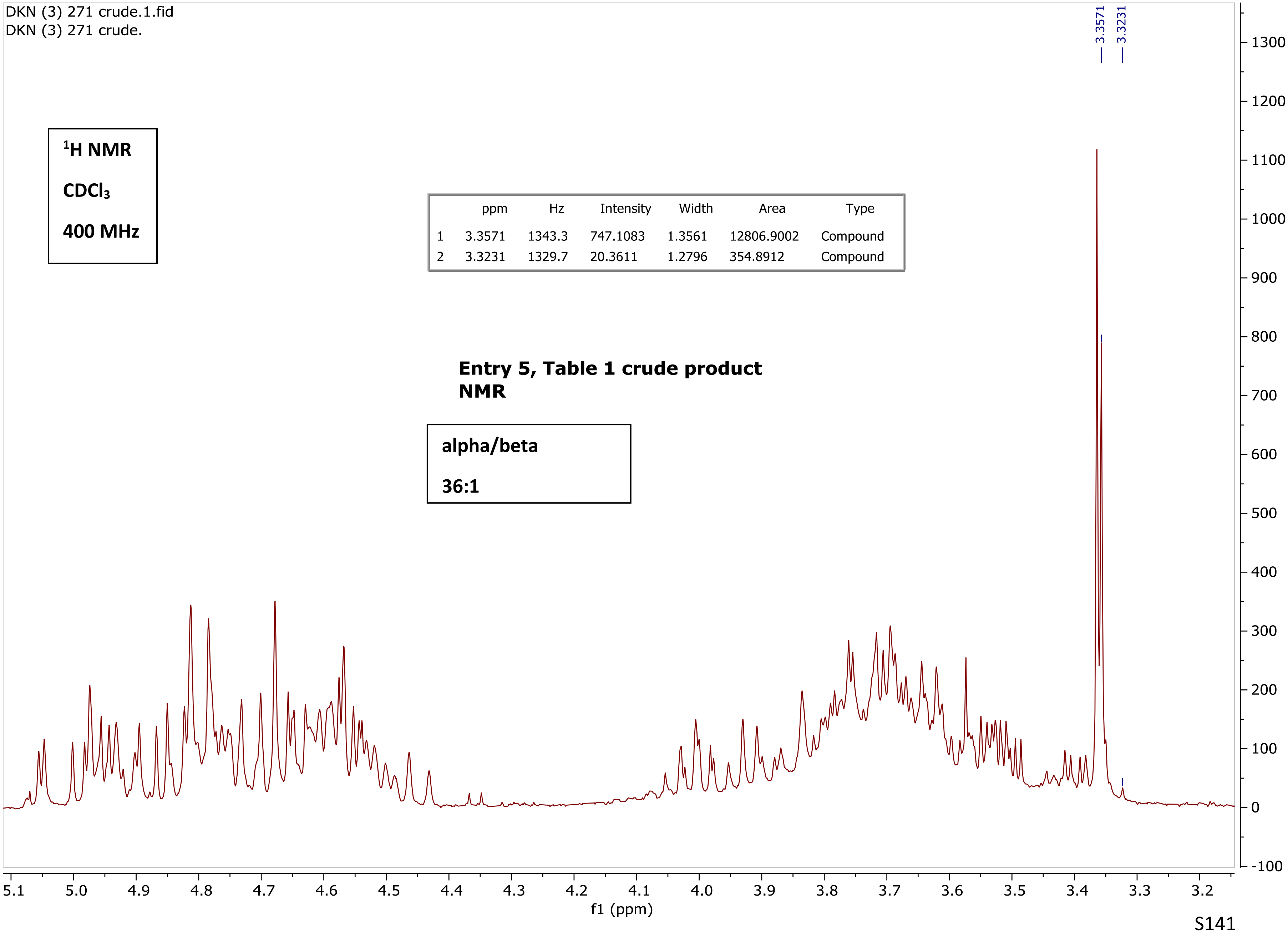

^1H NMR

$CDCl_3$

400 MHz

	ppm	Hz	Intensity	Width	Area	Type
1	3.3571	1343.3	747.1083	1.3561	12806.9002	Compound
2	3.3231	1329.7	20.3611	1.2796	354.8912	Compound

Entry 5, Table 1 crude product NMR

alpha/beta

36:1

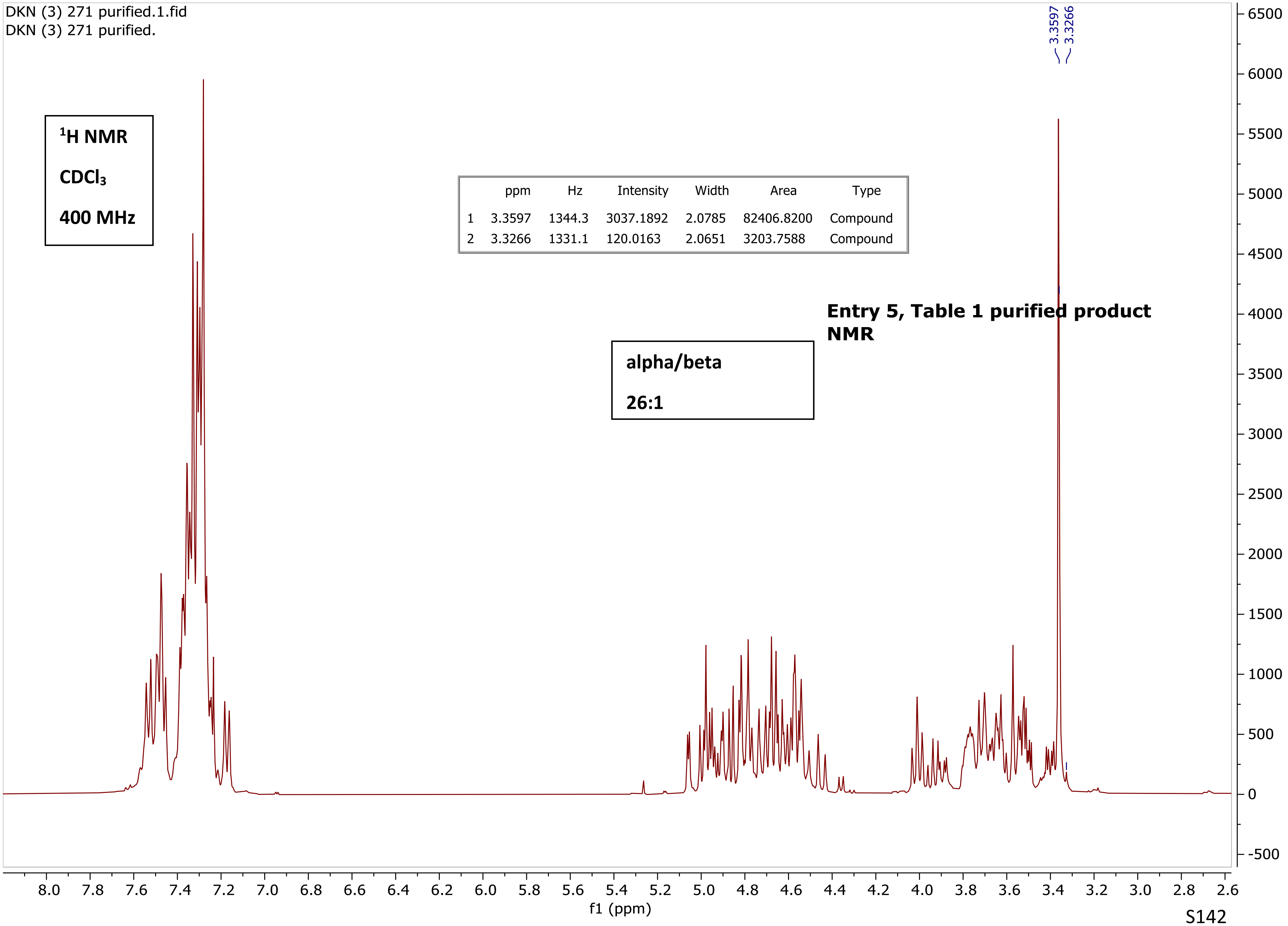

DKN (3) 271 purified.1.fid
DKN (3) 271 purified.
1H NMR
CDCl3
400 MHz
ppm Hz Intensity Width Area Type
1 3.3597 1344.3 3037.1892 2.0785 82406.8200 Compound
2 3.3266 1331.1 120.0163 2.0651 3203.7588 Compound
Entry 5, Table 1 purified product NMR
alpha/beta
26:1
3.3597
3.3266
f1 (ppm)

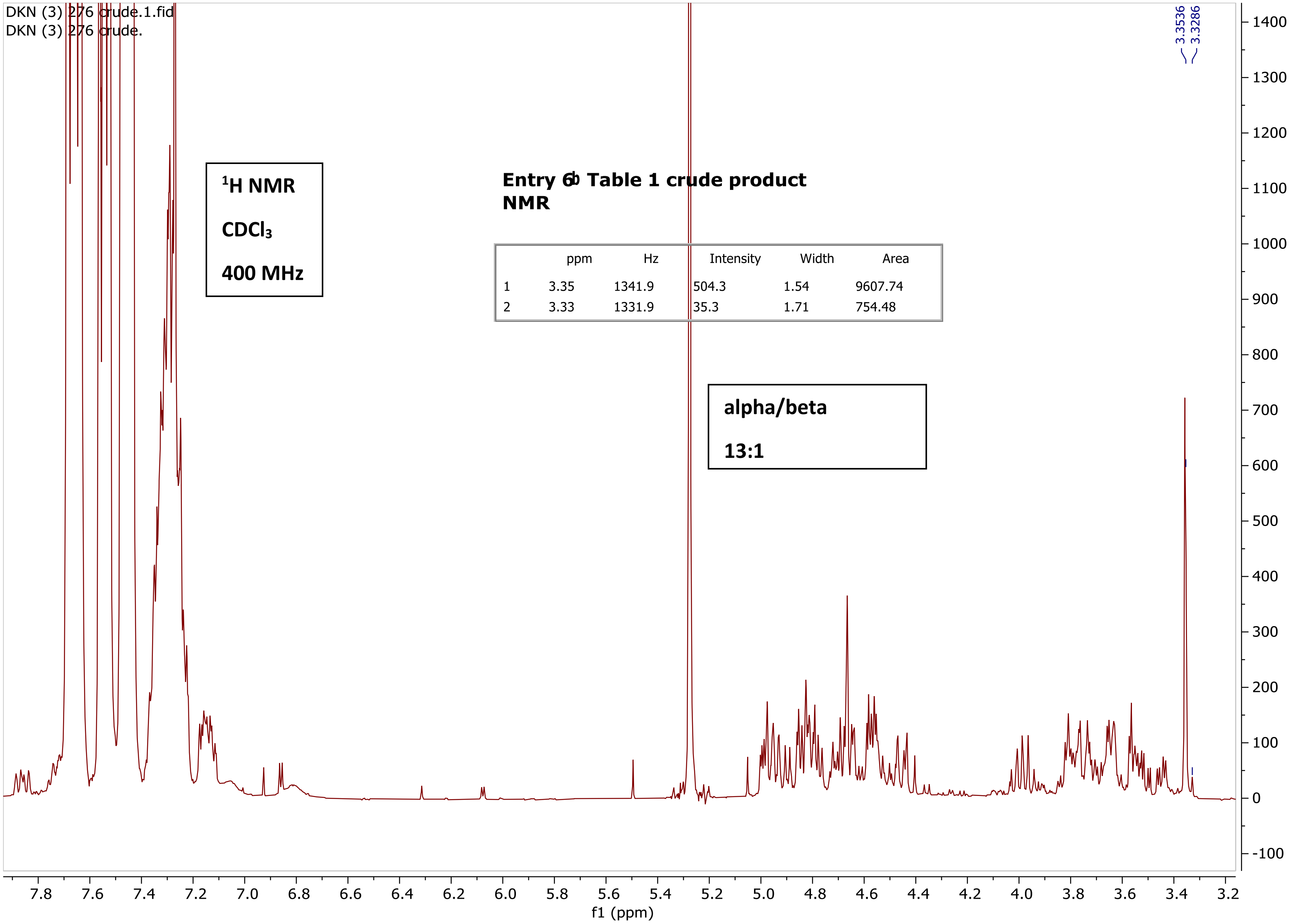

	ppm	Hz	Intensity	Width	Area
1	3.35	1341.9	504.3	1.54	9607.74
2	3.33	1331.9	35.3	1.71	754.48

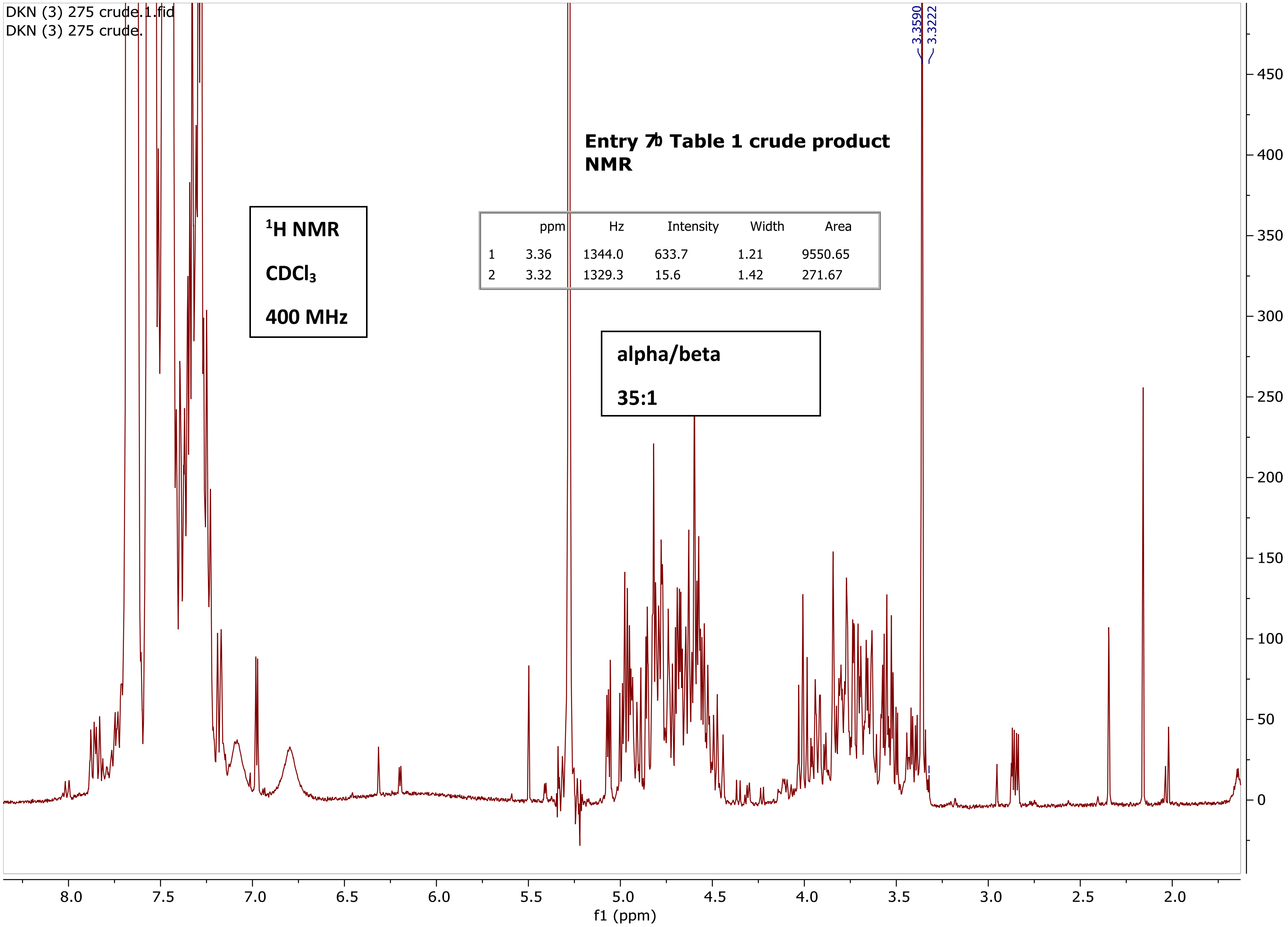

	ppm	Hz	Intensity	Width	Area
1	3.36	1344.0	633.7	1.21	9550.65
2	3.32	1329.3	15.6	1.42	271.67

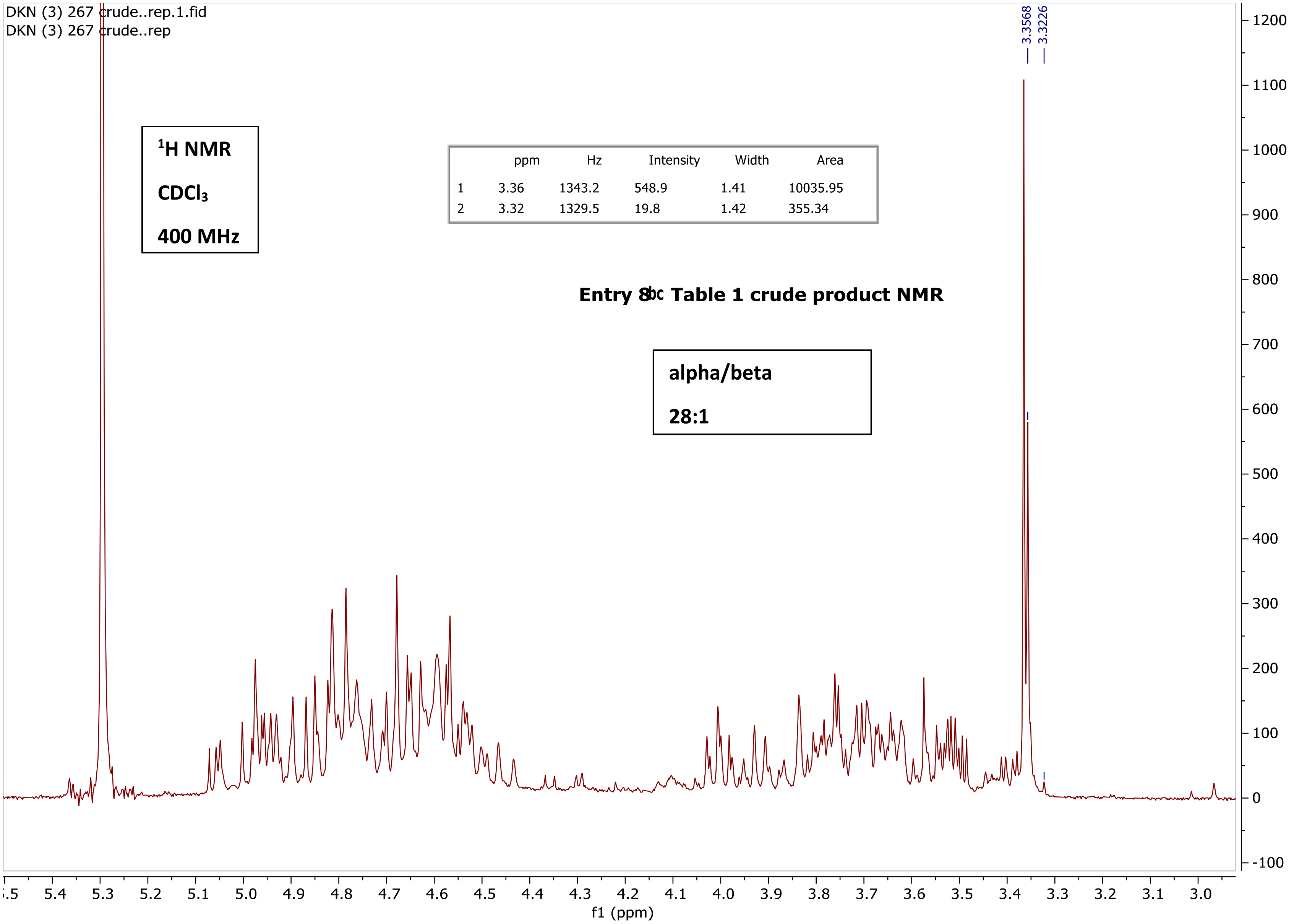

	ppm	Hz	Intensity	Width	Area
1	3.36	1343.2	548.9	1.41	10035.95
2	3.32	1329.5	19.8	1.42	355.34

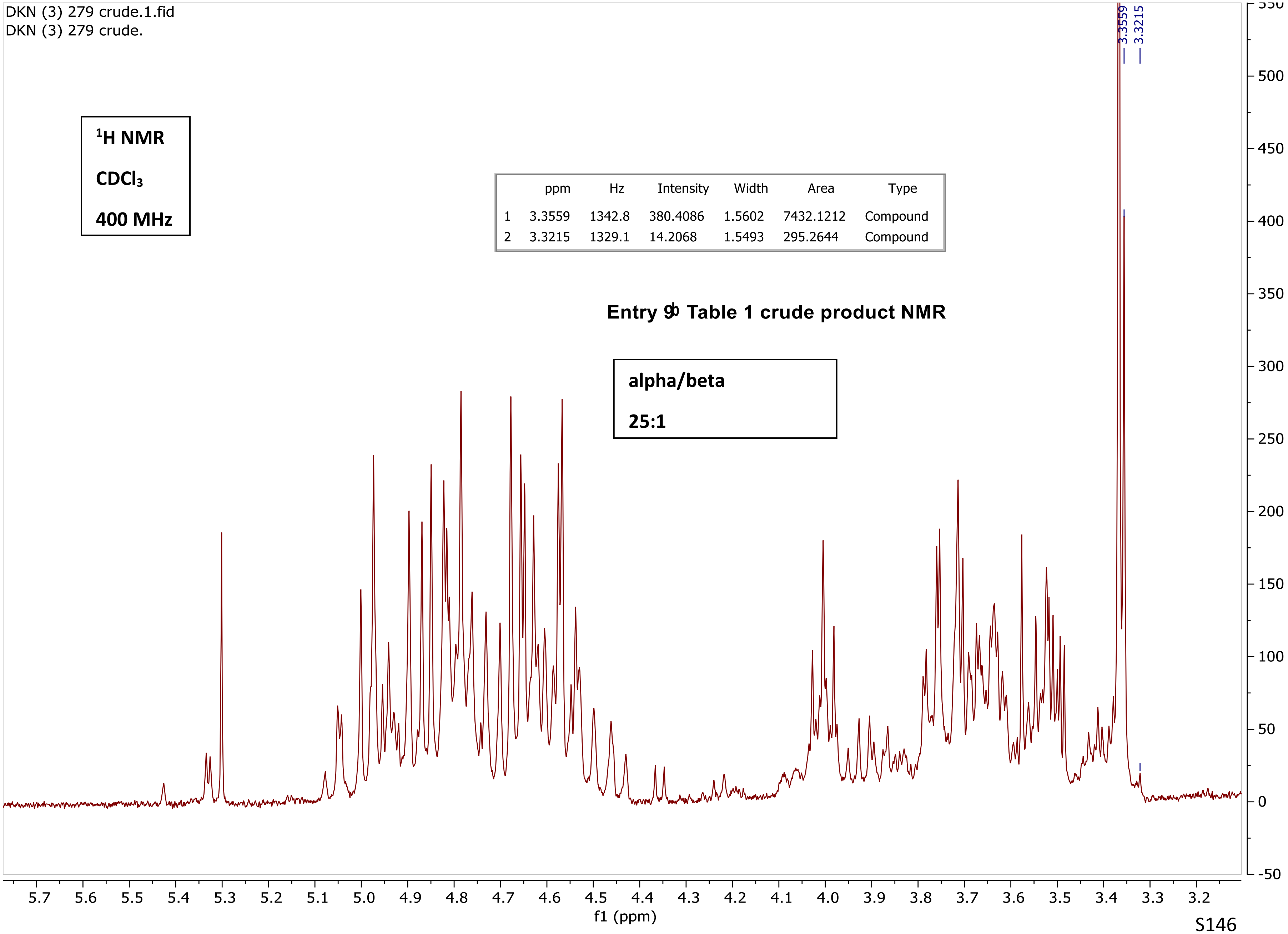

	ppm	Hz	Intensity	Width	Area	Type
1	3.3559	1342.8	380.4086	1.5602	7432.1212	Compound
2	3.3215	1329.1	14.2068	1.5493	295.2644	Compound

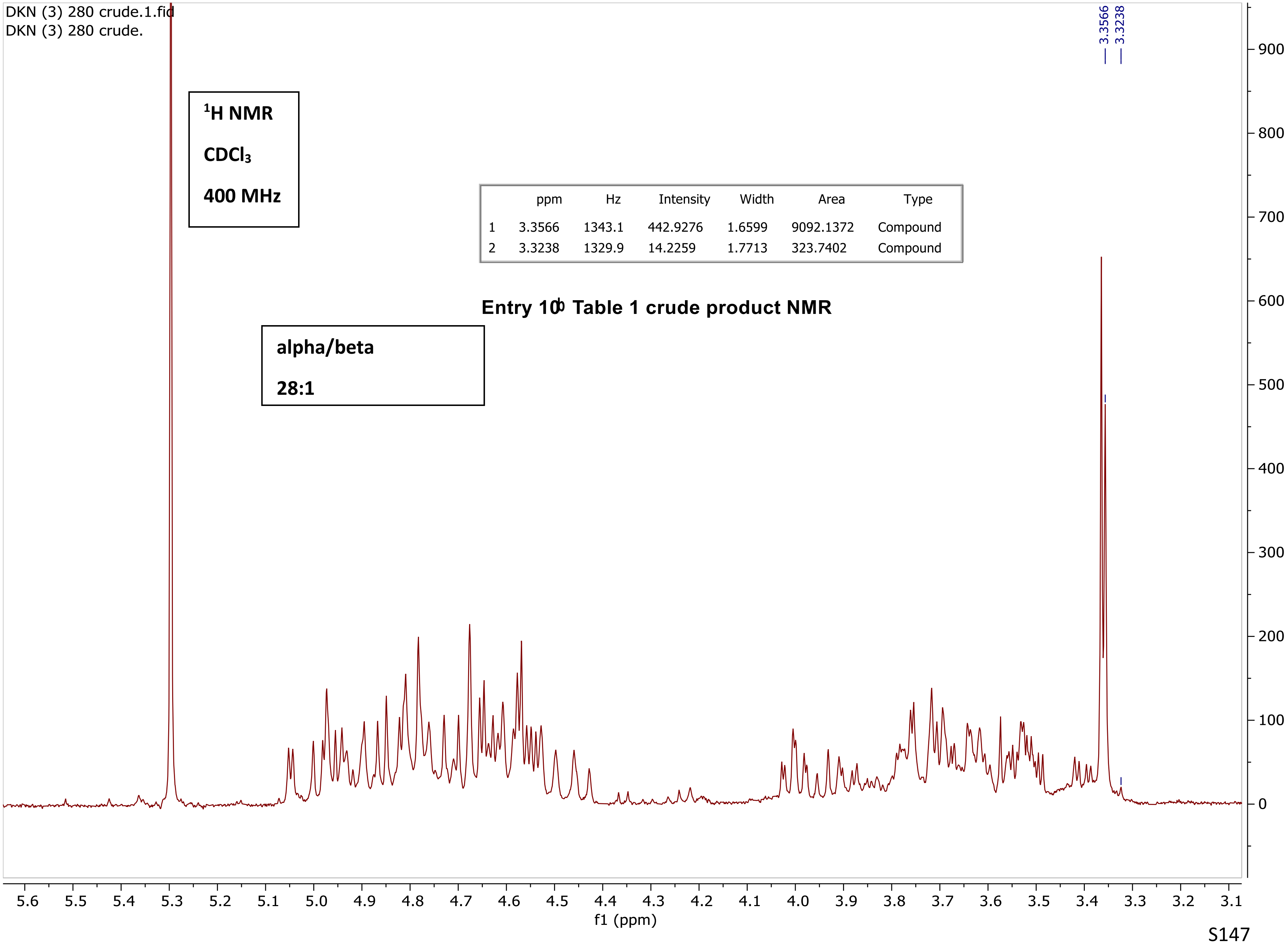

	ppm	Hz	Intensity	Width	Area	Type
1	3.3566	1343.1	442.9276	1.6599	9092.1372	Compound
2	3.3238	1329.9	14.2259	1.7713	323.7402	Compound

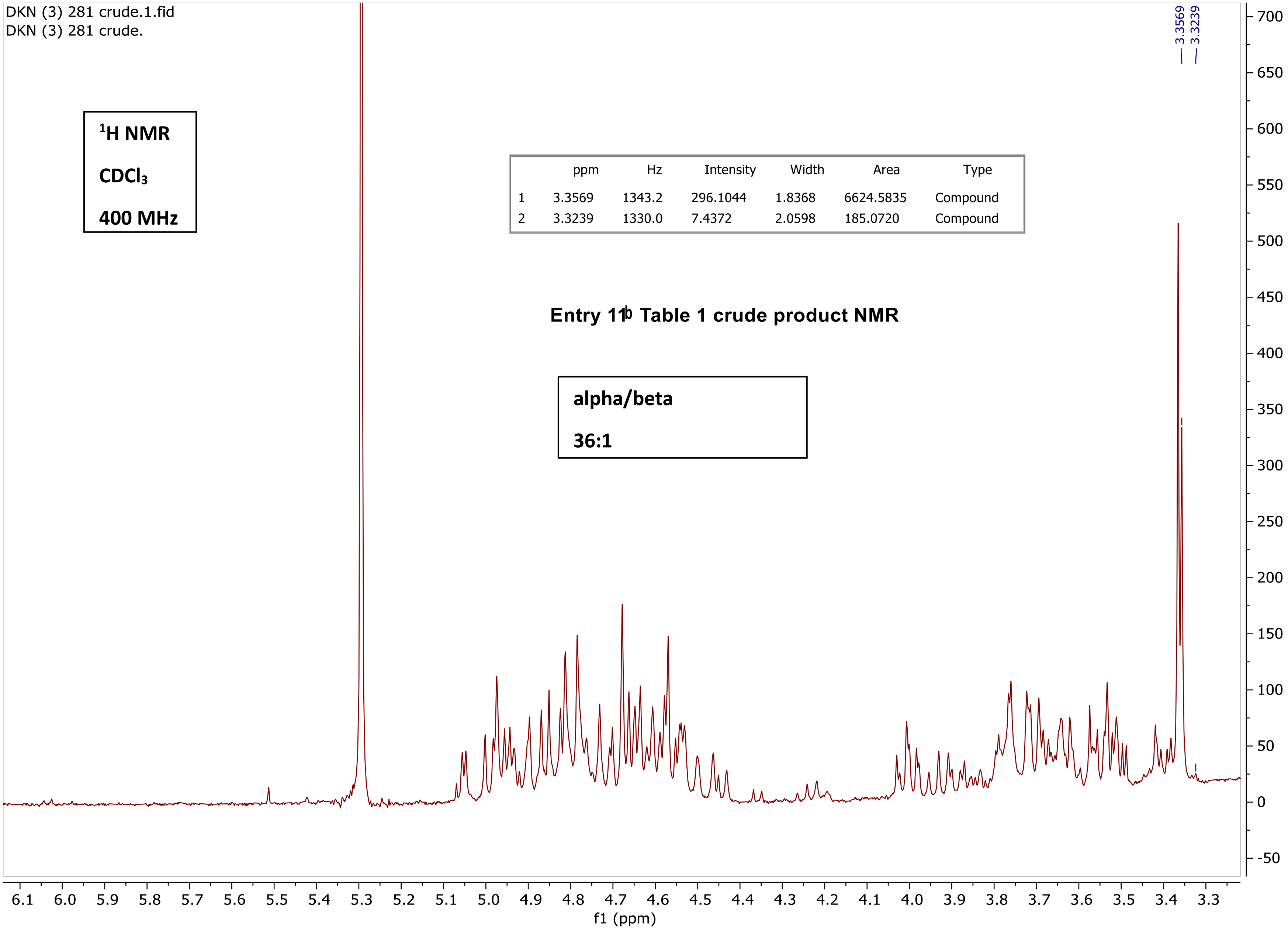

	ppm	Hz	Intensity	Width	Area	Type
1	3.3569	1343.2	296.1044	1.8368	6624.5835	Compound
2	3.3239	1330.0	7.4372	2.0598	185.0720	Compound

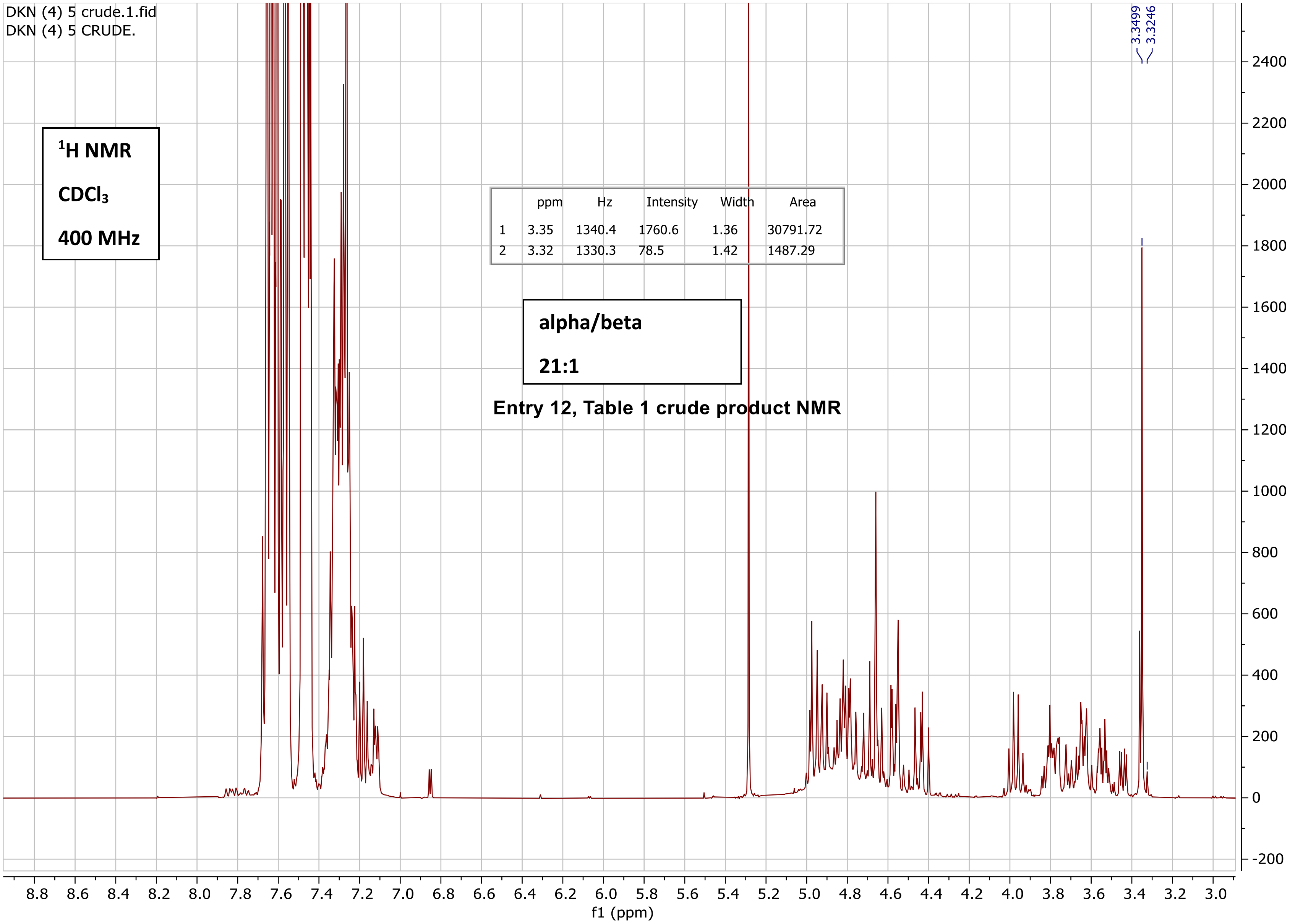

	ppm	Hz	Intensity	Width	Area
1	3.35	1340.4	1760.6	1.36	30791.72
2	3.32	1330.3	78.5	1.42	1487.29

Entry 12, Table 1 crude product NMR

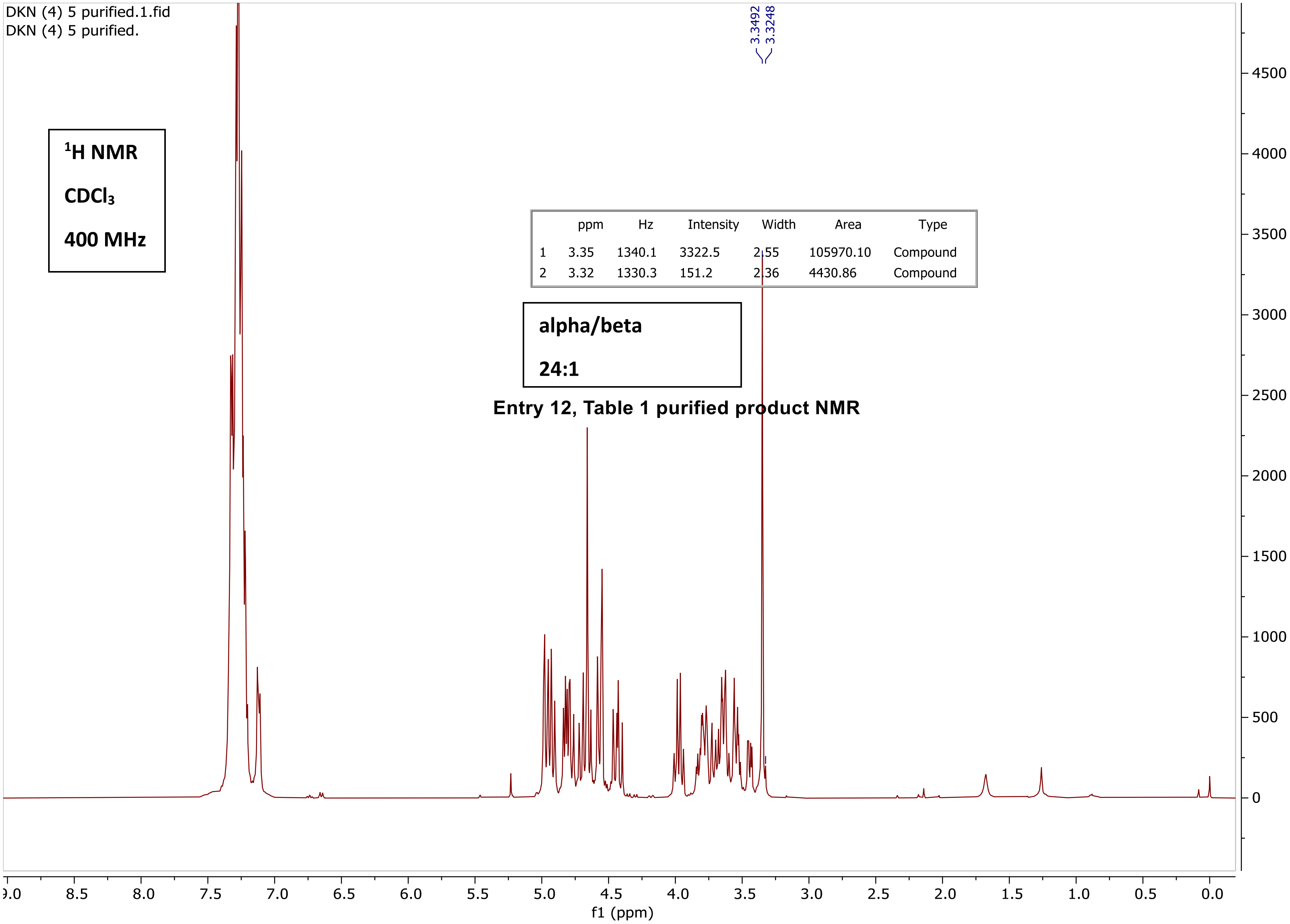

	ppm	Hz	Intensity	Width	Area	Type
1	3.35	1340.1	3322.5	2.55	105970.10	Compound
2	3.32	1330.3	151.2	2.36	4430.86	Compound

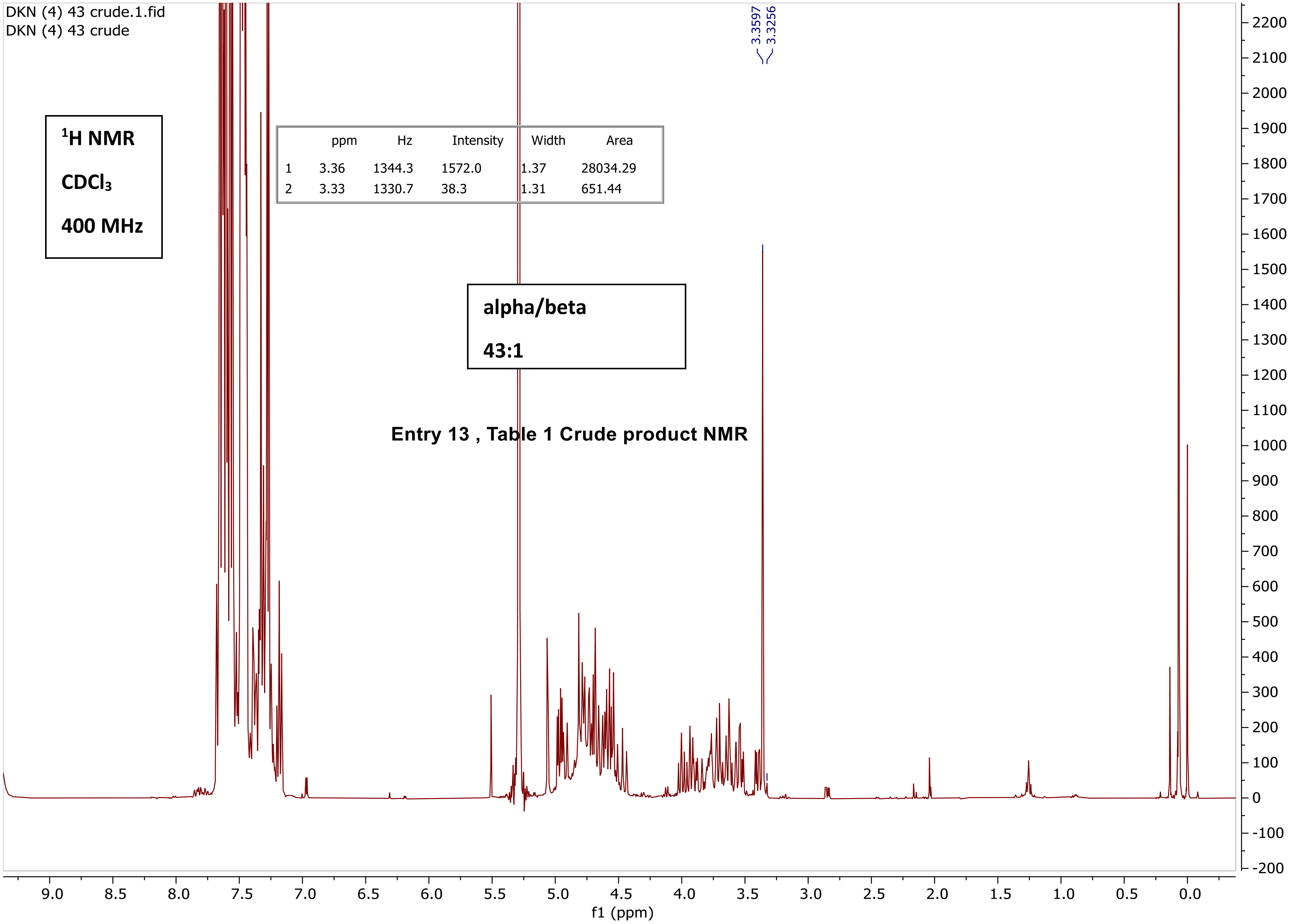
DKN (4) 43 crude.1.fid
DKN (4) 43 crude
3.3597
3.3256
^{1}H NMR
$CDCl_3$
400 MHz
	ppm	Hz	Intensity	Width	Area
1	3.36	1344.3	1572.0	1.37	28034.29
2	3.33	1330.7	38.3	1.31	651.44
alpha/beta
43:1
Entry 13 , Table 1 Crude product NMR
f1 (ppm)

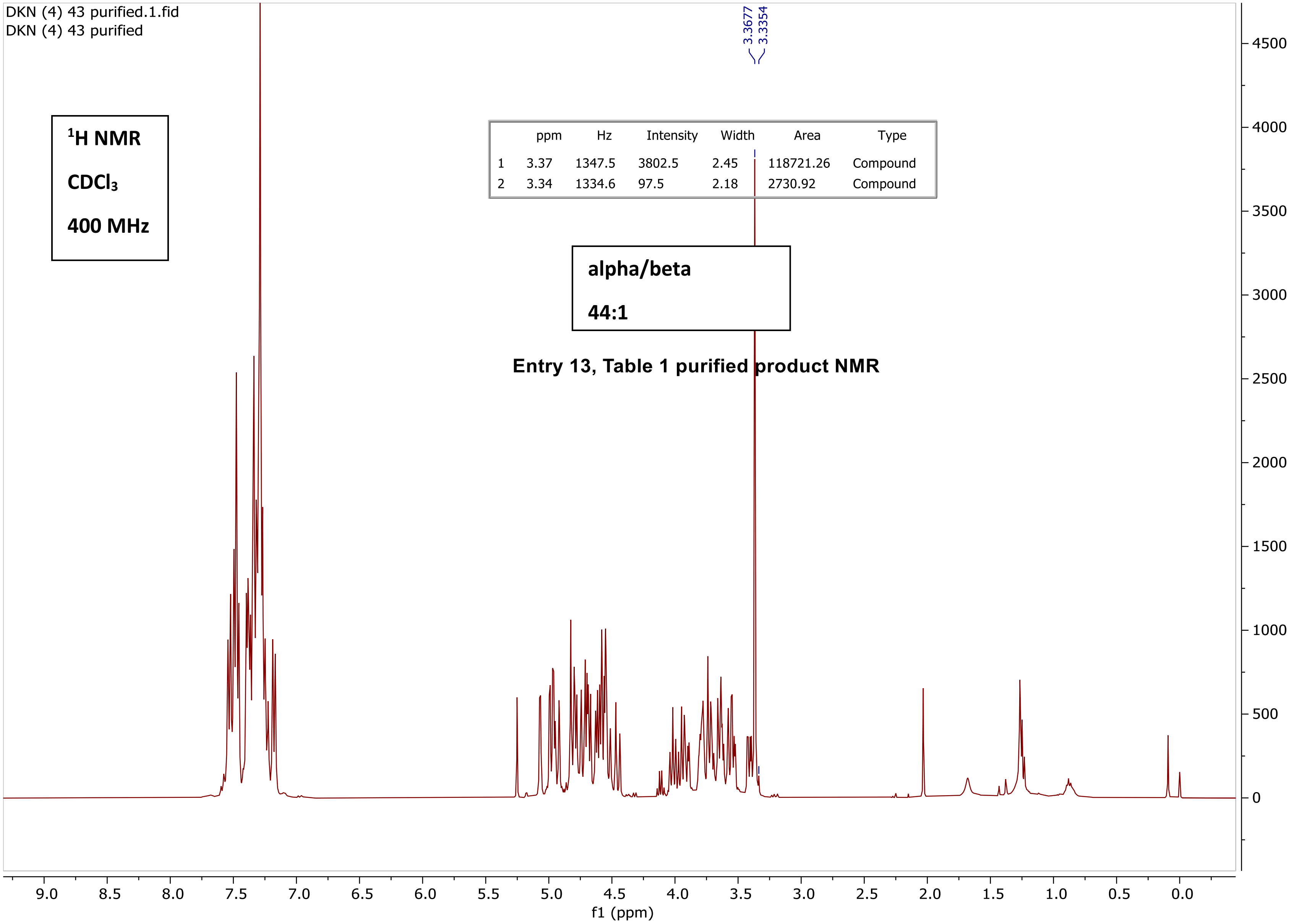

	ppm	Hz	Intensity	Width	Area	Type
1	3.37	1347.5	3802.5	2.45	118721.26	Compound
2	3.34	1334.6	97.5	2.18	2730.92	Compound

^1H NMR

$CDCl_3$

400 MHz

alpha/beta

44:1

Entry 13, Table 1 purified product NMR

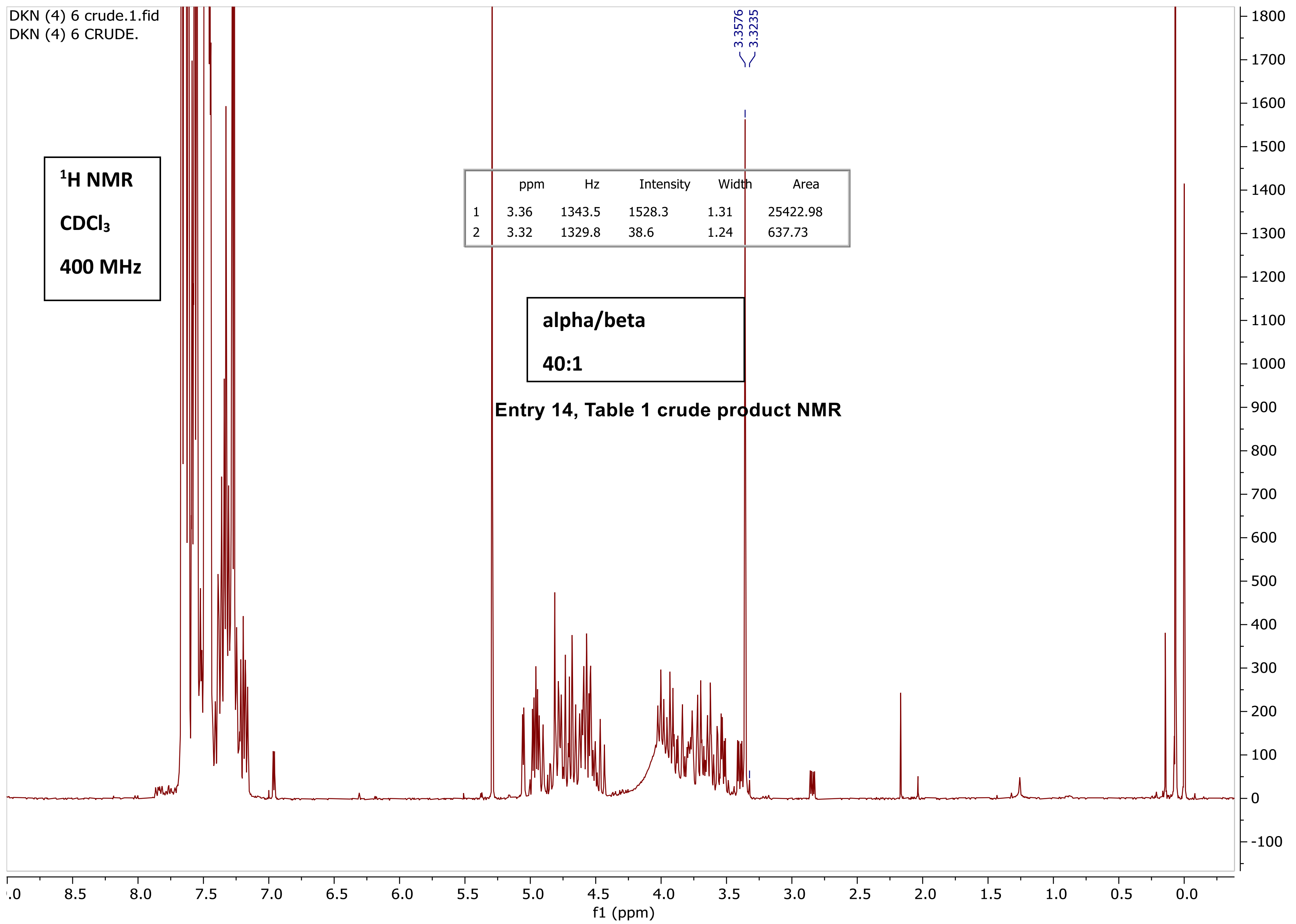
DKN (4) 6 crude.1.fid
DKN (4) 6 CRUDE.
^{1}H NMR
$CDCl_3$
400 MHz
3.3576
3.3235
ppm Hz Intensity Width Area
1 3.36 1343.5 1528.3 1.31 25422.98
2 3.32 1329.8 38.6 1.24 637.73
alpha/beta
40:1
Entry 14, Table 1 crude product NMR
f1 (ppm)

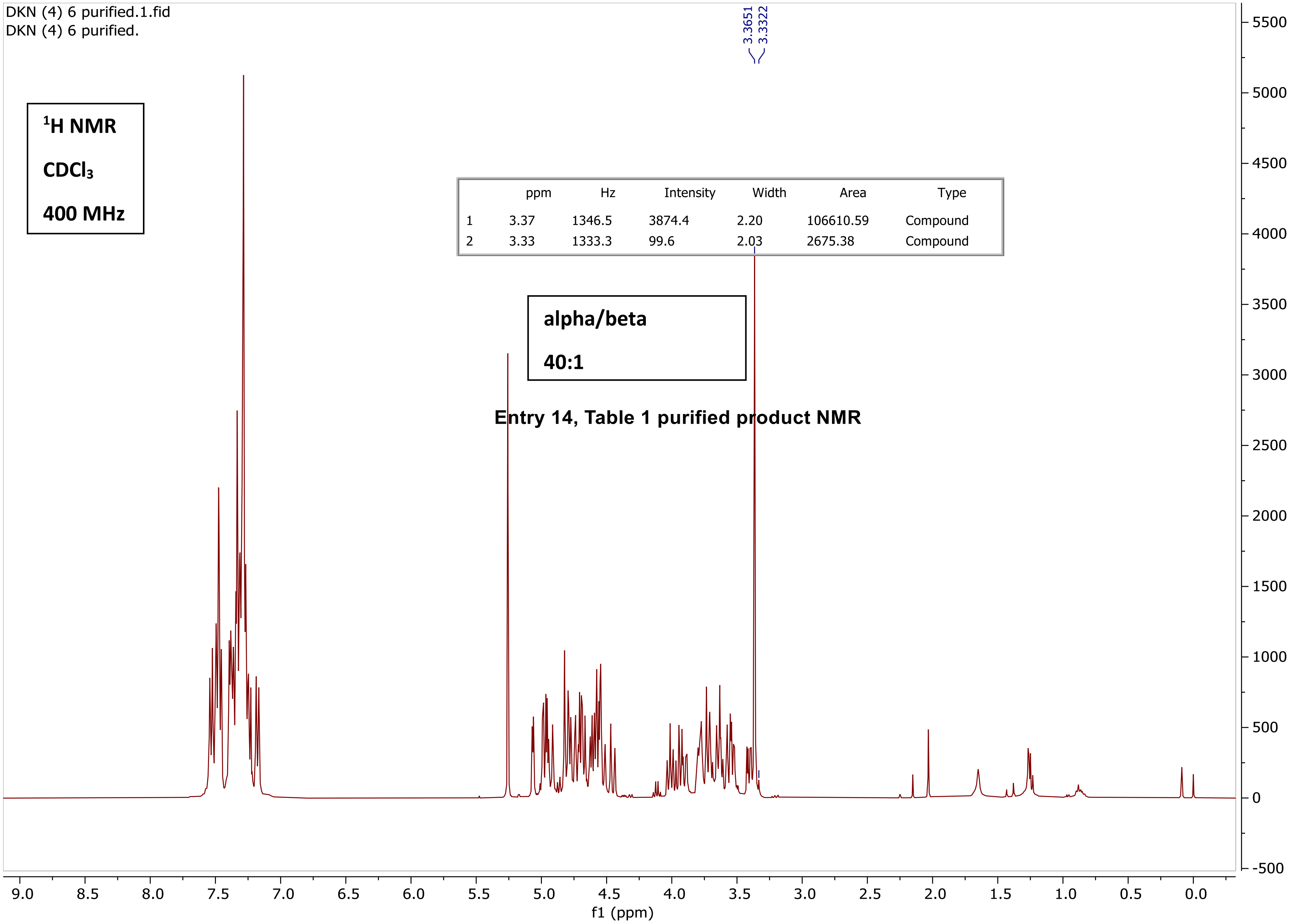

	ppm	Hz	Intensity	Width	Area	Type
1	3.37	1346.5	3874.4	2.20	106610.59	Compound
2	3.33	1333.3	99.6	2.03	2675.38	Compound

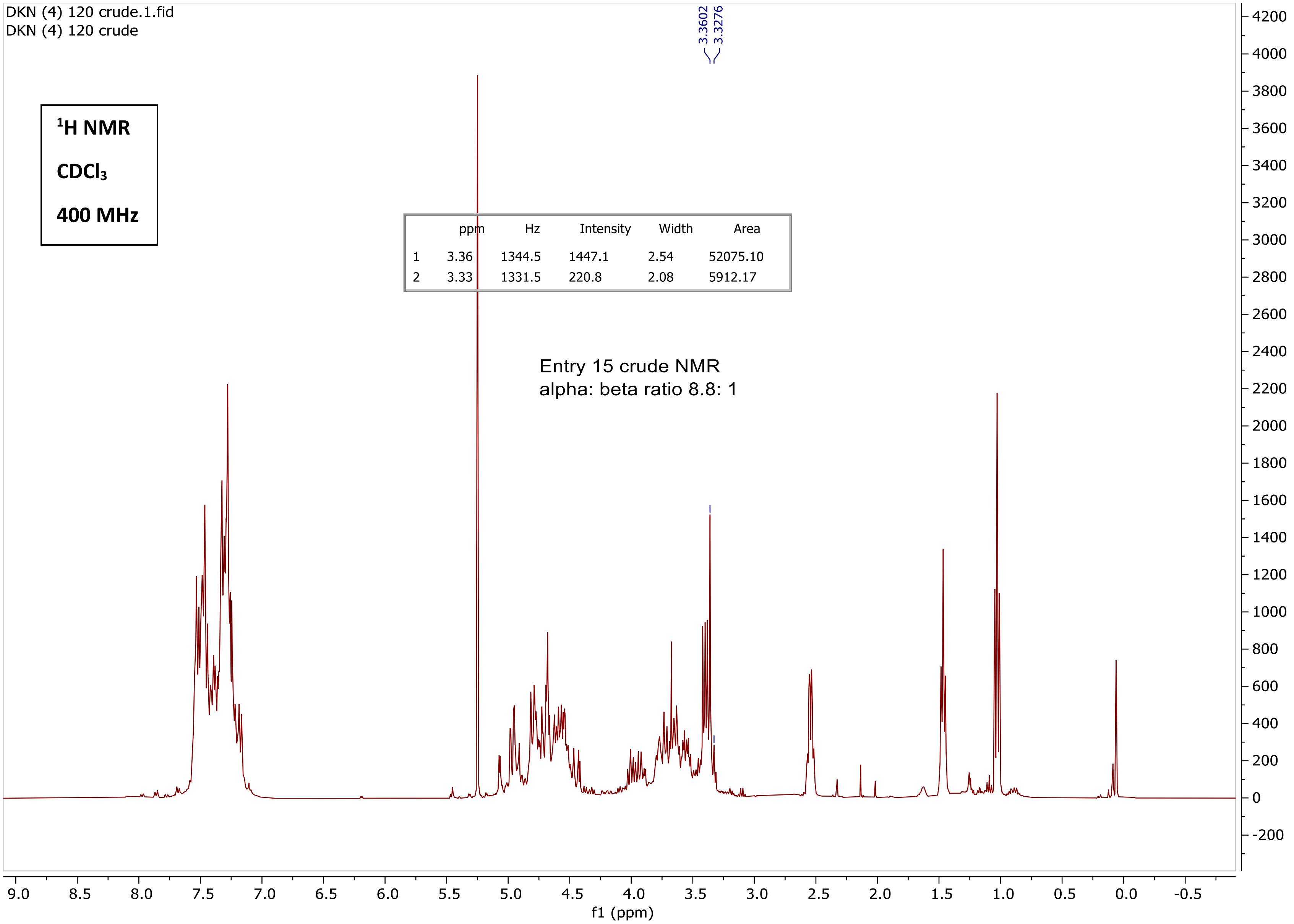

	ppm	Hz	Intensity	Width	Area
1	3.36	1344.5	1447.1	2.54	52075.10
2	3.33	1331.5	220.8	2.08	5912.17

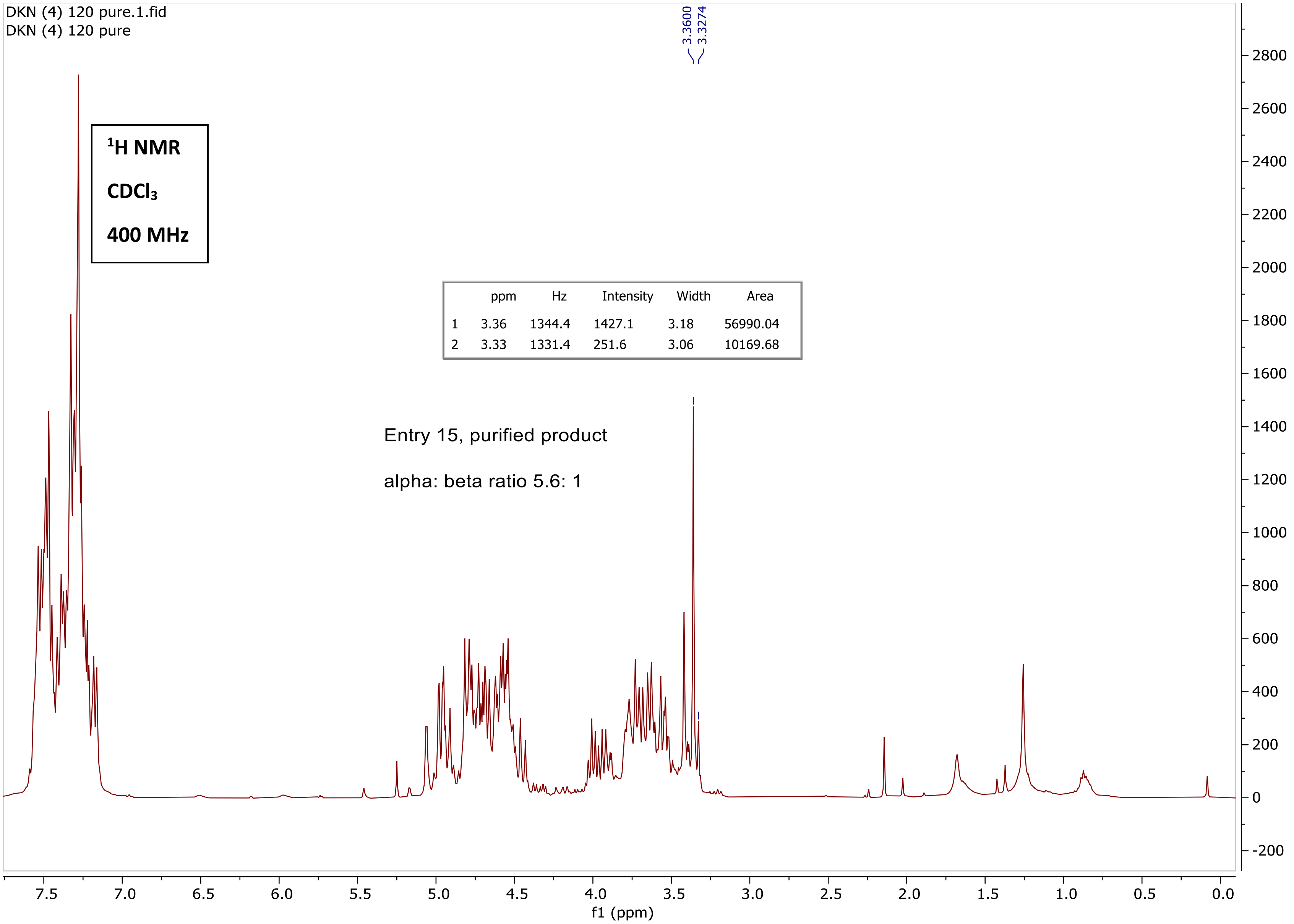

	ppm	Hz	Intensity	Width	Area
1	3.36	1344.4	1427.1	3.18	56990.04
2	3.33	1331.4	251.6	3.06	10169.68

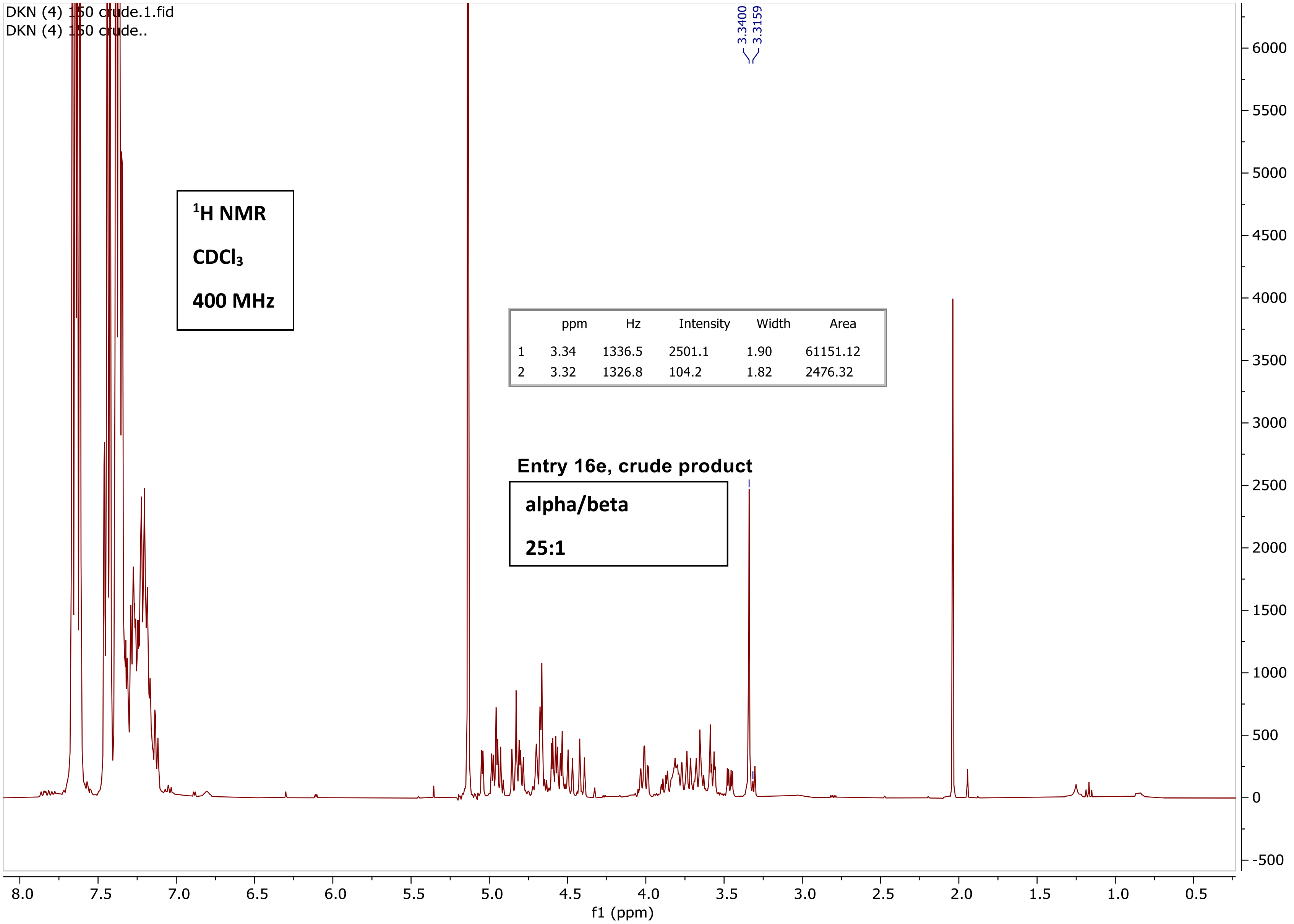

	ppm	Hz	Intensity	Width	Area
1	3.34	1336.5	2501.1	1.90	61151.12
2	3.32	1326.8	104.2	1.82	2476.32

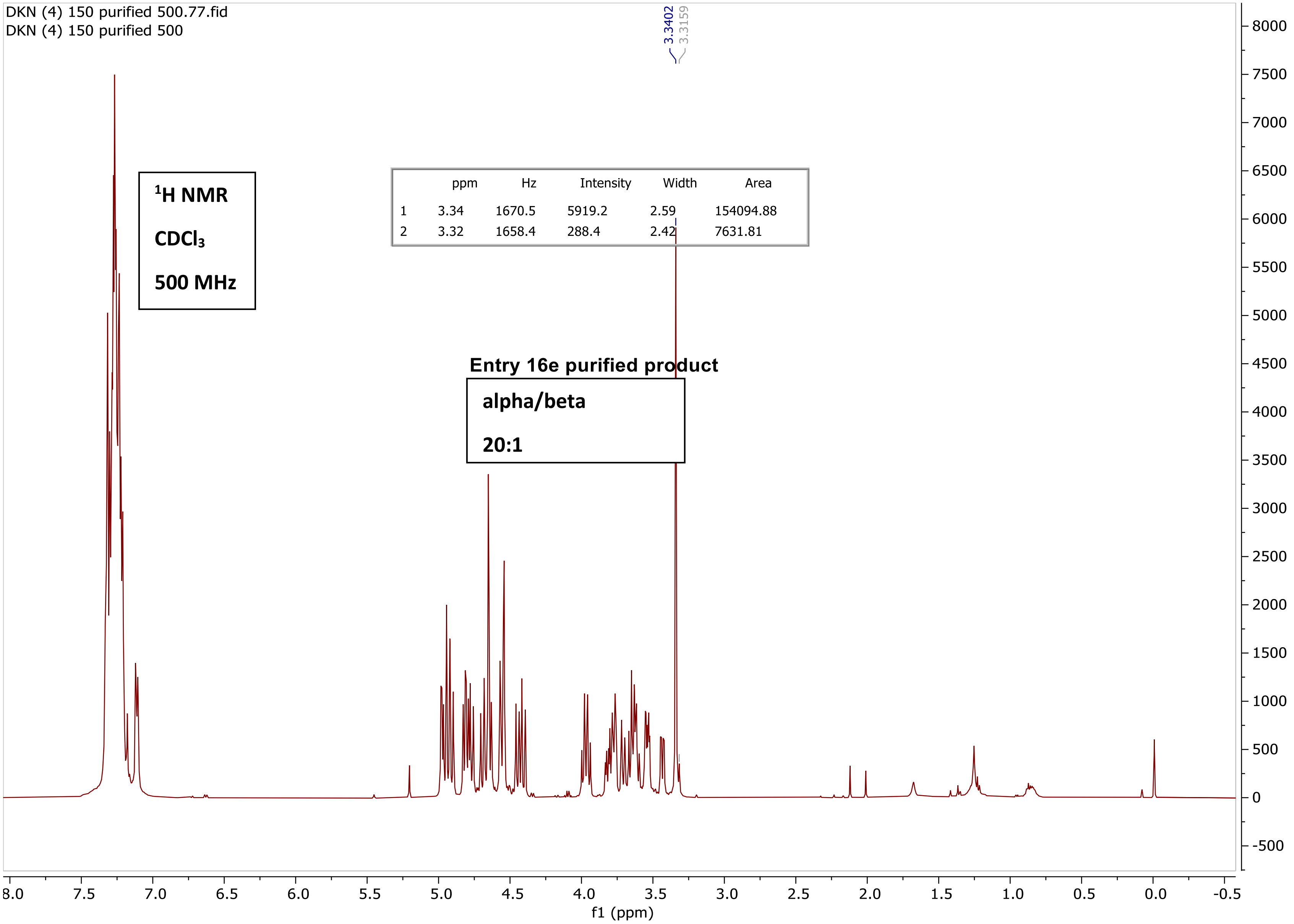

DKN (4) 150 purified 500.77.fid
DKN (4) 150 purified 500
3.3402
3.3159
1H NMR
CDCl3
500 MHz
ppm Hz Intensity Width Area
1 3.34 1670.5 5919.2 2.59 154094.88
2 3.32 1658.4 288.4 2.42 7631.81
Entry 16e purified product
alpha/beta
20:1
f1 (ppm)

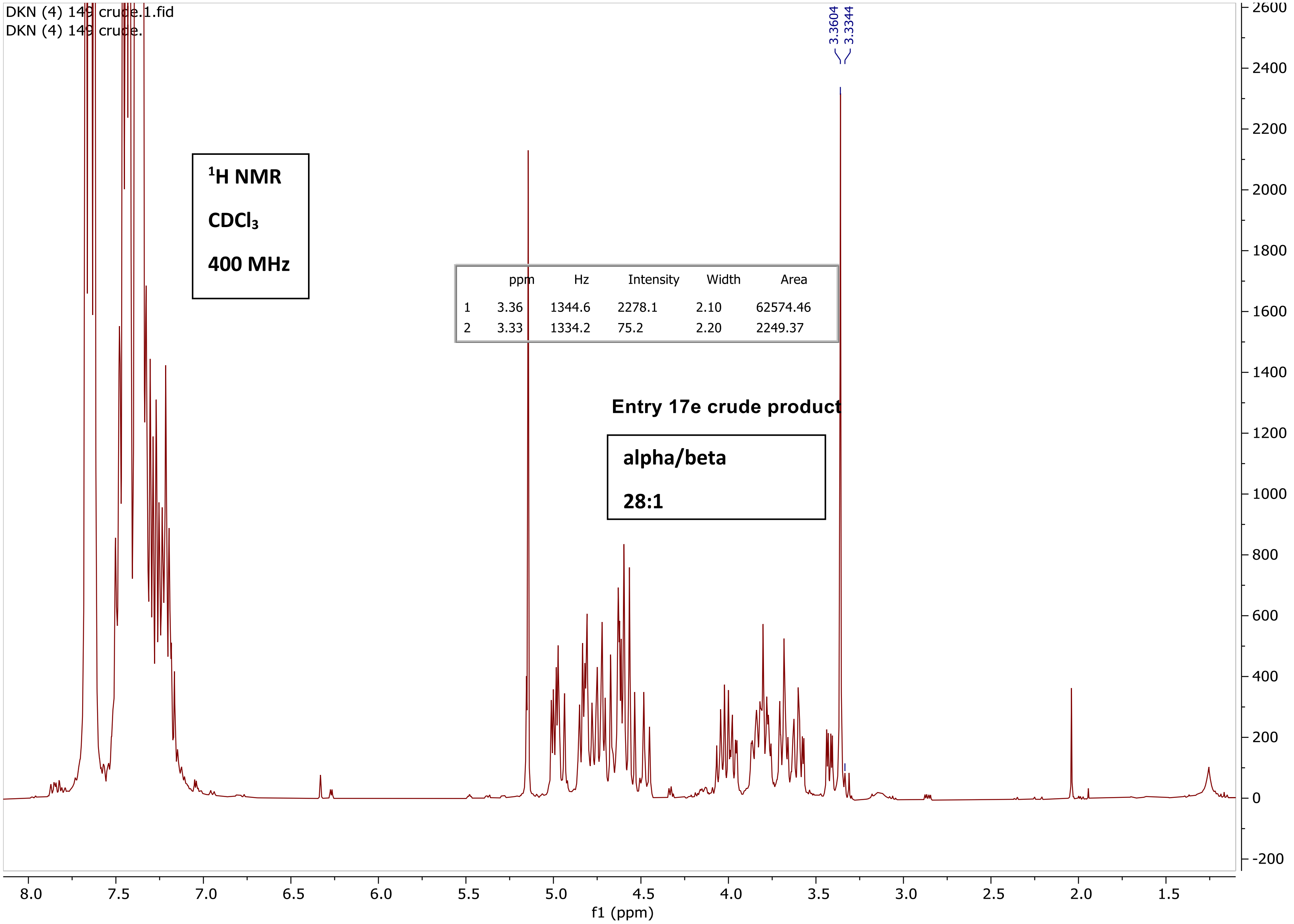
DKN (4) 149 crude.1.fid
DKN (4) 149 crude.
3.3604
3.3344
1H NMR
CDCl3
400 MHz
ppm Hz Intensity Width Area
1 3.36 1344.6 2278.1 2.10 62574.46
2 3.33 1334.2 75.2 2.20 2249.37
Entry 17e crude product
alpha/beta
28:1
f1 (ppm)

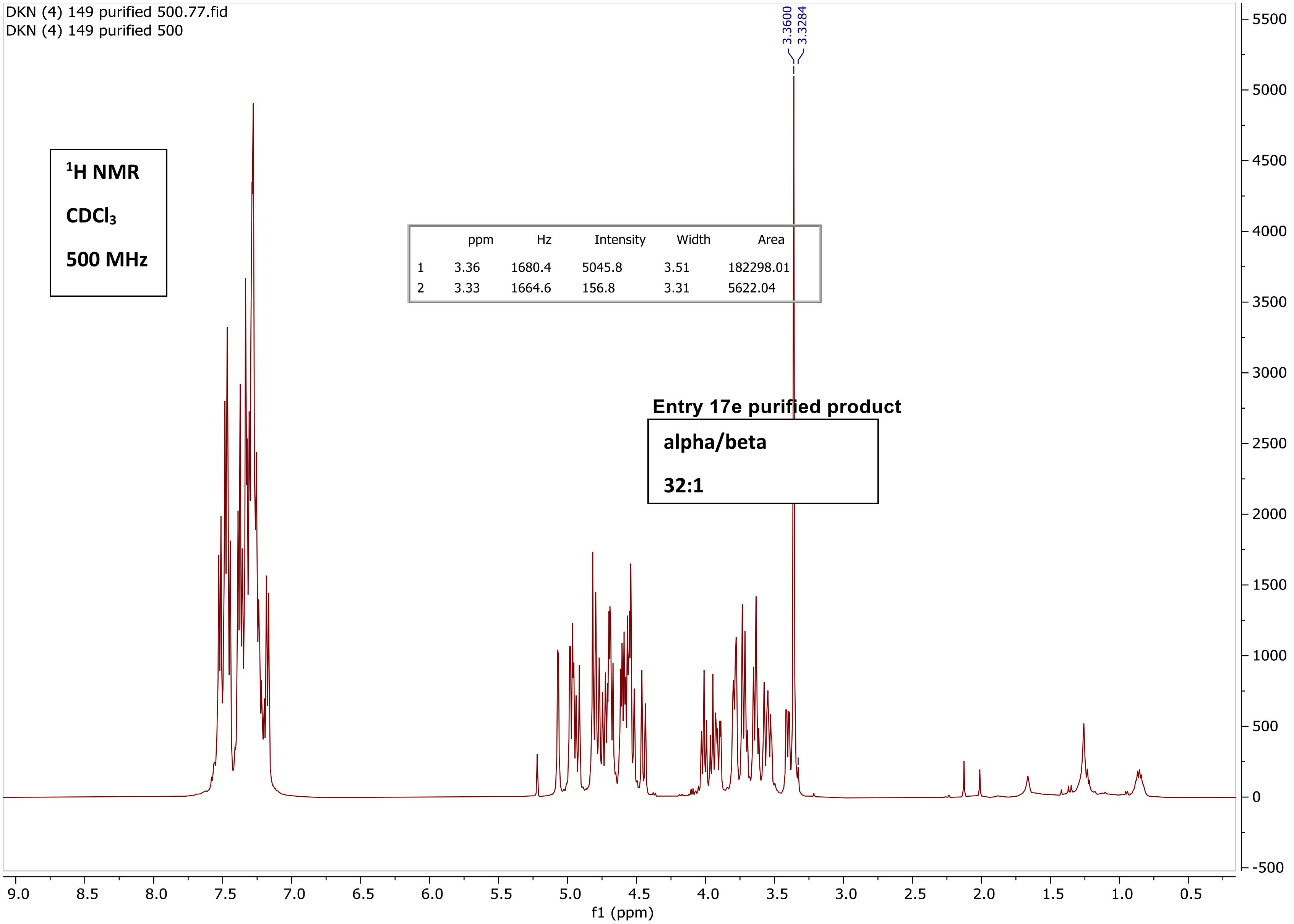

	ppm	Hz	Intensity	Width	Area
1	3.36	1680.4	5045.8	3.51	182298.01
2	3.33	1664.6	156.8	3.31	5622.04

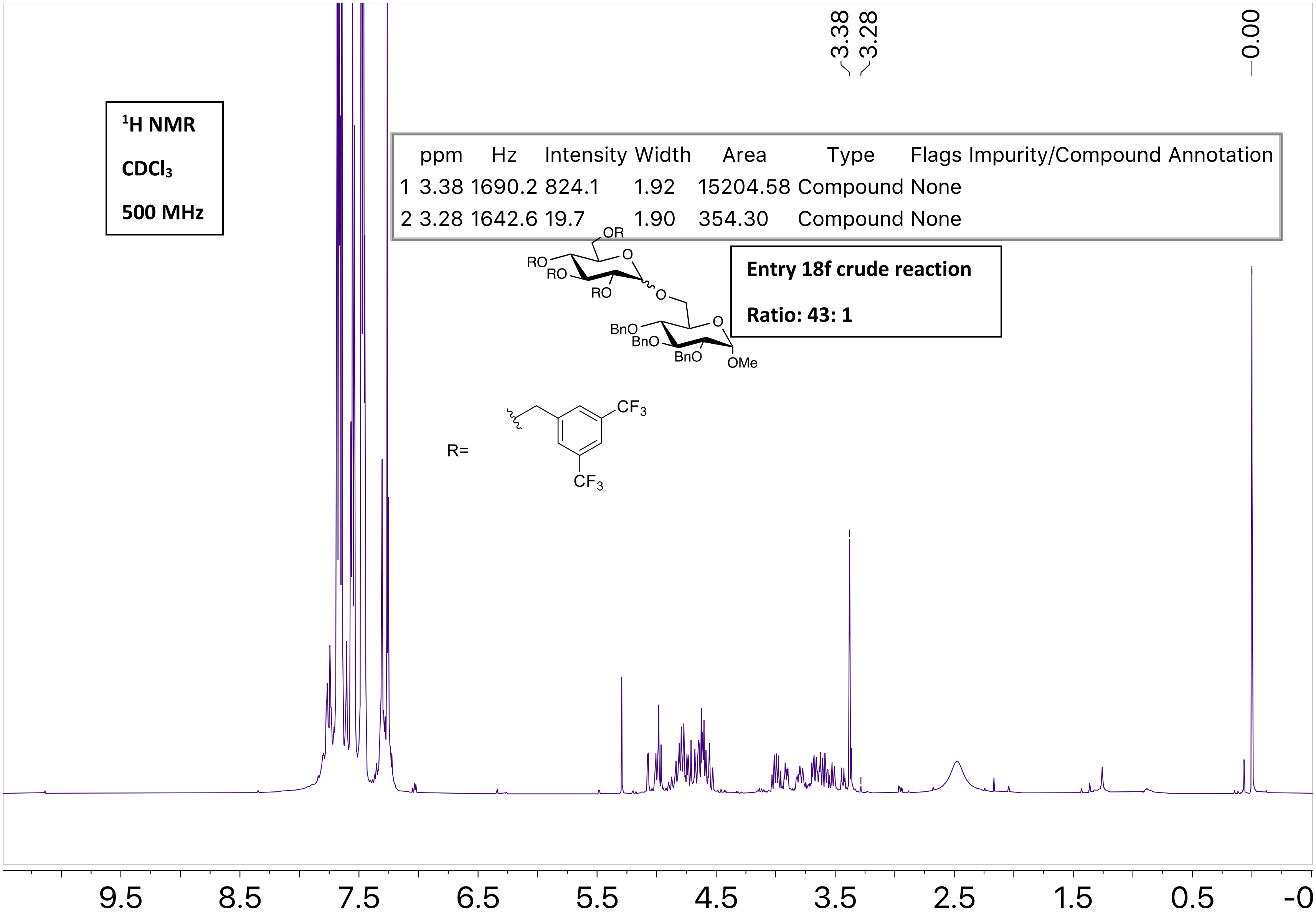

^{1}H NMR

$CDCl_3$

500 MHz

	ppm	Hz	Intensity	Width	Area	Type	Flags	Impurity/Compound	Annotation
1	3.38	1690.2	824.1	1.92	15204.58	Compound	None		
2	3.28	1642.6	19.7	1.90	354.30	Compound	None		

Entry 18f crude reaction

Ratio: 43: 1

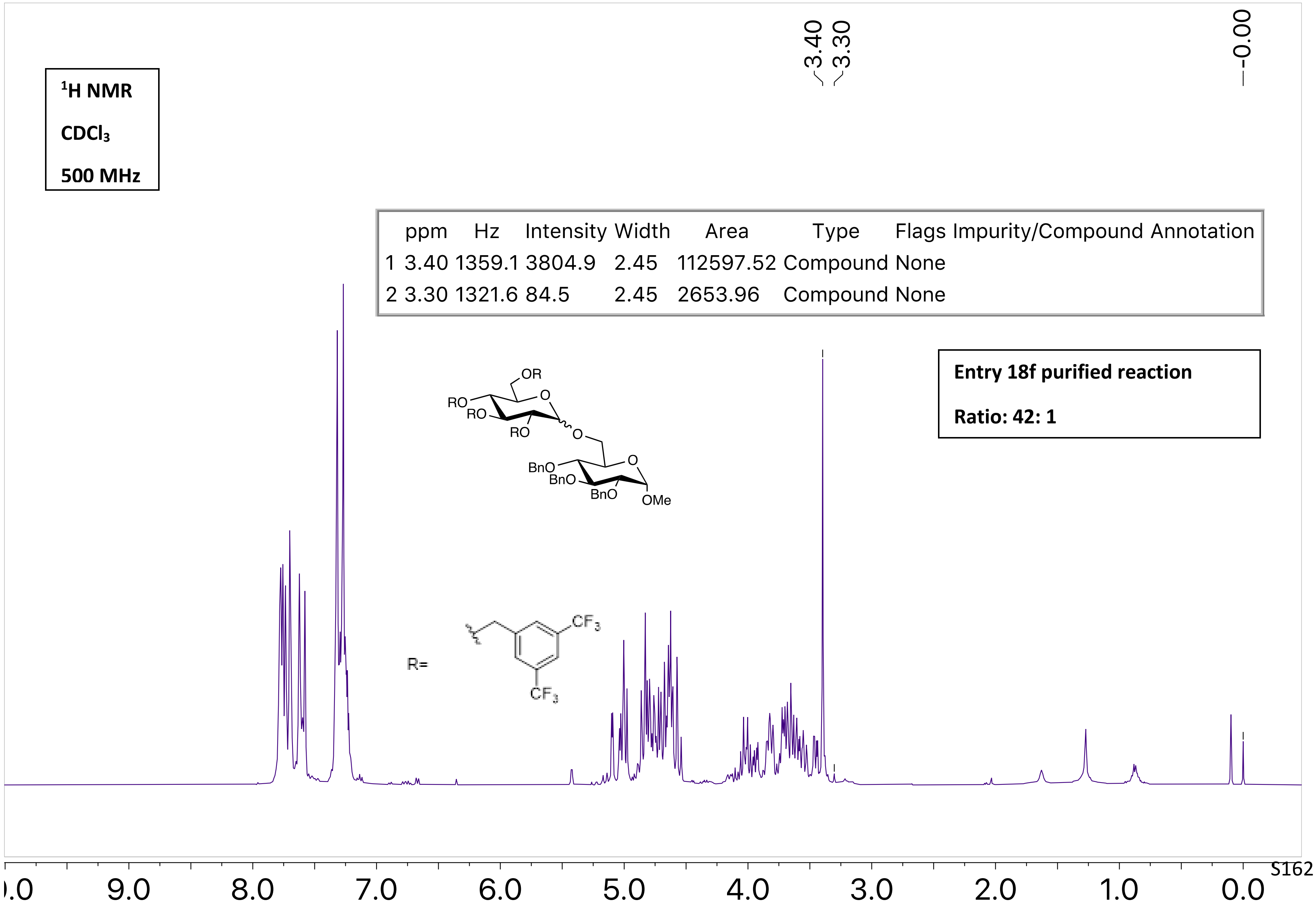

	ppm	Hz	Intensity	Width	Area	Type	Flags	Impurity/Compound	Annotation
1	3.40	1359.1	3804.9	2.45	112597.52	Compound	None		
2	3.30	1321.6	84.5	2.45	2653.96	Compound	None		

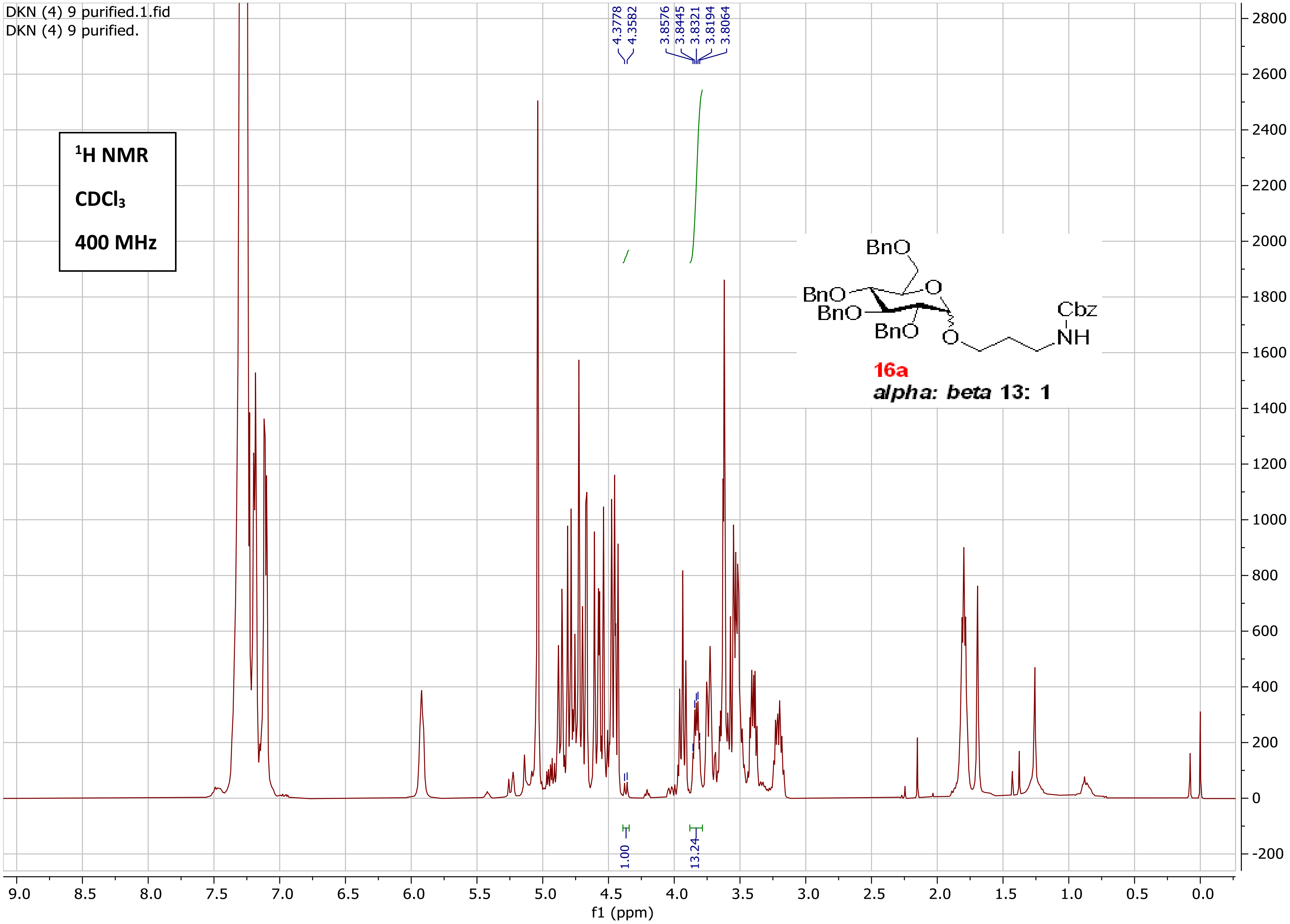
DKN (4) 9 purified.1.fid
DKN (4) 9 purified.
1H NMR
CDCl3
400 MHz
4.3778
4.3582
3.8576
3.8445
3.8321
3.8194
3.8064
BnO
BnO
BnO
BnO
O
O
Cbz
NH
16a
alpha: beta 13: 1
1.00
13.24
f1 (ppm)

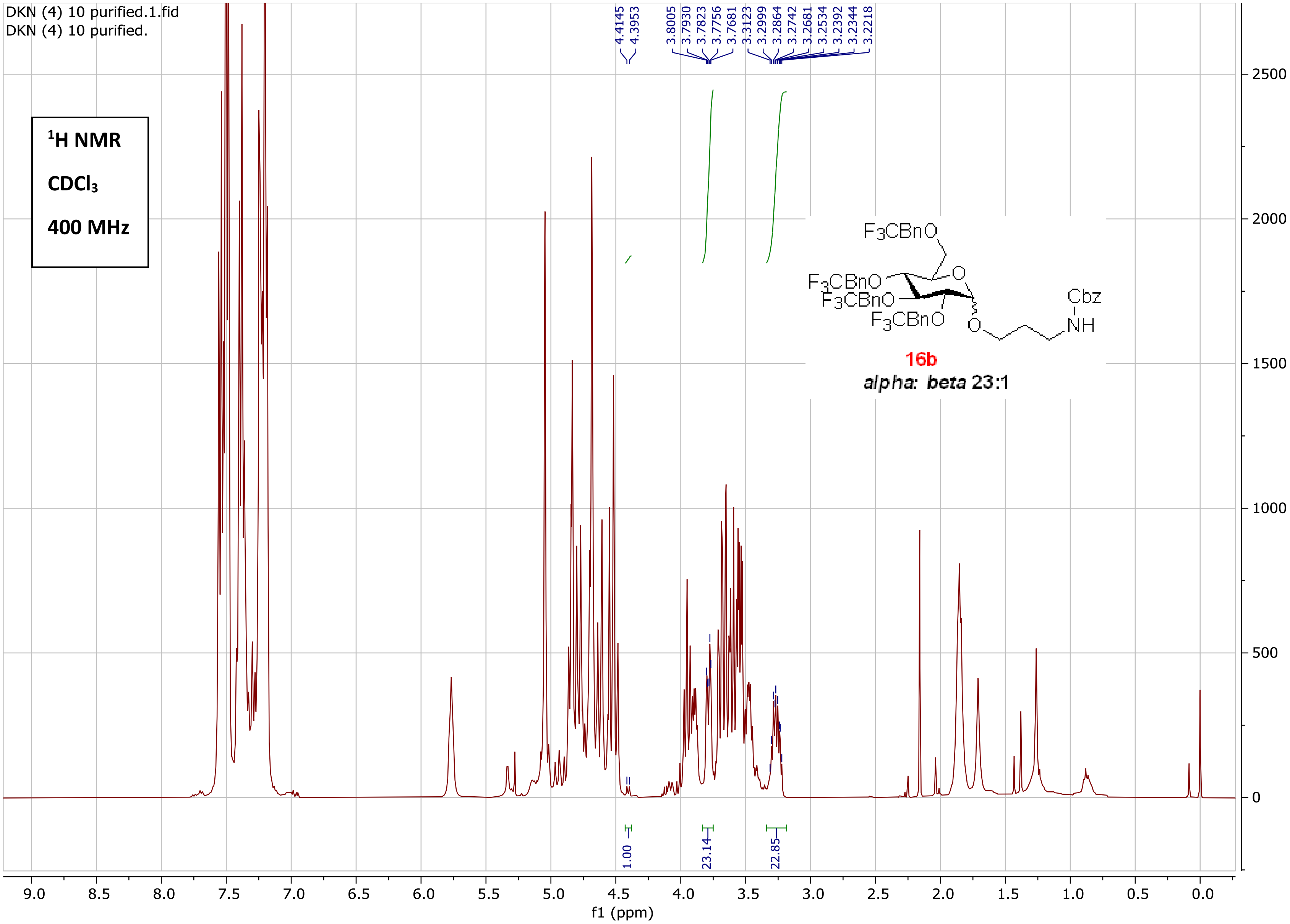

DKN (4) 10 purified.1.fid
DKN (4) 10 purified.
^1H NMR
$CDCl_3$
400 MHz
4.4145
4.3953
3.8005
3.7930
3.7823
3.7756
3.7681
3.3123
3.2999
3.2864
3.2742
3.2681
3.2534
3.2392
3.2344
3.2218
F3CBnO
F3CBnO
F3CBnO
F3CBnO
O
O
Cbz
NH
16b
alpha: beta 23:1
1.00
23.14
22.85
f1 (ppm)

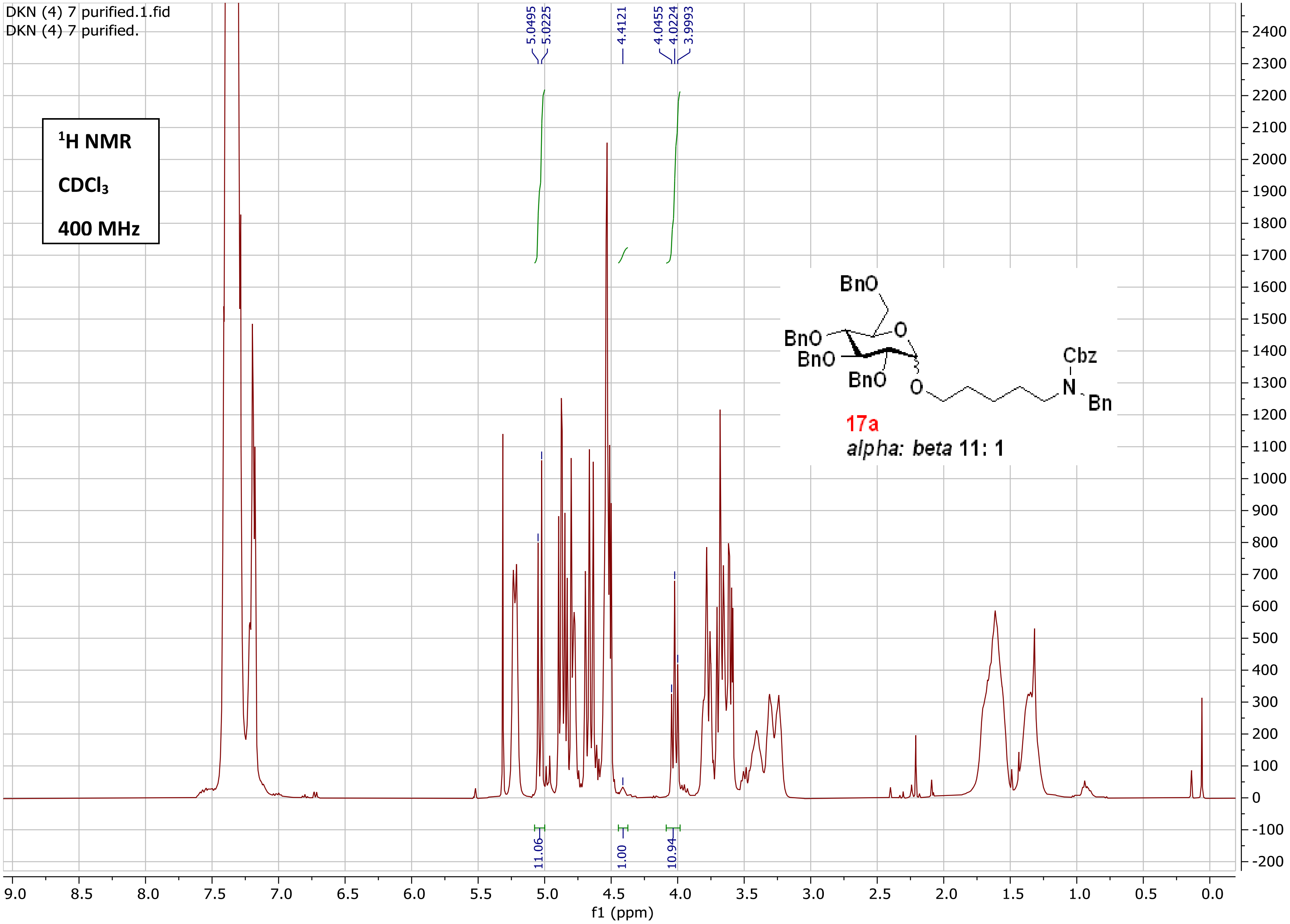

DKN (4) 7 purified.1.fid
DKN (4) 7 purified.
^{1}H NMR
CDCl$_3$
400 MHz
5.0495
5.0225
4.4121
4.0455
4.0224
3.9993
BnO
O
BnO
BnO
BnO
O
Cbz
N
Bn
17a
alpha: beta 11: 1
11.06
1.00
10.94
f1 (ppm)

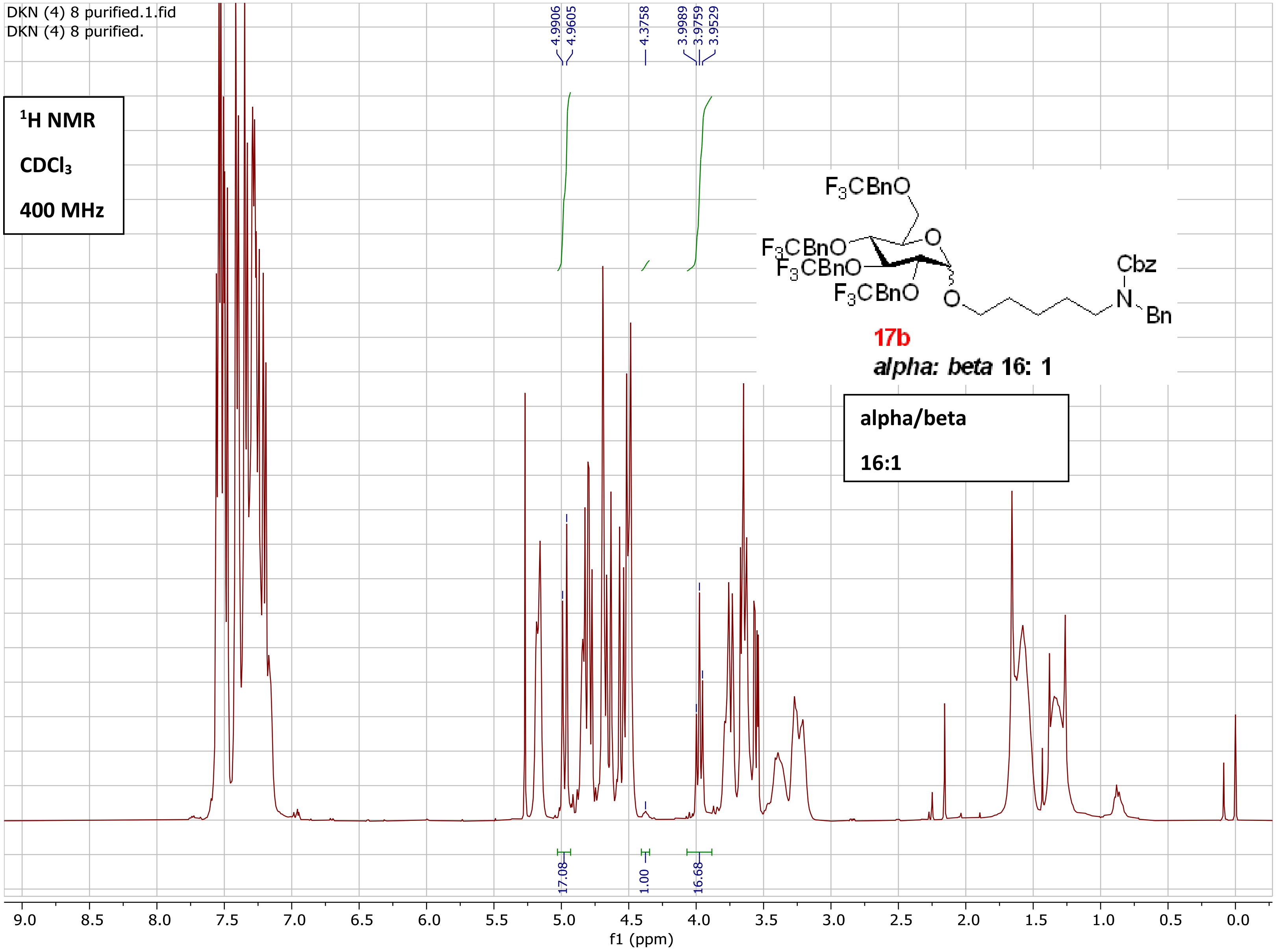
DKN (4) 8 purified.1.fid
DKN (4) 8 purified.
1H NMR
CDCl3
400 MHz
4.9906
4.9605
4.3758
3.9989
3.9759
3.9529
F3CBnO
F3CBnO
F3CBnO
F3CBnO
O
O
Cbz
N
Bn
17b
alpha: beta 16: 1
alpha/beta
16:1
17.08
1.00
16.68
f1 (ppm)

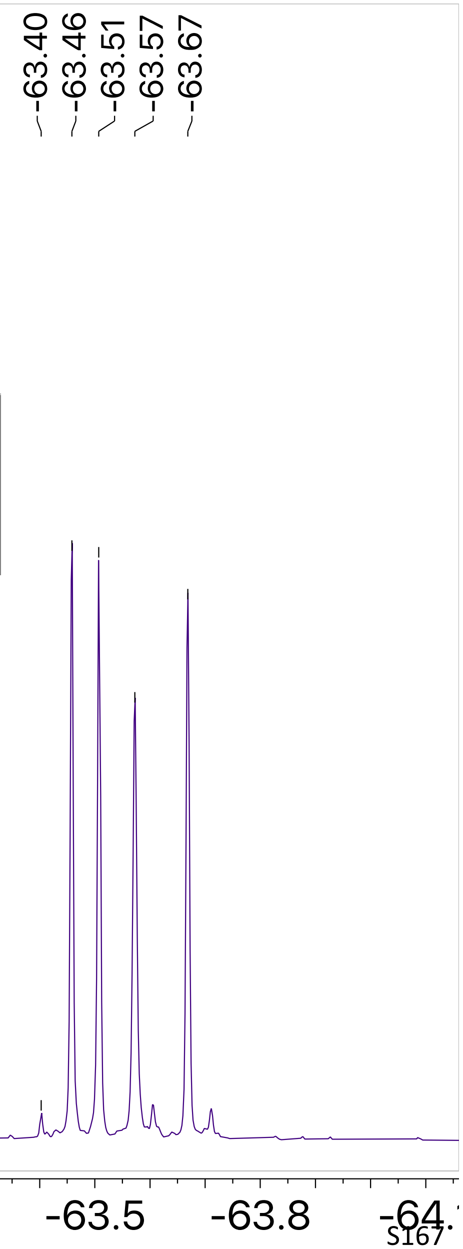

	ppm	Hz	Intensity	Width	Area	Type	Flags	Impurity/ Compound	Annotation
1	-63.40	-29836.9	1627.4	2.60	6014.51	Compound	None		
2	-63.46	-29863.1	45223.4	2.55	187244.18	Compound	None		

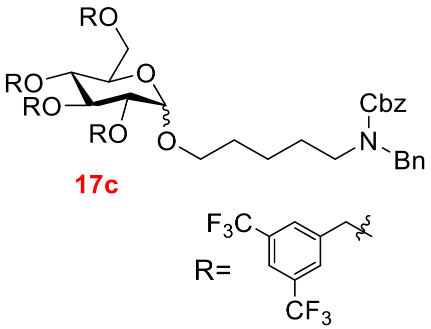

17c

^{19}F NMR *alpha: beta* 31: 1

**$CDCl_3$,
471 MHz**

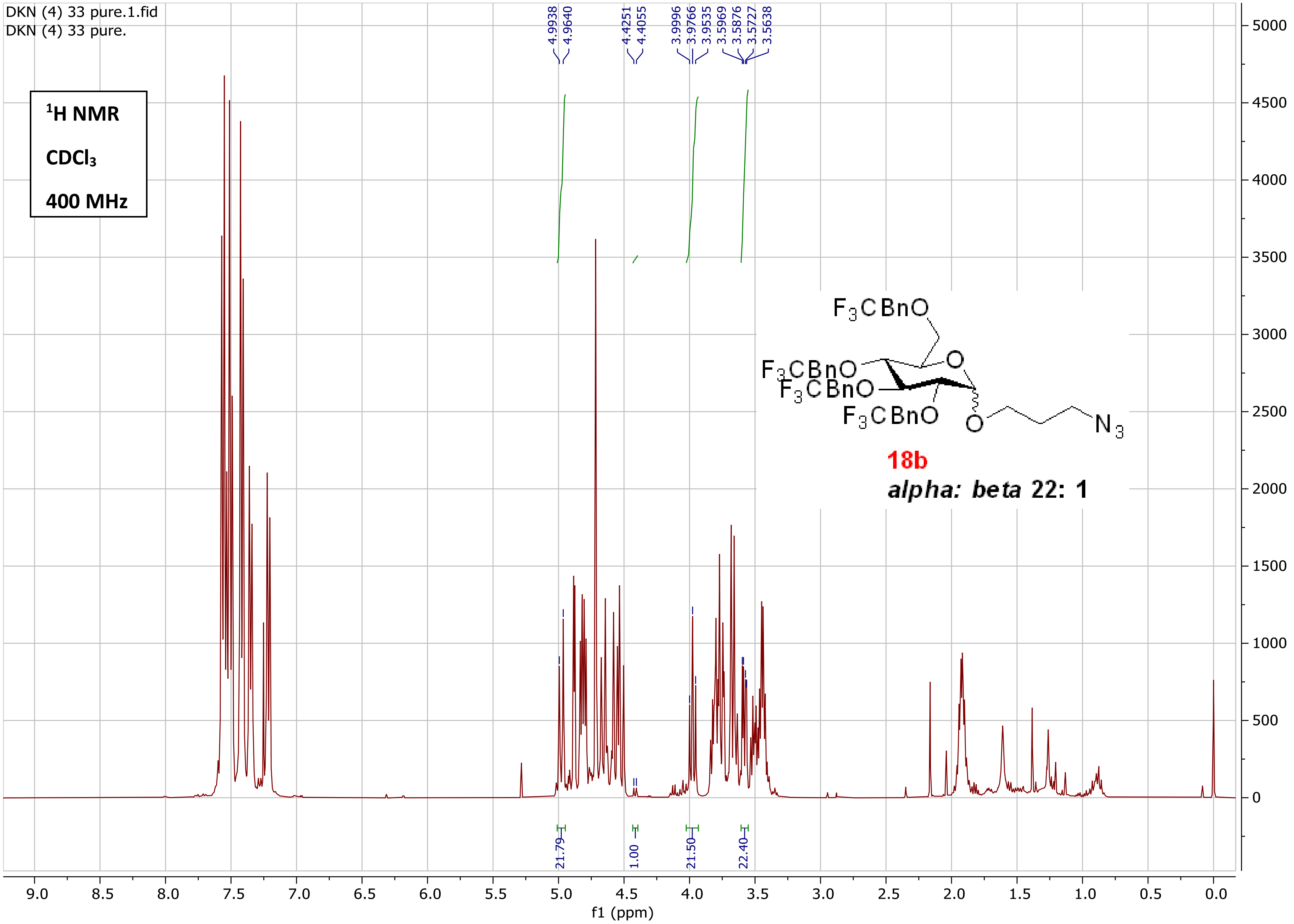
DKN (4) 33 pure.1.fid
DKN (4) 33 pure.
1H NMR
CDCl3
400 MHz
F3CBnO
F3CBnO
F3CBnO
F3CBnO
O
O
N3
18b
alpha: beta 22: 1
f1 (ppm)

	ppm	Hz	Intensity	Width	Area	Type	Flags	Impurity/Compound	Annotation
1	-63.46	-29862.9	440.7	4.42	3530.55	Compound	None		
2	-63.50	-29882.7	14854.7	4.56	118413.71	Compound	None		

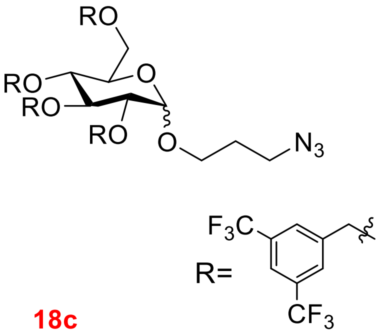

CDCl$_3$, 471 MHz

18c

^{19}F NMR *alpha: beta* 34: 1

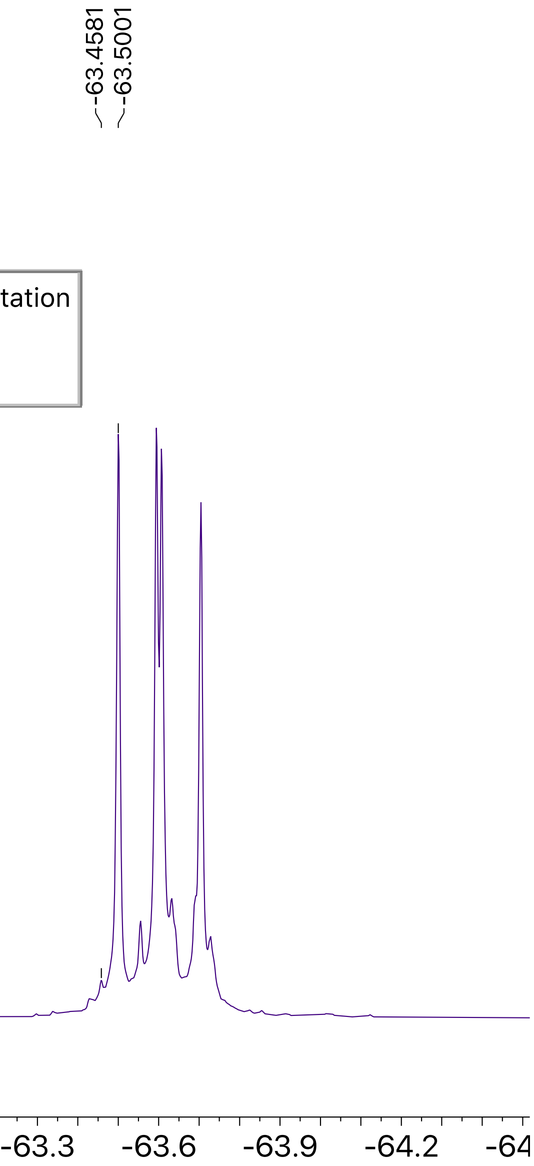

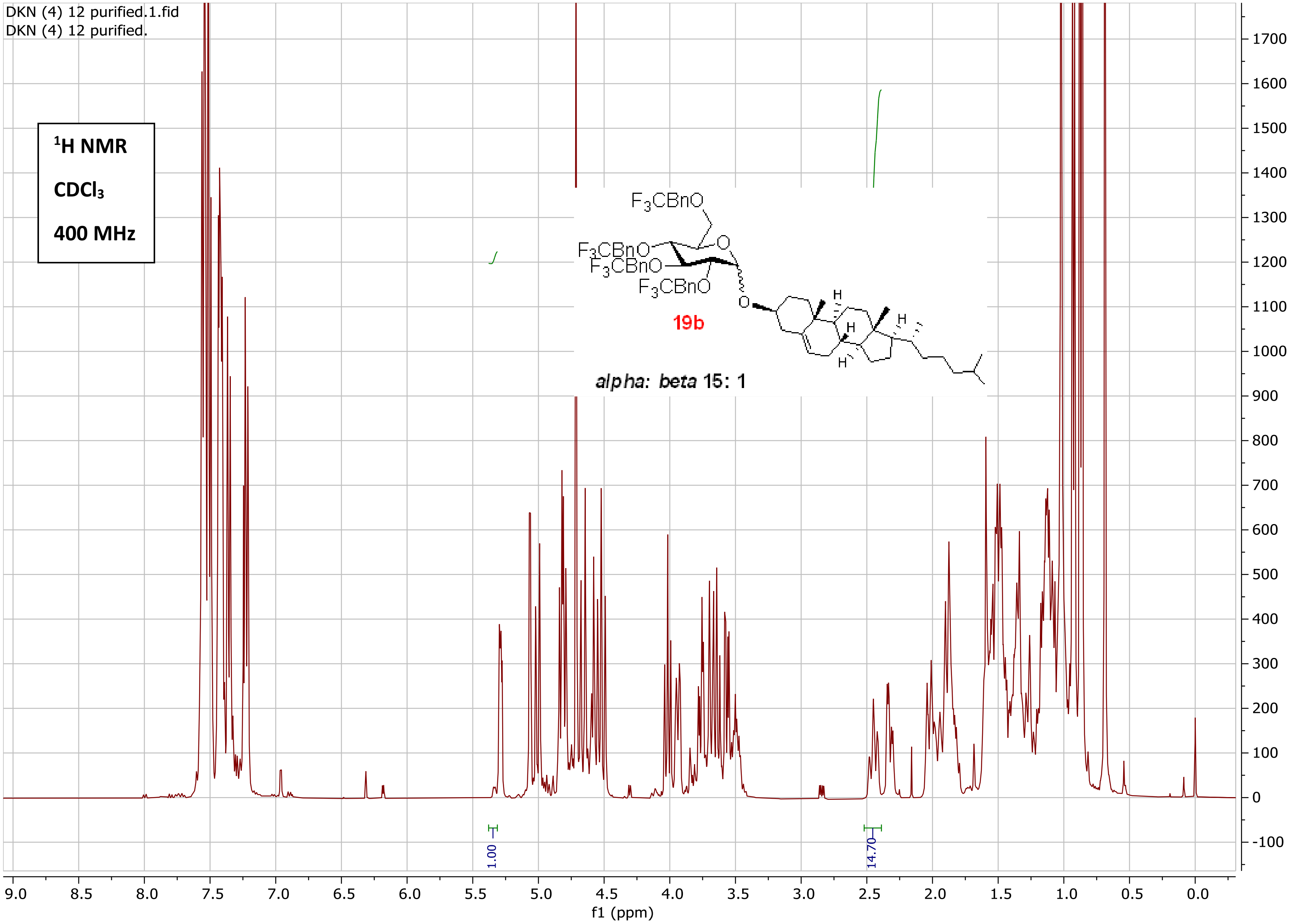

DKN (4) 12 purified.1.fid
DKN (4) 12 purified.
¹H NMR
CDCl3
400 MHz
F3CBnO
F3CBnO
F3CBnO
F3CBnO
O
O
H
H
H
H
19b
alpha: beta 15: 1
1.00
14.70
f1 (ppm)

-63.4106
-63.4675

	ppm	Hz	Intensity	Width	Area	Type	Flags	Impurity/Compound	Annotation
1	-63.41	-29840.5	1053.2	3.26	5583.80	Compound	None		
2	-63.47	-29867.3	23528.3	3.27	129895.74	Compound	None		

CDCl$_3$,
471 MHz

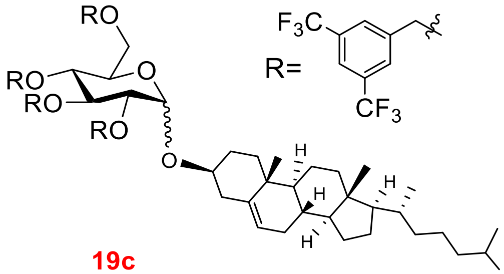

19c

^{19}F NMR *alpha: beta* 23:1

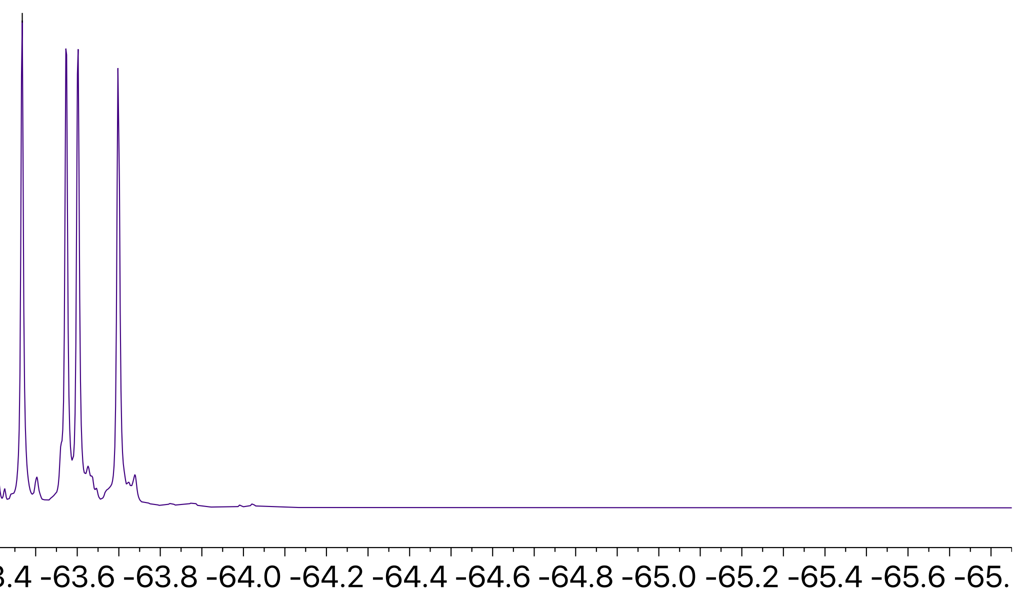

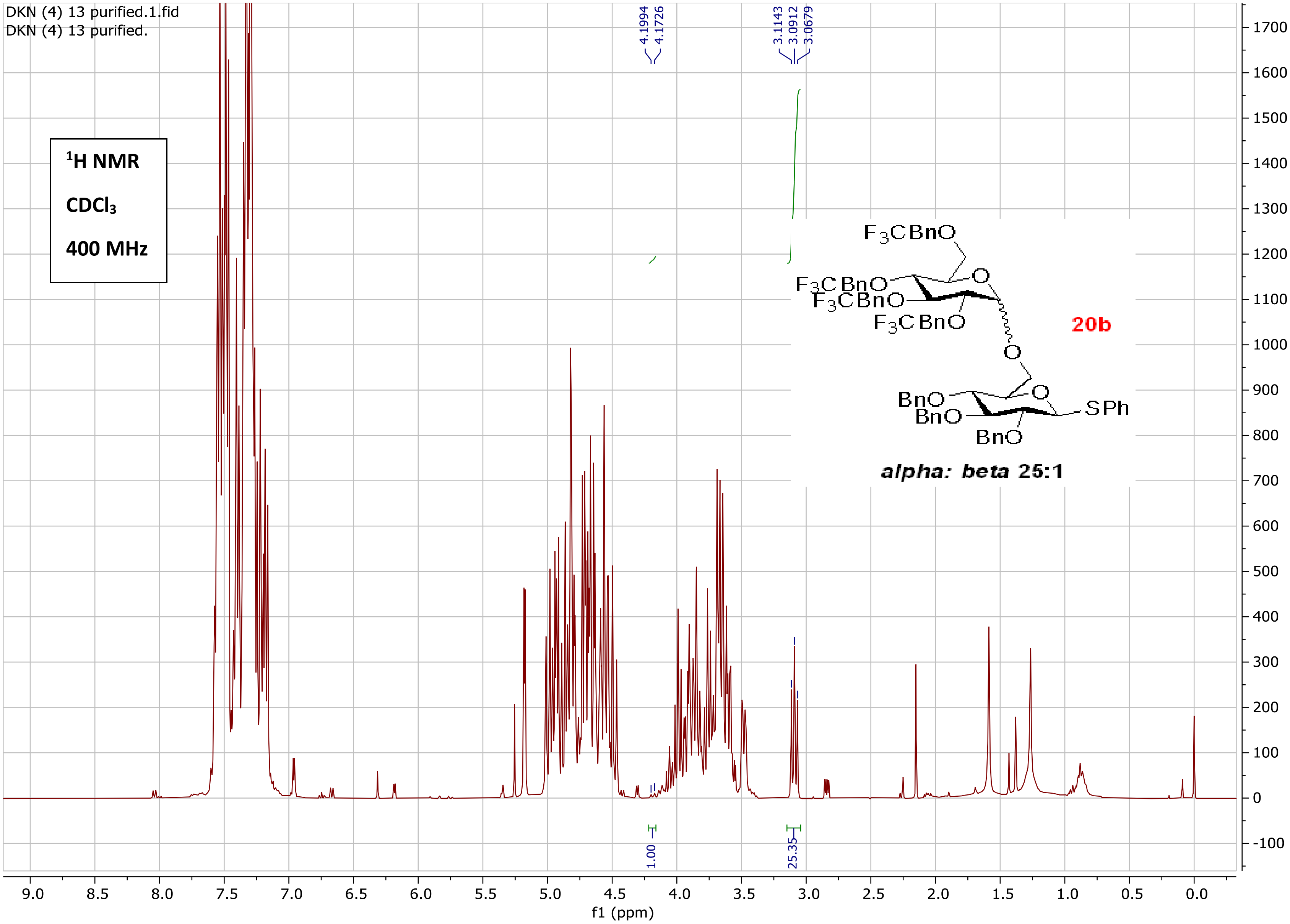
DKN (4) 13 purified.1.fid
DKN (4) 13 purified.
¹H NMR
CDCl₃
400 MHz
4.1994
4.1726
3.1143
3.0912
3.0679
F3CBnO
F3CBnO
F3CBnO
F3CBnO
O
O
O
BnO
BnO
BnO
SPh
20b
alpha: beta 25:1
1.00
25.35
f1 (ppm)

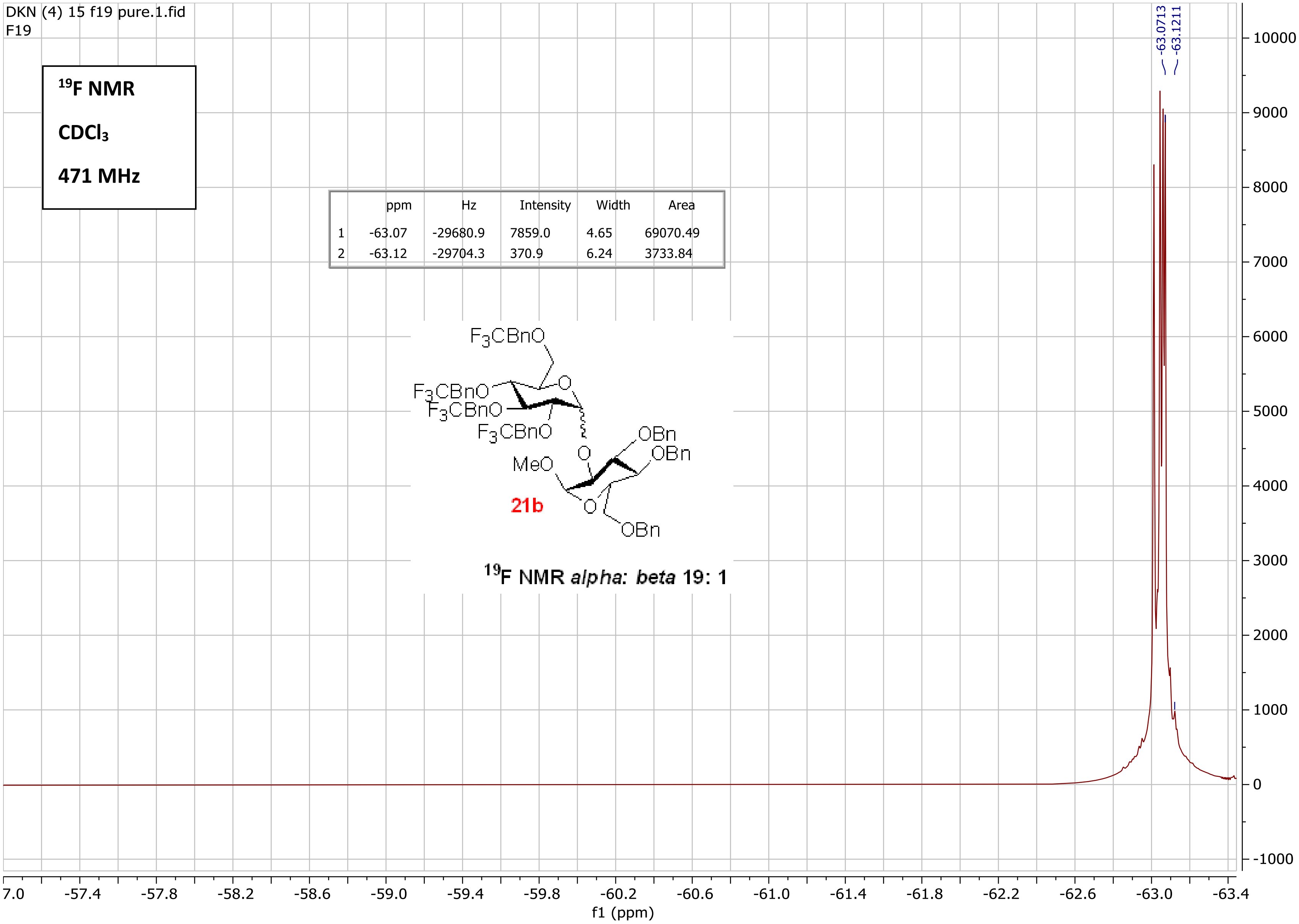

^{19}F NMR

$CDCl_3$

471 MHz

	ppm	Hz	Intensity	Width	Area
1	-63.07	-29680.9	7859.0	4.65	69070.49
2	-63.12	-29704.3	370.9	6.24	3733.84

21b

^{19}F NMR *alpha: beta* 19: 1

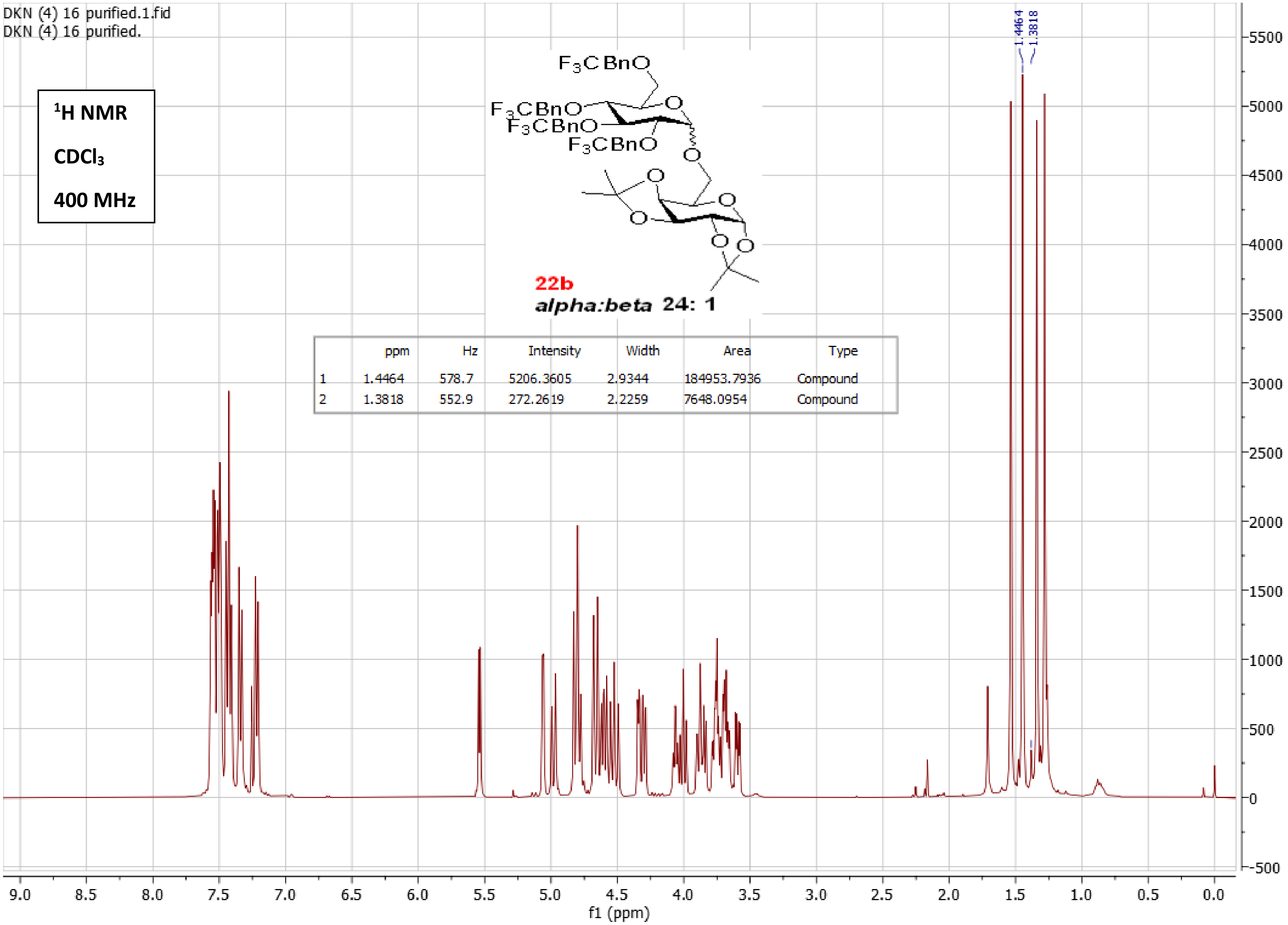

	ppm	Hz	Intensity	Width	Area	Type
1	1.4464	578.7	5206.3605	2.9344	184953.7936	Compound
2	1.3818	552.9	272.2619	2.2259	7648.0954	Compound

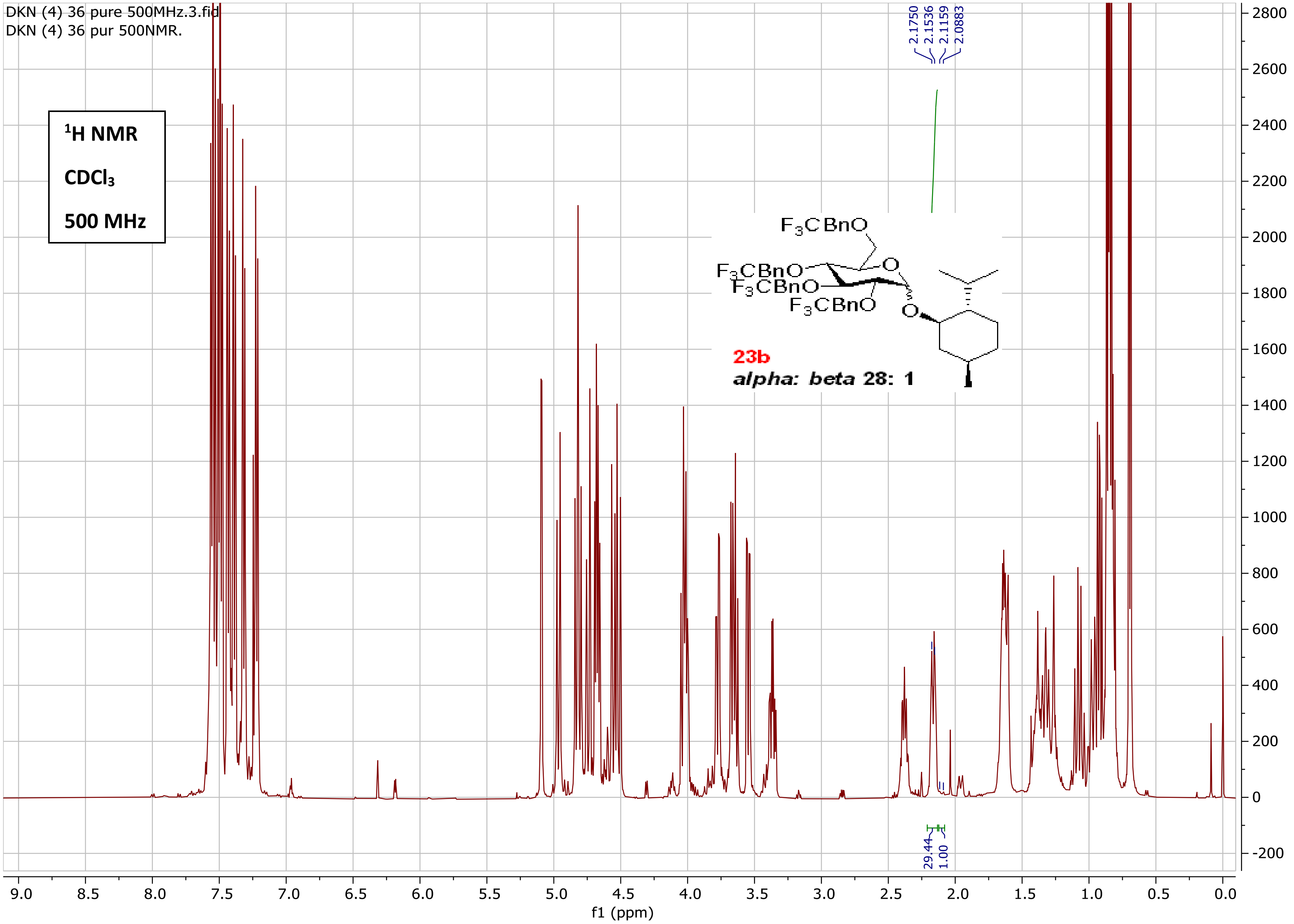

DKN (4) 36 pure 500MHz.3.fid
DKN (4) 36 pur 500NMR.
1H NMR
CDCl3
500 MHz
2.1750
2.1536
2.1159
2.0883
F3CBnO
F3CBnO
F3CBnO
F3CBnO
O
O
23b
alpha: beta 28: 1
29.44
1.00
f1 (ppm)

1 mmol-scale

glycosylation

^{1}H NMR

$CDCl_3$

400 MHz

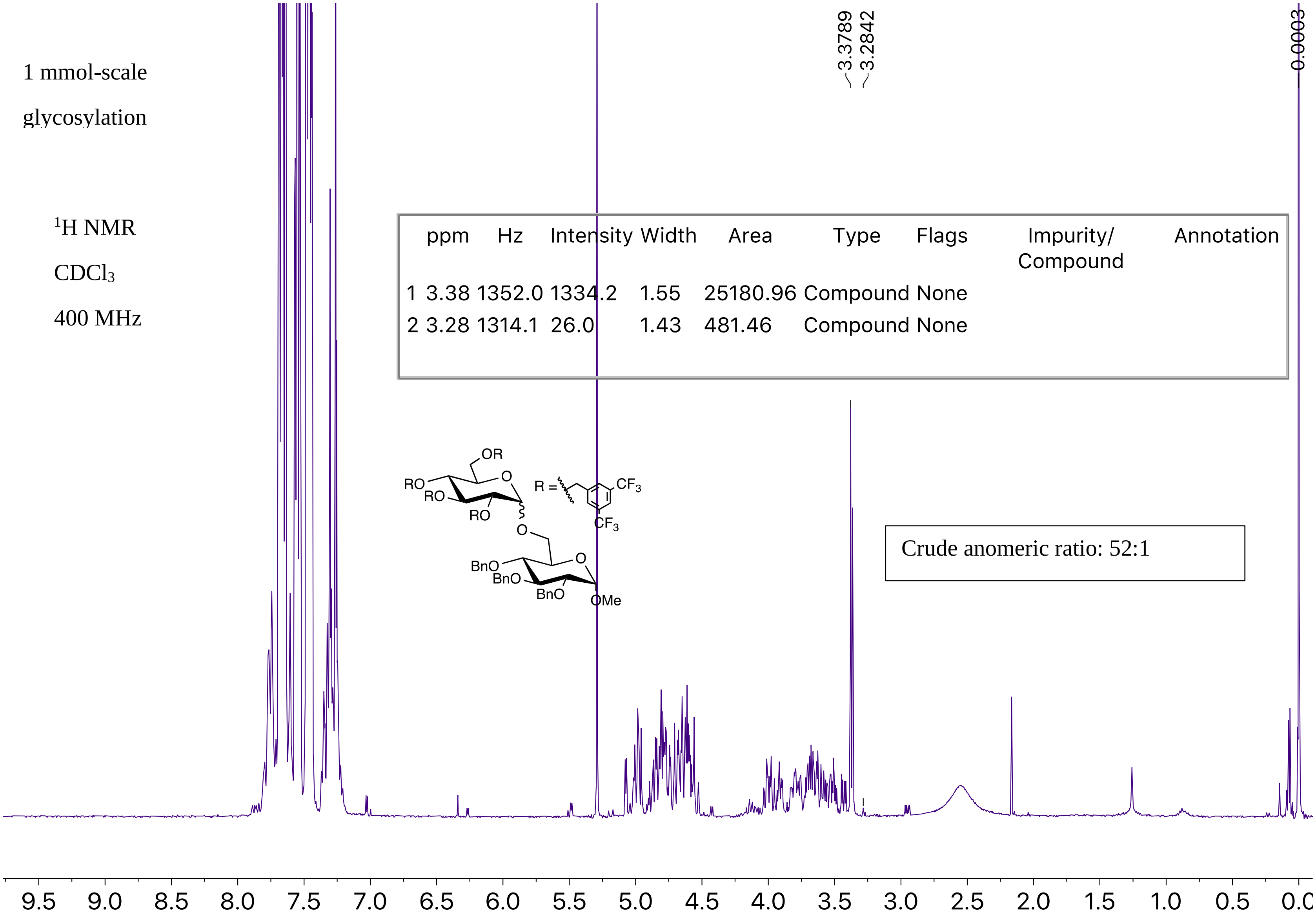

	ppm	Hz	Intensity	Width	Area	Type	Flags	Impurity/ Compound	Annotation
1	3.38	1352.0	1334.2	1.55	25180.96	Compound	None		
2	3.28	1314.1	26.0	1.43	481.46	Compound	None		

Crude anomeric ratio: 52:1

1 mmol-scale glycosylation

^{1}H NMR

$CDCl_3$

400 MHz

	ppm	Hz	Intensity	Width	Area	Type	Flags	Impurity/Compound	Annotation
1	3.41	1363.9	5175.7	1.40	91207.35	Compound	None		
2	3.31	1325.3	109.1	1.40	1913.85	Compound	None		

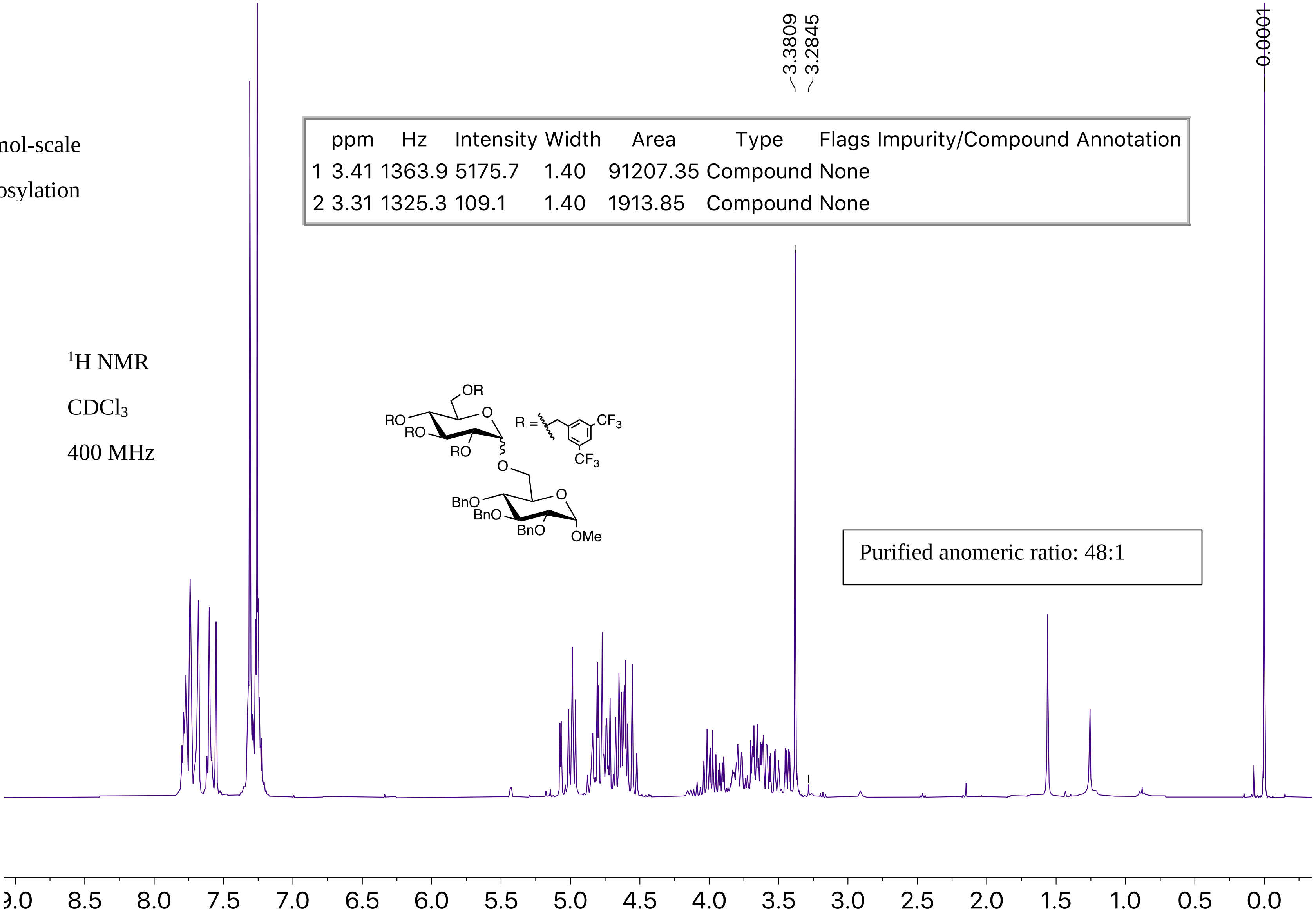

Purified anomeric ratio: 48:1

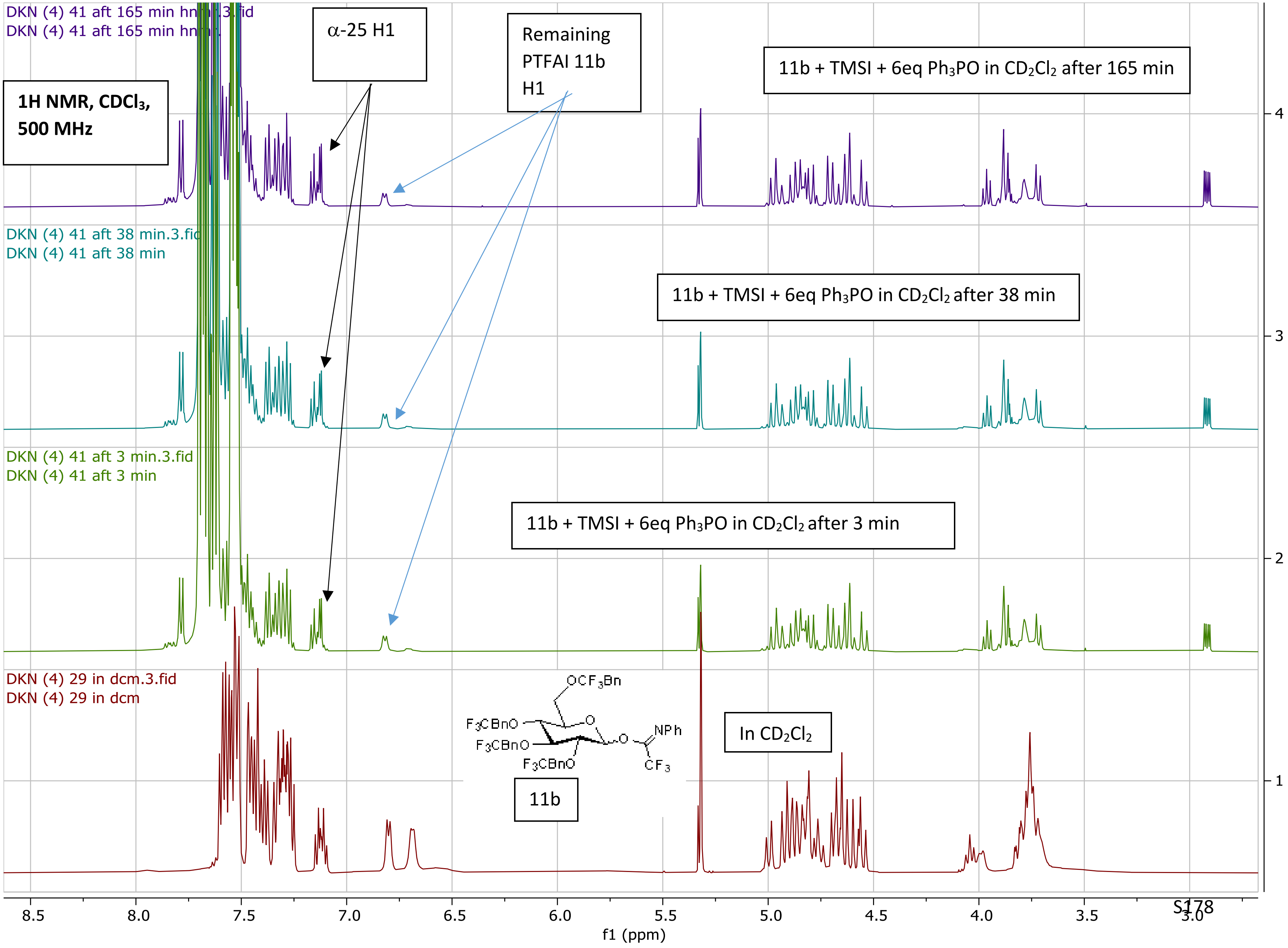
DKN (4) 41 aft 165 min hnmr.3.fid
DKN (4) 41 aft 165 min hnmr
α-25 H1
Remaining PTFAI 11b H1
11b + TMSI + 6eq Ph3PO in CD2Cl2 after 165 min
1H NMR, CDCl3, 500 MHz
DKN (4) 41 aft 38 min.3.fid
DKN (4) 41 aft 38 min
11b + TMSI + 6eq Ph3PO in CD2Cl2 after 38 min
DKN (4) 41 aft 3 min.3.fid
DKN (4) 41 aft 3 min
11b + TMSI + 6eq Ph3PO in CD2Cl2 after 3 min
DKN (4) 29 in dcm.3.fid
DKN (4) 29 in dcm
OCF3Bn
F3CBnO
F3CBnO
F3CBnO
O
O
NPh
CF3
11b
In CD2Cl2
8.5
8.0
7.5
7.0
6.5
6.0
5.5
5.0
4.5
4.0
3.5
3.0
f1 (ppm)
4
3
2
1
